# Synthesis of Oligosaccharides Resembling the *Streptococcus suis* Serotype 18 Capsular Polysaccharide as a Basis for Glycoconjugate Vaccine Development

**DOI:** 10.1021/acs.orglett.2c00596

**Published:** 2022-03-21

**Authors:** Rajat Kumar Singh, Julinton Sianturi, Peter H. Seeberger

**Affiliations:** † Department of Biomolecular Systems, Max Planck Institute of Colloids and Interfaces, Am Mühlenberg 1, 14476 Potsdam, Germany; ‡ Institute of Chemistry and Biochemistry, Freie Universität Berlin, Arnimallee 22, 14195 Berlin, Germany

## Abstract

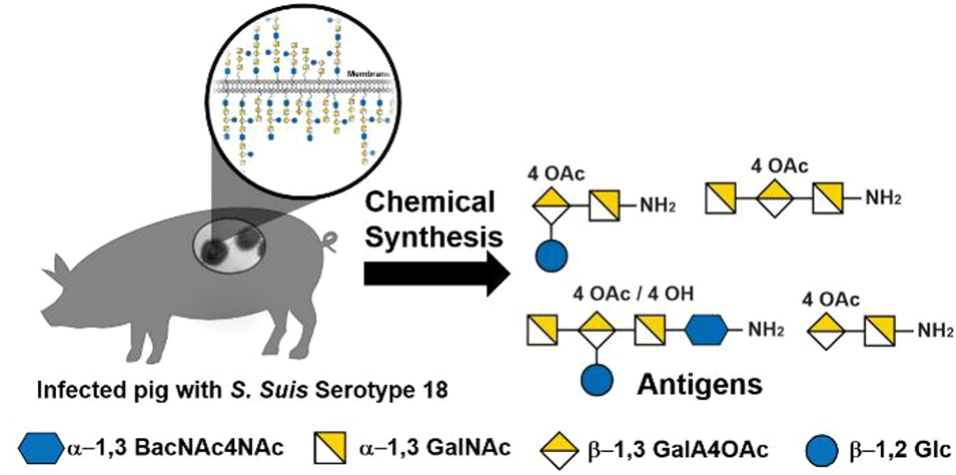

Here we report the first total synthesis of several oligosaccharides resembling the capsular polysaccharide of swine pathogen *S. suis* serotype 18 repeating unit [→3)-d-GalNAc(α1-3)[d-Glc(β1-2)]-d-GalA4OAc(β1-3)-d-GalNAc(α1-3)-d-BacNAc4NAc(α1→]_*n*_. Access to the pentasaccharide repeating unit antigen proved to be very challenging due to the poor reactivity in the context of the trisaccharide. The challenge was overcome by the creation of a galacturonic acid in a late stage of the synthesis.

*Streptococcus suis* (*S. suis*) infections of farmed pigs cause serious economic losses, and humans have been increasingly infected by these antibiotic-resistant bacteria.^1−3^ Capsular polysaccharides (CPSs) surrounding Gram-negative bacteria are the basis of very successful glycoconjugate vaccines against the human pathogen *Streptococcus pneumoniae*. The vaccination of pigs and humans against *S. suis* to prevent rather than treat the disease would avoid the use of antibiotics and reduce the development of antibiotic resistance. Thirty-five *S. suis* serotypes can be distinguished based on their CPS structures. A glycoconjugate vaccine candidate against *S. suis* serotype 2 (SS2) based on isolated CPS^4^ and semisynthetic glycoconjugate vaccine candidates for SS2, SS3, SS9, and SS14 have been evaluated.^4,5^ The *S. suis* serotype 18 CPS pentasaccharide repeating unit provides an interesting challenge for synthetic chemists as a first step toward a glycoconjugate vaccine for this serotype. The SS18 pentasaccharide repeating unit made up of [→3)-d-GalNAc(α1-3)[d-Glc-(β1-2)]d-GalA4OAc(β1-3)-d-GalNAc(α1-3)-d-BacNAc4NAc(α1→]_*n*_ (Figure 1a)^6^ requires the installation of a 1,2-*cis* linkage between d-bacillosamine and the reducing-end linker. The central galacturonic acid branching unit is a challenge concerning the poor reactivity and protecting group orthogonality.

**Figure 1:**
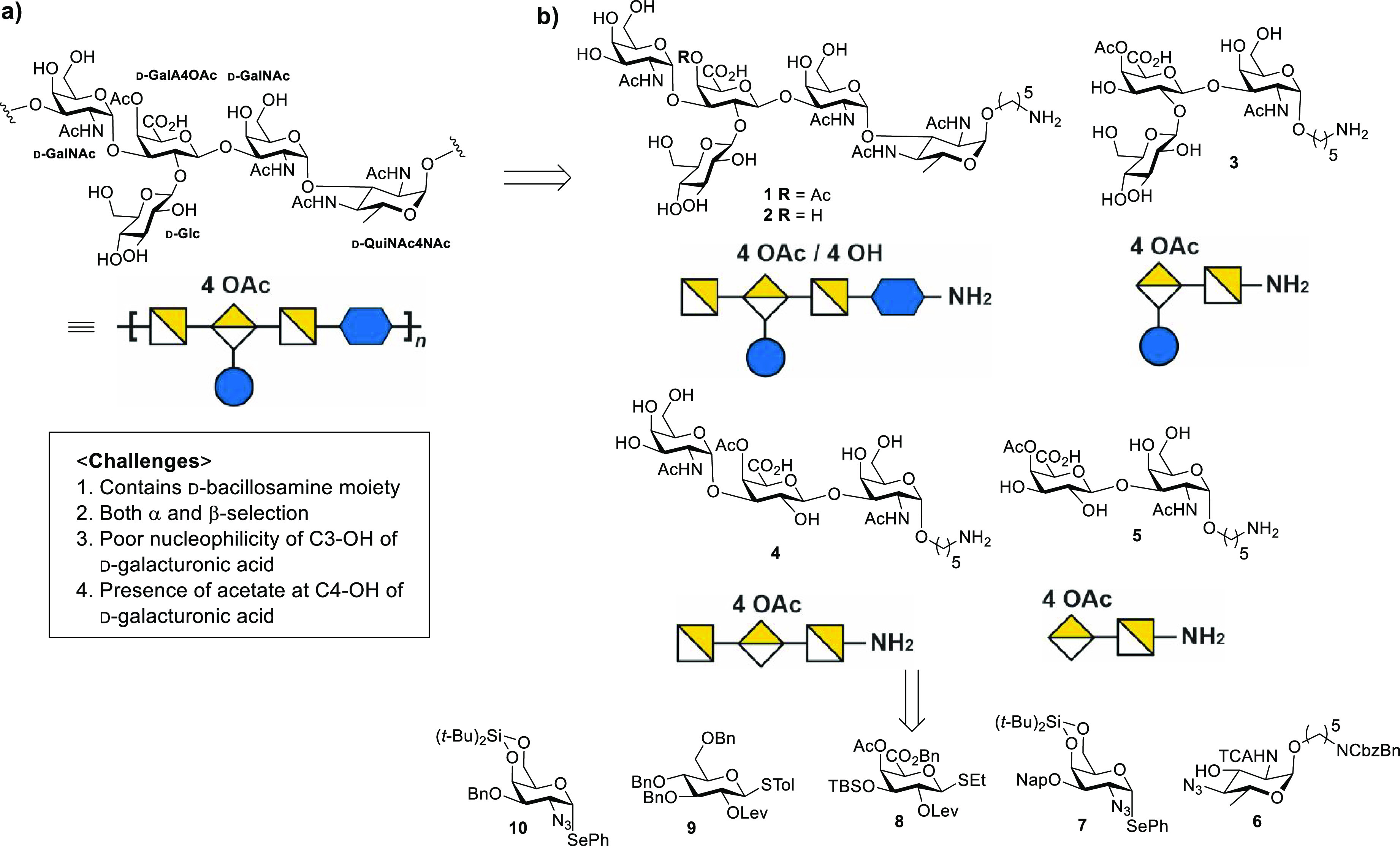
(a) Repeating unit of the *S. suis* serotype 18 CPS. (b) Retrosynthetic analysis of target oligosaccharides **1**–**5**.

The SS18 pentasaccharide repeating unit (Figure 1a) contains the rare deoxy amino sugar d-bacillosamine in addition to d-galactosamine, d-galacturonic acid, d-glucose, and d-galactose. A linear approach will require the synthesis of the rare sugar d-bacillosamine derivative building block and the stereocontrolled 1,2-*cis* linkage between the d-bacillosamine derivative and the linker subsequently used for conjugation. The central galacturonic acid will have to be glycosylated twice at the C2 and C3 positions. The presence of C4-OAc at the d-galacturonic acid of pentasaccharide **1** complicates the synthesis by not allowing the use of most ester protecting groups. Target pentasaccharides **1** and oligosaccharides **2**–**5** resembling different portions of the CPS repeating unit can be prepared using a linear synthesis strategy with five building blocks **6**–**10** (Figure 1b).

d-Bacillosamine derivative **6** (Scheme 1) was synthesized starting from d-galactosamine building block^7^**7** and was used to glycosylate the protected reducing end linker **11** using NIS/TMSOTf as a promoter to afford the exclusively α-linked glycoside **12** in 68% yield. The bulky alkyl substituents of the 4,6-*O*-silylidene group prevent the attack of the nucleophile from the β-face of the donor, combined with through-space electron donation that stabilizes the oxocarbenium-like intermediate^8^ to ensure the complete stereoselectivity of the glycosylation. Silylidene removal using HF in pyridine^9^ yielded dihydroxy galactosamine derivative **13** (96%) followed by tosylation of the primary C6 hydroxyl^10^ to give **14** in 95% yield. C6-Deoxygenation was achieved via iodination with NaI in refluxing acetone (93% yield of **15**), and subsequent dehalogenation/reduction with tributyltin hydride yielded fucosamine^11^ derivative **16** (76%). Selective acylation of the amine in **16** using trichloroacetyl choride^12^ afforded **17** in 85% yield. The triflation of **17** using triflic anhydride followed by C4 inversion with stoichiometric amounts of sodium azide provided d-bacillosamine derivative^13^**18** in 68% yield over two steps. Oxidative cleavage of the naphthyl ether (Nap) by 2,3-dichloro-5,6-dicyano-1,4-benzoquinone (DDQ) afforded d-bacillosamine derivative^14^ building block **6** in 90% yield.

**Scheme 1:**
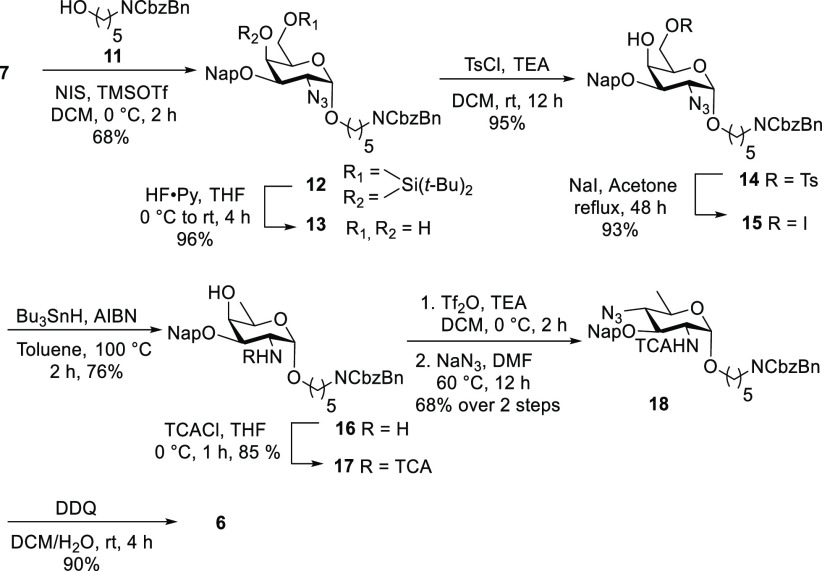
Synthesis of d-Bacillosamine Derivative Acceptor **6**

Galacturonic acid building block **8** was prepared from differentially protected galactose thioglycoside **19** (Scheme 2).^15^ Levulinoylation, followed by benzylidene acetal hydrolysis,^16^ gave dihydroxy galactose thioglycoside **21**. Selective oxidation of the primary C6 alcohol to the carboxylic acid^17^ using TEMPO and subsequent benzylation followed by acetylation gave rise to d-galacturonic acid thioglycoside **8** in 68% yield over three steps. Glucose building block **9** was synthesized in one step from a known thioglycoside **S1**.^18^ (See the Supporting Information.)

**Scheme 2:**
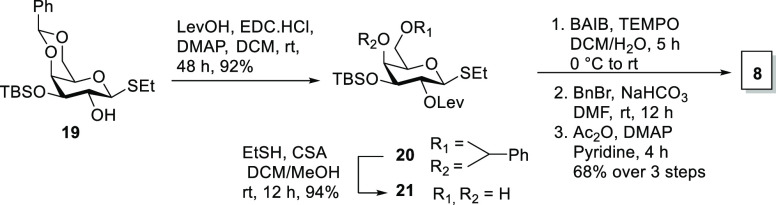
Synthesis of Differentially Protected Galacturonic Acid Building Block **8**

The oligosaccharide assembly commenced with the NIS/TMSOTf-promoted union of d-bacillosamine derivative **6** and d-galactosamine **7**. Removal of the silylidene group by treatment with HF·Py followed by benzylation gave the differentially protected disaccharide **24** (Scheme 3). Oxidative removal of the naphthyl ether using DDQ furnished disaccharide acceptor **25** in 85% yield. The glycosylation of disaccharide **25** using galacturonic acid **8** afforded the protected trisaccharide. The subsequent cleavage of the silyl ether using HF·Py proved difficult and furnished a complex mixture of products. Desilylation using BF_3_·Et_2_O was successful and produced the desired acceptor^19^**26** in 56% yield over two steps. Several attempts to synthesize tetrasaccharide **28** by the glycosylation of trisaccharide acceptor **26** using selenoglycoside **10** and the corresponding trichloroacetimidate **27**(20,21) were not met with success. The poor nucleophilicity of the free hydroxyl group of **26** is a result of the electron-withdrawing groups at C4 and C6, which rendered glycosylations doomed to failure.

**Scheme 3:**
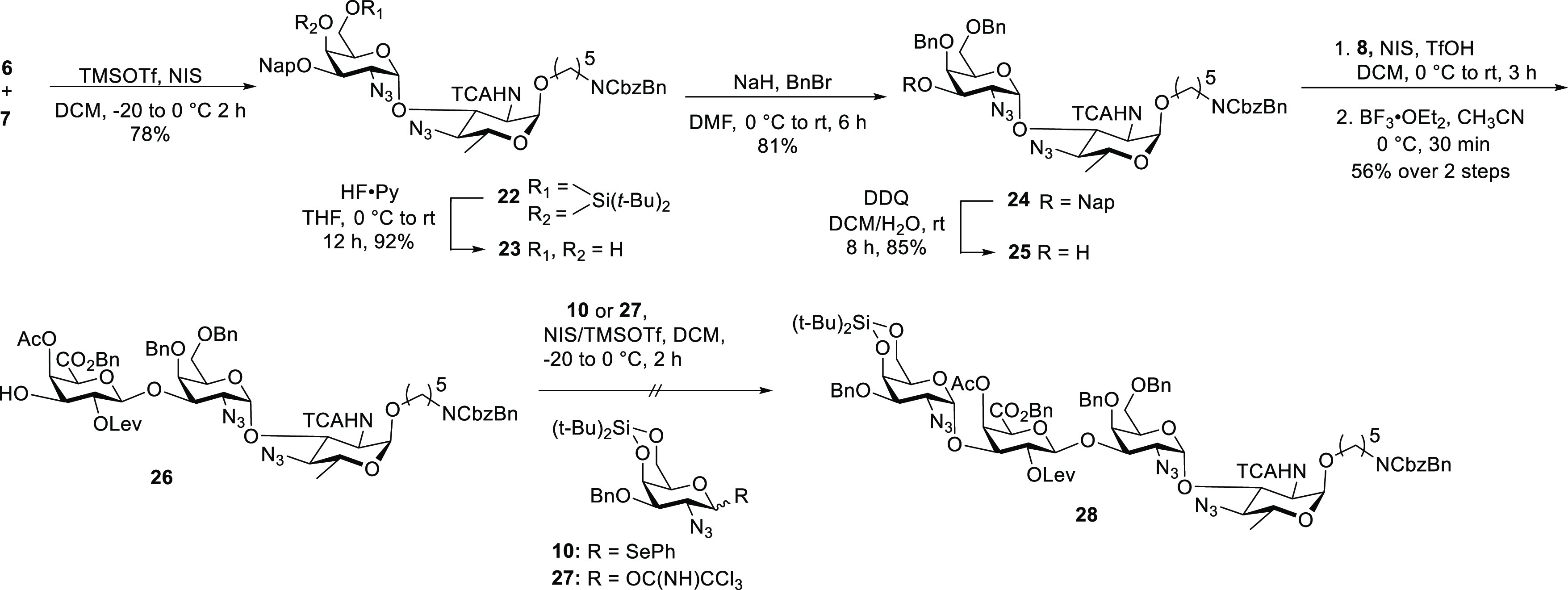
Attempted Synthesis of Tetrasaccharide **28**

A less direct method using galactose in place of galacturonic acid had to be explored to overcome the reactivity problems associated with the low nucleophilicity of the central galacturonic acid unit. The glycosylation of disaccharide **25** with galactose building block **20** afforded trisaccharide **29** in 94% yield, which was liberated from the silyl ether protective group to furnish trisaccharide acceptor **30**. The union of trisaccharide **30** and selenoglycoside **10** followed by the cleavage of the levulinoyl ester using hydrazine acetate afforded tetrasaccharide **32**. The glycosylation of acceptor **32** with glucosamine building block **9** produced pentasaccharide **33** in 64% yield. The central galacturonic acid moiety was prepared by the camphorsulfonic acid (CSA)-mediated hydrolysis of the benzylidene acetal followed by BAIB/TEMPO oxidation, and the selective benzylation of carboxylic acid afforded pentasaccharide **35**. The azide of pentasaccharide **35** was converted into the corresponding acetamide using zinc powder in a THF/Ac_2_O/AcOH mixture; subsequently, silylidene ether and the levulinoyl ester group were deprotected, and the hydrogenation reaction provided the *S. suis* serotype 18 CPS resembling the repeating unit pentasaccharide target **2** in 11% yield over four steps. In addition, pentasaccharide **35** was acetylated followed by azide conversion to acetamide, and the subsequent removal of silylidene and levulinoyl ester and the hydrogenation reaction provided the *S. suis* serotype 18 CPS repeating unit pentasaccharide target **1** in 15% yield over five steps^22^ (Scheme 4).

**Scheme 4:**
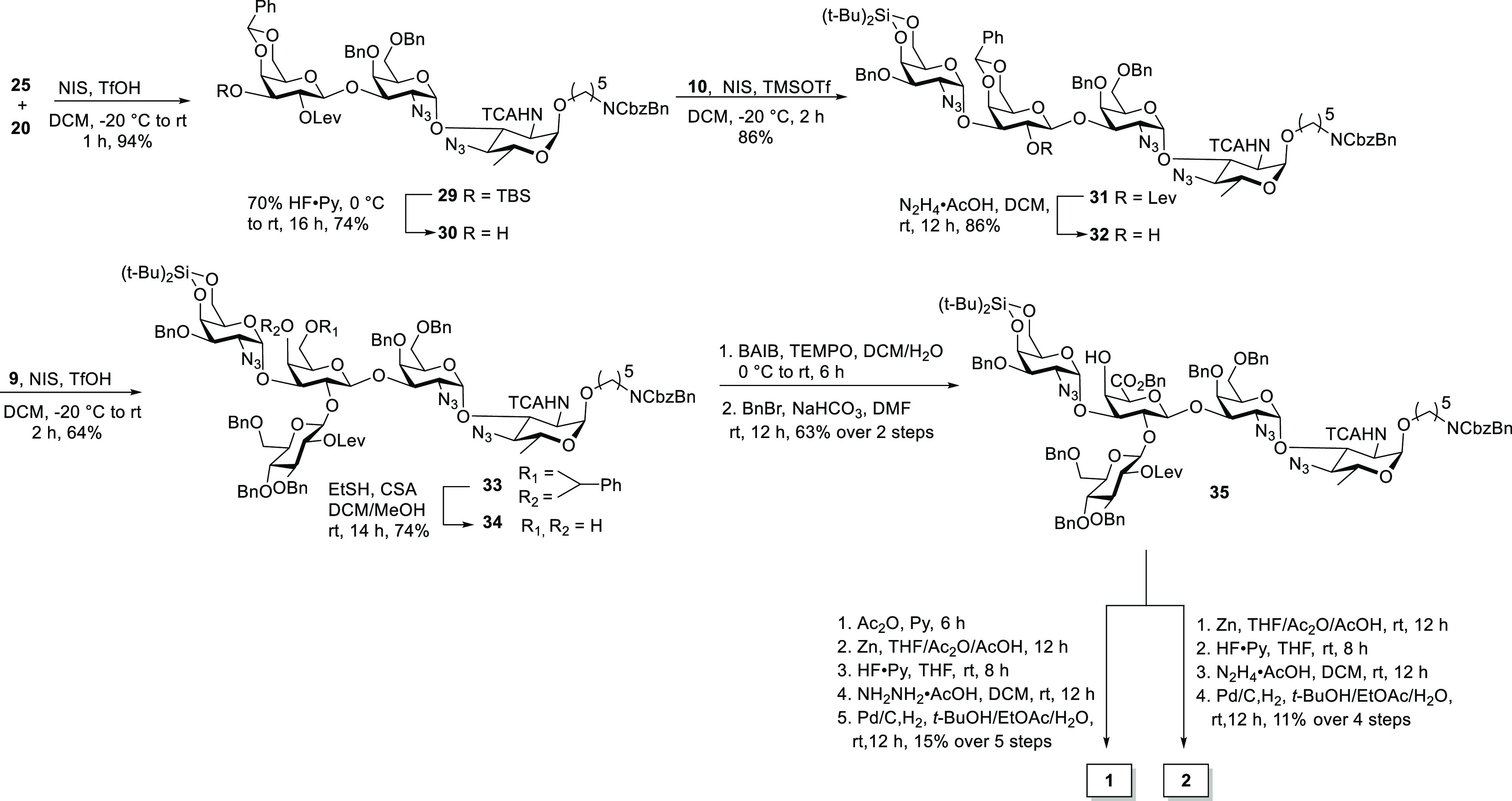
Synthesis of Unprotected Pentasaccharides **1** and **2**

Three oligosaccharides (**3**, **4**, and **5**) that will be essential for subsequent immunological studies to identify the minimally protective glycan epitope were prepared using a divergent synthesis approach (Scheme 5). The benzylation of galactosamine diol **13** afforded **36** (see the Supporting Information), which was freed from the Nap ether to provide **37**. The glycosylation of monosaccharide **37** with galacturonic acid building block **8** exclusively furnished the β-isomer of disaccharide **38** in 75% yield. Levulinoyl ester cleavage using hydrazine acetate afforded **39**; then, glycosylation with **9** in the presence of TfOH and *N*-iodosuccinimide (NIS) at −20 °C produced trisaccharide **40** in 68% yield. The cleavage of levulinoyl ester and *tert*-butyldimethylsilyl (TBS) ether afforded diol **41**. Conversion of the azide to the corresponding acetamide using Zn/AcOH/Ac_2_O followed by hydrogenation afforded trisaccharide **3** (46% yield over two steps). Trisaccharide **4** was prepared by the TBS removal of **38** in preparation for glycosylation with **10** to furnish trisaccharide **43**. The global deprotection of **43** produced the desired trisaccharide **4**. Disaccharide **5** was readily accessible by deprotection of disaccharide **39** in 46% yield over three steps.

**Scheme 5:**
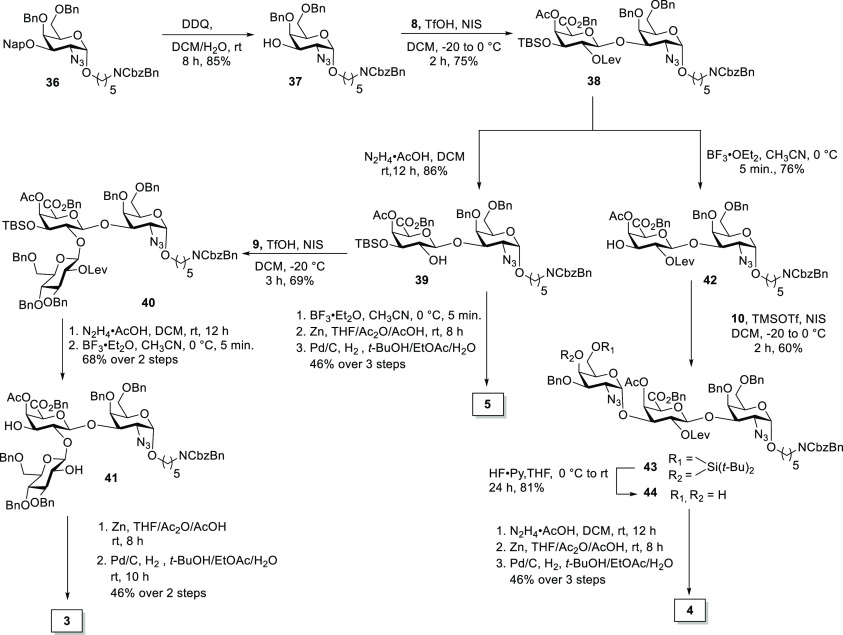
Synthesis of Oligosaccharides Resembling *S. suis* Serotype 18

In conclusion, we report the total synthesis of several oligosaccharides resembling the CPS of swine pathogen *S. suis* serotype 18 that are the basis for immunological studies and the development of a glycoconjugate vaccine. The rare d-bacillosamine derivative was prepared from d-galactosamine using tin-mediated reduction and dehalogenation. Access to the pentasaccharide repeating unit antigen proved to be very challenging due to the poor reactivity of the trisaccharide intermediate. The challenge was overcome by the creation of galacturonic acid in a late stage of the synthesis. The conjugation-ready glycans prepared using the total synthesis approach will be used for immunological studies.

## Supporting Information Available

Complete experimental procedures and NMR spectra of synthetic compounds (PDF)
