# Supplementary material for: Synthesis of Oligosaccharides Resembling the Streptococcus suis Serotype 18 Capsular Polysaccharide as a Basis for Glycoconjugate Vaccine Development
Source: Org Lett. 2022 Mar 21;24(12):2371–5. doi: 10.1021/acs.orglett.2c00596 (PMC8981331; doi:10.1021/acs.orglett.2c00596)
Supplement: Supplementary file 1 — ol2c00596_si_001.pdf [file ol2c00596_si_001.pdf]

Synthesis of Oligosaccharides Resembling the *Streptococcus suis* Serotype 18 Capsular Polysaccharide as Basis for Glycoconjugate Vaccine Development

Rajat Kumar Singh[a][b], Julinton Sianturi[a] and Peter H. Seeberger[a][b]

a] Department of Biomolecular Systems, Max Planck Institute of Colloids and Interfaces, Am Mühlenberg 1, 14476 Potsdam, Germany

[b] Institute of Chemistry and Biochemistry, Freie Universität Berlin, Arnimallee 22, 14195 Berlin, Germany

General Information

All the glassware were dried in the oven prior to reaction. Commercial grade solvents and reagents were used without further purification. Reactions sensitive to moisture were carried out under an atmosphere of nitrogen. Sodium iodide (NaI) used in the reaction was dried at 80 °C under vacuum, sugar building blocks indicated as commercially available were purchased from GlycoUniverse GmbH. Anhydrous solvents were obtained from a solvent drying system (JCMeyer) or dried according to reported procedures. Analytical TLC was performed on Kieselgel 60 F_{254} glass (Macherey-Nagel). Spots were visualized with UV light (λ: 254 nm), sulphuric acid stain [1 mL of 3-methoxyphenol in 1 L of EtOH and 30 mL H_2SO_4] orceric ammonium molybdate stain [0.5 g $Ce(NH_4)_4(SO_4)_4$•$2H_2O$, 12 g $(NH_4)_6Mo_7O_{24}$•$4H_2O$ and 15 mL H_2SO_4 in 235 mL H_2O]. Flash chromatography was performed on Kieselgel 60 230-400 mesh (Sigma-Aldrich). Preparative HPLC purifications were performed with an Agilent 1200 Series or Agilent 1260 Infinity II. NMR spectra were recorded on a Varian 400 MHz spectrometer (Agilent), Ascend 400 MHz (cryoprobe, Bruker), Ascend 700 MHz (cryoprobe, Bruker) or Varian 600 MHz (Agilent) at 25 °C unless indicated otherwise. Chemical shifts (δ) are reported in parts per million (ppm) relative to the respective residual solvent peaks ($CHCl_3$: δ 7.26 in ^{1}H and 77.16 in ^{13}C; HDO δ 4.79 in ^{1}H). Bidimensional and non-decoupled experiments were performed to assign identities of peaks showing relevant structural features. The following abbreviations are used to indicate peak multiplicities: s (singlet), d (doublet) dd (doublet of doublets), t (triplet), dt (doublet of triplets), td (triplet of doublets), q (quartet), p (pentet), m (multiplet). Additional descriptors b (broad signal) and app (apparent first-order multiplet) are also employed when required. Coupling constants (J) are reported in Hertz (Hz). NMR spectra were processed using MestreNova 14.1 (MestreLab Research).High-resolution mass spectra (ESI-HRMS) were recorded with a Xevo G2-XS Q-Tof (Waters).

N-(Benzyl)benzyloxycarbonyl-5-aminopentyl 2-azido-2-deoxy-3-O-(2-napthylmethyl)-4,6-O-silylidene-α-D-galactopyranoside (12)

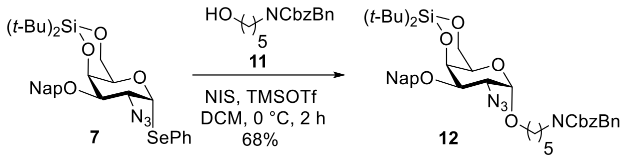

To a solution of donor[1] **7** (2.2 g, 3.52 mmol.), *N*-benzyloxycarbonyl-*N*-benzyl-5-aminopentanol (**11**) (2.31 g, 7.04 mmol) in DCM (20 mL) were added 4Å molecular sieves. After ~30 minutes, the mixture was cooled to 0 °C and *N*-iodosuccinimide (1.03 g, 4.58 mmol) and TMSOTf (63 µL, 0.35 mmol) were added. After TLC analysis indicated complete consumption of the starting material (~2 hours), the reaction was quenched with Et_3N (2 mL) and the mixture was diluted with DCM. After filtration over Celite® 353, the mixture was washed with 10% aqueous $Na_2S_2O_3$, saturated aqueous $NaHCO_3$ and brine, dried over Na_2SO_4, filtered and concentrated *in vacuo*. The crude was purified by silica gel column chromatography using 8% ethyl acetate in hexanes to afford the title product **12** as the sole isomer (1.9 g, 68%). ^{1}H NMR (400 MHz, $CDCl_3$) δ 7.86 – 7.81 (m, 4H), 7.58 (m, 1H), 7.48 (dd, J = 6.4, 3.2 Hz, 2H), 7.38 – 7.24 (m, 9H), 7.17 (d, J = 7.4 Hz, 1H), 5.18 (d, J = 21.1 Hz, 2H), 4.94 – 4.88 (m, 2H), 4.84 (d, J = 11.8 Hz, 1H), 4.61 (d, J = 12.3 Hz, 1H), 4.50 (d, J = 14.6 Hz, 2H), 4.24 (t, J = 14.5 Hz, 1H), 4.13 (m, 1H), 3.90 (dd, J = 17.0, 10.5 Hz, 1H), 3.82 – 3.77 (m, 1H), 3.60 (m, 2H), 3.46 – 3.36 (m, 1H), 3.28 – 3.17 (m, 2H), 1.63 – 1.50 (m, 4H), 1.35 – 1.28 (m, 2H), 1.08 (s, 9H), 1.06 (s, 9H). ^{13}C NMR (101 MHz, $CDCl_3$) δ 156.8, 156.3, 138.0, 136.9, 135.5, 133.4, 133.2, 128.7, 128.6, 128.5, 128.1, 127.9, 127.8, 127.5, 127.4, 127.3, 126.7, 126.2, 126.1, 126.0, 98.4, 75.5, 70.6, 70.0, 68.3, 67.5, 67.3, 58.5, 50.6, 50.3, 47.2, 46.2, 29.8, 29.2, 27.8, 27.5, 23.6, 23.5, 20.9. HR-ESI-MS (m/z): calculated for $C_{45}H_{58}N_4O_7SiNa$ $[M+Na]^+$: 817.3972, found: 817.3978

N-(Benzyl)benzyloxycarbonyl-5-aminopentyl 2-azido-2-deoxy-3-O-(2-napthylmethyl)-α-D-galactopyranoside (13)

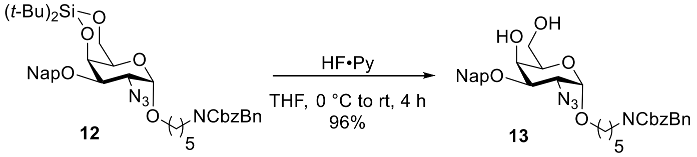

To a solution of **12** (1.64 g, 2.06 mmol) in THF (20 mL) was added HF•Py (70% HF, 2.65 mL, 20.6 mmol). After TLC analysis indicated complete conversion of the starting material (~4 hours), the reaction was quenched with Et_3N (2 mL). The mixture was concentrated, dissolved in EtOAc and subsequently washed with saturated aqueous $NaHCO_3$ and brine. The aqueous layers were then extracted with EtOAc, combined organic layers were dried over Na_2SO_4, filtered and concentrated *in vacuo*. The residue was purified by silica column chromatography using 25% ethyl acetate in hexanes to furnish the title compound **13** (1.3 g, 96%). ^{1}H NMR (400 MHz, $CDCl_3$) δ 7.89 – 7.79 (m, 4H), 7.56 – 7.45 (m, 3H), 7.41 – 7.13 (m, 10H), 5.19 (d, J = 17.3 Hz, 2H), 4.94 – 4.82 (m, 3H), 4.56 – 4.41 (m, 2H), 4.19 (dd, J = 24.3, 3.2 Hz, 1H), 4.01 – 3.54 (m, 6H), 3.48 – 3.11 (m, 3H), 2.95 (bs, 1H), 1.66 – 1.44 (m, 4H), 1.39 – 1.24 (m, 2H). ^{13}C NMR (101 MHz, $CDCl_3$)

δ 156.8, 156.4, 137.8, 136.9, 136.6, 134.6, 133.2, 133.2, 128.6, 128.5, 128.5, 128.0, 128.0, 127.9, 127.8, 127.8, 127.8, 127.4, 127.2, 126.9, 126.4, 126.3, 126.2, 125.7, 98.0, 98.0, 77.5, 77.4, 77.2, 76.8, 76.1, 75.9, 72.0, 69.8, 69.5, 68.2, 67.7, 67.4, 67.3, 67.3, 67.1, 62.8, 62.4, 59.1, 59.0, 50.5, 50.3, 47.2, 46.1, 29.0, 28.9, 27.9, 27.1, 23.3, 23.2. HR-ESI-MS (m/z): calculated for $C_{37}H_{42}N_4O_7Na$ $[M+Na]^+$: 677.2951, found: 677.2966.

_N_-(Benzyl)benzyloxycarbonyl-5-aminopentyl 2-azido-2-deoxy-3-_O_-(2-napthylmethyl)-6-_O_-_p_-toluenesulfonyl–α-D-galactopyranoside (14)

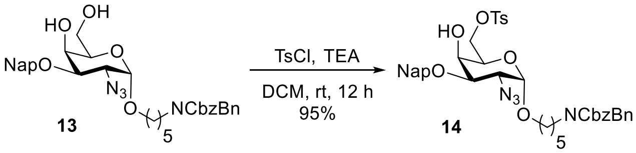

To a solution of **13** (1.3 g, 1.99 mmol) in DCM (15 mL), _p_-toluenesulfonyl chloride (455 mg, 2.39 mmol) and TEA (0.69 mL, 4.98 mmol) were added. The reaction mixture was stirred overnight at rt, when TLC showed that the starting material was completely consumed, diluted with DCM (10 mL), washed with saturated aqueous $NaHCO_3$, dried over Na_2SO_4, filtered and concentrated _in vacuo_. The residue was purified by silica gel column chromatography using 20% ethyl acetate in hexanes to furnish compound **14** (1.53 g, 95%). ^1H NMR (400 MHz, $CDCl_3$) δ 7.89 – 7.81 (m, 4H), 7.79 – 7.74 (m, 2H), 7.54 – 7.46 (m, 3H), 7.41 – 7.24 (m, 11H), 7.19 (d, _J_ = 7.2 Hz, 1H), 5.19 (d, _J_ = 13.3 Hz, 2H), 4.90 – 4.78 (m, 3H), 4.51 (d, _J_ = 8.3 Hz, 2H), 4.26 – 4.12 (m, 2H), 4.06 (d, _J_ = 4.0 Hz, 1H), 4.03 – 3.86 (m, 2H), 3.60 (dd, _J_ = 10.4, 3.6 Hz, 2H), 3.41 – 3.15 (m, 4H), 2.42 (s, 3H), 1.63 – 1.49 (m, 4H, 1.37 – 1.23 (m, 2H). ^{13}C NMR (101 MHz, $CDCl_3$) δ 156.8, 156.3, 145.1, 138.0, 136.8, 134.5, 133.3, 133.2, 132.7, 130.0, 128.7, 128.6, 128.6, 128.5, 128.1, 128.0, 127.9, 127.8, 127.4, 127.3, 127.3, 127.1, 126.5, 126.4, 125.7, 97.9, 77.5, 77.4, 77.2, 76.8, 75.7, 72.3, 68.9, 68.4, 67.9, 67.2, 65.9, 58.9, 50.6, 50.3, 47.2, 46.2, 29.0, 27.9, 27.5, 23.4, 23.3, 21.7. HR-ESI-MS (m/z): calculated for $C_{44}H_{48}N_4O_9SNa$ $[M+Na]^+$: 831.3040, found: 831.3060.

_N_-(Benzyl)benzyloxycarbonyl-5-aminopentyl 2-azido-2,6-di-deoxy-6-iodo-3-_O_-(2-napthylmethyl)-α-D-galactopyranoside (15)

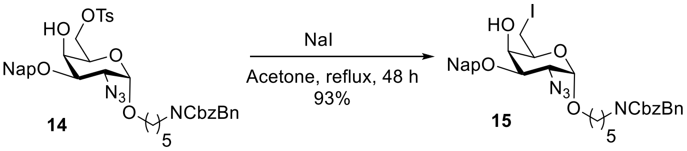

To a solution of **14** (1.53 g, 1.89 mmol) in acetone (25 mL) was added NaI (2.83 g, 18.9 mmol). The mixture was refluxed using oil bath under nitrogen for 48 h. After cooling to rt, ethyl acetate (25 mL) was added and the mixture was washed with saturated aqueous $Na_2S_2O_3$ solution and water. The organic phase was dried over Na_2SO_4, filtered, and concentrated. The residue was purified by silica gel column chromatography using 15% ethyl acetate in hexanes to give product **15** (1.35 g, 93%). ^1H NMR (400 MHz, $CDCl_3$) δ 7.89 – 7.80 (m, 4H), 7.55 – 7.47 (m, 3H), 7.40 – 7.23 (m, 10H), 7.17 (d, _J_ = 7.2 Hz, 1H), 5.18 (d, _J_ = 14.4 Hz, 2H), 4.88 (q, _J_ = 11.5 Hz, 3H), 4.50 (d, _J_ = 9.4 Hz, 2H), 4.23 – 4.15 (m, 1H), 3.99 – 3.82 (m, 2H), 3.81 – 3.68 (m, 1H), 3.63 (dd, _J_ = 10.4, 3.6 Hz, 1H), 3.48 – 3.36 (m, 1H), 3.32 (t, _J_ = 4.7 Hz, 2H), 3.30 – 3.16 (m, 2H), 1.67 – 1.47

(m, 3H), 1.40 –1.28(m, 2H). ^{13}C NMR (101 MHz, $CDCl_3$) δ 156.9, 156.3, 138.0, 138.0, 137.0, 136.8, 134.5, 133.3, 128.8, 128.7, 128.6, 128.6, 128.4, 128.2, 128.1, 128.1, 127.9, 127.9, 127.5, 127.4, 127.3, 127.2, 126.5, 126.4, 125.8, 97.9, 77.5, 77.4, 77.2, 76.8, 72.5, 70.7, 68.4, 67.3, 67.2, 58.8, 50.6, 50.3, 47.2, 46.2, 32.1, 29.8, 29.8, 29.5, 29.1, 28.0, 27.6, 23.5, 23.4, 22.8, 14.3, 3.0, 1.2. HR-ESI-MS (m/z): calculated for $C_{37}H_{41}IN_4O_6Na$ $[M+Na]^+$: 787.1968, found: 787.1990

_N_-(Benzyl)benzyloxycarbonyl-5-aminopentyl 2-amino-2,6-di-deoxy-3-_O_-(2-napthylmethyl) -α-D-galactopyranoside (16)

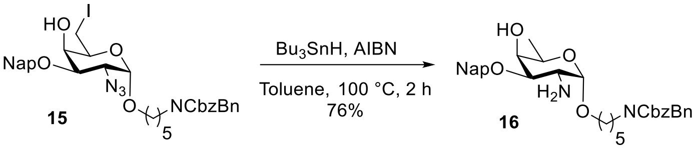

Compound **15** (1.35 g, 1.77 mmol) was co-evaporated with toluene for three times and dried under high vacuum for 8 h. To this, Bu_3SnH (1.05 mL, 3.89 mmol) and AIBN (57 mg, 0.35 mmol) were added and stirred at 100 °C using oil bath for 2 h, after which time the reaction mixture was diluted with EtOAc (30 mL), the organic layer was washed with water (80 mL) and brine (80 mL), dried over Na_2SO_4, filtered and concentrated *in vacuo*. The resultant oil was purified by silica gel column chromatography using 5% methanol in DCM to afford desired compound **16** (821 mg, 76%) as oil. ^{1}H NMR (600 MHz, $CDCl_3$) δ 7.87 – 7.77 (m, 3H), 7.51 – 7.45 (m, 3H), 7.39 – 7.20 (m, 10H), 7.15 (d, *J* = 7.4 Hz, 1H), 5.16 (d, *J* = 24.7 Hz, 2H), 4.88 – 4.79 (m, 2H), 4.70 (d, *J* = 11.2 Hz, 1H), 4.48 (d, *J* = 20.4 Hz, 2H), 3.89 – 3.81 (m, 2H), 3.65 – 3.45 (m, 2H), 3.43 – 3.29 (m, 1H), 3.27 – 3.09 (m, 4H), 2.45 (bs, 2H), 1.57 – 1.45 (m, 4H), 1.32 – 1.19 (m, 5H). ^{13}C NMR (151 MHz, $CDCl_3$) δ 156.8, 156.3, 138.0, 136.8, 135.3, 133.3, 133.2, 130.5, 128.8, 128.6, 128.6, 128.5, 128.5, 128.2, 128.0, 127.9, 127.9, 127.8, 127.7, 127.6, 127.4, 127.3, 126.8, 126.7, 126.4, 126.2, 126.1, 126.0, 125.8, 99.4, 99.3, 80.2, 77.4, 77.4, 77.3, 77.2, 77.1, 76.9, 76.9, 71.5, 69.6, 68.2, 68.0, 67.3, 67.3, 65.8, 50.6, 50.3, 47.2, 46.2, 29.3, 28.0, 27.9, 27.6, 26.9, 23.6, 23.5, 16.6, 13.7. HR-ESI-MS (m/z): calculated for $C_{37}H_{44}N2O_6Na$ $[M+Na]^+$: 635.3097, found: 635.3084

_N_-(Benzyl)benzyloxycarbonyl-5-aminopentyl 2-trichloroacetamido-2,6-di-deoxy-3-_O_-(2-napthylmethyl) -α-D-galactopyranoside (17)

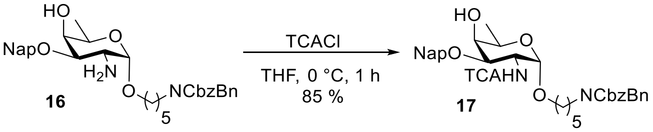

The amine **16** (821 mg, 1.34 mmol) was dissolved in anhydrous THF (13 mL) and cooled to 0 °C. To this stirred solution, TCACl (0.19 mL, 1.74 mmol) was slowly added and stirred for 1 h at 0 °C under a nitrogen atmosphere. The mixture was diluted with DCM (20 mL) and washed with brine (3x), organic phase was dried over Na_2SO_4, filtered and evaporated. The crude product was purified by silica column chromatography using 10% ethyl acetate in hexane to give **17** (861 mg, 85%) as a yellow oil. ^{1}H NMR (600 MHz, $CDCl_3$) δ 7.84 – 7.79 (m, 3H), 7.75 (s, 1H), 7.51 – 7.41 (m, 3H), 7.38 – 7.20 (m, 8H), 7.16 (d, *J* = 7.2 Hz, 1H), 6.77 (dd, *J* = 87.1, 9.2 Hz, 1H), 5.16 (d, *J* = 26.2 Hz, 2H), 4.88 – 4.78 (m, 2H), 4.77 – 4.69 (m, 1H), 4.52 – 4.40 (m, 3H), 3.92 (d, *J* = 3.1 Hz,

1H), 3.90 – 3.81 (m, 1H), 3.77 – 3.51 (m, 3H), 3.41 – 3.28 (m, 1H), 3.19 (dt, *J* = 46.0, 7.0 Hz, 2H), 1.59 – 1.42 (m, 4H), 1.32 (d, *J* = 6.6 Hz, 3H), 1.30 – 1.17 (m, 2H). ^{13}C NMR (151 MHz, $CDCl_3$) δ 156.3, 138.0, 134.9, 133.3, 133.3, 128.7, 128.7, 128.6, 128.1, 127.9, 127.9, 127.5, 127.3, 126.7, 126.5, 126.3, 125.6, 97.1, 92.9, 77.4, 77.2, 76.9, 76.6, 71.5, 68.3, 68.2, 67.3, 65.9, 50.7, 50.4, 47.2, 46.2, 29.8, 29.1, 28.0, 27.5, 23.7, 23.4, 16.5. HR-ESI-MS (m/z): calculated for $C_{39}H_{43}Cl_3N_2O_7Na$ $[M+Na]^+$: 779.2034, found: 779.2021

_N_-(Benzyl)benzyloxycarbonyl-5-aminopentyl 2-trichloroacetamido-4-azido-3-_O_-(2-napthylmethyl)- 2,4,6-tri-deoxy–α-D-glucosidepyranoside (18)

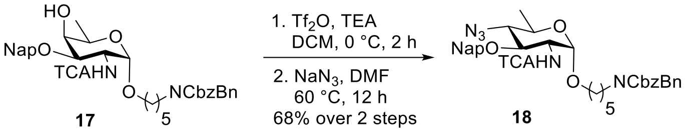

Triethylamine (0.81 mL, 13.9 mmol) was added to a stirred solution of **17** (880 mg, 1.16 mmol) in anhydrous DCM (10 mL). After being cooled to 0 °C, Tf_2O (0.29 mL, 1.74 mmol) was added dropwise. The reaction was brought to rt over 2 h and partitioned between DCM (3 x 30 mL) and water (20 mL). Washing process was repeated three times and the combined organic layer was washed with saturated aqueous $NaHCO_3$, 1N HCl, brine (20 mL), dried over Na_2SO_4, filtered and concentrated. The crude product was directly taken to the next step without purification. The crude triflate derivative was dissolved in anhydrous DMF (10 mL). To this stirred solution, NaN_3 (377 mg, 5.8 mmol) was added and gradually brought to 60 °C for 12 h. The reaction mixture was diluted with DCM (30 mL) and were washed with water (50 mL) and saturated aqueous brine (50 mL). Washing process was repeated three times and the combined organic layer was dried over Na_2SO_4, filtered and concentrated. The crude product was purified by silica gel column chromatography using 8% ethyl acetate in hexanes to afford compound **18** (613 mg, 68% over two steps). ^{1}H NMR (600 MHz, $CDCl_3$) δ 7.84 – 7.76 (m, 4H), 7.48 – 7.42 (m, 3H), 7.40 – 7.22 (m, 8H), 7.17 (d, *J* = 7.3 Hz, 1H), 7.02 – 6.72 (m, 1H), 5.18 (d, *J* = 28.7 Hz, 2H), 4.98 – 4.84 (m, 2H), 4.80 – 4.71 (m, 1H), 4.50 (d, *J* = 11.6 Hz, 2H), 4.29 – 4.19 (m, 1H), 3.78 – 3.55 (m, 3H), 3.42 – 3.14 (m, 4H), 1.59 – 1.45 (m, 4H), 1.34 (d, *J* = 6.2 Hz, 3H), 1.28 – 1.19 (m, 1H). ^{13}C NMR (151 MHz, $CDCl_3$) δ 161.8, 156.4, 137.9, 136.8, 134.9, 133.4, 133.2, 128.7, 128.6, 128.4, 128.2, 128.1, 127.9, 127.8, 127.5, 127.3, 127.1, 126.2, 126.1, 126.1, 96.9, 92.7, 78.9, 77.4, 77.2, 76.9, 75.2, 68.2, 67.4, 66.8, 55.0, 50.7, 50.5, 47.2, 46.2, 29.0, 28.0, 27.5, 23.8, 23.4, 18.5. HR-ESI-MS (m/z): calculated for $C_{39}H_{42}Cl_3N_5O_6Na$ $[M+Na]^+$: 804.2098, found: 804.2090

_N_-(Benzyl)benzyloxycarbonyl-5-aminopentyl 2-trichloroacetamido-4-azido-2,4,6-tri-deoxy-α-D-galactopyranoside (6)

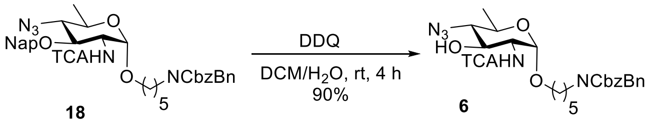

To a well stirred emulsion of **18** (613 mg, 0.78 mmol) in DCM and H_2O (7/1, *v*/*v*, 8 mL) was added DDQ (265 mg, 1.17 mmol) under the exclusion of light and stirred at rt for 6 h. The mixture was diluted with DCM (40 mL) and washed (2 x 30 mL) with 10% aqueous $Na_2S_3O_3$ to reduce the

remaining DDQ. The organic layer was dried over Na_2SO_4, filtered and concentrated *in vacuo*. The residue was purified by silica column chromatography using 10% ethyl acetate in hexanes to afford product **6** as yellowish liquid (455 mg, 90%). ^{1}H NMR (400 MHz, $CDCl_3$) δ 7.42 – 7.20 (m, 8H), 7.17 (d, *J* = 7.3 Hz, 1H), 5.26 – 5.11 (m, 2H), 4.81 (d, *J* = 14.5 Hz, 1H), 4.50 (d, *J* = 12.7 Hz, 2H), 3.97 (d, *J* = 11.2 Hz, 1H), 3.61 (dq, *J* = 15.7, 8.2 Hz, 2H), 3.45 – 3.31 (m, 1H), 3.22 – 3.10 (m, 2H), 3.05 (t, *J* = 9.6 Hz, 1H), 2.68 (s, 1H), 1.68 – 1.43 (m, 4H), 1.30 (d, *J* = 6.3 Hz, 5H). ^{13}C NMR (101 MHz, $CDCl_3$) δ 128.7, 128.6, 128.1, 128.0, 127.4, 127.3, 97.6, 77.4, 77.2, 76.9, 70.9, 69.0, 68.4, 67.3, 66.4, 63.5, 50.7, 50.4, 47.2, 46.3, 29.2, 28.0, 27.6, 23.5, 18.4. HR-ESI-MS (m/z): calculated for $C_{28}H_{34}Cl_3N_5O_6Na$ $[M+Na]^+$: 664.1472, found: 664.1473

Ethyl 4,6-*O*-benzylidene-3-*tert*-butyl dimethylsilyl-2-*O*-levulinoy-1-thio-β-D-galactopyranoside (20)

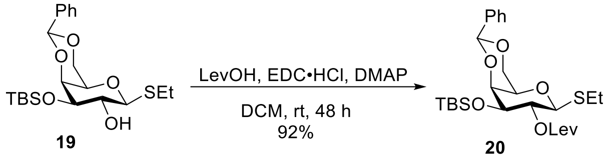

To a solution of compound **19** (3.43 g, 8.04 mmol) in DCM (35 mL) were added 4-dimethylaminopyridine (99 mg, 0.81 mmol), levulinic acid (1.65 mL, 16.1 mmol) and EDC•HCl (2.31 g, 12.1 mmol). The mixture was stirred overnight at rt, washed with saturated aqueous $NaHCO_3$ and brine, the organic layer was dried over Na_2SO_4, filtered and concentrated. The residue was purified by silica gel column chromatography using 30% ethyl acetate in hexanes to afford compound **20** (3.87 g, 92%) as a white solid. ^{1}H NMR (600 MHz, $CDCl_3$) δ 7.49 (dt, *J* = 7.6, 1.4 Hz, 2H), 7.37 – 7.31 (m, 3H), 5.48 (d, *J* = 1.7 Hz, 1H), 5.32 – 5.27 (m, 1H), 4.37 – 4.30 (m, 2H), 4.08 – 4.06 (m, 1H), 4.00 (dd, *J* = 12.4, 1.8 Hz, 1H), 3.85 (dd, *J* = 9.3, 3.6 Hz, 1H), 3.47 – 3.44 (m, 1H), 2.86 – 2.79 (m, 1H), 2.75 (td, *J* = 6.6, 1.8 Hz, 2H), 2.70 – 2.56 (m, 3H), 2.17 (d, *J* = 2.1 Hz, 3H), 1.25 (t, 3H), 0.84 (s, 9H), 0.07 (s, 3H), 0.06 (s, 3H). ^{13}C NMR (151 MHz, $CDCl_3$) δ 206.3, 171.5, 137.9, 128.9, 128.1, 128.1, 126.3, 101.1, 82.8, 77.4, 77.2, 76.9, 76.8, 73.3, 70.2, 69.8, 69.4, 38.1, 30.0, 28.3, 25.6, 25.6, 22.8, 22.8, 18.0, 14.9, -4.5, -4.6. HR-ESI-MS (m/z): calculated for $C_{26}H_{40}O_7SSiNa$ $[M+Na]^+$: 547.2162, found: 547.2168

Ethyl 3-*tert*-butyl dimethylsilyl 2-*O*-levulinoy-1-thio-β-D-galactopyranoside (21)

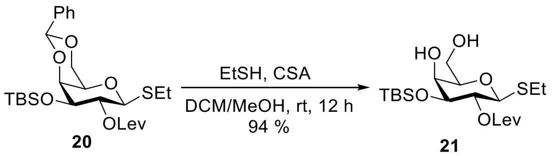

To a solution of compound **20** (4.21 g, 8.02 mmol) in a mixture of DCM/MeOH (99:1, *v*/*v*) (32 mL), were added ethanethiol (4.13 mL, 56.2 mmol) and CSA (373 mg, 1.6 mmol). After stirring at rt for 6 h, the mixture was quenched with Et_3N and concentrated *in vacuo*. The residue was purified by silica gel column chromatography using 40% ethyl acetate in hexanes to give **21** (3.3 g, 94%). ^{1}H NMR (600 MHz, $CDCl_3$) δ 5.14 (t, *J* = 9.5 Hz, 1H), 4.33 (d, *J* = 10.0 Hz, 1H), 4.01 –

3.95 (m, 1H), 3.86 (dd, J = 3.5, 1.2 Hz, 1H), 3.82 – 3.75 (m, 2H), 3.62 – 3.56 (m, 1H), 2.81 – 2.70 (m, 3H), 2.70 – 2.59 (m, 3H), 2.19 (s, 3H), 1.25 (t, J = 7.5 Hz, 3H), 0.88 (s, 9H), 0.12 (s, 3H), 0.10 (s, 3H). ^{13}C NMR (151 MHz, $CDCl_3$) δ 206.3, 171.8, 83.5, 78.5, 77.4, 77.2, 76.9, 73.9, 70.5, 70.4, 62.7, 38.1, 30.1, 28.3, 25.7, 23.8, 18.0, 15.0, -4.6, -4.6. HR-ESI-MS (m/z): calculated for $C_{19}H_{36}O_7SSiNa$ $[M+Na]^+$: 459.1849, found: 459.1857

Ethyl 4-*O*-acetyl-6-*O*-benzyl-3-*tert*-butyl dimethylsilyl-2-*O*-levulinoy-1-thio-β-D-glucopyranoside uronate (8)

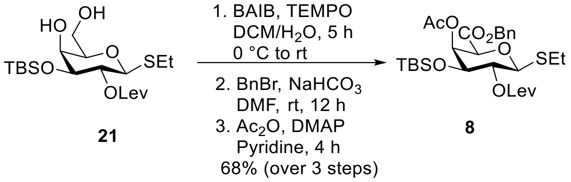

Diol **21** (167 mg, 0.382 mmol) was dissolved in a mixture of DCM/H_2O (5:1, *v*/*v*, 6 mL) and cooled to 0 °C before TEMPO (6 mg, 0.038 mmol) and BAIB (197 mg, 0.611 mmol) were added and stirred at 0 °C for 20 min and slowly warmed to rt and stirred for 3 h. Reaction mixture was then diluted with DCM (5 mL) and water (5 mL) and the aqueous layer was extracted four times with DCM (5 mL each). Combined organics were dried over Na_2SO_4, filtered, and concentrated. The crude product was dissolved in DMF (1 mL), $NaHCO_3$ (96 mg, 1.15 mmol) and BnBr (91 µL, 0.764 mmol) were added at 0 °C. Then the reaction was stirred overnight at rt. After complete consumption of starting material, the mixture was diluted with DCM (20 mL) and washed with brine. The separated organic layer was dried over Na_2SO_4 and concentrated *in vacuo*. Crude hydroxyl compound dissolved in pyridine (5 mL), was added acetic anhydride (0.24 mL, 2.56 mmol) at 0 °C. After being stirred at rt for 6 h, the mixture was concentrated *in vacuo*, diluted with DCM (10 mL) and organic layer was washed with saturated $NaHCO_3$, brine (10 mL) and dried over Na_2SO_4, filtered and concentrated *in vacuo*. The residue was purified by silica gel column chromatography using 20% ethyl acetate in hexanes to give **8** (165 mg, 68% over 3 steps). ^{1}H NMR (400 MHz, $CDCl_3$) δ 7.39 – 7.26 (m, 4H), 5.57 (dd, J = 3.6, 1.3 Hz, 1H), 5.19 – 5.05 (m, 3H), 4.38 (d, J = 10.0 Hz, 1H), 4.22 (d, J = 1.3 Hz, 1H), 3.84 (dd, J = 9.2, 3.6 Hz, 1H), 2.78 – 2.71 (m, 0H), 2.71 – 2.62 (m, 1H), 2.62 – 2.55 (m, 2H), 2.16 (s, 3H), 1.90 (d, J = 0.9 Hz, 3H), 1.25 (t, J = 7.4 Hz, 3H), 0.78 (s, 8H), 0.07 (s, 3H), 0.05 (s, 3H). ^{13}C NMR (101 MHz, $CDCl_3$) δ 206.2, 171.5, 169.7, 166.3, 135.0, 129.1, 128.6, 128.6, 83.7, 77.5, 77.2, 76.8, 76.0, 71.8, 70.5, 70.1, 67.5, 37.9, 30.0, 28.1, 25.4, 24.2, 20.5, 17.7, 14.9, -4.7, -5.0. HR-ESI-MS (m/z): calculated for $C_{28}H_{42}O_9SSiNa$ $[M+Na]^+$: 605.2216, found: 605.2230

Ethyl 2-*O*-levulinoyl-3,4,6-*O*-tri-benzyl-1-thio-β-D-glucopyranoside (9)

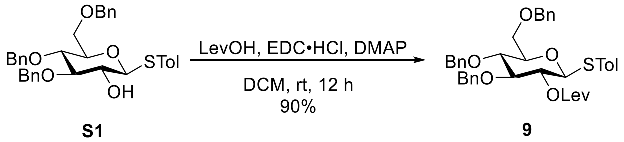

To a solution of compound[2] **S1** (1.54 g, 2.77 mmol) in dichloromethane (15 mL) were added 4-dimethylaminopyridine (84 mg, 0.69 mmol), levulinic acid (0.425 mL, 4.14 mmol) and EDC•HCl

(847 mg, 4.42 mmol). The mixture was stirred overnight at rt, washed with saturated $NaHCO_3$ and brine, the organic layer was dried over Na_2SO_4, filtered and concentrated *in vacuo*. The residue was purified by flash column chromatography using 15% ethyl acetate in hexanes to afford compound **9** (1.62 g, 90%) as a white solid. ^{1}H NMR (400 MHz, $CDCl_3$) δ 7.41 – 7.35 (m, 2H), 7.32 – 7.15 (m, 13H), 7.17 – 7.09 (m, 2H), 6.96 (d, *J* = 7.9 Hz, 2H), 4.95 (dd, *J* = 10.0, 8.7 Hz, 1H), 4.76 – 4.60 (m, 3H), 4.58 – 4.39 (m, 4H), 3.75 – 3.54 (m, 4H), 3.46 (dq, *J* = 7.5, 2.4 Hz, 1H), 2.71 – 2.56 (m, 2H), 2.56 – 2.35 (m, 2H), 2.21 (s, 3H), 2.07 (s, 3H). ^{13}C NMR (101 MHz, $CDCl_3$) δ 206.1, 171.3, 138.1, 138.0, 137.9, 137.8, 133.1, 129.5, 128.5, 128.3, 128.3, 128.2, 127.9, 127.7, 127.6, 127.5, 127.4, 85.9, 84.2, 79.2, 77.5, 77.5, 77.2, 76.8, 75.1, 74.9, 73.3, 72.0, 68.7, 37.7, 29.7, 28.0, 21.0. HR-ESI-MS (m/z): calculated for $C_{39}H_{42}O_7SNa$ $[M+Na]^+$: 677.2549, found: 677.2568

_N_-(Benzyl)benzyloxycarbonyl-5-aminopentyl 2-azido-2-deoxy-3-_O_-(2-naphthylmethyl)-4,6-_O_-silylidene-α-D-galactopyranosyl-(1→3)-2-trichloroacetamido-4-azido-2,4,6-tri-deoxy-a-glucopyranosyl (22)

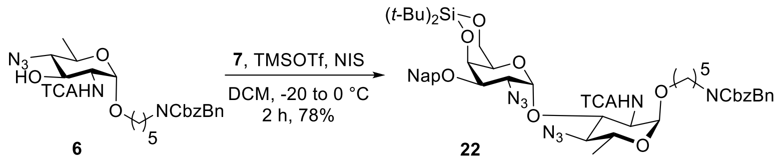

Acceptor **6** (100 mg, 0.156 mmol) and selenoglycoside **7** (127 mg 0.203 mmol) were mixed, co-evaporated with toluene (3 x 10 mL) and dried under high vacuum for 2 h. The mixture was freshly activated 4Å molecular sieves, dissolved in anhydrous DCM (12 mL) under a nitrogen atmosphere and stirred for 30 min at rt. The mixture was cooled to 0 °C, to this stirred suspension, NIS (52.6 mg, 0.234 mmol) and TMSOTf (5.6 µL, 31 µmol) were slowly added. After stirring at rt for 2 h, diluted with DCM (10 mL), quenched with Et_3N, warmed to rt and 4Å molecular sieves were filtered. The filtrate was washed with 10% aqueous $Na_2S_2O_3$, saturated aqueous $NaHCO_3$ and brine. The combined organic layer was dried over Na_2SO_4, filtered and evaporated *in vacuo*. The crude product was purified by silica column chromatography using10% ethyl acetate in hexanes to afford the desired protected disaccharide **22** (135 mg, 78%). ^{1}H NMR (600 MHz, $CDCl_3$) δ 7.88 – 7.80 (m, 4H), 7.57 (dd, *J* = 8.4, 1.7 Hz, 1H), 7.50 – 7.44 (m, 2H), 7.39 – 7.14 (m, 9H), 6.87 (dd, *J* = 59.6, 9.4 Hz, 1H), 5.40 (d, *J* = 0.9 Hz, 1H), 5.18 (d, *J* = 18.2 Hz, 2H), 4.91 – 4.82 (m, 2H), 4.72 – 4.65 (m, 1H), 4.54 – 4.46 (m, 3H), 4.18 – 4.04 (m, 3H), 4.00 (dd, *J* = 10.6, 3.7 Hz, 1H), 3.91 (dd, *J* = 10.6, 2.7 Hz, 1H), 3.78 – 3.52 (m, 4H), 3.38 – 3.15 (m, 4H), 1.60 – 1.44 (m, 4H), 1.38 (d, *J* = 6.2 Hz, 3H), 1.33 – 1.20 (m, 2H), 1.04 (s, 9H), 1.02 (s, 9H). ^{13}C NMR (151 MHz, $CDCl_3$) δ 162.0, 156.3, 137.9, 136.8, 135.5, 133.4, 133.2, 128.7, 128.6, 128.4, 128.2, 128.0, 127.9, 127.8, 127.5, 127.3, 126.8, 126.2, 126.1, 126.0, 99.4, 96.5, 92.4, 77.4, 77.2, 76.9, 76.8, 76.4, 71.2, 70.1, 69.7, 68.3, 68.2, 67.5, 67.4, 67.2, 58.7, 54.5, 50.7, 50.5, 47.1, 46.2, 29.1, 28.0, 27.7, 27.5, 27.5, 23.6, 23.5, 20.8, 18.3. HR-ESI-MS (m/z): calculated for $C_{53}H_{67}C_{l3}N_8O_{10}SiNa$ $[M+Na]^+$: 1131.3713, found: 1131.3721

N-(Benzyl)benzyloxycarbonyl-5-aminopentyl 2-azido-2-deoxy-3-O-(2-naphthylmethyl)-α-D-galactopyranosyl-(1→3)-2-trichloroacetamido-4-azido-2,4,6-tri-deoxy-α-D-glucopyranosyl (23)

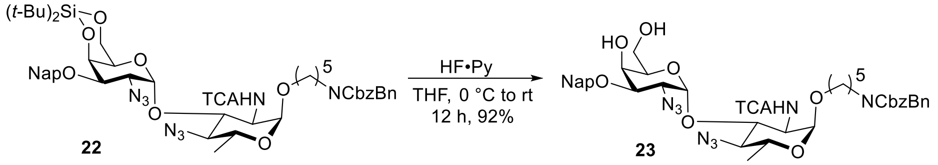

To a solution of **22** (315 mg, 0.287 mmol) in THF (2.8 mL, 0.1 M) was added HF•Py (70% HF, 0.74 mL, 5.75 mmol). After TLC analysis (40% ethyl acetate in hexanes) indicated complete conversion of the starting material (~8 hours), the reaction was quenched with Et_3N (4 mL). The mixture was concentrated, dissolved in EtOAc and subsequently washed with saturated aqueous $NaHCO_3$ and brine. The aqueous layers were then extracted with EtOAc, combined organic layers were dried over Na_2SO_4, filtered and concentrated *in vacuo*. The residue was purified by silica gel column chromatography using 35% ethyl acetate in hexanes to furnish the title compound **23** (257 mg, 92%). ^{1}H NMR (600 MHz, $CDCl_3$) δ 7.87 – 7.82 (m, 4H), 7.54 – 7.48 (m, 3H), 7.40 – 7.21 (m, 10H), 7.18 (d, *J* = 7.3 Hz, 1H), 5.46 (dd, *J* = 33.6, 3.6 Hz, 1H), 5.19 (d, *J* = 22.6 Hz, 2H), 4.90 – 4.78 (m, 2H), 4.74 – 4.65 (m, 1H), 4.48 (dd, *J* = 27.9, 16.1 Hz, 2H), 4.22 – 4.14 (m, 1H), 4.01 (t, *J* = 5.7 Hz, 1H), 3.97 – 3.79 (m, 5H), 3.77 – 3.72 (m, 1H), 3.70 – 3.52 (m, 2H), 3.31 – 3.17 (m, 4H), 3.00 (bs, 1H), 1.59 – 1.45 (m, 5H), 1.37 (d, *J* = 6.2 Hz, 3H), 1.34 – 1.23 (m, 2H). ^{13}C NMR (151 MHz, $CDCl_3$) δ 162.2, 162.1, 156.8, 156.3, 137.7, 136.8, 136.6, 134.6, 133.2, 133.2, 128.6, 128.5, 128.5, 128.4, 128.1, 128.0, 127.9, 127.9, 127.8, 127.7, 127.5, 127.4, 127.2, 127.1, 126.3, 126.2, 125.8, 98.3, 98.0, 96.6, 96.4, 92.4, 77.4, 77.2, 76.9, 76.5, 76.4, 75.9, 75.3, 72.4, 70.7, 69.2, 69.0, 68.1, 68.0, 67.9, 67.8, 67.3, 66.8, 63.2, 63.1, 59.4, 54.6, 54.5, 50.6, 50.2, 47.0, 46.0, 29.7, 28.9, 27.8, 27.4, 23.5, 23.3, 18.2. HR-ESI-MS (m/z): calculated for $C_{45}H_{51}Cl_3N_8NaO_{10}Na$ $[M+Na]^+$: 991.2691, found: 991.2698

N-(Benzyl)benzyloxycarbonyl-5-aminopentyl 2-azido-2-deoxy-3-O-(2-naphthylmethyl)-4,6-O-di-benzyl-α-D-galactopyranosyl-(1→3)-2-trichloroacetamido-4-azido-2,4,6-tri-deoxy-α-D-glucopyranosyl (24)

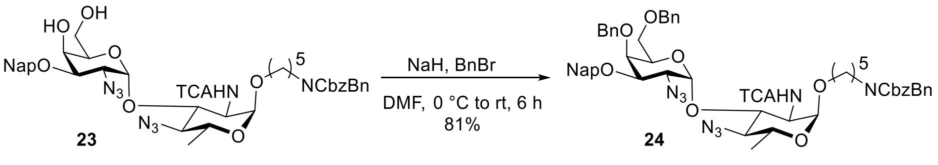

To a cooled solution of **23** (380 mg, 0.392 mmol) in DMF (2 mL) at 0 °C were added benzyl bromide (0.103 mL, 0.862 mmol) and NaH (60% disp.) (21.6 mg, 0.902 mmol). After stirring for 6 h, the mixture was neutralized with H_2O and added EtOAc. The organic layer was washed with H_2O for five times and brine, dried over Na_2SO_4 and concentrated *in vacuo*. The residue was purified by silica column chromatography using 12% ethyl acetate in hexanes to yield the desired compound **24** (364 mg, 81%). ^{1}H NMR (400 MHz, $CDCl_3$) δ 7.89 – 7.83 (m, 4H), 7.55 – 7.48 (m, 3H), 7.37 – 7.21 (m, 21H), 7.07 – 6.85 (m, 1H), 5.50 (d, *J* = 3.1 Hz, 1H), 5.20 (d, *J* = 11.0 Hz,

2H), 4.92 – 4.85 (m, 3H), 4.80 – 4.69 (m, 1H), 4.54 – 4.47 (m, 4H), 4.41 (d, *J* = 11.6 Hz, 1H), 4.20 – 4.11 (m, 1H), 4.10 – 3.99 (m, 4H), 3.89 – 3.59 (m, 3H), 3.56 (d, *J* = 6.8 Hz, 2H), 3.37 – 3.13 (m, 4H), 1.58 – 1.44 (m, 4H), 1.39 (d, *J* = 6.1 Hz, 3H), 1.28 – 1.18 (m, 2H). ^{13}C NMR (101 MHz, $CDCl_3$) δ 162.1, 156.8, 156.3, 138.4, 137.9, 137.7, 136.9, 136.7, 135.2, 133.4, 133.1, 133.1, 128.7, 128.6, 128.5, 128.4, 128.4, 128.4, 128.3, 128.2, 128.1, 128.1, 128.0, 127.9, 127.9, 127.8, 127.8, 127.4, 127.3, 126.6, 126.2, 126.1, 126.0, 125.8, 98.2, 96.4, 92.4, 77.5, 77.5, 77.4, 77.2, 76.8, 75.0, 74.9, 73.7, 73.6, 73.4, 72.5, 70.3, 69.5, 68.3, 68.2, 68.1, 67.3, 67.0, 60.0, 54.4, 50.6, 50.3, 47.1, 46.1, 29.8, 29.0, 27.9, 27.4, 23.5, 23.3, 18.3. HR-ESI-MS (m/z): calculated for $C_{59}H_{63}Cl_3N_8O_{10}Na$ $[M+Na]^+$: 1171.3630, found: 1171.3637

_N_-(Benzyl)benzyloxycarbonyl-5-aminopentyl 2-azido-2-deoxy-4,6-_O_-di-benzyl-α-D-galactopyranosyl-(1→3)-2-trichloroacetamido-4-azido-2,4,6-tri-deoxy-α-D-glucopyranosyl (25)

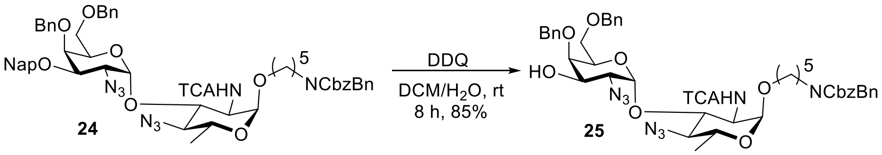

To a well stirred emulsion of **24** (685 mg, 0.60 mmol) in DCM and H_2O (7/1, *v*/*v*, 8 mL) was added DDQ (202 mg, 0.89 mmol) under the exclusion of light and stirred at rt for 8 h. The mixture was diluted with DCM (10 mL) and washed (2 x 15 mL) with 10% aqueous $Na_2S_3O_3$ to reduce the remaining DDQ. The organic layer was dried over Na_2SO_4, filtered and concentrated *in vacuo.* The residue was purified by silica column chromatography using 15% ethyl acetate in hexanes to afford product **25** as yellowish liquid (511 mg, 85%). ^{1}H NMR (700 MHz, $CDCl_3$) δ 7.37 – 7.21 (m, 18H), 7.19 – 7.13 (m, 1H), 7.05 – 6.88 (m, 1H), 5.44 (dd, *J* = 9.3, 3.9 Hz, 1H), 5.20 – 5.12 (m, 2H), 4.72 (d, *J* = 20.3 Hz, 1H), 4.67 – 4.59 (m, 2H)j, 4.52 – 4.44 (m, 3H), 4.41 (d, *J* = 11.7 Hz, 1H), 4.12 – 4.06 (m, 2H), 4.05 – 4.01 (m, 1H), 3.89 (s, 1H), 3.82 – 3.63 (m, 2H), 3.63 – 3.48 (m, 4H), 3.36 – 3.11 (m, 4H), 2.28 (bs, 1H), 1.55 – 1.40 (m, 4H), 1.36 – 1.32 (m, 3H), 1.29 – 1.15 (m, 2H). ^{13}C NMR (176 MHz, $CDCl_3$) δ 162.1, 156.8, 156.3, 138.1, 137.9, 137.7, 137.6, 136.9, 136.8, 128.7, 128.7, 128.6, 128.5, 128.4, 128.2, 128.1, 128.1, 128.0, 128.0, 127.9, 127.9, 127.5, 127.3, 98.1, 96.4, 92.5, 77.4, 77.3, 77.2, 77.2, 77.0, 77.0, 76.6, 76.6, 75.3, 75.1, 75.0, 73.7, 70.2, 69.4, 68.7, 68.6, 68.3, 68.2, 68.1, 67.3, 67.0, 61.4, 54.4, 50.7, 50.4, 47.1, 46.2, 29.0, 27.9, 27.5, 23.5, 23.3, 18.2. HR-ESI-MS (m/z): calculated for $C_{48}H_{55}Cl_3N_8O_{10}Na$ $[M+Na]^+$: 1031.3004, found: 1031.3011

N-(Benzyl)benzyloxycarbonyl-5-aminopentyl 2-O-levulinoyl-4-O-acetyl-6-O-benzyl-β-D-galactopyranosyl uronate-(1→3)-2-azido-2-deoxy-4,6-O-di-benzyl-α-D-galactopyranosyl-(1→3)-2-trichloroacetamido-4-azido-2,4,6-tri-deoxy-α-D-glucopyranosyl (26)

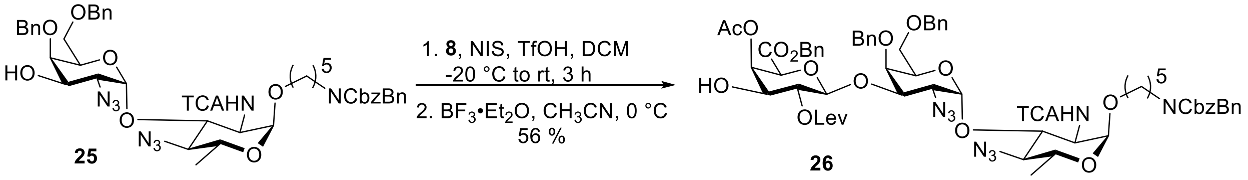

Acceptor **25** (76 mg, 0.075 mmol) and thioglycoside **8** (127 mg 0.135 mmol) were mixed, co-evaporated with toluene (3 x 10 mL) and dried under high vacuum for 2 h. The added freshly activated 4Å molecular sieves were dissolved in anhydrous DCM (5 mL) under a nitrogen atmosphere and stirred for 30 min at rt. The mixture was cooled to -20 °C, to this stirred suspension, NIS (30.3 mg, 0.135 mmol) and TfOH (2.6 µL, 0.03 mmol) were slowly added. After stirring at rt for 2 h, diluted with DCM (10 mL), quenched with Et_3N, warmed to rt and 4Å molecular sieves were filtered. The filtrate was washed with 10% aqueous $Na_2S_2O_3$, saturated aqueous $NaHCO_3$ and brine. The combined organic layer was dried over Na_2SO_4, filtered and evaporated *in vacuo*. The residue was passed through short silica column using ethyl acetate and evaporated *in vacuo.* The crude was dissolved in the anhydrous ACN (3 mL), cooled to 0 °C and BF_3•Et_2O (9.2 µmol, 0.075 mmol) was added. After stirring for 5 min, saturated aqueous $NaHCO_3$ was added and extracted the organic layer with DCM. The combined layers were dried over Na_2SO_4, filtered and evaporated *in vacuo*. The crude was purified by silica gel column chromatography using 20% ethyl acetate in hexanes to afford compound **26** (60 mg, 56% over two steps). ^{1}H NMR (400 MHz, $CDCl_3$) δ 7.31 – 7.14 (m, 23H), 7.08 (d, *J* = 7.2 Hz, 1H), 7.02 – 6.87 (m, 1H), 5.61 (dd, *J* = 3.6, 1.3 Hz, 1H), 5.45 (d, *J* = 3.6 Hz, 1H), 5.16 – 5.04 (m, 4H), 4.95 (dd, *J* = 16.1, 11.7 Hz, 2H), 4.73 (d, *J* = 7.9 Hz, 1H), 4.61 (d, *J* = 16.1 Hz, 1H), 4.52 (d, *J* = 11.6 Hz, 1H), 4.43 – 4.21 (m, 5H), 4.19 – 4.00 (m, 2H), 3.98 – 3.86 (m, 3H), 3.87 – 3.76 (m, 2H), 3.61 (d, *J* = 6.2 Hz, 1H), 3.54 – 3.37 (m, 2H), 3.25 (s, 1H), 3.18 – 3.03 (m, 4H), 2.88 – 2.77 (m, 1H), 2.73 – 2.61 (m, 1H), 2.60 – 2.47 (m, 2H), 2.10 (s, 3H), 1.80 (s, 3H), 1.45 – 1.32 (m, 4H), 1.28 (d, *J* = 6.2 Hz, 3H), 1.17 – 1.04 (m, 2H). ^{13}C NMR (101 MHz, $CDCl_3$) δ 208.0, 173.0, 170.3, 166.0, 138.9, 138.1, 137.9, 135.0, 129.1, 128.8, 128.7, 128.6, 128.4, 128.4, 128.1, 128.0, 127.9, 127.8, 127.7, 127.5, 127.3, 102.0, 97.7, 96.5, 92.3, 78.8, 77.5, 77.4, 77.2, 76.8, 76.4, 75.1, 73.6, 72.7, 72.5, 71.1, 70.4, 69.5, 67.6, 67.3, 66.9, 60.0, 54.4, 50.7, 50.4, 47.1, 46.2, 38.7, 29.9, 29.9, 29.0, 28.4, 20.6, 18.3. HR-ESI-MS (m/z): calculated for $C_{68}H_{77}Cl_3N_8O_{19}Na$ $[M+Na]^+$: 1437.4268, found: 1437.4261

_N_-(Benzyl)benzyloxycarbonyl-5-aminopentyl 2-levulinoyl-3-_tert_-butyl dimethylsilyl-4,6-_O_-benzylidene-β-D-galactopyranosyl-(1→3)-2-azido-2-deoxy-4,6-_O_-di-benzyl-α-D-galactopyranosyl-(1→3)-2-trichloroacetamido-4-azido-2,4,6-tri-deoxy-α-D-glucopyranosyl (29)

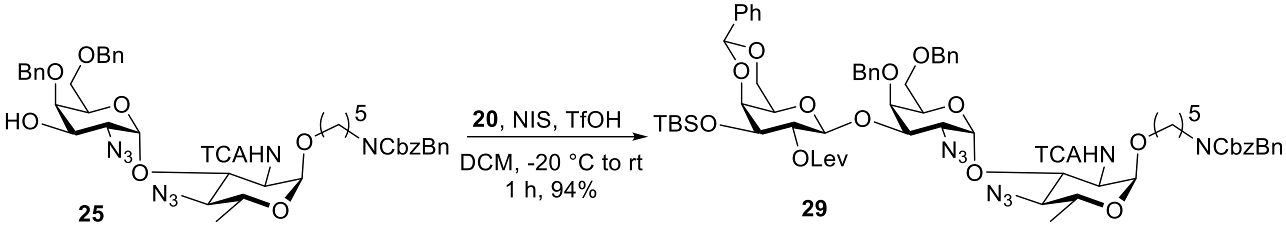

Acceptor **25** (200 mg, 0.198 mmol) and thioglycoside **20** (145 mg, 0.277 mmol) were mixed, co-evaporated with toluene (3 x 10 mL) and dried under vacuum for 2 h. The mixture was dissolved in anhydrous DCM (6 mL), freshly activated 4Å molecular sieves were added under a nitrogen atmosphere and stirred for 30 min at rt. The mixture was cooled to -20 °C, to this stirred suspension, NIS (68 mg, 0.297 mmol) and TfOH (26 µL, 0.059 mmol) were slowly added. The mixture was brought to 0 °C over 4 h, diluted with DCM (5 mL), quenched with Et_3N, warmed to rt and 4Å molecular sieves were filtered. The filtrate was washed with saturated aqueous $Na_2S_2O_3$, saturated aqueous $NaHCO_3$ and brine. The combined organic layer was dried over Na_2SO_4, filtered and evaporated *in vacuo*. The crude product was purified by silica column chromatography using 20% ethyl acetate in hexanes to afford the desired protected trisaccharide **29** (275 mg, 94%). ^1H NMR (600 MHz, $CDCl_3$) δ 7.53 – 7.48 (m, 2H), 7.46 – 7.18 (m, 24H), 7.07 (dd, *J* = 49.5, 9.3 Hz, 1H), 5.64 (d, *J* = 3.7 Hz, 1H), 5.59 (s, 1H), 5.41 (dd, *J* = 9.8, 7.9 Hz, 1H), 5.24 (d, *J* = 16.7 Hz, 2H), 5.11 (d, *J* = 11.7 Hz, 1H), 4.83 – 4.74 (m, 2H), 4.65 (d, *J* = 11.7 Hz, 1H), 4.54 (d, *J* = 15.3 Hz, 2H), 4.49 – 4.43 (m, 1H), 4.41 – 4.31 (m, 2H), 4.24 – 4.13 (m, 4H), 4.08 (td, *J* = 9.6, 8.5, 4.3 Hz, 2H), 4.00 – 3.88 (m, 3H), 3.82 – 3.61 (m, 2H), 3.61 – 3.53 (m, 2H), 3.43 – 3.36 (m, 2H), 3.35 – 3.25 (m, 2H), 3.22 (d, *J* = 7.7 Hz, 1H), 2.90 – 2.83 (m, 1H), 2.80 (dd, *J* = 7.7, 5.8 Hz, 1H), 2.78 – 2.73 (m, 2H), 2.22 (s, 3H), 1.58 – 1.48 (m, 4H), 1.44 (d, *J* = 6.2 Hz, 3H), 1.35 – 1.30 (m, 2H), 0.93 (s, 9H), 0.16 (s, 3H), 0.16 (s, 3H). ^{13}C NMR (151 MHz, $CDCl_3$) δ 206.7, 171.5, 162.2, 156.8, 156.3, 138.7, 138.0, 137.9, 137.8, 128.9, 128.8, 128.8, 128.7, 128.6, 128.5, 128.4, 128.4, 128.2, 128.1, 128.1, 128.0, 127.9, 127.8, 127.7, 127.4, 127.3, 126.3, 102.6, 101.2, 98.0, 96.4, 92.4, 77.4, 77.3, 77.2, 77.0, 76.9, 76.4, 76.1, 74.9, 73.5, 73.3, 73.2, 72.0, 71.9, 70.4, 69.6, 69.2, 68.2, 68.1, 67.3, 67.0, 66.6, 59.8, 54.5, 50.7, 50.3, 47.1, 46.1, 38.1, 30.0, 29.8, 29.0, 28.1, 27.9, 27.4, 26.0, 25.6, 23.4, 23.3, 18.3, 18.1, 1.1, -4.4, -4.5. HR-ESI-MS (m/z): calculated for $C_{72}H_{89}Cl_3N_8O_{17}SiNa$ $[M+Na]^+$: 1493.5078, found: 1493.5072

N-(Benzyl)benzyloxycarbonyl-5-aminopentyl 2-levulinoyl-4,6-O-benzylidene-β-D-galactopyranosyl-(1→3)-2-azido-2-deoxy-4,6-O-di-benzyl-α-D-galactopyranosyl-(1→3)-2-trichloroacetamido-4-azido-2,4,6-tri-deoxy-α-D-glucopyranosyl (30)

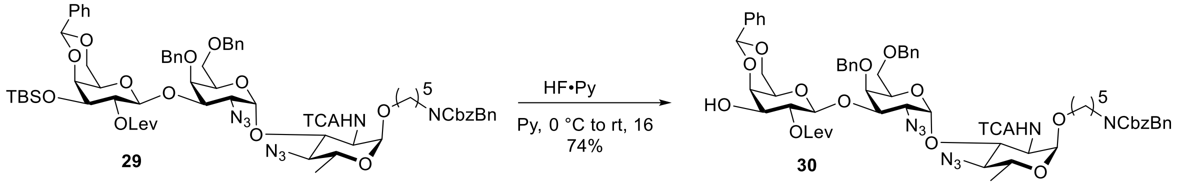

Protected trisaccharide **29** (290 mg g, 0.197 mmol) was dissolved in anhydrous pyridine (5 mL) at 0 °C and to this, HF•Py (0.76 mL, 5.91 mmol) was added dropwise and the reaction was stirred overnight at rt. The reaction mixture was diluted with water and extracted three times with DCM (3 x10 mL). Organic layers were combined and then washed twice with cold dil. HCl (20 mL each time), saturated aqueous $NaHCO_3$ (20 mL), brine (10 mL), dried over Na_2SO_4, filtered and concentrated *in vacuo*. The residue was purified by silica gel column chromatography using 20% ethyl acetate in hexanes to yield **30** (204 mg, 74%) as a yellowish liquid. ^{1}H NMR (400 MHz, $CDCl_3$) δ 7.33 – 7.05 (m, 25H), 6.89 (dd, *J* = 31.0, 9.6 Hz, 1H), 5.48 (s, 1H), 5.45 (d, *J* = 3.7 Hz, 1H), 5.17 (dd, *J* = 10.0, 7.8 Hz, 1H), 5.09 (d, *J* = 8.7 Hz, 2H), 5.02 (d, *J* = 11.5 Hz, 1H), 4.74 (d, *J* = 7.9 Hz, 1H), 4.66 – 4.58 (m, 1H), 4.53 (d, *J* = 11.5 Hz, 1H), 4.39 (d, *J* = 7.5 Hz, 2H), 4.35 – 4.21 (m, 2H), 4.20 – 4.16 (m, 2H), 4.10 – 3.99 (m, 3H), 3.95 (td, *J* = 10.2, 9.7, 4.5 Hz, 2H), 3.82 (dd, *J* = 10.8, 3.6 Hz, 1H), 3.75 (dt, *J* = 10.0, 7.8 Hz, 2H), 3.66 – 3.55 (m, 1H), 3.53 – 3.37 (m, 3H), 3.29 (dd, *J* = 9.2, 6.5 Hz, 2H), 3.14 (t, *J* = 9.6 Hz, 3H), 2.81 – 2.74 (m, 1H), 2.72 – 2.66 (m, 1H), 2.63 – 2.54 (m, 2H), 2.08 (s, 3H), 1.42 (t, *J* = 6.4 Hz, 1H), 1.29 (d, *J* = 6.2 Hz, 3H), 1.17 – 1.04 (m, 3H). ^{13}C NMR (151 MHz, $CDCl_3$) δ 207.1, 172.8, 162.3, 156.9, 156.4, 138.8, 138.1, 138.0, 137.6, 137.0, 129.4, 128.8, 128.8, 128.7, 128.5, 128.4, 128.4, 128.2, 128.2, 128.1, 128.0, 127.9, 127.8, 127.7, 127.5, 127.4, 126.6, 102.3, 101.8, 98.1, 96.5, 92.5, 78.2, 77.5, 77.3, 77.1, 77.0, 76.3, 75.5, 75.2, 74.0, 73.6, 72.8, 71.6, 70.4, 69.7, 69.2, 69.0, 68.3, 67.4, 67.1, 66.6, 60.1, 54.6, 50.4, 47.2, 38.5, 30.0, 29.9, 29.1, 28.4, 28.0, 27.5, 23.5, 23.4, 18.4. HR-ESI-MS (m/z): calculated for $C_{66}H_{75}Cl_3N_8O_{17}Na$ $[M+Na]^+$: 1379.4213; found 1379.4221

N-(Benzyl)benzyloxycarbonyl-5-aminopentyl 2-azido-2-deoxy-3-O-benzyl-4,6-O-silylidene-α-D-galactopyranosyl-(1→3)-2-levulinoyl-4,6-O-benzylidene-β-D-galactopyranosyl-(1→3)-2-azido-2-deoxy-4,6-O-di-benzyl-α-D-galactopyranosyl-(1→3)-2-trichloroacetamido-4-azido-2,4,6-tri-deoxy-α-D-glucopyranosyl (31)

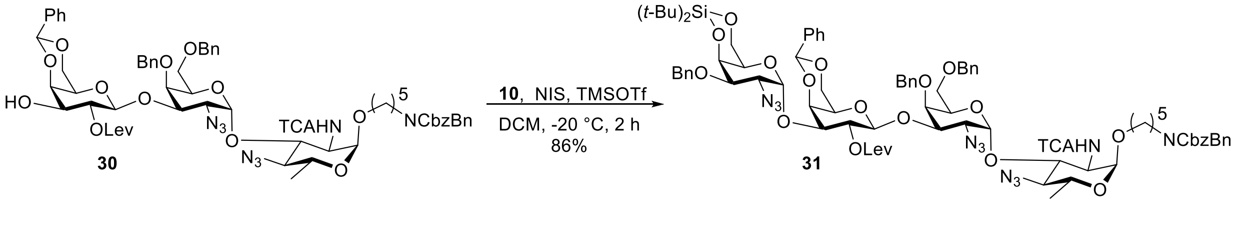

Selenoglycoside donor **10** (119 mg, 0.207 mmol) and trisaccharide acceptor **30** (204 mg, 0.138 mmol) were dried azeotropically with toluene in rotary evaporator. DCM (5 mL) was then added followed by activated 4Å molecular sieves and the solution stirred at rt for 30 min before cooling

to -20 °C. NIS (49.7 mg, 0.221 mmol) and TMSOTf (7.5 µL, 0.041 mmol) were added and the reaction mixture stirred at -20 °C for 2 h. The reaction mixture was quenched with Et_3N, warmed to rt and 4Å molecular sieves were filtered. The filtrate was washed with saturated aqueous $Na_2S_2O_3$, saturated aqueous $NaHCO_3$ and brine. The combined organic layer was dried over Na_2SO_4, filtered and evaporated *in vacuo*. The crude product was purified by silica gel column chromatography using 20% ethyl acetate in hexanes to afford the desired protected tetrasaccharide **31** (210 mg, 86%). ^{1}H NMR (600 MHz, $CDCl_3$) δ 7.42 – 7.38 (m, 2H), 7.32 (d, *J* = 7.7 Hz, 2H), 7.30 – 7.20 (m, 8H), 7.19 – 7.15 (m, 8H), 7.15 – 7.05 (m, 10H), 6.96 – 6.83 (m, 1H), 5.53 (s, 1H), 5.48 (d, *J* = 3.8 Hz, 1H), 5.38 (dd, *J* = 10.0, 7.9 Hz, 1H), 5.12 – 5.06 (m, 3H), 4.94 (d, *J* = 11.7 Hz, 1H), 4.86 (d, *J* = 2.8 Hz, 1H), 4.72 – 4.66 (m, 2H), 4.51 (dd, *J* = 18.5, 11.4 Hz, 2H), 4.39 (d, *J* = 16.0 Hz, 3H), 4.34 (d, *J* = 3.7 Hz, 1H), 4.32 – 4.27 (m, 2H), 4.21 (td, *J* = 12.6, 3.9 Hz, 2H), 4.14 (d, *J* = 12.8 Hz, 1H), 4.10 – 4.01 (m, 3H), 3.97 – 3.87 (m, 6H), 3.83 – 3.77 (m, 1H), 3.74 – 3.67 (m, 2H), 3.63 (q, *J* = 7.6, 6.8 Hz, 2H), 3.50 – 3.46 (m, 1H), 3.41 (q, *J* = 9.9, 7.7 Hz, 2H), 3.29 – 3.21 (m, 2H), 3.13 (dd, *J* = 12.1, 7.1 Hz, 3H), 3.10 – 3.04 (m, 1H), 2.90 – 2.82 (m, 1H), 2.56 – 2.47 (m, 1H), 2.46 – 2.37 (m, 2H), 2.04 (s, 3H), 1.44 – 1.32 (m, 4H), 1.29 (d, *J* = 6.3 Hz, 3H), 1.20 – 1.13 (m, 2H), 1.00 (s, 9H), 0.97 (d, *J* = 1.6 Hz, 9H). ^{13}C NMR (151 MHz, $CDCl_3$) δ 206.8, 171.2, 162.2, 156.7, 156.3, 138.6, 138.6, 138.3, 138.0, 137.9, 137.6, 136.9, 136.8, 133.2, 129.4, 129.3, 128.9, 128.7, 128.7, 128.6, 128.5, 128.4, 128.4, 128.3, 128.2, 128.1, 128.1, 128.1, 128.0, 128.0, 127.9, 127.8, 127.7, 127.7, 127.4, 127.4, 127.3, 126.4, 126.3, 102.6, 101.2, 98.1, 96.4, 95.6, 92.4, 77.4, 77.2, 76.9, 76.0, 75.0, 75.0, 74.9, 74.2, 73.5, 71.7, 70.4, 70.4, 70.3, 69.7, 69.6, 69.1, 69.0, 68.4, 68.2, 68.1, 67.3, 67.2, 66.9, 66.5, 59.8, 57.8, 54.5, 50.7, 50.3, 47.1, 37.5, 30.1, 29.0, 28.1, 28.1, 27.8, 27.8, 27.8, 27.6, 27.4, 27.3, 23.5, 23.4, 23.3, 20.8, 18.2. HR-ESI-MS (m/z): calculated for $C_{87}H_{106}Cl_3N_{11}O_{21}SiNa$ $[M+Na]^+$: 1796.6297, found: 1796.6286

_N_-(Benzyl)benzyloxycarbonyl-5-aminopentyl 2-azido-2-deoxy-3-_O_-benzyl-4,6-_O_-silylidene-α-D-galactopyranosyl-(1→3)-4,6-_O_-benzylidene-β-D-galactopyranosyl-(1→3)-2-azido-2-deoxy-4,6-_O_-di-benzyl-α-D-galactopyranosyl-(1→3)-2-trichloroacetamido-4-azido-2,4,6-tri-deoxy-α-D-glucopyranosyl (32)

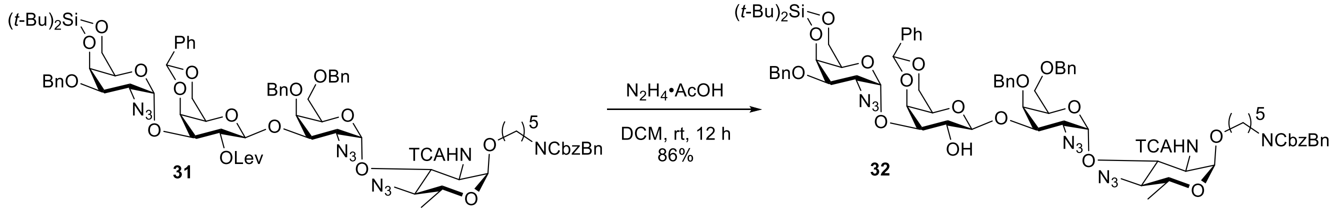

To a solution of **31** (210 mg, 0.118 mmol) in DCM (5 mL) was added N_2H_4•AcOH (109 g, 1.18 mmol) and the mixture was stirred at rt for 12 h. The reaction was quenched by the addition of acetone (1 mL) and evaporated under high vacuum. Residue was purified by silica gel chromatography using 20% ethyl acetate in hexanes to afford product **32** (170 mg, 86%) as colorless syrup. ^{1}H NMR (600 MHz, $CDCl_3$) δ 7.44 (dd, *J* = 7.5, 2.4 Hz, 2H), 7.37 (dd, *J* = 7.2, 2.1 Hz, 2H), 7.35 – 7.14 (m, 26H), 7.12 (d, *J* = 7.3 Hz, 1H), 7.00 – 6.86 (m, 1H), 5.56 (s, 1H), 5.52 (t, *J* = 3.2 Hz, 1H), 5.19 – 5.11 (m, 3H), 4.99 (dd, *J* = 11.9, 2.5 Hz, 1H), 4.69 (d, *J* = 2.4 Hz, 1H), 4.62 – 4.56 (m, 4H), 4.44 (d, *J* = 14.4 Hz, 2H), 4.38 – 4.31 (m, 2H), 4.29 – 4.21 (m, 3H), 4.18 – 4.12 (m, 2H), 4.11 – 4.06 (m, 3H), 4.01 – 3.90 (m, 5H), 3.86 – 3.76 (m, 3H), 3.72 – 3.52 (m, 2H),

3.51 (s, 1H), 3.47 – 3.29 (m, 4H), 3.26 – 3.15 (m, 2H), 3.12 (t, *J* = 7.6 Hz, 1H), 2.61 (bs, 1H), 1.50 – 1.38 (m, 4H), 1.33 (d, *J* = 6.0 Hz, 3H), 1.23 – 1.19 (m, 2H), 1.04 (s, 9H), 1.02 (s, 9H). ^{13}C NMR (151 MHz, $CDCl_3$) δ 162.2, 156.8, 156.3, 138.7, 137.9, 137.9, 137.8, 136.9, 136.8, 128.8, 128.7, 128.6, 128.6, 128.5, 128.4, 128.3, 128.3, 128.2, 128.2, 128.2, 128.1, 128.1, 128.0, 127.9, 127.9, 127.7, 127.7, 127.5, 127.4, 127.3, 126.4, 126.3, 105.3, 101.1, 97.8, 96.4, 95.4, 92.4, 79.3, 77.4, 77.2, 76.9, 76.1, 75.5, 75.4, 74.8, 74.0, 73.4, 71.9, 70.7, 70.2, 69.9, 69.6, 69.5, 69.3, 68.8, 68.3, 68.1, 67.8, 67.3, 67.3, 67.0, 66.6, 60.5, 60.0, 58.0, 54.4, 50.7, 50.4, 47.1, 46.1, 29.8, 29.0, 28.0, 27.9, 27.8, 27.4, 23.5, 23.4, 23.3, 21.2, 20.8, 18.3, 14.3. HR-ESI-MS (m/z): calculated for $C_{82}H_{100}Cl_3N_{11}O_{19}SiNa$ $[M+Na]^+$: 1698.5929, found: 1698.5945

***N*-(Benzyl)benzyloxycarbonyl-5-aminopentyl 2-azido-2-deoxy-3-*O*-benzyl-4,6-*O*-silylidene-α-D-galactopyranosyl-(1→3)-[3,4,6-*O*-benzyl-2-*O*-levulinoyl-β-D-glucopyranosyl-(1→3)]-4,6-*O*-benzylidene-β-D-galactopyranosyl-(1→3)-2-azido-2-deoxy-4,6-*O*-di-benzyl-a-D-galactopyranosyl-(1→3)-2-trichloroacetamido-4-azido-2,4,6-tri-deoxy-α-D-glucopyranoside (33)**

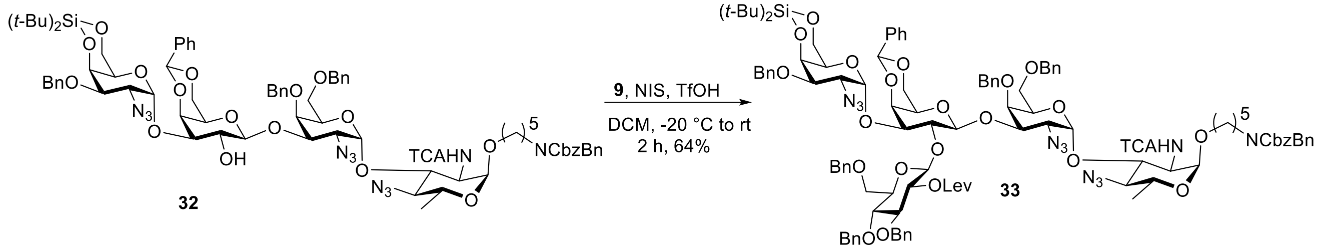

Acceptor **32** (170 mg, 0.101 mmol) and thioglycoside donor **9** (119 mg, 0.182 mmol) were mixed, co-evaporated with toluene (3 x 10 mL) and dried under vacuum for 2 h. The mixture was dissolved in anhydrous DCM (5 mL), freshly activated 4Å molecular sieves were added under a nitrogen atmosphere and stirred for 30 min at rt. The mixture was cooled to -20 °C, to this stirred suspension, NIS (41 mg; 0.182 mmol) and TfOH (3.6 µL, 0.04 mmol) were slowly added. The mixture was brought to 0 °C over 4 h, diluted with DCM (5 mL), quenched with Et_3N, warmed to rt and 4Å molecular sieves were filtered. The filtrate was washed with 10% aqueous $Na_2S_2O_3$, saturated aqueous $NaHCO_3$ and brine. The combined organic layer was dried over Na_2SO_4, filtered and evaporated *in vacuo*. The crude product was purified by silica gel column chromatography using 20% ethyl acetate in hexanes to afford protected pentasaccharide **33** (142 mg, 64%). ^{1}H NMR (600 MHz, $CDCl_3$) δ 7.46 – 7.43 (m, 2H), 7.42 – 7.39 (m, 2H), 7.35 – 7.19 (m,387H), 7.14 (t, *J* = 7.6 Hz, 2H), 7.00 – 6.88 (m, 1H), 5.56 (s, 1H), 5.45 (d, *J* = 3.7 Hz, 1H), 5.21 – 5.15 (m, 3H), 5.15 – 5.11 (m, 2H), 4.87 (d, *J* = 7.5 Hz, 1H), 4.79 (dd, *J* = 16.5, 11.2 Hz, 3H), 4.74 – 4.68 (m, 5H), 4.66 – 4.57 (m, 3H), 4.51 – 4.43 (m, 3H), 4.38 (dd, *J* = 16.4, 11.8 Hz, 2H), 4.32 (d, *J* = 3.3 Hz, 1H), 4.27 – 4.21 (m, 3H), 4.19 (d, *J* = 1.9 Hz, 2H), 4.17 – 4.08 (m, 3H), 4.08 – 4.01 (m, 3H), 3.97 – 3.88 (m, 2H), 3.86 – 3.77 (m, 3H), 3.73 – 3.65 (m, 3H), 3.62 – 3.55 (m, 2H), 3.55 – 3.47 (m, 3H), 3.44 (s, 1H), 3.25 (t, *J* = 9.6 Hz, 3H), 2.56 – 2.49 (m, 1H), 2.40 – 2.31 (m, 2H), 2.20 (dt, *J* = 14.5, 4.4 Hz, 1H), 1.85 (s, 3H), 1.53 (t, *J* = 7.8 Hz, 4H), 1.37 (d, *J* = 6.3 Hz, 3H), 1.33 – 1.29 (m, 1H), 1.06 (s, 9H), 1.05 (s, 9H). ^{13}C NMR (151 MHz, $CDCl_3$) δ 206.4, 172.1, 162.3, 156.3, 139.0, 138.7, 138.5, 138.3, 138.2, 138.0, 137.9, 137.9, 136.8, 129.0, 128.7, 128.6, 128.5, 128.5, 128.4, 128.4, 128.4, 128.3, 128.2, 128.2, 128.1, 128.1, 128.0, 128.0, 127.9, 127.9, 127.8, 127.8,

127.7, 127.7, 127.6, 127.6, 127.5, 127.4, 127.3, 126.3, 103.9, 101.2, 98.9, 98.8, 96.4, 95.1, 92.5, 83.3, 77.8, 77.4, 77.2, 76.9, 76.8, 76.5, 75.8, 75.7, 75.5, 75.4, 75.3, 75.2, 75.0, 74.3, 74.0, 73.5, 73.5, 71.9, 70.4, 70.3, 70.0, 69.8, 69.2, 68.7, 68.3, 68.1, 67.3, 67.3, 67.1, 66.2, 65.3, 65.1, 63.4, 60.5, 59.0, 58.2, 54.5, 50.7, 50.4, 47.1, 46.2, 37.7, 34.9, 34.4, 34.3, 32.1, 31.6, 30.4, 30.3, 29.8, 29.8, 29.7, 29.7, 29.6, 29.6, 29.5, 29.4, 29.3, 29.0, 28.7, 28.0, 28.0, 27.9, 27.5, 27.2, 26.0, 25.0, 23.5, 23.4, 22.8, 21.2, 20.9, 18.2, 14.3, 14.3. HR-ESI-MS (m/z): calculated for $C_{114}H_{134}Cl_3N_{11}O_{26}SiNa$ $[M+Na]^+$: 2228.8234, found: 2228.8242

***N*-(Benzyl)benzyloxycarbonyl-5-aminopentyl 2-azido-2-deoxy-3-*O*-benzyl-4,6-*O*-silylidene-α-D-galactopyranosyl-(1→3)-[3,4,6-*O*-benzyl-2-*O*-levulinoyl-β-D-glucopyranosyl-(1→3)]-β-D-galactopyranosyl-(1→3)-2-azido-2-deoxy-4,6-*O*-di-benzyl-a-D-galactopyranosyl-(1→3)-2-trichloroacetamido-4-azido-2,4,6-tri-deoxy-α-D-glucopyranoside (34)**

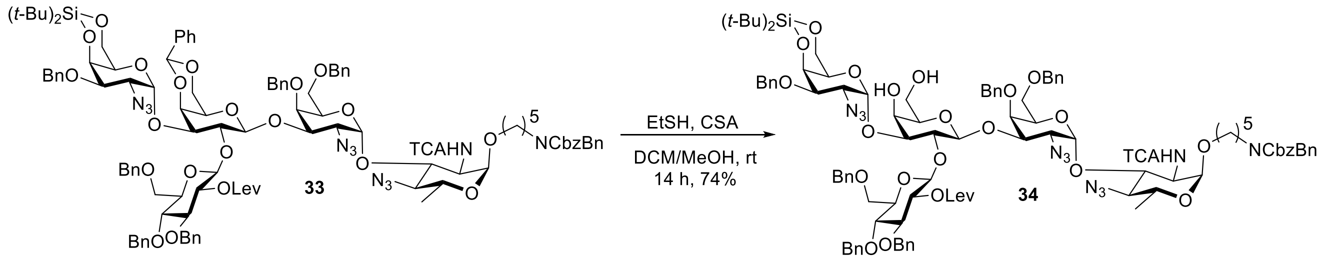

To a solution of compound **34** (100 mg, 0.045 mmol) in a mixture of DCM/MeOH (99 : 1, *v*/*v*, 4 mL) were added ethanethiol (16.2 µL, 0.225 mmol) and CSA (2.1 mg, 0.009 mmol) at rt. After being stirred at rt overnight, the mixture was quenched with Et_3N and concentrated *in vacuo*. The residue was purified by silica gel column chromatography using 30% ethyl acetate in hexanes to afford **34** (71 mg, 74%). ^{1}H NMR (600 MHz, $CDCl_3$) δ 7.49 – 7.44 (m, 2H), 7.40 – 7.38 (m, 1H), 7.36 – 7.23 (m, 32H), 7.22 – 7.15 (m, 4H), 7.09 – 6.99 (m, 1H), 5.61 (d, *J* = 3.5 Hz, 1H), 5.22 – 5.15 (m, 3H), 5.03 – 4.98 (m, 2H), 4.88 – 4.83 (m, 2H), 4.80 – 4.74 (m, 3H), 4.73 – 4.69 (m, 3H), 4.65 – 4.62 (m, 2H), 4.61 – 4.56 (m, 2H), 4.52 – 4.44 (m, 4H), 4.38 – 4.33 (m, 1H), 4.29 – 4.23 (m, 3H), 4.15 (d, *J* = 15.0 Hz, 1H), 4.11 – 4.06 (m, 2H), 4.02 (q, *J* = 4.0, 2.9 Hz, 4H), 4.00 – 3.96 (m, 1H), 3.93 – 3.90 (m, 1H), 3.86 (d, *J* = 9.4 Hz, 1H), 3.83 – 3.79 (m, 2H), 3.77 – 3.71 (m, 3H), 3.68 (td, *J* = 9.4, 1.7 Hz, 1H), 3.62 (d, *J* = 9.2 Hz, 1H), 3.57 – 3.52 (m, 2H), 3.50 (t, *J* = 5.2 Hz, 1H), 3.47 – 3.42 (m, 3H), 3.38 – 3.22 (m, 3H), 3.20 – 3.15 (m, 1H), 2.80 – 2.74 (m, 1H), 2.58 (t, *J* = 6.6 Hz, 2H), 2.52 – 2.45 (m, 1H), 2.43 – 2.35 (m, 1H), 1.97 (s, 3H), 1.56 – 1.42 (m, 4H), 1.38 (d, *J* = 6.2 Hz, 3H), 1.31 – 1.25 (m, 2H), 1.09 (s, 9H), 1.07 (s, 9H). ^{13}C NMR (151 MHz, $CDCl_3$) δ 206.1, 171.8, 162.3, 156.7, 156.2, 138.8, 138.4, 138.3, 138.2, 138.2, 137.9, 128.7, 128.6, 128.5, 128.5, 128.5, 128.4, 128.4, 128.4, 128.1, 128.0, 128.0, 128.0, 127.9, 127.8, 127.8, 127.8, 127.8, 127.7, 127.7, 127.7, 127.6, 127.5, 127.3, 102.5, 98.5, 98.0, 96.4, 94.2, 92.3, 83.1, 78.0, 77.4, 77.3, 77.2, 77.0, 76.9, 76.4, 75.5, 75.4, 75.3, 75.3, 74.9, 74.8, 74.5, 74.4, 73.6, 73.5, 73.4, 70.3, 69.8, 69.5, 69.3, 68.8, 68.1, 68.0, 67.3, 67.3, 67.0, 66.3, 63.0, 60.5, 59.5, 59.3, 54.4, 50.7, 50.4, 47.1, 46.1, 37.7, 29.6, 29.0, 28.1, 27.9, 27.8, 27.7, 27.6, 27.5, 27.4, 27.4, 27.3, 23.4, 23.3, 20.9, 18.2, 14.3. HR-ESI-MS (m/z): calculated for $C_{107}H_{130}Cl_3N_{11}O_{26}SiNa$ $[M+Na]^+$: 2140.7921, found: 2140.7931

N-(Benzyl)benzyloxycarbonyl-5-aminopentyl 2-azido-2-deoxy-3-O-benzyl-4,6-O-silylidene-α-D-galactopyranosyl-(1→3)-[3,4,6-O-benzyl-2-O-levulinoyl-β-D-glucopyranosyl-(1→3)]-β-D-galactopyranosyl-(1→3)-2-azido-2-deoxy-4,6-O-di-benzyl-a-D-galactopyranosyl-(1→3)-2-trichloroacetamido-4-azido-2,4,6-tri-deoxy-α-D-glucopyranoside (35)

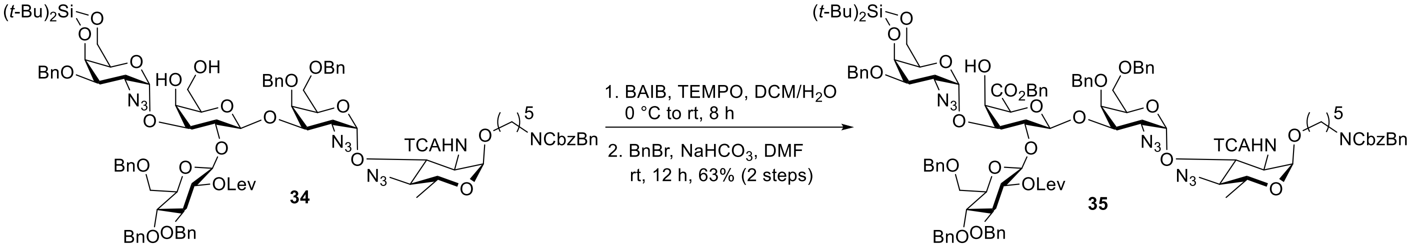

Diol **35** (73 mg, 0.034 mmol) was dissolved in a mixture of DCM/*t*-BuOH/H_2O (2:2:1, *v*/*v*/*v*, 4 mL) and cooled to 0 °C before TEMPO (1.1 mg, 0.007 mmol) and BAIB (27 mg, 0.085 mmol) were added and stirred at 0 °C for 8 h. The reaction mixture was then diluted with DCM (5 mL) and water (5 mL) and the aqueous layer was extracted four times with DCM (10 mL each). Combined organics were dried over Na_2SO_4, filtered, and concentrated. The crude product was dissolved in DMF (1 mL), $NaHCO_3$ (8.6 mg, 0.102 mmol) and BnBr (8 µL, 0.068 mmol) were added at 0 °C. Then the reaction was stirred overnight at rt. After complete consumption of starting material, the mixture was diluted with DCM (20 mL) and washed with brine. The separated organic layer was dried over Na_2SO_4 and concentrated *in vacuo.* The crude was purified by silica gel column chromatography using 20% ethyl acetate in hexanes to furnish product **35** (48 mg, 63% over two steps). ^{1}H NMR (400 MHz, $CDCl_3$) δ 7.47 – 7.43 (m, 2H), 7.39 – 7.23 (m,42H), 7.21 – 7.18 (m, 3H), 5.55 (d, *J* = 3.6 Hz, 1H), 5.29 (d, *J* = 12.2 Hz, 1H), 5.20 – 5.08 (m, 4H), 5.06 – 4.96 (m, 2H), 4.85 – 4.72 (m, 6H), 4.71 – 4.63 (m, 4H), 4.61 (d, *J* = 4.2 Hz, 1H), 4.58 – 4.51 (m, 2H), 4.47 (d, *J* = 7.1 Hz, 3H), 4.43 – 4.37 (m, 2H), 4.32 (q, *J* = 2.6 Hz, 2H), 4.22 (s, 2H), 4.08 – 3.97 (m, 7H), 3.94 (dd, *J* = 9.6, 3.2 Hz, 1H), 3.88 (d, *J* = 9.3 Hz, 1H), 3.81 – 3.66 (m, 6H), 3.62 – 3.48 (m, 4H), 3.41 – 3.33 (m, 2H), 3.24 (dd, *J* = 14.3, 4.9 Hz, 2H), 2.57 (td, *J* = 6.6, 2.7 Hz, 2H), 2.48 – 2.35 (m, 2H), 1.94 (s, 3H), 1.53 – 1.38 (m, 4H), 1.35 (d, *J* = 6.2 Hz, 3H), 1.23 – 1.12 (m, 2H), 1.06 (s, 9H), 1.03 (s, 9H). ^{13}C NMR (101 MHz, $CDCl_3$) δ 206.1, 171.8, 167.0, 162.3, 139.1, 138.5, 138.4, 138.2, 138.2, 138.1, 138.0, 135.4, 128.8, 128.7, 128.6, 128.6, 128.6, 128.5, 128.4, 128.3, 128.2, 128.1, 128.1, 128.0, 127.9, 127.8, 127.8, 127.7, 127.7, 127.6, 127.3, 103.2, 98.5, 98.2, 96.4, 94.6, 92.4, 83.2, 78.0, 77.5, 77.4, 77.2, 77.0, 76.8, 75.6, 75.5, 75.3, 75.1, 74.4, 73.6, 73.5, 73.2, 70.4, 70.0, 69.7, 69.4, 68.7, 68.3, 67.3, 67.3, 67.1, 66.4, 59.3, 59.1, 54.5, 37.8, 29.8, 29.6, 29.0, 28.2, 27.8, 27.5, 23.5, 23.3, 20.9, 18.2, 14.3. HR-ESI-MS (m/z): calculated for $C_{114}H_{134}Cl_3N_{11}O_{27}SiNa$ $[M+Na]^+$: 2244.8183, found: 2244.8189.

5-Aminopentyl 2-acetamido-2-deoxy-α-D-galactopyranosyl-(1→3)-[β-D-glucopyranosyl-(1→2)]-4-O-acetyl-β-D-galactopyranosyl urinate-(1→3)-2-acetamido-2-deoxy-α-D-galactopyranosyl-(1→3)-2,4-di-acetamido-2,4,6-tri-deoxy-α-D-glucopyranosyl (1)

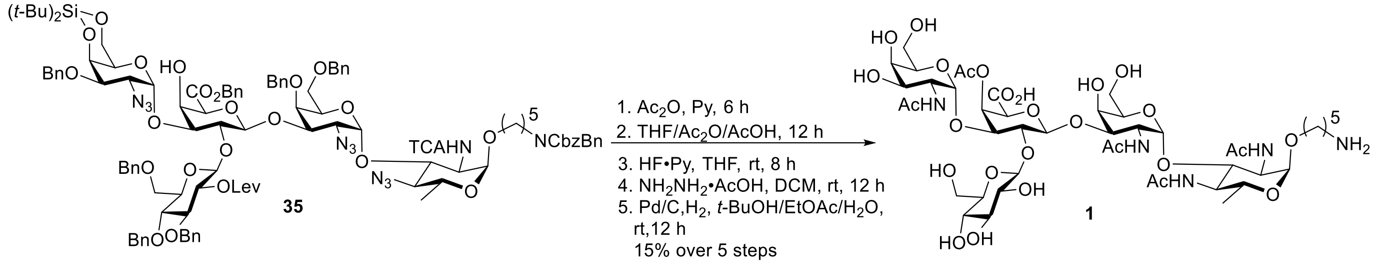

To a solution of pentasaccharide **36** (20 mg, 0.009 mmol) in pyridine (3 mL) was added acetic anhydride (2.5 µL, 0.027 mmol) at 0 °C. After stirring at rt for 6 h, the mixture was concentrated *in vacuo*, diluted with DCM (10 mL) and organic layer was washed with saturated $NaHCO_3$, brine (10 mL) and dried over Na_2SO_4, filtered and concentrated *in vacuo*. The crude was dissolved in the solution of THF/AcOH/Ac_2O (2/1/1, *v*/*v*/*v*, 3 mL) and added freshly activated zinc (200 mg). After stirring 12 h at rt, the mixture was diluted with ethyl acetate and filtered over Celite® 353, evaporated *in vacuo* and dried in vacuum for 2 h. To a solution of crude in THF (3 mL) was added HF•Py (70% HF, 12 µL, 0.09 mmol). After TLC analysis indicated complete conversion of the starting material (~8 hours), the reaction was quenched with Et_3N (0.5 mL). The mixture was concentrated, dissolved in EtOAc and subsequently washed with saturated aqueous $NaHCO_3$ and brine. The aqueous layers were then extracted with EtOAc, combined organic layers were dried over Na_2SO_4, filtered and concentrated *in vacuo*. The crude was dissolved in DCM (3 mL), N_2H_4•AcOH (8 mg, 0.09 mmol) was added and the mixture was stirred at rt for 12 h. The reaction was quenched by the addition of acetone (1 mL) and the solvent was removed under vacuum. The crude was filtered through a small bed of silica gel, evaporated *in vacuo*. The triol intermediate was dissolved in the solution of EtOAc/*t*-BuOH/H_2O (2/1/1, *v*/*v*/*v*, 2 mL) and Pd/C (50 mg) was added to the solution. After stirring for 12 h under hydrogen atmosphere (1 atm, balloon), the mixture was filtered through a PTFE filter (0.45 µm pore size) and concentrated. The crude material was purified by HPLC (Hypercarb column, 150x10 mm, H_2O (0.1% formic acid) isocratic (5 min), linear gradient to 30% ACN (30 min), linear gradient to 100% ACN (10 min) and lyophilized to obtain pure pentasaccharide **1** (1.5 mg, 15%). ^{1}H NMR (600 MHz, D_2O) δ 5.63 (d, *J* = 2.9 Hz, 1H), 5.12 (d, *J* = 3.7 Hz, 1H), 5.08 (d, *J* = 4.0 Hz, 1H), 4.74 (s, 1H), 4.73 (s, 1H), 4.57 (d, *J* = 7.8 Hz, 2H), 4.26 (d, *J* = 2.7 Hz, 1H), 4.16 (dd, *J* = 11.2, 4.0 Hz, 1H), 4.15 – 4.12 (m, 1H), 4.08 (s, 1H), 4.04 (d, *J* = 3.0 Hz, 1H), 4.01 – 3.96 (m, 2H), 3.89 (d, *J* = 3.2 Hz, 1H), 3.87 – 3.84 (m, 2H), 3.81 (d, *J* = 10.8 Hz, 1H), 3.74 – 3.69 (m, 6H), 3.64 (d, *J* = 3.7 Hz, 1H), 3.63 – 3.60 (m, 1H), 3.60 – 3.55 (m, 2H), 3.42 – 3.39 (m, 1H), 3.34 (t, *J* = 9.2 Hz, 2H), 3.19 (d, *J* = 9.5 Hz, 1H), 3.13 (dd, *J* = 9.4, 7.8 Hz, 1H), 2.92 – 2.89 (m, 2H), 2.02 (d, *J* = 0.8 Hz, 3H), 1.94 (s, 9H), 1.88 (d, *J* = 0.8 Hz, 3H), 1.61 – 1.55 (m, 4H), 1.38 – 1.31 (m, 2H), 1.06 (d, *J* = 5.9 Hz, 3H). ^{13}C NMR (176 MHz, D_2O) δ 174.4, 174.2, 173.4, 173.3, 172.1, 171.0, 102.3, 102.0, 96.8, 96.5, 92.2, 76.8, 76.1, 75.8, 73.5, 73.3, 73.1, 70.9, 70.5, 70.2, 68.8, 68.2, 67.7, 67.4, 67.3, 61.6, 61.4, 60.9, 52.8, 49.1, 48.2, 39.4, 28.0, 26.5, 22.8, 22.3, 22.1, 22.0, 20.2, 16.3. HR-ESI-MS (m/z): calculated for $C_{45}H_{76}N_5O_{27}$ $[M+H]^+$: 1118.4728, found: 1118.4741

5-Aminopentyl 2-acetamido-2-deoxy-α-D-galactopyranosyl-(1→3)-[β-D-glucopyranosyl-(1→2)]-β-D-galactopyranosyl urinate-(1→3)-2-acetamido-2-deoxy-α-D-galactopyranosyl-(1→3)-2,4-di-acetamido-2,4,6-tri-deoxy-α-D-glucopyranosyl (2)

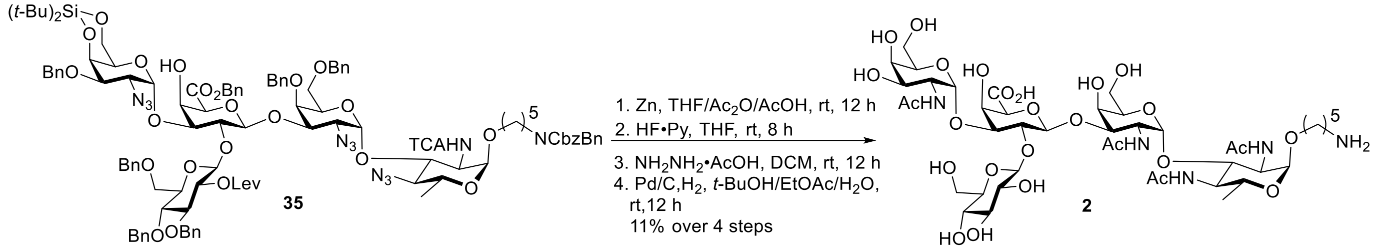

Pentasaccharide **36** (20 mg, 0.009 mmol) was dissolved in a mixture of THF/AcOH (2/1/1, *v*/*v*/*v*, 3 mL) and added freshly activated zinc (200 mg). After stirring for 12 h at rt, the mixture was diluted with ethyl acetate and filtered over Celite® 353, evaporated *in vacuo* and dried in vacuum for 2h. To a solution of crude in THF (3 mL) was added HF•Py (70% HF, 12 µL, 0.09 mmol). After TLC analysis indicated complete conversion of the starting material (~8 hours), the reaction was quenched with Et_3N (0.5 mL). The mixture was concentrated, dissolved in EtOAc and subsequently washed with saturated aqueous $NaHCO_3$ and brine. The aqueous layers were then extracted with EtOAc, combined organic layers were dried over Na_2SO_4, filtered and concentrated *in vacuo*. The crude was dissolved in DCM (2 mL), N_2H_4•AcOH (8 mg, 0.09 mmol) was added and the mixture was stirred at rt for 12 h. The reaction was quenched by the addition of acetone (1 mL) and the solvent was removed under vacuum. The crude was filtered through a small bed of silica gel, evaporated *in vacuo*. The triol intermediate was dissolved in the solution of EtOAc/*t*-BuOH/H_2O (2/1/1, *v*/*v*/*v*, 2 mL) and Pd/C (50 mg) was added to the solution. After stirring for 12 h under hydrogen atmosphere (1 atm, balloon), the mixture was filtered through a PTFE filter (0.45 µm pore size) and concentrated. The crude material was purified by HPLC (Hypercarb column, 150 x 10 mm, H_2O (0.1% formic acid) isocratic (5 min), linear gradient to 30% ACN (30 min), linear gradient to 100% ACN (10 min)) and lyophilized to obtain pure pentasaccharide **2** (1.1 mg, 11%). ^{1}H NMR (700 MHz, D_2O) δ 5.19 (dd, *J* = 18.6, 3.9 Hz, 2H), 4.86 – 4.82 (m, 1H), 4.72 (d, *J* = 7.8 Hz, 1H), 4.58 (d, *J* = 7.3 Hz, 1H), 4.48 – 4.44 (m, 1H), 4.38 (d, *J* = 3.0 Hz, 1H), 4.32 (dd, *J* = 8.2, 4.2 Hz, 1H), 4.27 (dd, *J* = 11.1, 3.8 Hz, 2H), 4.12 – 4.08 (m, 1H), 4.06 – 3.98 (m, 6H), 3.95 (t, *J* = 6.4 Hz, 1H), 3.90 (d, *J* = 12.0 Hz, 1H), 3.85 – 3.78 (m, 6H), 3.75 (dd, *J* = 12.1, 4.1 Hz, 1H), 3.70 (d, *J* = 9.1 Hz, 1H), 3.66 (dd, *J* = 12.3, 7.0 Hz, 1H), 3.54 – 3.49 (m, 1H), 3.48 – 3.41 (m, 2H), 3.31 (t, *J* = 9.5 Hz, 1H), 3.24 (t, *J* = 8.8 Hz, 1H), 3.01 (t, *J* = 7.8 Hz, 2H), 2.05 (s, 2H), 2.05 (s, 3H), 2.04 (s, 3H), 1.98 (s, 3H), 1.73 – 1.63 (m, 4H), 1.45 (q, *J* = 8.2 Hz, 2H), 1.16 (d, *J* = 5.4 Hz, 3H). ^{13}C NMR (176 MHz, D_2O) δ 174.9, 174.7, 174.3, 173.3, 102.5, 102.0, 96.8, 96.5, 91.8, 77.1, 76.1, 76.0, 75.4, 75.4, 73.4, 73.2, 71.1, 70.5, 70.1, 68.7, 68.0, 67.9, 67.7, 65.0, 61.4, 61.3, 61.0, 52.8, 49.5, 48.1, 39.4, 28.0, 26.5, 22.8, 22.3, 22.1, 22.0, 16.3. HR-ESI-MS (m/z): calculated for $C_{43}H_{74}N_5O_{26}$ $[M+H]^+$: 1076.4622, found: 1076.4631

N-(Benzyl)benzyloxycarbonyl-5-aminopentyl 2-azido-4,6-O-di-benzyl-2-deoxy-3-O-(2-napthylmethyl)-α-D-galactopyranoside (36)

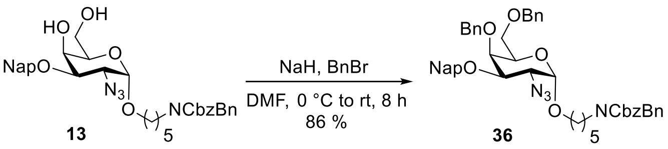

To a solution of diol **13** (653 mg, 1.12 mmol) in DMF (2 mL) was cooled to 0 °C and treated with NaH (60% disp.) (112 mg, 2.80 mmol). The mixture was stirred for 20 min before benzyl bromide (0.332 mL, 2.80 mmol) was added. After stirring for 8 h, quenched with aqueous saturated NH_4Cl and diluted with EtOAc. The organic layer was washed with H_2O and brine, dried over Na_2SO_4 and concentrated *in vacuo*. The crude was purified by silica gel column chromatography using 8% ethyl acetate in hexanes to yield the desired compound **36** (734 mg, 86%). ^{1}H NMR (400 MHz, $CDCl_3$) δ 7.91 – 7.80 (m, 4H), 7.55 (dd, *J* = 8.5, 1.7 Hz, 1H), 7.52 – 7.48 (m, 2H), 7.42 – 7.26 (m, 19H), 7.20 (d, *J* = 7.2 Hz, 1H), 5.21 (d, *J* = 12.6 Hz, 2H), 4.98 – 4.84 (m, 4H), 4.61 (d, *J* = 11.3 Hz, 1H), 4.52 (t, *J* = 6.2 Hz, 3H), 4.46 (d, *J* = 11.7 Hz, 1H), 4.11 (s, 1H), 4.07 – 3.94 (m, 2H), 3.91 (dd, *J* = 10.6, 3.5 Hz, 1H), 3.74 – 3.54 (m, 3H), 3.50 – 3.35 (m, 1H), 3.34 – 3.18 (m, 2H), 1.69 – 1.47 (m, 54), 1.43 – 1.31 (m, 2H). ^{13}C NMR (101 MHz, $CDCl_3$) δ 156.8, 156.3, 138.4, 138.0, 137.9, 137.0, 136.9, 135.2, 133.4, 133.1, 128.6, 128.6, 128.5, 128.4, 128.2, 128.1, 128.0, 127.9, 127.8, 127.4, 127.3, 126.6, 126.2, 126.1, 125.8, 98.2, 77.5, 77.4, 77.2, 76.8, 74.9, 73.6, 73.5, 72.3, 69.7, 68.9, 68.2, 67.2, 59.9, 50.6, 50.3, 47.2, 46.2, 29.8, 29.2, 27.9, 27.5, 23.5, 1.1. HR-ESI-MS (m/z): calculated for $C_{51}H_{54}N_4O_7Na$ $[M+Na]^+$: 857.3890, found: 857.3898

N-(Benzyl)benzyloxycarbonyl-5-aminopentyl 2-azido-4,6-O-di-benzyl-2-deoxy-α-D-galactopyranoside (37)

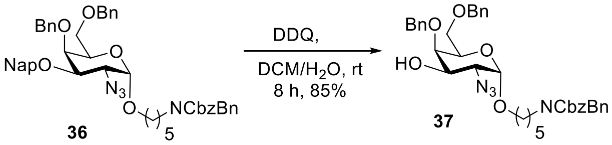

To a well stirred emulsion of **36** (500 g, 0.598 mmol) in DCM and H_2O (7/1, *v/v*, 8 mL) was added DDQ (203 mg, 0.897 mmol) and stirred at rt for 8 h. The mixture was diluted with DCM (20 mL) and washed (2 x 20 mL) with 10% aqueous $Na_2S_3O_3$ to reduce the remaining DDQ. The organic layer was dried over Na_2SO_4, filtered and concentrated *in vacuo.* The residue was purified by silica column chromatography using 10% ethyl acetate in hexanes to afford product **37** (353 mg, 85%) as yellowish liquid. ^{1}H NMR (400 MHz, $CDCl_3$) δ 7.30 – 7.11 (m, 19H), 7.07 (d, *J* = 7.3 Hz, 1H), 5.08 (d, *J* = 13.4 Hz, 2H), 4.81 – 4.73 (m, 1H), 4.59 (d, *J* = 1.8 Hz, 2H), 4.48 – 4.35 (m, 4H), 3.98 – 3.86 (m, 2H), 3.82 (d, *J* = 4.7 Hz, 1H), 3.61 – 3.43 (m, 3H), 3.33 (dd, *J* = 10.6, 3.5 Hz, 2H), 3.21 – 3.02 (m, 2H), 2.16 (bs, 1H), 1.57 – 1.36 (m, 4H), 1.29 – 1.20 (m, 2H). ^{13}C NMR (101 MHz, $CDCl_3$) δ 156.8, 156.2, 138.0, 137.9, 137.9, 137.7, 136.9, 136.8, 128.7, 128.6, 128.5, 128.5, 128.3, 128.3, 128.3, 128.2, 128.1, 128.0, 128.0, 127.9, 127.9, 127.4, 127.3, 127.2, 98.2, 77.5, 77.4, 77.2, 76.9, 76.8, 75.5, 73.6, 69.3, 68.5, 68.5, 68.2, 68.1, 67.2, 67.2, 67.2, 61.1, 50.5, 50.2, 47.1, 46.2, 29.8, 29.1, 27.9, 27.5, 23.4, 23.3. HR-ESI-MS (m/z): calculated for $C_{40}H_{46}N_4O_7Na$ $[M+Na]^+$: 717.3264, found: 717.3271

N-(Benzyl)benzyloxycarbonyl-5-aminopentyl 4-O-acetyl-6-O-benzyl-3-tert-butyl dimethylsilyl-2-O-levulinoy-β-D-galactopyranosyl uronate-(1→3)-2-azido-4,6-O-di-benzyl-2-deoxy-α-D-galactopyranoside (38)

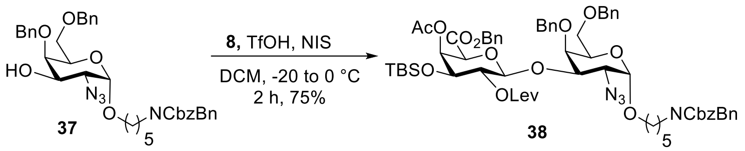

Acceptor **37** (50 mg, 0.072 mmol) and donor **8** (63 mg 0.108 mmol) were mixed, co-evaporated with toluene (3 x 10 mL), dried under high vacuum overnight and then dissolved in DCM under nitrogen atmosphere. Freshly activated 4Å molecular sieves were added and stirred for 30 min at rt. The mixture was cooled to -20 °C, to this stirred suspension, NIS (24 mg; 0.108 mmol) and TfOH (3.2 µL, 36 µmol) were slowly added. After being stirred at -20 °C for 2 h, diluted with DCM (10 mL), quenched with Et_3N, warmed to rt and 4Å molecular sieves were filtered. The filtrate was washed with 10% aqueous $Na_2S_2O_3$, saturated aqueous $NaHCO_3$ and brine. The combined organic layer was dried over Na_2SO_4, filtered and evaporated *in vacuo*. The crude product was purified by silica gel column chromatography using 10% ethyl acetate in hexanes to afford the desired protected disaccharide **38** (66 mg, 75%) ^{1}H NMR (700 MHz, $CDCl_3$) δ 7.34 (d, *J* = 7.5 Hz, 2H), 7.32 – 7.16 (m, 24H), 7.10 (d, *J* = 7.5 Hz, 1H), 5.52 (d, *J* = 3.6 Hz, 1H), 5.17 – 5.06 (m, 4H), 5.02 – 4.95 (m, 2H), 4.84 (d, *J* = 7.2 Hz, 1H), 4.64 (d, *J* = 8.0 Hz, 1H), 4.57 (d, *J* = 11.6 Hz, 1H), 4.45 – 4.36 (m, 3H), 4.30 (d, *J* = 11.8 Hz, 1H), 4.23 (s, 1H), 4.17 (d, *J* = 3.0 Hz, 1H), 4.01 (d, *J* = 10.8 Hz, 1H), 3.89 – 3.82 (m, 2H), 3.67 (d, *J* = 10.7 Hz, 1H), 3.63 – 3.53 (m, 1H), 3.48 – 3.43 (m, 1H), 3.40 – 3.27 (m, 2H), 3.24 – 3.10 (m, 2H), 2.72 – 2.66 (m, 1H), 2.66 – 2.59 (m, 3H), 2.08 (s, 3H), 1.81 (s, 3H), 1.56 – 1.40 (m, 4H), 1.30 – 1.19 (m, 2H), 0.76 (s, 9H), 0.05 (s, 3H), 0.03 (s, 3H). ^{13}C NMR (176 MHz, $CDCl_3$) δ 206.3, 171.6, 169.6, 166.2, 156.8, 156.3, 139.0, 138.2, 138.0, 135.1, 129.0, 128.8, 128.8, 128.7, 128.7, 128.6, 128.6, 128.5, 128.5, 128.4, 128.3, 128.0, 127.9, 127.8, 127.7, 127.6, 127.4, 127.3, 102.2, 98.3, 77.7, 77.3, 77.2, 77.0, 76.4, 74.9, 73.5, 72.6, 72.0, 70.7, 70.5, 69.9, 69.5, 68.2, 68.1, 67.6, 67.3, 59.8, 50.6, 50.3, 47.2, 46.1, 37.9, 30.0, 29.8, 29.2, 29.2, 28.0, 27.9, 27.6, 25.7, 25.5, 25.5, 25.4, 23.5, 23.4, 20.5, 18.0, 17.8, 17.6, 1.1, -4.5, -4.6, -4.8, -4.8, -4.9, -5.1. HR-ESI-MS (m/z): calculated for $C_{66}H_{82}N_4O_{16}SiNa$ $[M+Na]^+$: 1237.5393, found: 1237.5383

N-(Benzyl)benzyloxycarbonyl-5-aminopentyl 4-O-acetyl-6-O-benzyl-3-tert-butyl dimethylsilyl-β-D-galactopyranosyl uronate-(1→3)-2-azido-4,6-O-di-benzyl-2-deoxy-α-D-galactopyranoside (39)

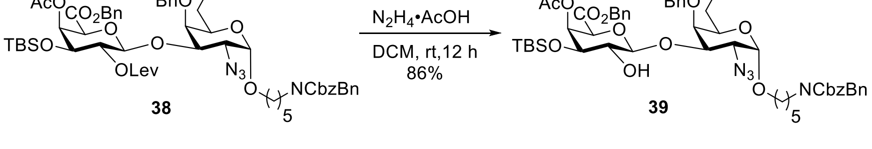

To a solution of disaccharide **38** (72 mg, 0.059 mmol) in DCM (5 mL) was added N_2H_4•AcOH (27 mg, 0.296 mmol) and the mixture was stirred at rt overnight. The reaction was quenched by the addition of acetone (1 mL) and the solvent was removed under vacuum. The residue was

purified by silica gel chromatography using 15% ethyl acetate in hexanes to give product **39** (56 mg, 86%) as colorless syrup. ^{1}H NMR (700 MHz, $CDCl_3$) δ 7.29 (d, *J* = 7.4 Hz, 2H), 7.26 – 7.11 (m, 24H), 7.05 (d, *J* = 7.5 Hz, 1H), 5.45 (d, *J* = 3.7 Hz, 1H), 5.05 (q, *J* = 9.8 Hz, 3H), 4.92 (dd, *J* = 12.0, 5.1 Hz, 2H), 4.80 (d, *J* = 10.9 Hz, 1H), 4.57 (d, *J* = 12.0 Hz, 1H), 4.45 (d, *J* = 7.6 Hz, 1H), 4.40 – 4.30 (m, 3H), 4.24 (d, *J* = 11.9 Hz, 1H), 4.19 – 4.15 (m, 2H), 4.03 – 3.96 (m, 1H), 3.86 – 3.80 (m, 1H), 3.73 (dd, *J* = 10.7, 3.6 Hz, 1H), 3.67 (dd, *J* = 9.3, 3.7 Hz, 1H), 3.59 – 3.48 (m, 2H), 3.40 (dd, *J* = 9.7, 6.3 Hz, 1H), 3.32 – 3.21 (m, 2H), 3.19 – 3.03 (m, 2H), 2.37 (bs, 1H), 1.74 (s, 3H), 1.52 – 1.36 (m, 4H), 1.25 – 1.17 (m, 2H), 0.76 (s, 9H), 0.03 (s, 3H), 0.00 (s, 3H). ^{13}C NMR (176 MHz, $CDCl_3$) δ 169.6, 166.5, 156.9, 156.3, 139.0, 138.2, 138.0, 137.0, 136.9, 135.2, 129.0, 128.8, 128.8, 128.7, 128.7, 128.6, 128.6, 128.5, 128.4, 128.4, 128.3, 128.3, 128.2, 128.0, 127.9, 127.9, 127.8, 127.7, 127.6, 127.4, 127.3, 104.9, 98.0, 79.4, 77.3, 77.2, 77.1, 77.0, 76.6, 75.0, 73.5, 72.7, 72.4, 72.2, 70.6, 69.8, 69.5, 68.2, 68.1, 67.5, 67.3, 60.5, 59.8, 50.6, 50.3, 47.2, 46.2, 29.2, 29.2, 28.0, 27.6, 25.7, 23.5, 23.4, 21.2, 20.5, 18.2, 14.3, 1.1, -4.5, -4.9. HR-ESI-MS (m/z): calculated for $C_{61}H_{76}N_4O_{14}SiNa$ $[M+Na]^+$: 1139.5025, found: 1139.5033

_N_-(Benzyl)benzyloxycarbonyl-5-aminopentyl 3,4,6-tri-_O_-benzyl-2-_O_-levulinoyl-β-D-glucopyranosyl-(1→2)-4-_O_-acetyl-6-_O_-benzyl-3-_tert_-butyl dimethylsilyl–β-D-galactopyranosyl uronate-(1→3)-2-azido-4,6-di-_O_-benzyl-2-deoxy-α-D-galactopyranoside (40)

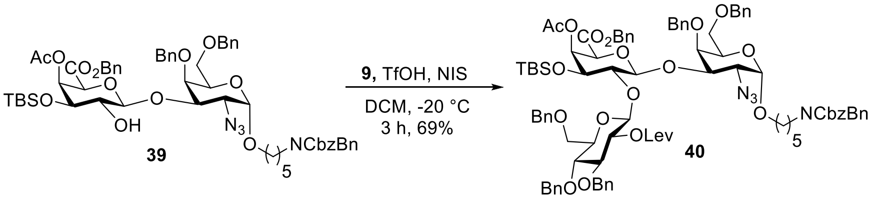

Acceptor **39** (48 mg, 0.072 mmol) and donor **9** (42 mg, 0.064 mmol) were mixed, co-evaporated with toluene (3 x 10 mL) and dried under high vacuum for 2 h. Freshly activated 4Å molecular sieves were added, dissolved in anhydrous DCM (12 mL) under a nitrogen atmosphere and stirred for 30 min at rt. The mixture was cooled to -20 °C, to this stirred suspension, NIS (16.4 mg, 0.073 mmol) and TfOH (1.5 µL, 17 µmol) were slowly added. After being stirred at -20 °C for 3 h, diluted with DCM (10 mL), quenched with Et_3N, warmed to rt and 4Å molecular sieves were filtered. The filtrate was washed with 10% aqueous $Na_2S_2O_3$, saturated aqueous $NaHCO_3$ and brine. The combined organic layer was dried over Na_2SO_4, filtered and evaporated *in vacuo*. The crude product was purified by silica gel column chromatography using 15% ethyl acetate in hexanes to afford the desired protected trisaccharide **40** (49 mg, 69%). ^{1}H NMR (700 MHz, $CDCl_3$) δ 7.36 (d, *J* = 7.4 Hz, 2H), 7.30 – 7.18 (m, 36H), 7.15 – 7.09 (m, 4H), 5.38 (d, *J* = 3.3 Hz, 1H), 5.10 (t, *J* = 13.2 Hz, 3H), 5.07 – 5.03 (m, 1H), 5.01 (d, *J* = 11.9 Hz, 1H), 4.96 – 4.92 (m, 2H), 4.73 – 4.67 (m, 4H), 4.63 (dd, *J* = 15.4, 11.5 Hz, 2H), 4.50 (dd, *J* = 13.6, 11.3 Hz, 2H), 4.45 – 4.38 (m, 4H), 4.32 – 4.28 (m, 2H), 4.21 (d, *J* = 3.0 Hz, 1H), 4.09 (s, 1H), 4.02 – 3.95 (m, 3H), 3.90 – 3.83 (m, 1H), 3.69 – 3.60 (m, 3H), 3.55 – 3.51 (m, 3H), 3.49 – 3.44 (m, 2H), 3.35 – 3.25 (m, 2H), 3.23 – 3.10 (m, 2H), 2.62 – 2.51 (m, 2H), 2.49 – 2.45 (m, 1H), 2.34 – 2.28 (m, 1H), 2.04 (s, 3H), 1.77 (s, 3H), 1.61 – 1.48 (m, 4H), 1.31 – 1.22 (m, 2H), 0.82 (s, 9H), 0.12 (s, 3H), 0.09 (s, 3H). ^{13}C NMR (176 MHz, $CDCl_3$) δ 206.4, 171.3, 169.9, 166.7, 139.3, 138.5, 138.4, 138.3, 138.1, 135.2,

129.0, 128.7, 128.7, 128.7, 128.6, 128.6, 128.5, 128.5, 128.4, 128.4, 128.3, 128.3, 128.2, 128.1, 128.0, 128.0, 127.9, 127.9, 127.8, 127.7, 127.7, 127.6, 127.6, 127.5, 127.4, 102.2, 98.5, 83.4, 78.2, 77.3, 77.3, 77.2, 77.0, 76.7, 75.3, 75.1, 74.9, 74.9, 73.9, 73.6, 73.4, 73.1, 72.2, 71.3, 69.7, 69.7, 69.4, 67.6, 67.3, 59.6, 50.6, 50.4, 47.3, 46.3, 38.0, 30.0, 29.2, 28.1, 28.0, 27.6, 26.0, 23.5, 20.6, 18.0, 14.3, 1.2, -4.2, -4.3. HR-ESI-MS (m/z): calculated for $C_{93}H_{110}N_4O_{21}SiNa$ $[M+Na]^+$: 1669.7335, found: 1669.7331

_N_-(Benzyl)benzyloxycarbonyl-5-aminopentyl 3,4,6-tri-_O_-benzyl-β-D-glucopyranosyl-(1→2) -4-_O_-acetyl-6-_O_-benzyl-β-D-galactopyranosyl uronate-(1→3)-2-azido-4,6-di-_O_-benzyl-2-deoxy-α-D-galactopyranoside (42)

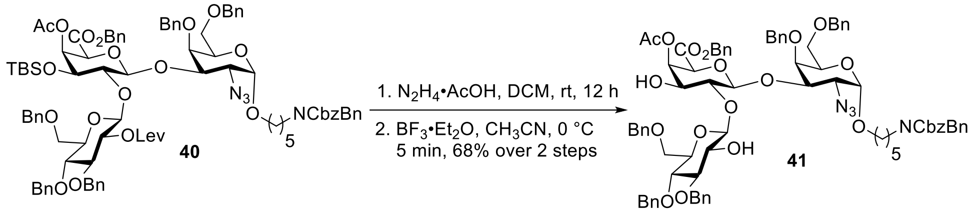

To a solution of trisaccharide **40** (34 mg, 0.021 mmol) in DCM (4 mL) was added N_2H_4•AcOH (10 mg, 0.103 mmol) and the mixture was stirred at rt for 12 h. The reaction was quenched by the addition of acetone (2 mL) and the solvent was removed under vacuum. The crude was dissolved in anhydrous ACN (4 mL), cooled to 0 °C and BF_3•Et_2O (3.4 µmol, 0.027 mmol) was added. After stirring for 5 min, saturated aqueous $NaHCO_3$ was added and extracted the organic layer with DCM. The combined layers were dried over Na_2SO_4, filtered and evaporated *in vacuo*. The crude was purified by silica gel column chromatography using 45% ethyl acetate in hexanes to afford diol **41** (20 mg, 68% over two steps). ^1H NMR (700 MHz, $CDCl_3$) δ 7.37 (d, *J* = 7.4 Hz, 2H), 7.34 – 7.30 (m, 9H), 7.28 – 7.20 (m, 26H), 7.12 (dd, *J* = 7.4, 2.0 Hz, 3H), 5.68 (d, *J* = 3.5 Hz, 1H), 5.13 (d, *J* = 13.1 Hz, 3H), 5.07 – 5.02 (m, 1H), 5.00 – 4.93 (m, 2H), 4.87 – 4.82 (m, 1H), 4.80 – 4.72 (m, 3H), 4.66 (d, *J* = 11.7 Hz, 1H), 4.57 (d, *J* = 8.2 Hz, 1H), 4.49 (dd, *J* = 11.5, 2.2 Hz, 2H), 4.45 – 4.38 (m, 4H), 4.35 – 4.30 (m, 2H), 4.27 (s, 1H), 4.15 (s, 1H), 4.10 (t, *J* = 9.2 Hz, 1H), 3.95 – 3.90 (m, 1H), 3.84 – 3.78 (m, 2H), 3.72 – 3.66 (m, 2H), 3.63 – 3.55 (m, 6H), 3.51 – 3.44 (m, 2H), 3.38 (dd, *J* = 9.7, 6.0 Hz, 1H), 3.25 – 3.12 (m, 2H), 1.76 (s, 3H), 1.59 – 1.49 (m, 4H), 1.32 – 1.25 (m, 2H). ^{13}C NMR (176 MHz, $CDCl_3$) δ 170.0, 166.3, 156.9, 156.3, 139.0, 138.9, 138.2, 138.2, 138.1, 138.0, 137.0, 136.9, 135.1, 129.0, 128.8, 128.7, 128.6, 128.6, 128.5, 128.5, 128.5, 128.4, 128.1, 128.1, 128.0, 128.0, 127.9, 127.8, 127.8, 127.8, 127.7, 127.7, 127.7, 127.4, 127.3, 102.4, 101.6, 98.4, 84.4, 77.6, 77.3, 77.2, 77.0, 76.9, 75.7, 75.6, 75.3, 75.3, 75.2, 73.6, 73.5, 72.6, 72.6, 71.0, 70.0, 69.8, 69.4, 68.6, 68.3, 68.1, 67.6, 67.3, 50.6, 50.4, 47.2, 46.1, 29.8, 29.3, 29.2, 27.9, 27.6, 23.5, 23.4, 20.6. HR-ESI-MS (m/z): calculated for $C_{87}H_{96}N_4O_{21}Na$ $[M+Na]^+$: 1555.6465, found: 1555.6461.

5-Aminopentyl β-D-glucopyranosyl-(1→2) -4-*O*-acetyl-β-D-galactopyranosyl uronate-(1→3)-2-acetamido-2-deoxy-α-D-galactopyranoside (3)

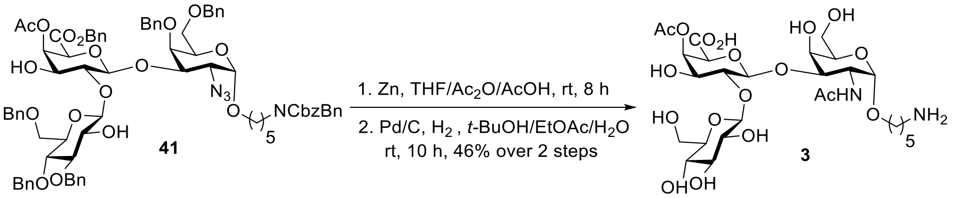

The diol **41** (16 mg, 0.011 mmol) intermediate was dissolved in the solution of THF/Ac_2O/AcOH (3/2/1, *v*/*v*/*v*, 3 mL) and added freshly activated zinc (200 mg). After stirring for 8 h at rt, the mixture was diluted with ethyl acetate and filtered over Celite® 353 and evaporated *in vacuo*. The crude product was dissolved in the solution of EtOAc/*t*-BuOH/H_2O (2/1/1, *v*/*v*/*v*, 2 mL) and Pd/C (50 mg) was added to the solution. After stirring for 10 h under hydrogen atmosphere (1 atm, balloon), the mixture was filtered through a PTFE filter (0.45 µm pore size) and concentrated. The crude material was purified by HPLC (Hypercarb column, 150 x10 mm, H_2O (0.1% formic acid) isocratic (5 min), linear gradient to 10% ACN (30 min), linear gradient to 100% ACN (10 min)) and lyophilized to obtain pure trisaccharide **3** (4.8 mg, 46% over two steps).^{1}H NMR (700 MHz, D_2O) δ 5.55 (dd, *J* = 3.7, 1.2 Hz, 1H), 4.89 (d, *J* = 3.8 Hz, 1H), 4.81 (s, 1H), 4.67 (d, *J* = 7.8 Hz, 1H), 4.35 – 4.30 (m, 2H), 4.17 (d, *J* = 1.3 Hz, 1H), 4.12 (dd, *J* = 9.6, 3.7 Hz, 1H), 4.05 (dd, *J* = 11.1, 3.1 Hz, 1H), 4.00 – 3.97 (m, 1H), 3.92 – 3.89 (m, 1H), 3.84 (dd, *J* = 9.6, 7.8 Hz, 1H), 3.80 – 3.75 (m, 2H), 3.71 (ddd, *J* = 10.0, 4.5, 1.6 Hz, 2H), 3.52 – 3.47 (m, 2H), 3.40 – 3.36 (m, 2H), 3.26 – 3.17 (m, 2H), 3.01 (t, *J* = 7.6 Hz, 2H), 2.14 (s, 3H), 2.04 (s, 3H), 1.72 – 1.62 (m, 4H), 1.48 – 1.44 (m, 2H). ^{13}C NMR (176 MHz, D_2O) δ 173.8, 173.7, 173.1, 102.0, 101.9, 97.1, 76.8, 76.3, 76.0, 75.8, 73.7, 73.6, 72.6, 72.4, 70.8, 69.6, 68.5, 67.6, 61.3, 60.8, 48.8, 46.7, 39.4, 28.0, 26.4, 22.4, 22.3, 20.3, 8.2. HR-ESI-MS (m/z): calculated for $C_{27}H_{47}N_2O_{18}$ $[M+H]^+$: 687.2824, found: 687.2829

***N*-(Benzyl)benzyloxycarbonyl-5-aminopentyl 4-*O*-acetyl-6-*O*-benzyl-2-*O*-levulinoy-β-D-galactopyranosyl uronate-(1→3)-2-azido-4,6-di-*O*-benzyl-2-deoxy-α-D-galactopyranoside (42)**

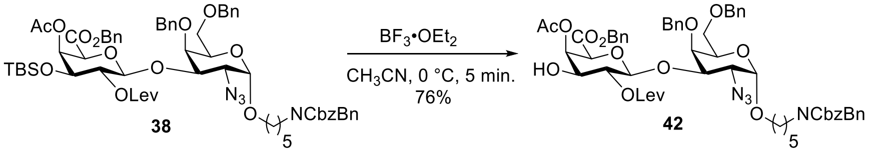

Disaccharide **38** (36 mg, 0.03 mmol) was dissolved in anhydrous ACN (5 mL) under a nitrogen atmosphere at 0 °C and to this, BF_3•OEt_2 (4.8 µL, 0.038 mmol) was added dropwise. After stirring for 5 min, quenched with saturated aqueous $NaHCO_3$, extracted the organic layer with DCM. The combined organic layer was dried over Na_2SO_4, filtered and evaporated *in vacuo*. The crude was purified by silica gel column chromatography using 15% ethyl acetate in hexanes to afford compound **42** (24 mg, 76%). ^{1}H NMR (400 MHz, $CDCl_3$) δ 7.37 – 7.32 (m, 2H), 7.30 – 7.26 (m, 4H), 7.25 – 7.14 (m, 18H), 7.09 (d, *J* = 7.1 Hz, 1H), 5.61 (dd, *J* = 3.5, 1.3 Hz, 1H), 5.13 – 5.07 (m, 3H), 5.01 (d, *J* = 4.7 Hz, 1H), 4.97 (d, *J* = 11.6 Hz, 1H), 4.81 (s, 1H), 4.73 (d, *J* = 7.8 Hz, 1H),

4.59 (d, J = 11.6 Hz, 1H), 4.45 – 4.34 (m, 3H), 4.29 (d, J = 11.8 Hz, 1H), 4.25 – 4.18 (m, 2H), 4.03 – 3.96 (m, 1H), 3.92 – 3.83 (m, 2H), 3.72 (dd, J = 10.7, 3.5 Hz, 1H), 3.62 – 3.50 (m, 1H), 3.45 (dd, J = 9.6, 6.3 Hz, 1H), 3.39 – 3.24 (m, 2H), 3.22 – 3.07 (m, 3H), 2.86 – 2.77 (m, 1H), 2.71 – 2.62 (m, 1H), 2.59 – 2.46 (m, 2H), 2.09 (s, 3H), 1.82 (s, 3H), 1.57 – 1.41 (m, 4H), 1.30 – 1.20 (m, 2H). ^{13}C NMR (101 MHz, $CDCl_3$) δ 207.9, 172.9, 170.3, 166.1, 138.9, 138.2, 138.0, 137.0, 136.8, 135.0, 129.0, 128.8, 128.8, 128.8, 128.7, 128.6, 128.5, 128.4, 128.3, 128.3, 128.0, 127.9, 127.9, 127.8, 127.7, 127.7, 127.4, 127.3, 101.9, 98.1, 78.8, 77.5, 77.4, 77.2, 76.8, 76.5, 76.5, 75.1, 73.5, 72.7, 72.5, 71.0, 70.4, 69.8, 69.4, 68.1, 68.0, 67.6, 67.3, 60.0, 50.5, 50.3, 47.2, 46.1, 38.6, 32.0, 30.4, 29.9, 29.8, 29.8, 29.7, 29.5, 29.4, 29.2, 29.2, 29.1, 28.2, 27.9, 27.6, 23.5, 23.4, 22.8, 20.6, 14.3. HR-ESI-MS (m/z): calculated for $C_{60}H_{68}N_4O_{16}Na$ $[M+Na]^+$: 1123.4528, found: 1123.4534

_N_-(Benzyl)benzyloxycarbonyl-5-aminopentyl 2-azido-2-deoxy-3-_O_-(2-napthylmethyl)-4,6-_O_-silylidene-α-D-galactopyranosyl-(1→3)-4-_O_-acetyl-6-_O_-benzyl-2-_O_-levulinoy-β-D-galactopyranosyl uronate-(1→3)-2-azido-4,6-di-_O_-benzyl-2-deoxy-α-D-galactopyranoside (43)

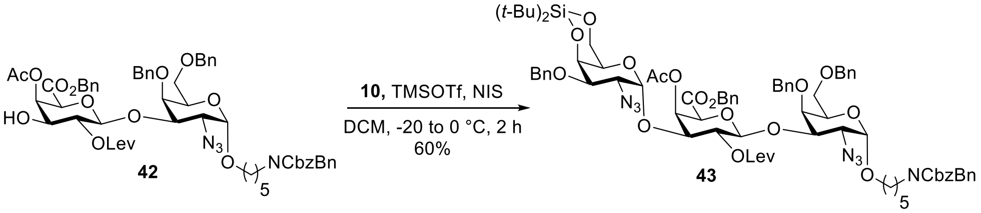

To a solution of acceptor **42** (48 mg, 0.044 mmol) and donor **10** (38 mg, 0.065 mmol) in DCM (5 mL) was added activated 4Å molecular sieves. After ~30 minutes of stirring at rt, the mixture was cooled to 0 °C, to this, NIS (15 mg, 0.065 mmol) and TMSOTf (1.9 µL, 0.022 mmol) were slowly added. After TLC analysis indicated complete consumption of the starting material (~2 hours), the reaction was quenched with Et_3N (2 mL) and the mixture was diluted with DCM. After filtration over Celite® 353, the mixture was washed with saturated aqueous $Na_2S_2O_3$, saturated aqueous $NaHCO_3$ and brine, dried over Na_2SO_4, filtered and concentrated *in vacuo*. The residue was purified by silica column chromatography using 12% ethyl acetate in hexanes to afford the title product **43** (40 mg, 60%) as the sole isomer. ^{1}H NMR (400 MHz, $CDCl_3$) δ 7.39 – 7.31 (m, 4H), 7.30 – 7.16 (m, 26H), 7.10 (d, J = 7.3 Hz, 1H), 5.83 – 5.80 (m, 1H), 5.26 (dd, J = 10.1, 7.8 Hz, 1H), 5.12 – 5.06 (m, 3H), 5.03 (d, J = 8.0 Hz, 2H), 4.99 – 4.92 (m, 2H), 4.84 – 4.80 (m, 1H), 4.75 – 4.66 (m, 2H), 4.58 (dd, J = 11.5, 8.9 Hz, 2H), 4.44 – 4.36 (m, 3H), 4.33 – 4.27 (m, 2H), 4.22 (d, J = 3.4 Hz, 2H), 4.15 (d, J = 12.3 Hz, 1H), 4.01 (dd, J = 10.1, 3.4 Hz, 2H), 3.90 – 3.83 (m, 2H), 3.83 – 3.75 (m, 2H), 3.67 – 3.55 (m, 2H), 3.49 – 3.43 (m, 1H), 3.40 – 3.25 (m, 2H), 3.23 – 3.09 (m, 2H), 2.91 (dd, J = 15.2, 10.7 Hz, 1H), 2.53 – 2.42 (m, 1H), 2.41 – 2.31 (m, 2H), 2.05 (s, 3H), 1.87 (s, 3H), 1.56 – 1.41 (m, 4H), 1.32 – 1.23 (m, 2H), 1.02 (s, 9H), 0.99 (s, 9H). ^{13}C NMR (101 MHz, $CDCl_3$) δ 206.7, 171.1, 169.9, 165.8, 156.8, 156.2, 138.8, 138.4, 138.1, 137.9, 136.9, 136.8, 134.7, 129.1, 129.0, 128.9, 128.8, 128.7, 128.7, 128.6, 128.6, 128.5, 128.5, 128.4, 128.4, 128.3, 128.3, 128.3, 128.0, 127.9, 127.9, 127.8, 127.7, 127.7, 127.6, 127.4, 127.3, 127.2, 102.3, 98.2, 94.2, 78.0, 77.5, 77.4, 77.2, 76.8, 76.3, 75.8, 75.0, 73.4, 72.4, 71.6, 70.4, 70.1, 69.8, 69.4, 69.3, 68.1, 67.9, 67.7, 67.2, 67.1, 65.5, 60.5, 59.8, 57.9, 50.4, 50.2, 47.1, 46.0, 37.3, 32.0, 30.0, 29.8,

29.7, 29.7, 29.4, 29.2, 29.1, 28.0, 28.0, 27.8, 27.8, 27.6, 27.6, 27.5, 27.4, 23.4, 23.3, 22.8, 21.1, 20.7, 20.3, 14.3, 14.2. HR-ESI-MS (m/z): calculated for $C_{81}H_{99}N_7O_{20}SiNa$ $[M+Na]^+$: 1540.6612, found: 1540.6619.

_N_-(Benzyl)benzyloxycarbonyl-5-aminopentyl 2-azido-2-deoxy-3-_O_-(2-napthylmethyl)-α-D-galactopyranosyl-(1→3)-4-_O_-acetyl-6-_O_-benzyl-2-_O_-levulinoy-β-D-galactopyranosyl uronate-(1→3)-2-azido-4,6-di-_O_-benzyl-2-deoxy-α-D-galactopyranoside (44)

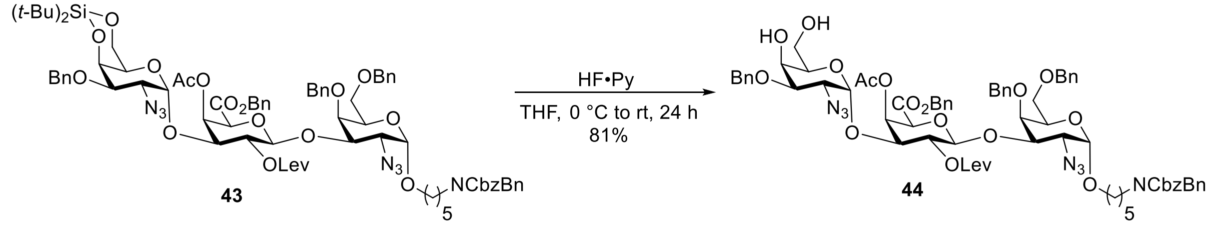

To a solution of trisaccharide **43** (140 mg, 0.092 mmol) in THF (5 mL) was added HF•Py (70% HF, 0.083 mL, 0.922 mmol) at °0 C. After TLC analysis indicated complete conversion of the starting material (~12 hours), the reaction was quenched with Et_3N (2 mL). The mixture was concentrated, dissolved in EtOAc and subsequently washed with saturated aqueous $NaHCO_3$ and brine. The aqueous layers were then extracted with EtOAc combined organic layers were dried over Na_2SO_4, filtered and concentrated *in vacuo*. The residue was purified by silica column chromatography using 50% ethyl acetate in hexanes to furnish the title compound **44** (103 mg, 81%).^{1}H NMR (400 MHz, $CDCl_3$) δ 7.42 – 7.37 (m, 5H), 7.36 (d, *J* = 1.0 Hz, 2H), 7.35 – 7.27 (m, 21H), 7.26 – 7.22 (m, 2H), 7.16 (d, *J* = 6.8 Hz, 1H), 5.86 – 5.83 (m, 1H), 5.32 (dd, *J* = 10.1, 7.8 Hz, 1H), 5.20 – 5.14 (m, 3H), 5.13 – 5.06 (m, 2H), 5.03 (d, *J* = 11.6 Hz, 1H), 4.89 (s, 1H), 4.80 – 4.68 (m, 3H), 4.65 (d, *J* = 11.6 Hz, 1H), 4.54 – 4.45 (m, 4H), 4.36 (d, *J* = 11.8 Hz, 1H), 4.28 (t, *J* = 2.5 Hz, 2H), 4.11 – 4.04 (m, 3H), 4.02 – 3.94 (m, 2H), 3.93 – 3.86 (m, 2H), 3.72 (ddd, *J* = 11.8, 8.3, 3.5 Hz, 2H), 3.65 (s, 1H), 3.52 (dd, *J* = 9.6, 6.3 Hz, 1H), 3.42 (dd, *J* = 9.7, 6.0 Hz, 2H), 3.30 – 3.16 (m, 2H), 3.03 – 2.93 (m, 1H), 2.76 – 2.67 (m, 1H), 2.61 (d, *J* = 14.4 Hz, 2H), 2.54 – 2.42 (m, 2H), 2.16 (s, 3H), 1.93 (s, 3H), 1.63 – 1.47 (m, 4H), 1.35 – 1.30 (m, 2H). ^{13}C NMR (151 MHz, $CDCl_3$) δ 207.3, 171.3, 169.9, 165.9, 156.9, 138.9, 138.2, 138.0, 137.7, 134.8, 129.1, 129.0, 128.8, 128.8, 128.7, 128.7, 128.6, 128.5, 128.3, 128.2, 128.0, 127.9, 127.8, 127.7, 127.7, 127.4, 127.3, 102.3, 98.2, 94.2, 78.1, 77.4, 77.2, 76.9, 76.4, 76.2, 75.0, 73.5, 72.4, 72.1, 70.2, 70.1, 69.8, 69.4, 68.2, 68.0, 67.7, 67.6, 67.3, 65.7, 63.2, 59.9, 58.8, 50.5, 50.3, 47.2, 46.1, 37.4, 32.0, 30.1, 29.8, 29.5, 29.2, 27.9, 27.7, 27.6, 23.5, 23.4, 22.8, 20.4, 14.3. HR-ESI-MS (m/z): calculated for $C_{81}H_{99}N_7O_{20}SiNa$ $[M+Na]^+$: 1400.5591, found: 1400.5599.

5-Aminopentyl 2-acetamido-2-deoxy-α-D-galactopyranosyl-(1→3)-4-_O_-acetyl-β-D-galactopyranosyl uronate-(1→3)-2-acetamido-2-deoxy-α-D-galactopyranoside (4)

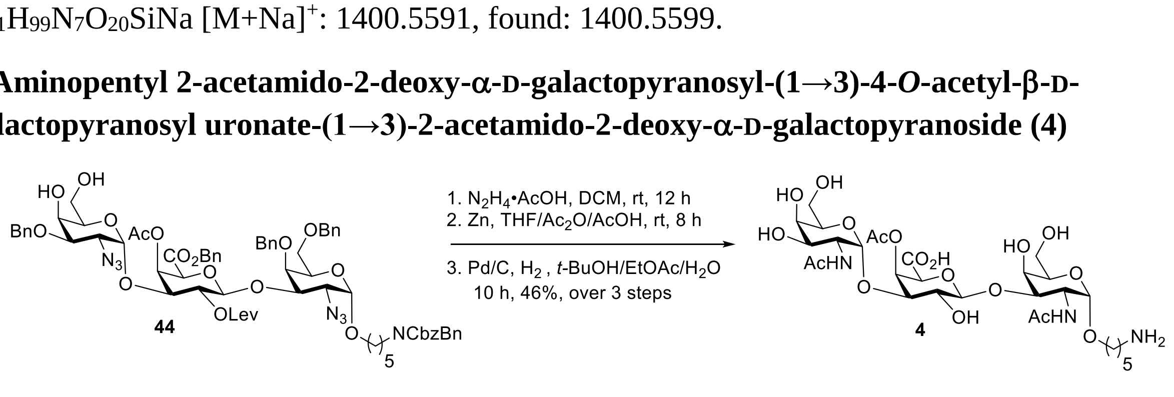

To a solution of trisaccharide **44** (20 mg, 0.014 mmol) in DCM (3 mL) was added N_2H_4•AcOH (8 mg, 0.087 mmol). After stirring for 12 h at rt, the reaction was quenched with acetone (1 mL) and evaporated under high vacuum. Residue was passed through silica column chromatography using ethyl acetate followed by evaporation to get quantitative amount of triol intermediate. The triol intermediate was dissolved in the solution of THF/Ac_2O/AcOH (3/2/1, *v*/*v*/*v*, 3 mL) and added freshly activated zinc (400 mg). After stirring for 8 h at rt, the mixture was diluted with ethyl acetate and filtered over Celite® 353 and evaporated *in vacuo*. The crude was dissolved in EtOAc/*t*-BuOH/H_2O (2/1/1, *v*/*v*/*v*, 2 mL) and Pd/C (50 mg) was added to the solution. After stirring for 10 h under hydrogen atmosphere (1 atm, balloon), the mixture was filtered through a PTFE filter (0.45 µm pore size) and concentrated. The crude material was purified by HPLC (Hypercarb column, 150 x 10 mm, H_2O (0.1% formic acid) isocratic (5 min), linear gradient to 10% ACN (30 min), linear gradient to 100% ACN (10 min)) and lyophilized to obtain pure trisaccharide **4** (4.8 mg, 46%). ^{1}H NMR (700 MHz, D_2O) δ 5.71 (dd, *J* = 3.2, 1.2 Hz, 1H), 5.17 (d, *J* = 3.8 Hz, 1H), 4.92 (d, *J* = 3.8 Hz, 1H), 4.63 (d, *J* = 7.9 Hz, 1H), 4.42 – 4.35 (m, 2H), 4.27 – 4.22 (m, 1H), 4.22 – 4.17 (m, 2H), 4.05 – 4.00 (m, 3H), 3.96 (ddd, *J* = 15.7, 10.5, 3.3 Hz, 2H), 3.82 – 3.77 (m, 4H), 3.77 – 3.72 (m, 1H), 3.69 (dd, *J* = 10.0, 7.9 Hz, 1H), 3.53 (dt, *J* = 9.9, 6.1 Hz, 1H), 3.03 (t, *J* = 7.7 Hz, 2H), 2.16 (s, 3H), 2.06 (s, 3H), 2.04 (s, 3H), 1.75 – 1.64 (m, 4H), 1.52 – 1.44 (m, 1H). ^{13}C NMR (176 MHz, D_2O) δ 174.5, 174.4, 173.5, 172.4, 103.9, 97.2, 93.5, 78.1, 74.1, 73.5, 70.8, 70.7, 69.0, 68.5, 68.4, 68.1, 67.7, 67.4, 61.4, 61.2, 49.3, 48.7, 39.4, 28.1, 26.5, 22.4, 22.2, 22.0, 20.3. HR-ESI-MS (m/z): calculated for $C_{29}H_{50}N_3O_{18}$ $[M+H]^+$: 728.3089, found: 728.3096

N-(Benzyl)benzyloxycarbonyl-5-aminopentyl 4-O-acetyl-β-D-galactopyranosyl uronate-(1→3)-2-acetamido-α-D-galactopyranoside (5)

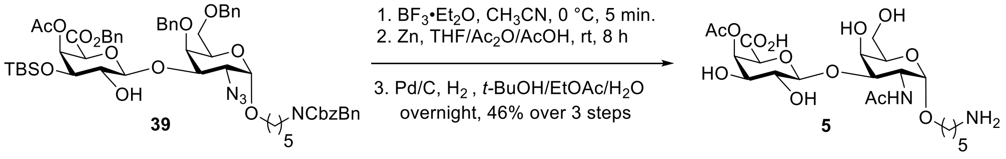

Disaccharide **39** (36 mg, 0.032 mmol) was dissolved in anhydrous ACN (5 mL) under a nitrogen atmosphere at 0 °C and to this, BF_3•OEt_2 (4.8 µL, 0.038 mmol) was added dropwise. After stirring for 5 min, quenched with saturated aqueous $NaHCO_3$, extracted the organic layer with DCM. The combined organic layer was dried over Na_2SO_4, filtered and evaporated *in vacuo*. The crude was dissolved in a mixture of THF/Ac_2O/AcOH (3/2/1, *v*/*v*/*v*, 3 mL) and added freshly activated Zn dust. After stirring overnight at rt, the mixture was diluted with DCM and filtered over Celite® 353. The filtrate was neutralized with saturated aqueous $NaHCO_3$ and washed with brine. The organic phase was dried over Na_2SO_4, filtered and evaporated *in vacuo*. The crude was dissolved in EtOAc/*t*-BuOH/H_2O (2/1/1, *v*/*v*/*v*, 3 mL) and added Pd/C (60 mg). The reaction mixture was stirred overnight at rt under hydrogen atmosphere (1 atm, balloon). The crude material was purified by HPLC (Hypercarb column, 150 x 10 mm, H_2O (0.1% formic acid) isocratic (5 min), linear gradient to 10% ACN (30 min), linear gradient to 100% ACN (10 min) and lyophilized to obtain pure disaccharide **5** (11 mg, 46%) as a white solid. ^{1}H NMR (400 MHz, D_2O) δ 5.54 (dt, *J* = 3.8, 1.0 Hz, 1H), 4.86 (d, *J* = 3.8 Hz, 1H), 4.51 (d, *J* = 7.8 Hz, 1H), 4.36 – 4.28 (m, 2H), 4.16 (d, *J* = 1.4 Hz, 1H), 4.03 – 3.94 (m, 2H), 3.85 (dd, *J* = 10.0, 3.7 Hz, 1H), 3.77 – 3.73 (m, 2H), 3.71 – 3.64

(m, 1H), 3.56 – 3.44 (m, 2H), 2.98 (t, *J* = 7.6 Hz, 2H), 2.09 (d, *J* = 0.7 Hz, 3H), 2.01 – 1.98 (m, 3H), 1.65 (dt, *J* = 15.1, 7.4 Hz, 4H), 1.50 – 1.39 (m, 2H). ^{13}C NMR (101 MHz, D_2O) δ 174.5, 173.5, 173.0, 103.9, 97.1, 77.1, 73.5, 72.1, 71.1, 70.7, 70.2, 68.5, 67.6, 61.3, 48.7, 39.3, 28.0, 26.4, 22.3, 21.9, 20.2. HR-ESI-MS (m/z): calculated for $C_{21}H_{37}N_2O_{13}$ $[M+H]^+$: 525.2296, found: 525.2288

^{1}H NMR (400 MHz, $CDCl_3$)

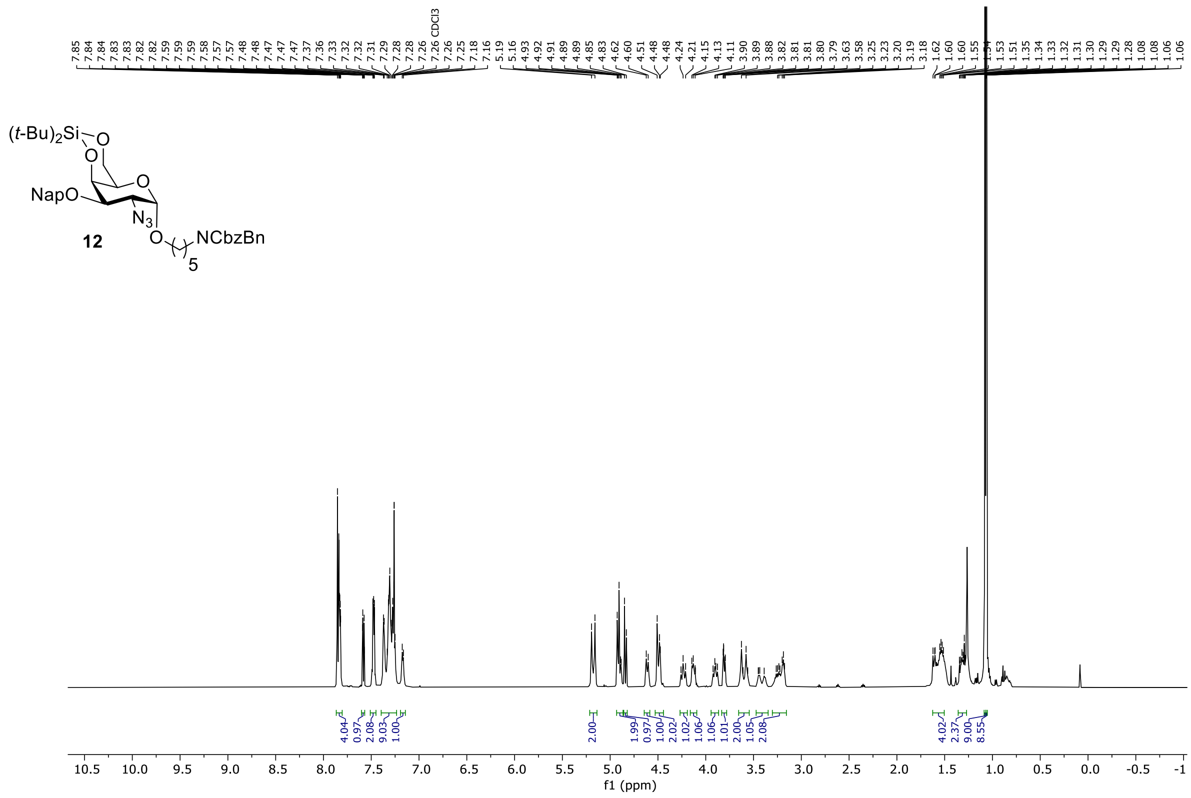

^{13}C NMR (101 MHz, $CDCl_3$)

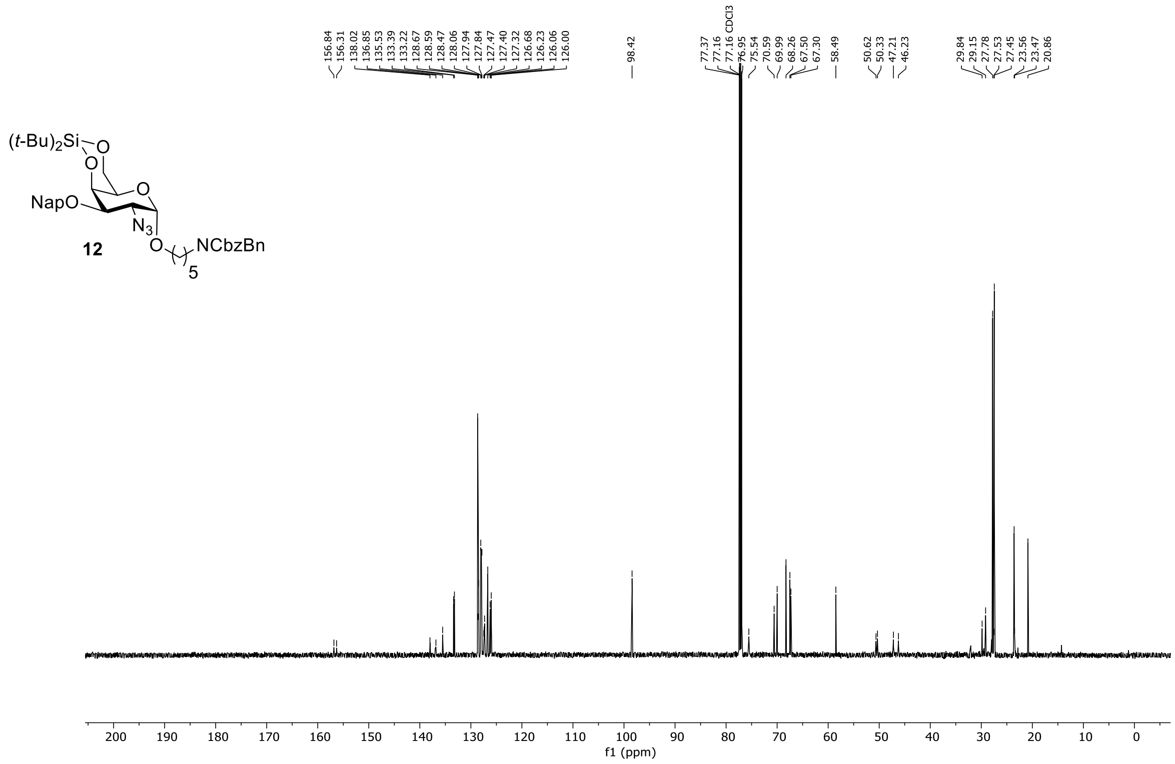

^{1}H-^{1}H COSY NMR (400 MHz, $CDCl_3$)

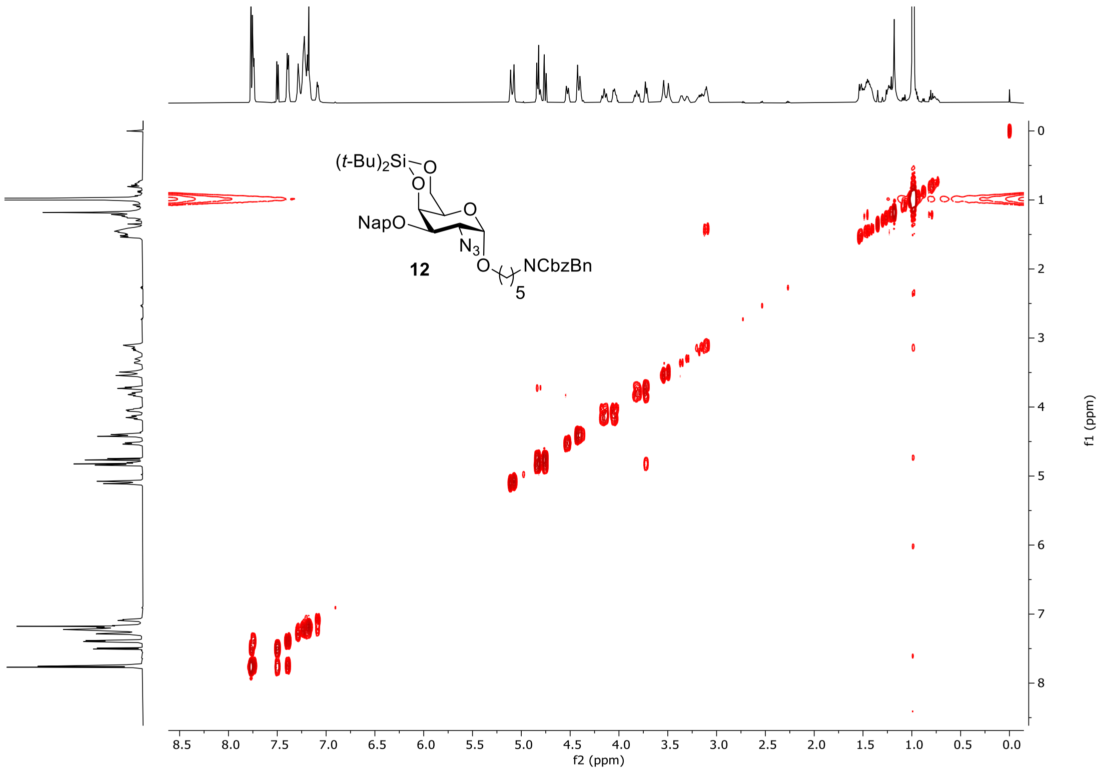

^{1}H-^{13}C HSQC NMR (400 MHz, $CDCl_3$)

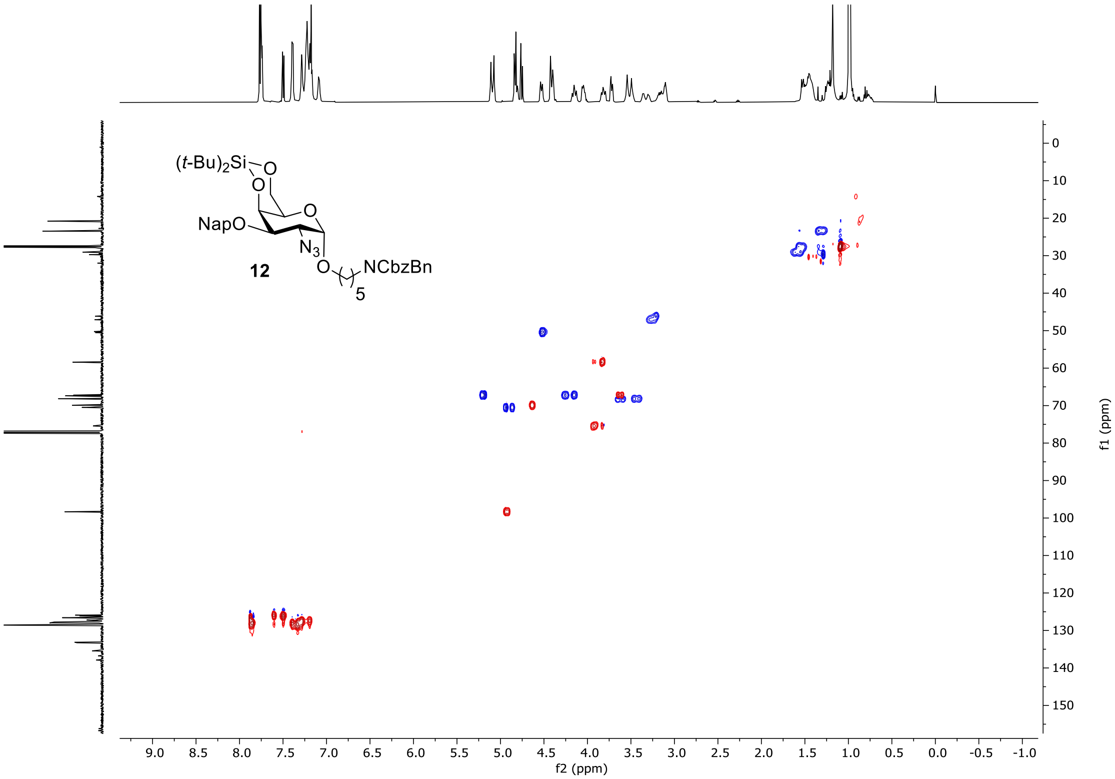

^{1}H-^{13}C Coupled HSQC NMR (400 MHz, $CDCl_3$)

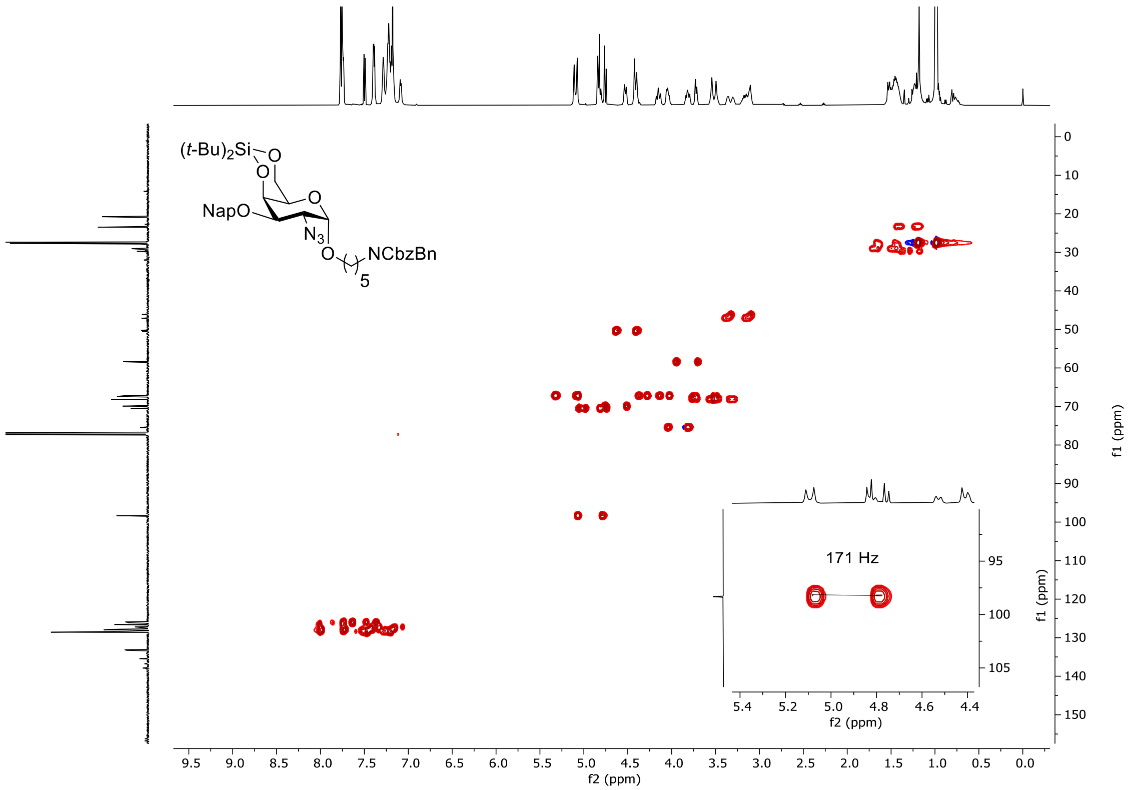

^{1}H NMR (400 MHz, $CDCl_3$)

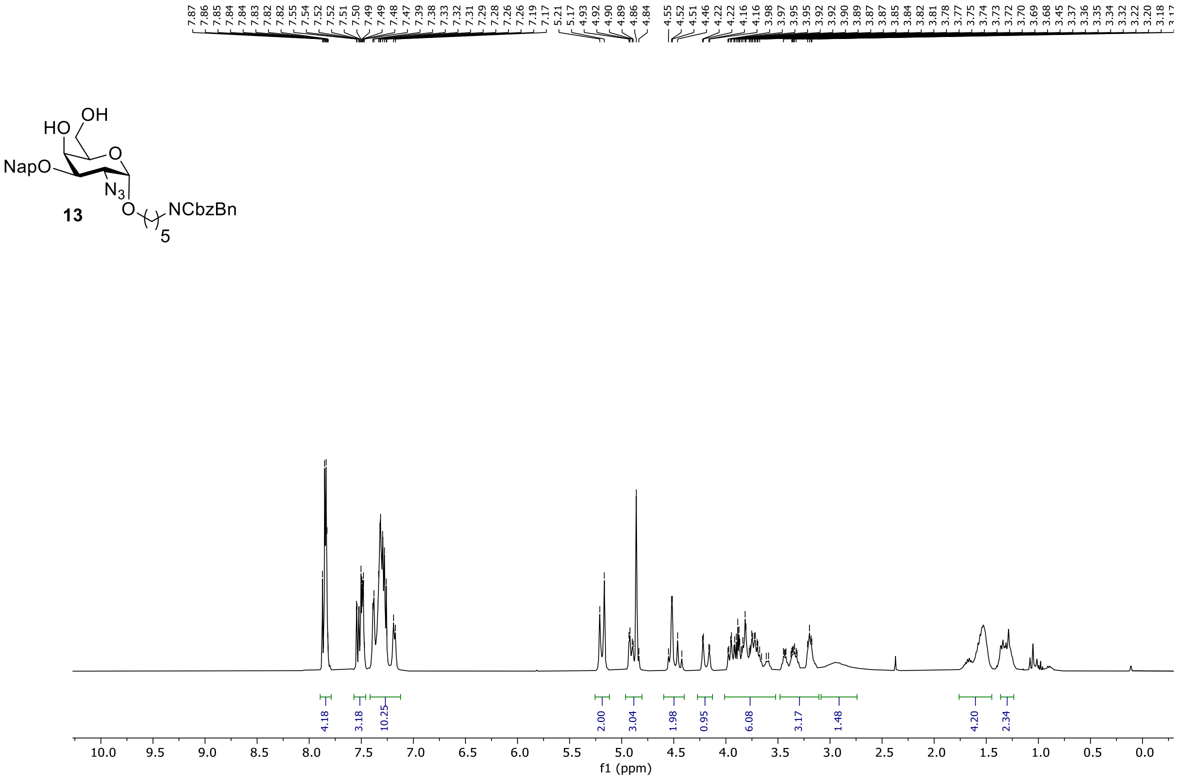

^{13}C NMR (101 MHz, $CDCl_3$)

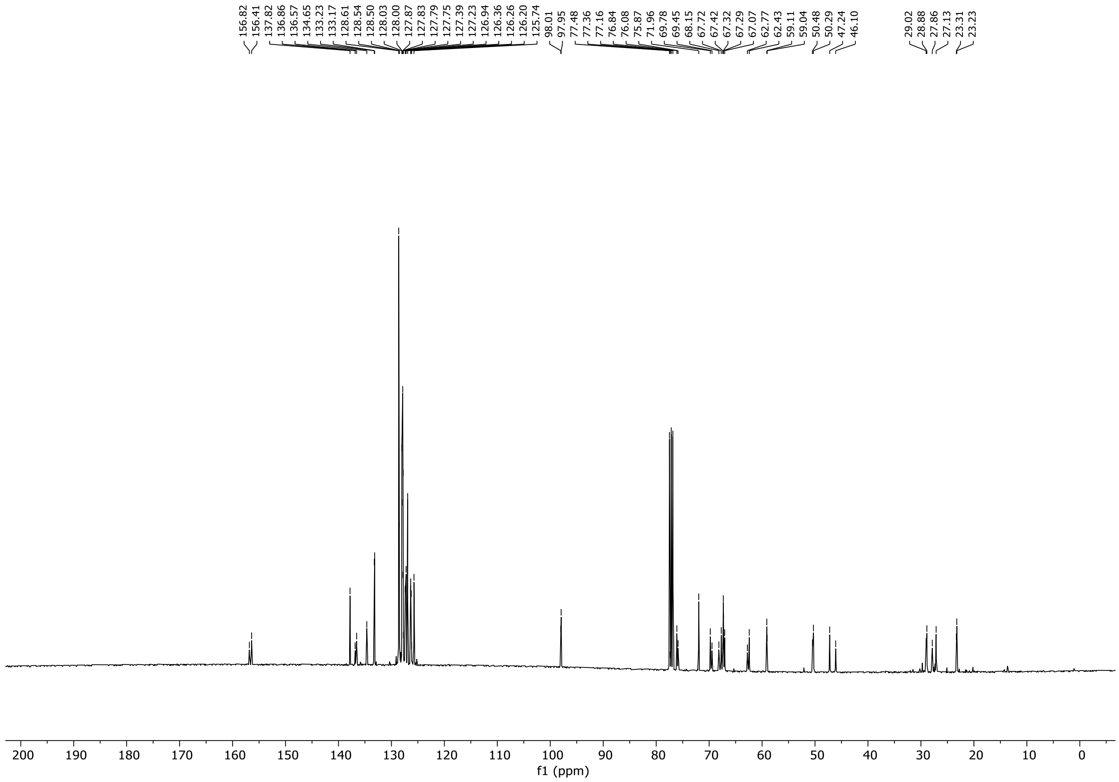

^{1}H-^{1}H COSY NMR (400 MHz, $CDCl_3$)

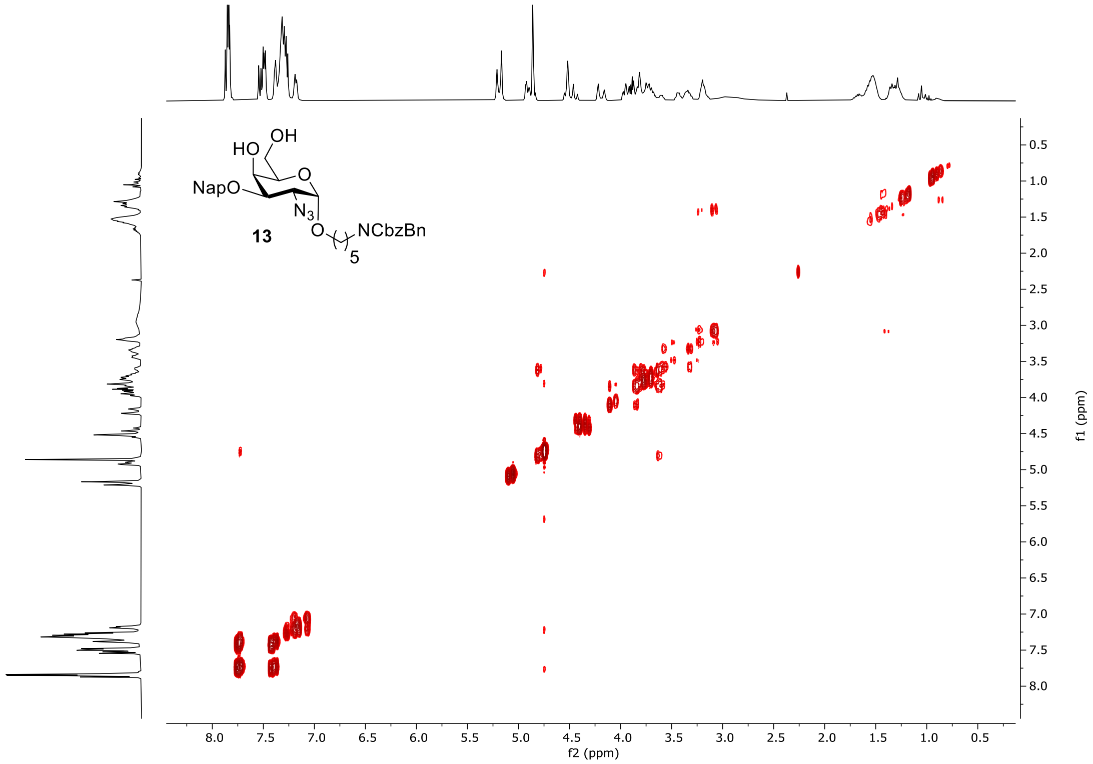

^{1}H-^{13}C HSQC NMR (400 MHz, $CDCl_3$)

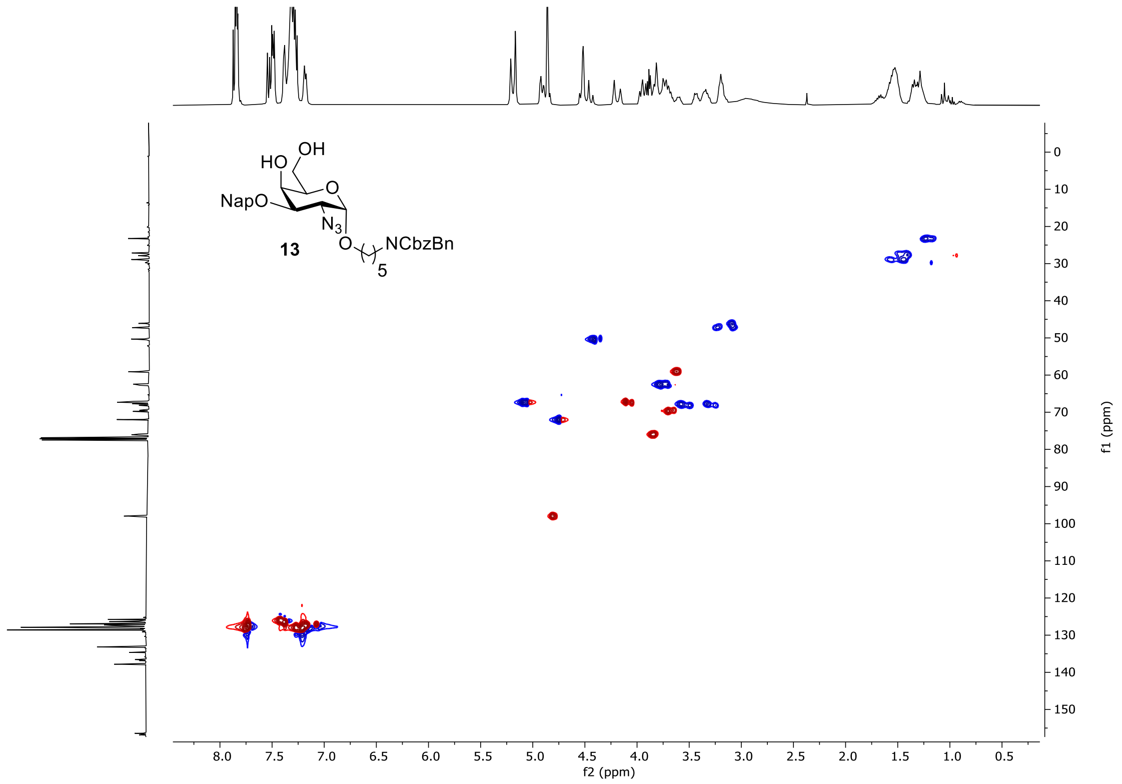

^{1}H-^{13}C Coupled HSQC NMR (400 MHz, $CDCl_3$)

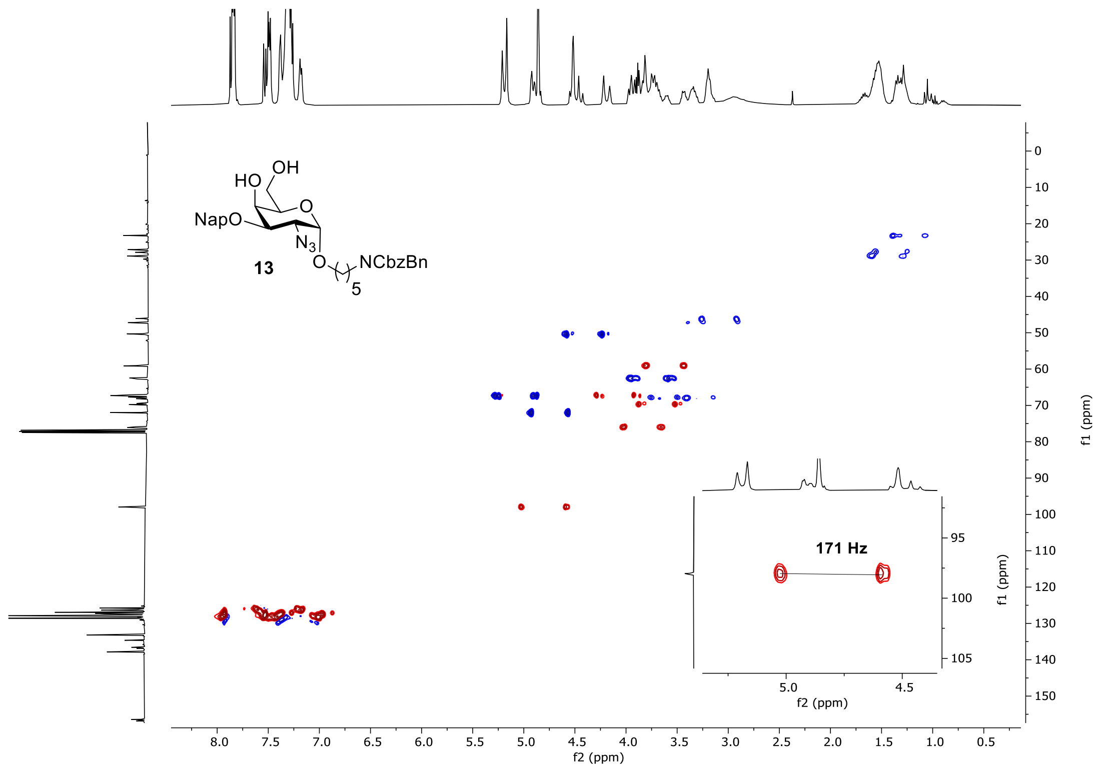

^{1}H NMR (400 MHz, $CDCl_3$)

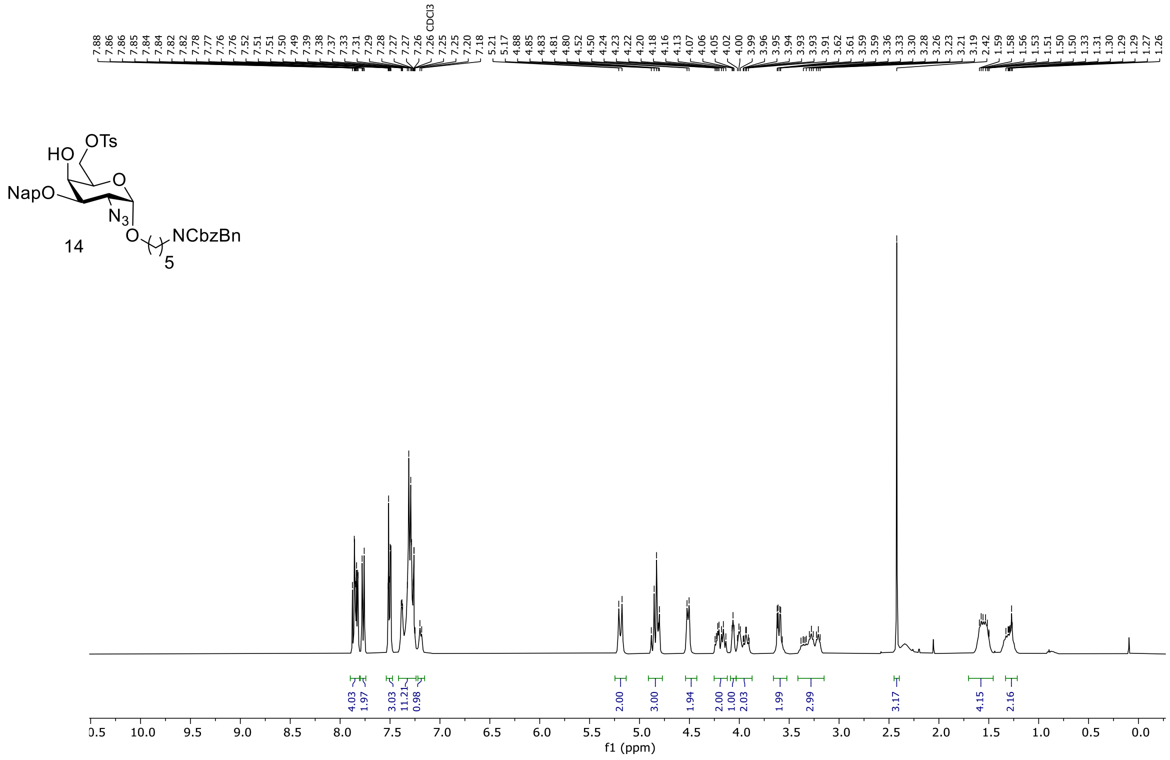

^{13}C NMR (101 MHz, $CDCl_3$)

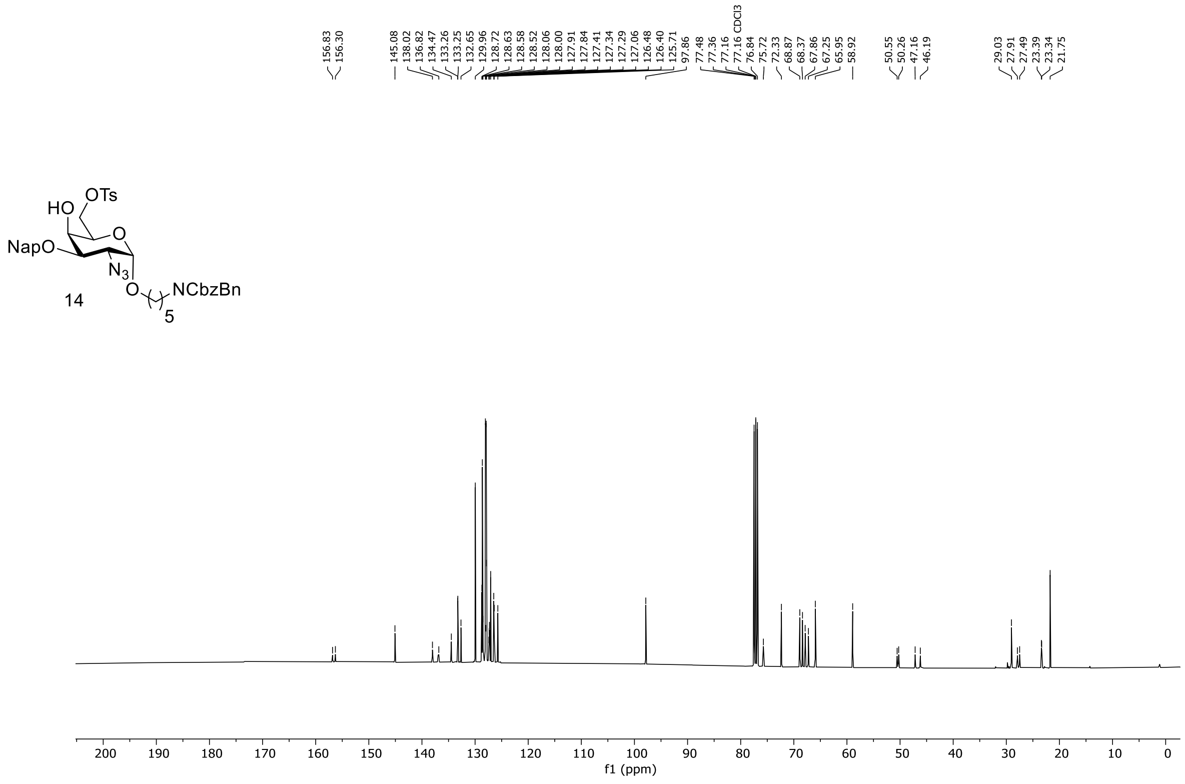

^{1}H-^{1}H COSY NMR (400 MHz, $CDCl_3$)

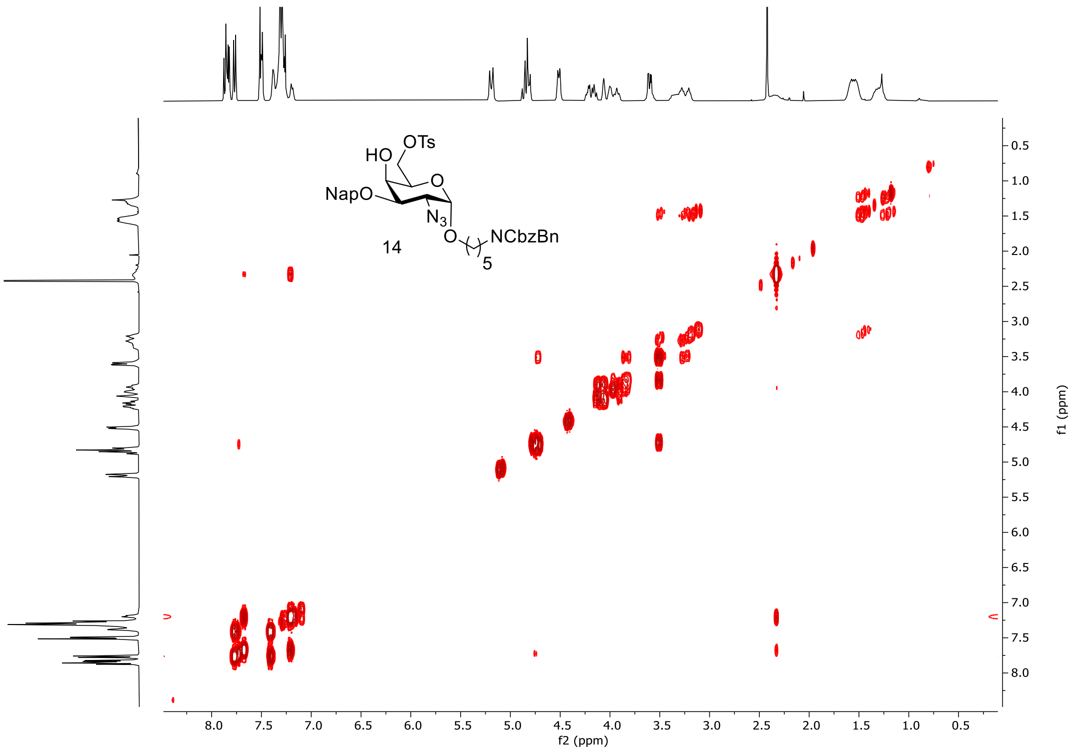

^{1}H-^{13}C HSQC NMR (400 MHz, $CDCl_3$)

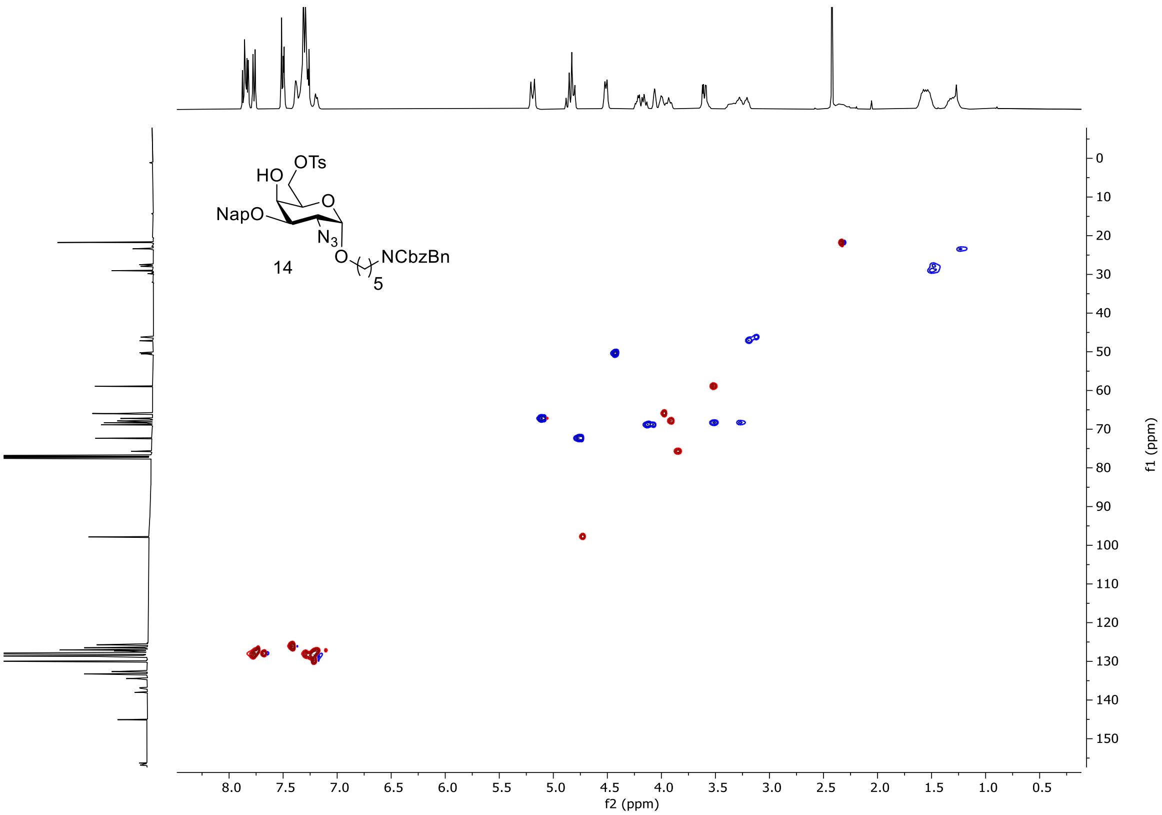

^{1}H-^{13}C Coupled HSQC NMR (400 MHz, $CDCl_3$)

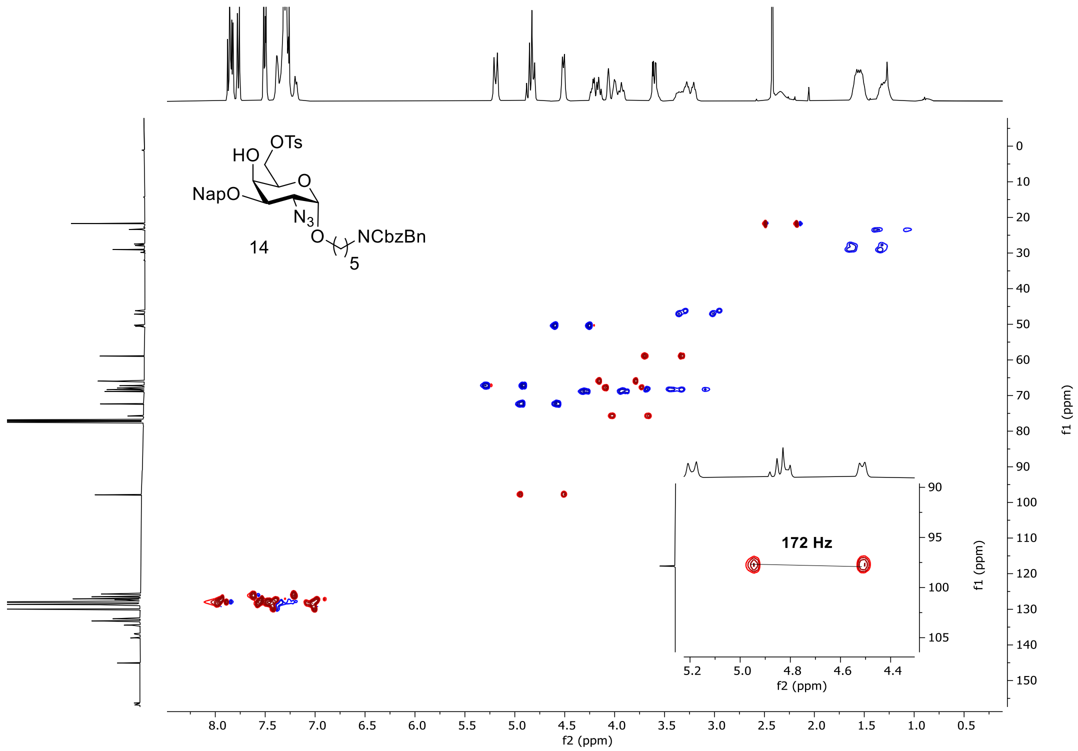

^{1}H NMR (400 MHz, $CDCl_3$)

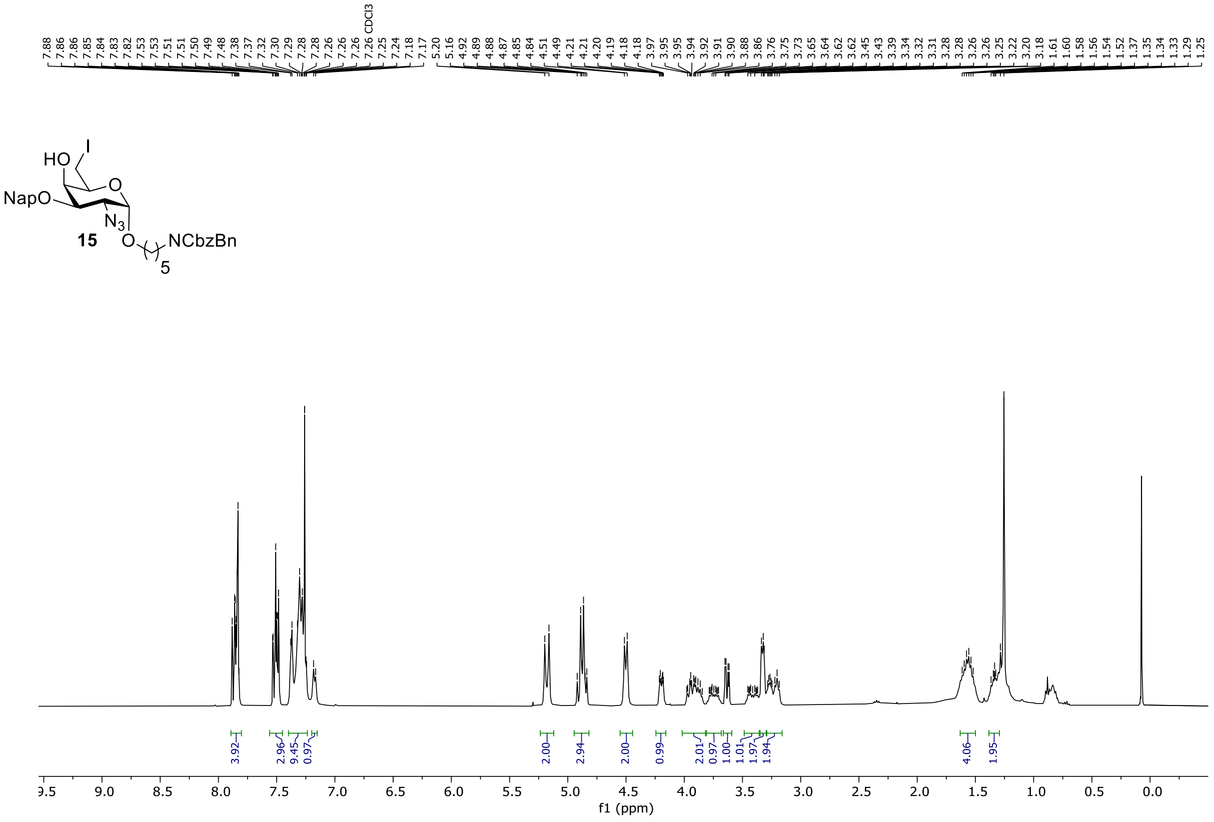

^{13}C NMR (101 MHz, $CDCl_3$)

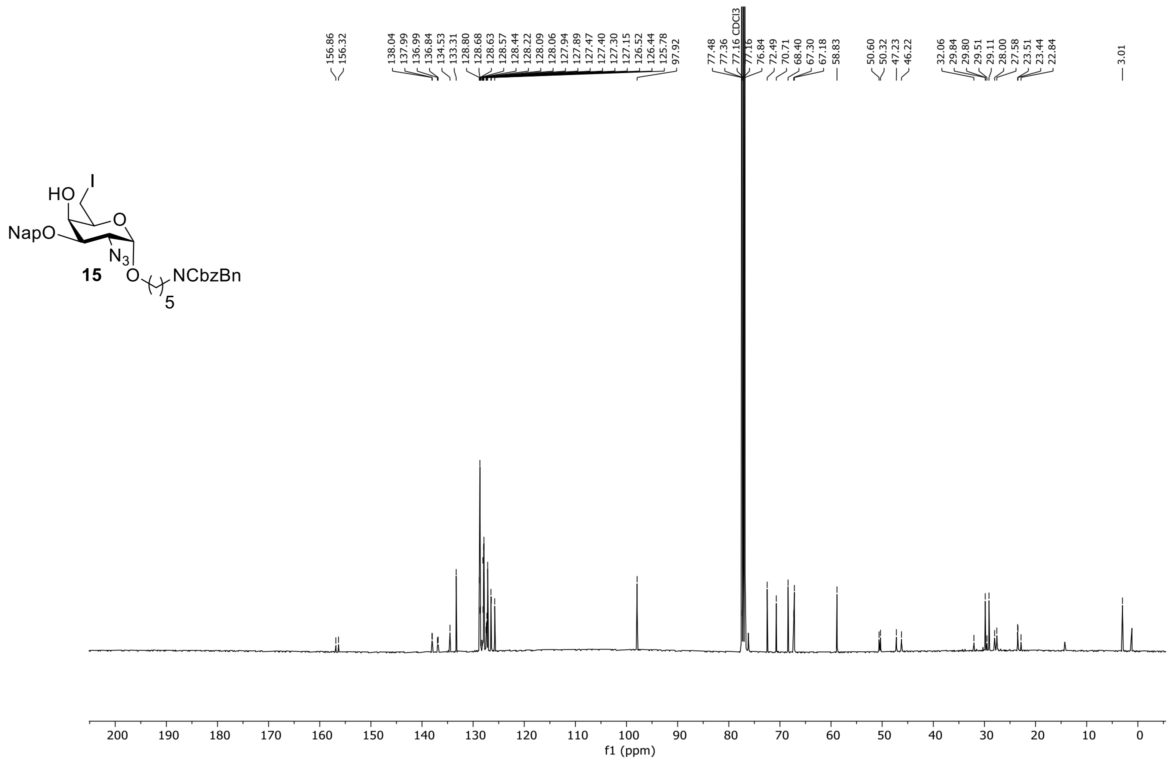

^1H-^1H COSY NMR (400 MHz, $CDCl_3$)

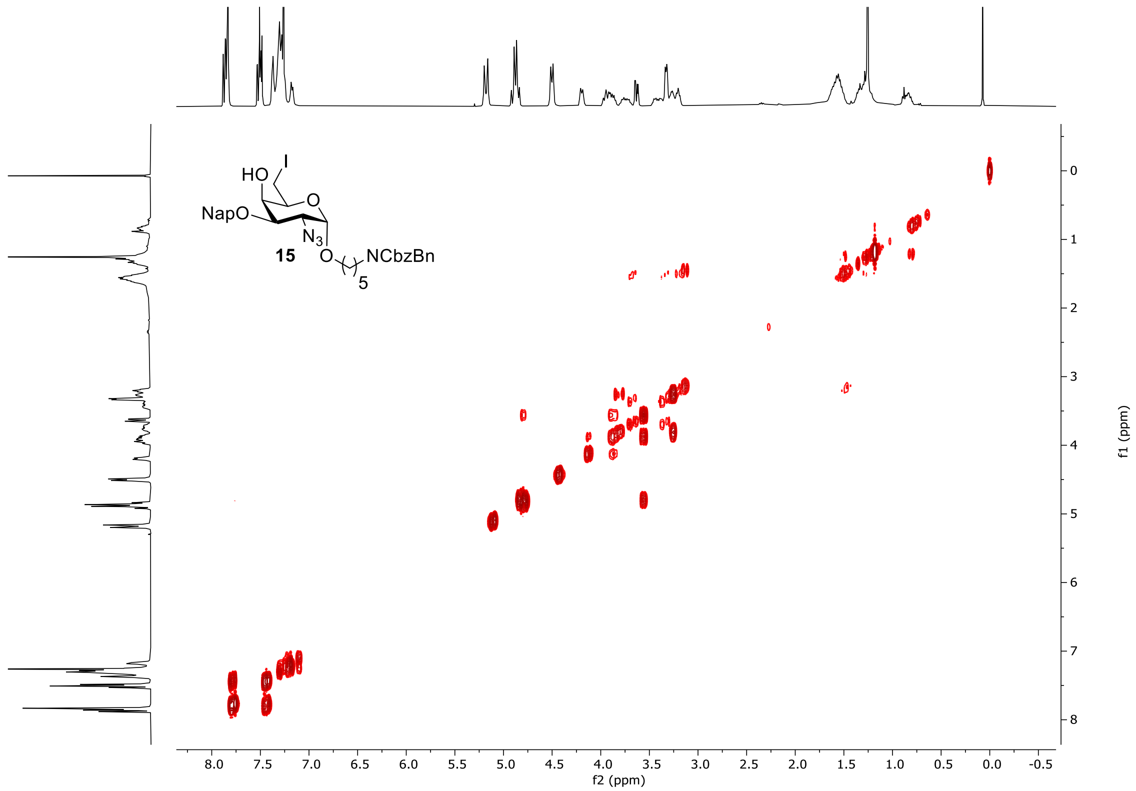

^{1}H-^{13}C HSQC NMR (400 MHz, $CDCl_3$)

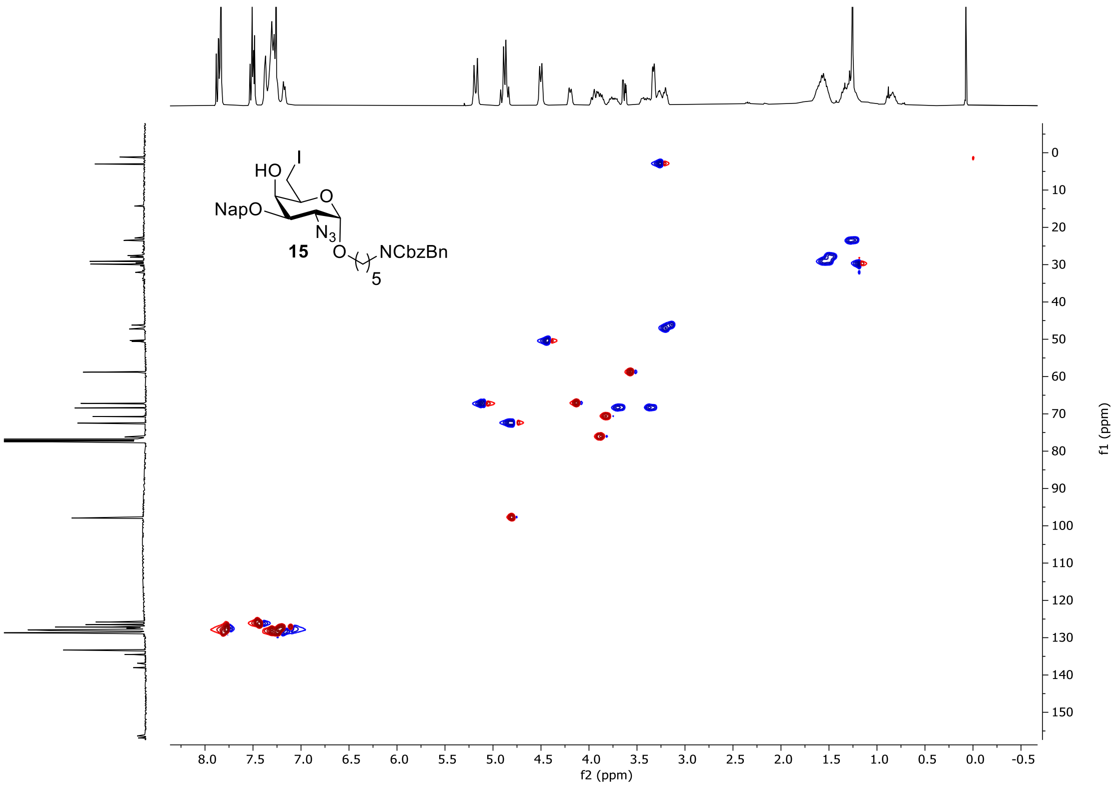

^{1}H-^{13}C Coupled HSQC NMR (400 MHz, $CDCl_3$)

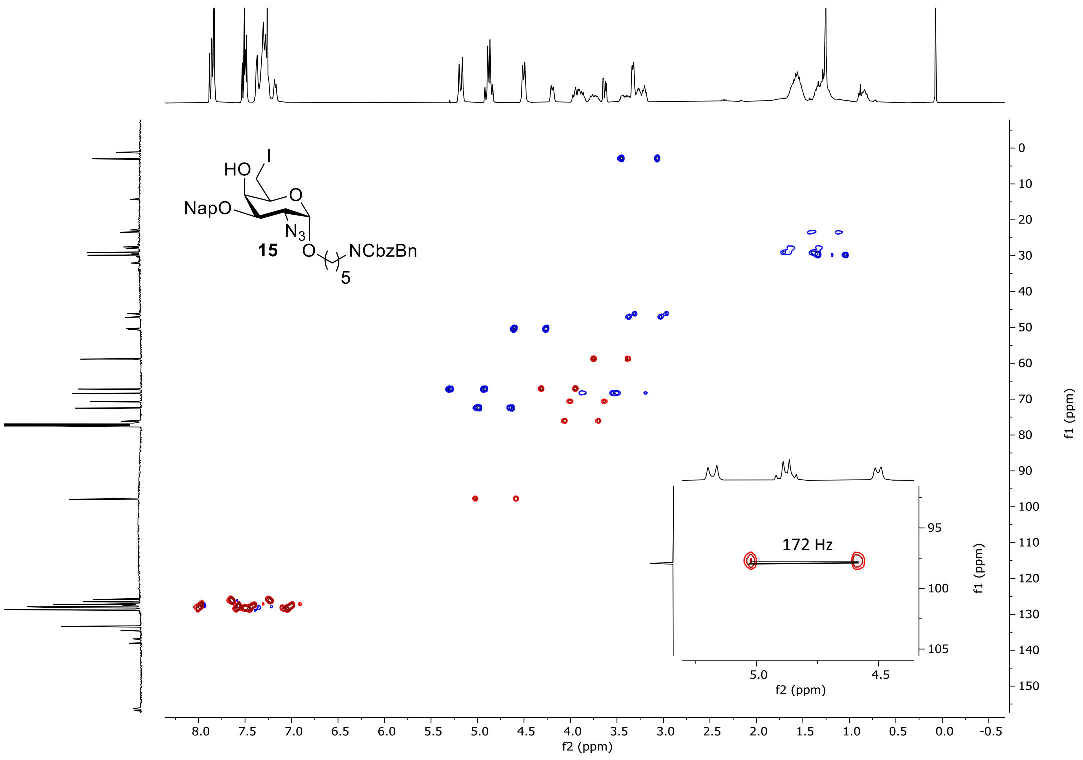

^{1}H NMR (400 MHz, CDCl$_3$)

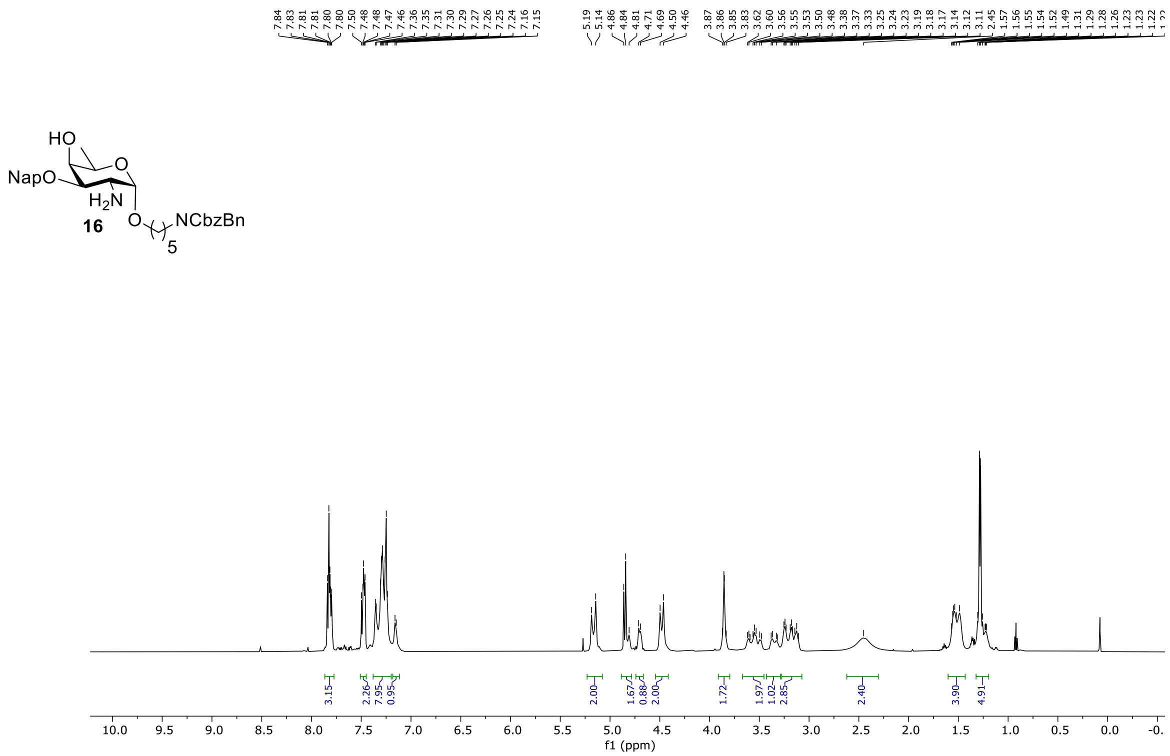

^{13}C NMR (101 MHz, CDCl$_3$)

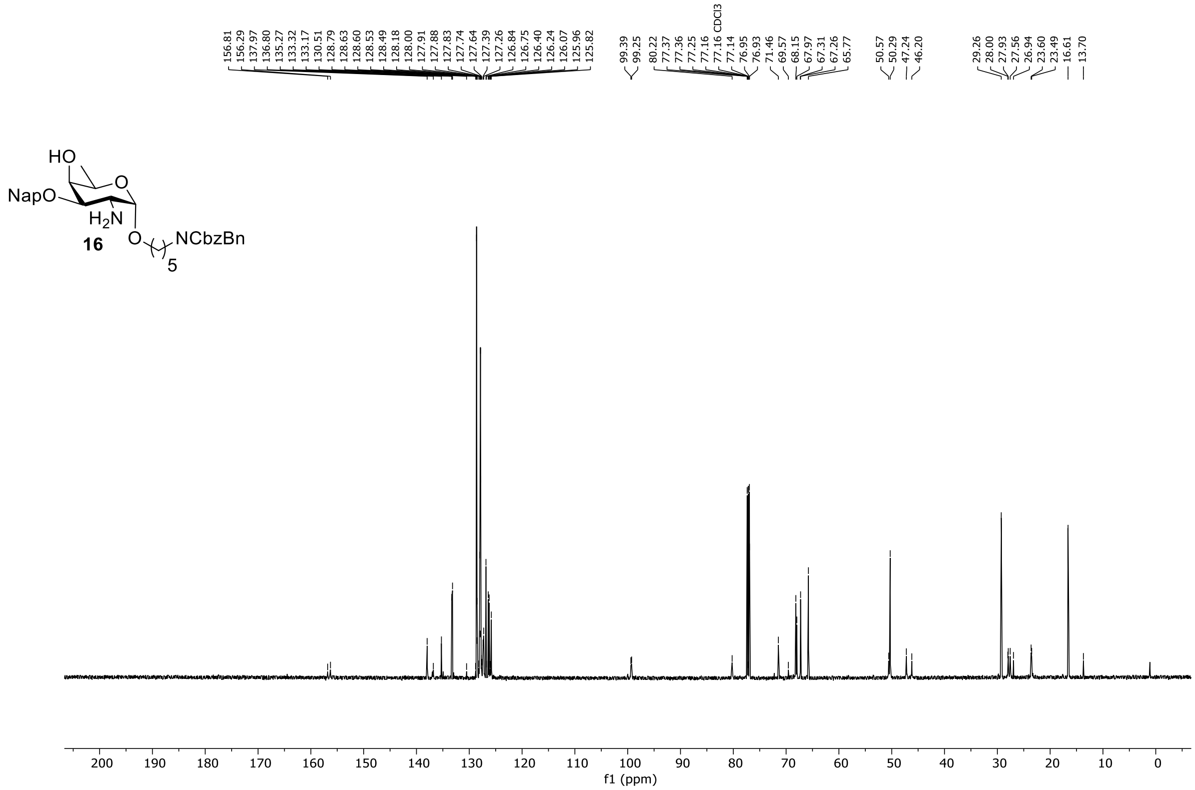

^{1}H-^{1}H COSY NMR (400 MHz, $CDCl_3$)

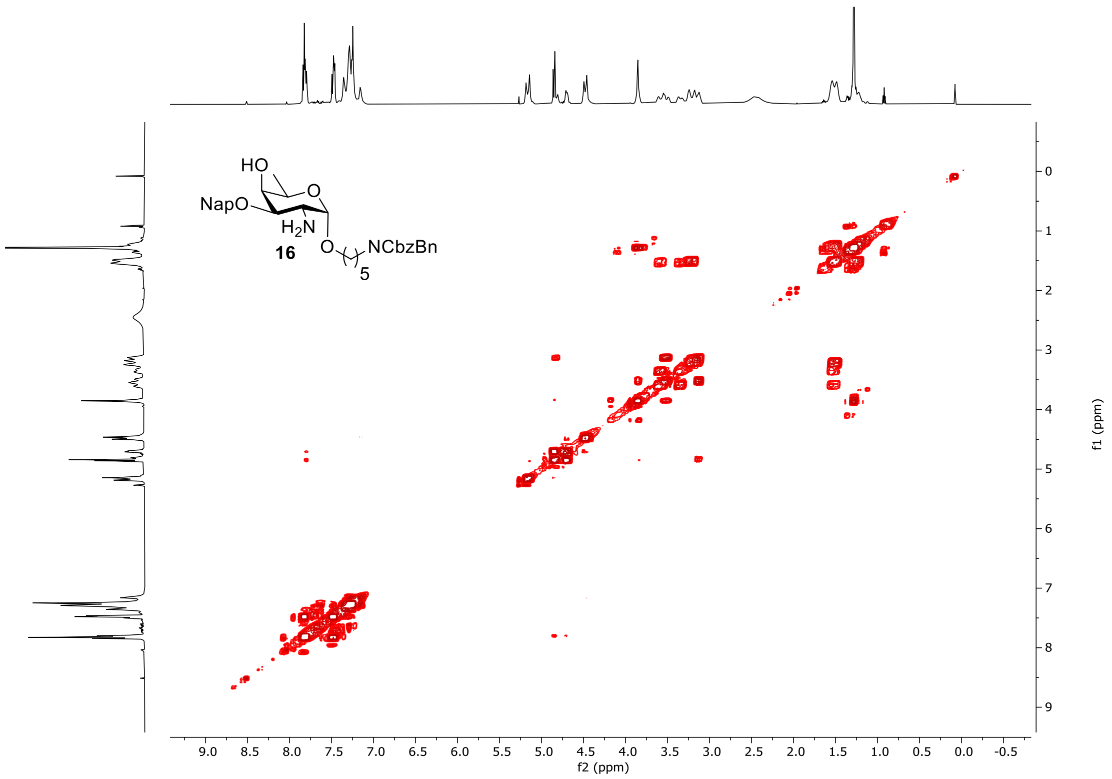

^{1}H-^{13}C HSQC NMR (400 MHz, $CDCl_3$)

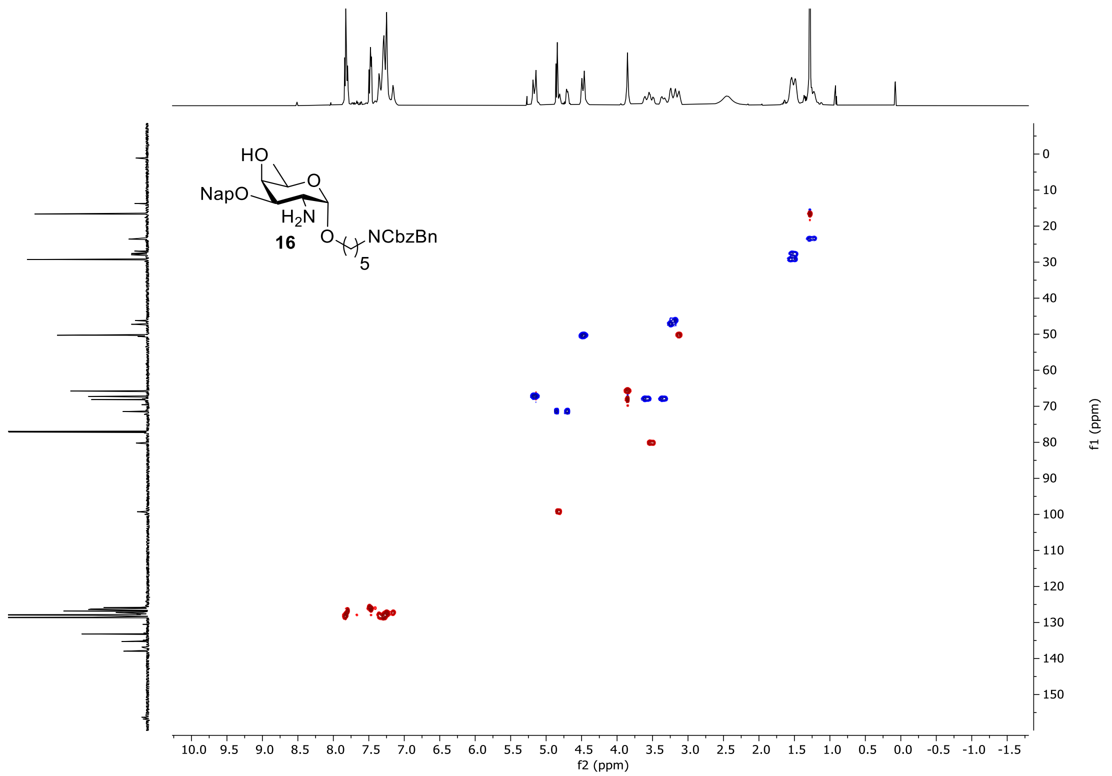

^{1}H-^{13}C Coupled HSQC NMR (400 MHz, $CDCl_3$)

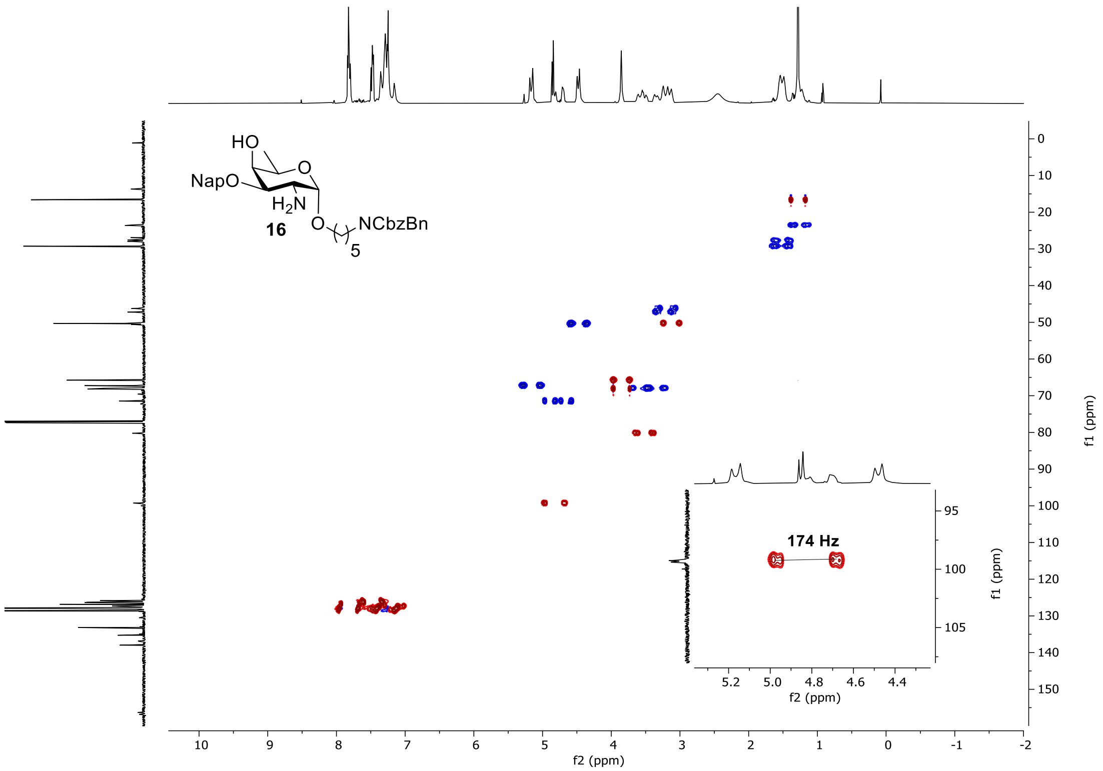

^{1}H NMR (400 MHz, $CDCl_3$)

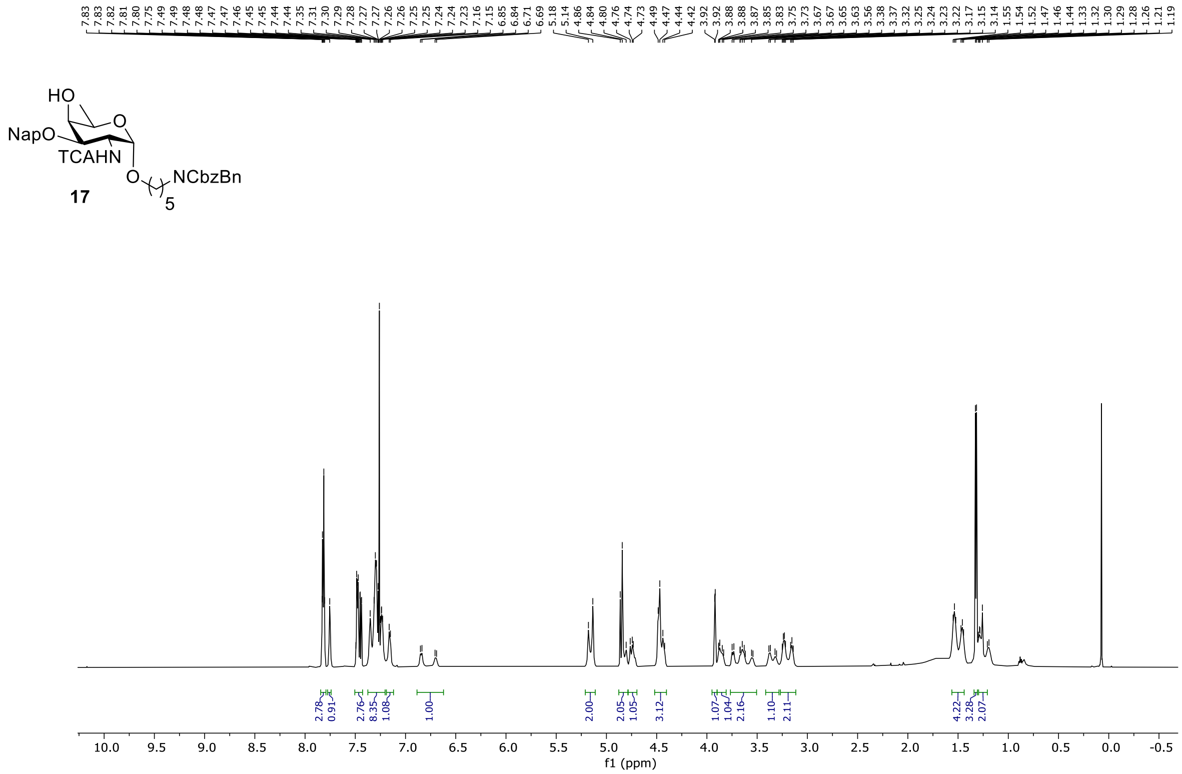

^{13}C NMR (101 MHz, $CDCl_3$)

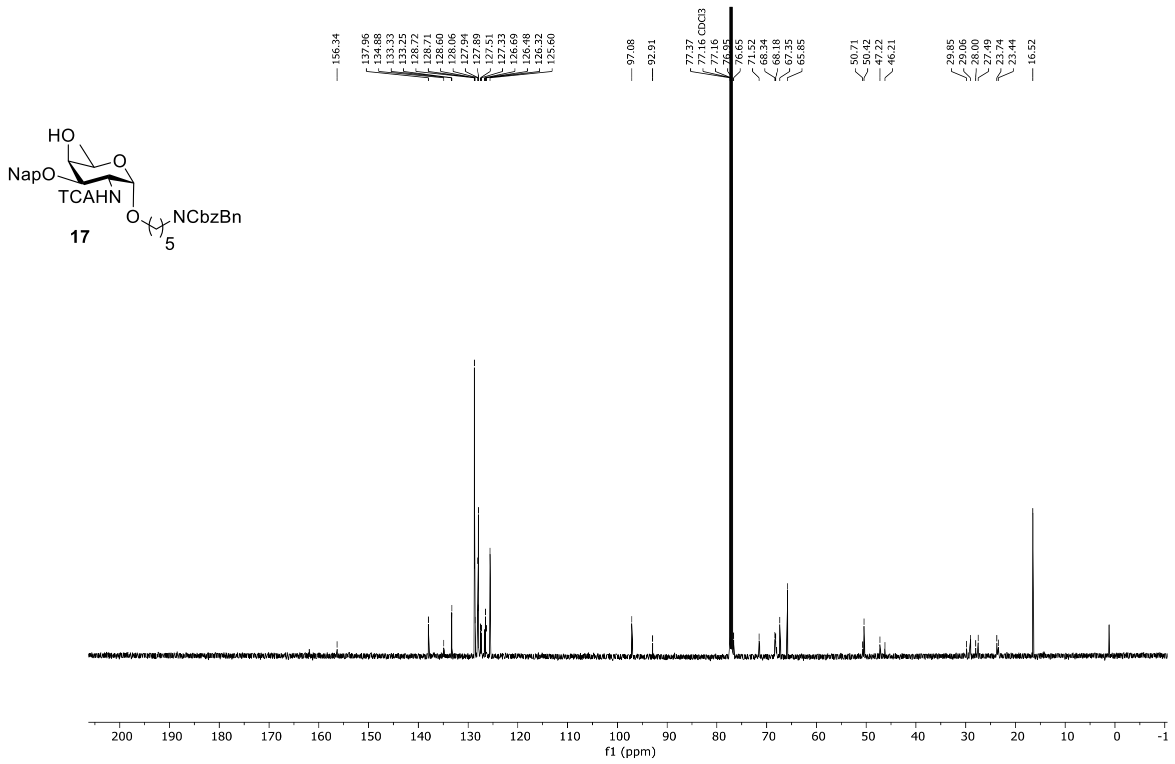

^{1}H-^{1}H COSY NMR (400 MHz, $CDCl_3$)

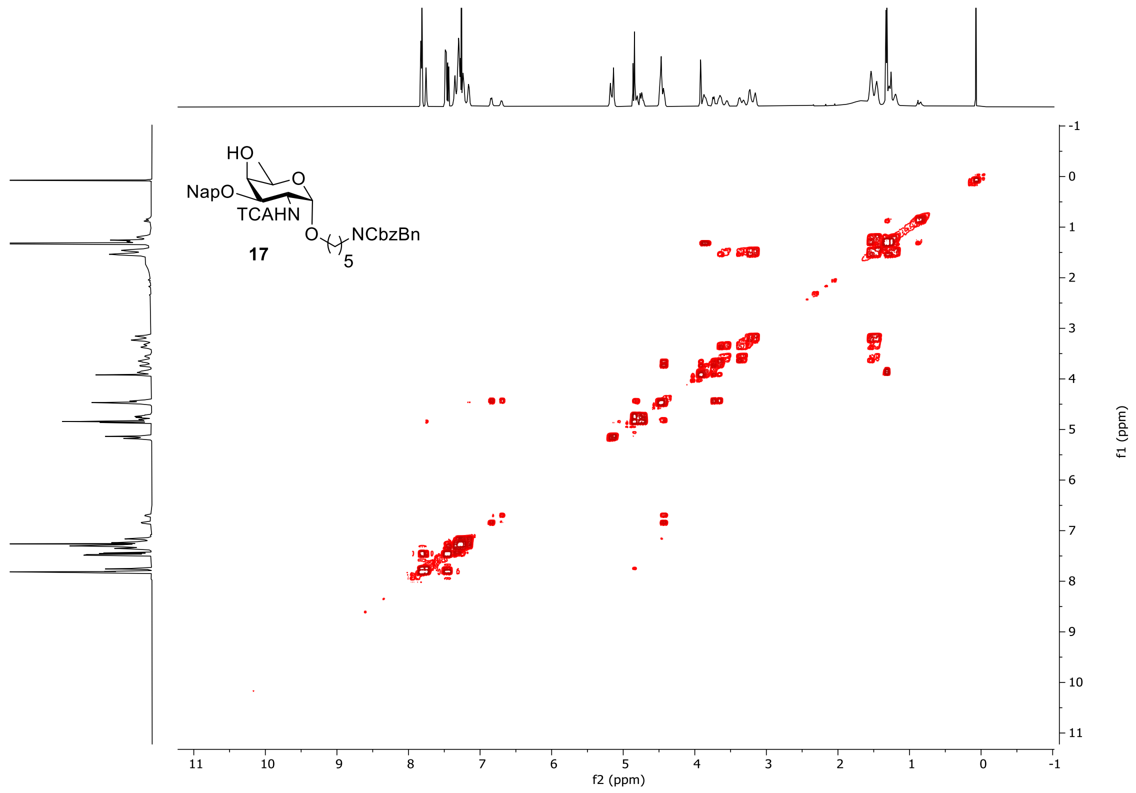

^{1}H-^{13}C HSQC NMR (400 MHz, $CDCl_3$)

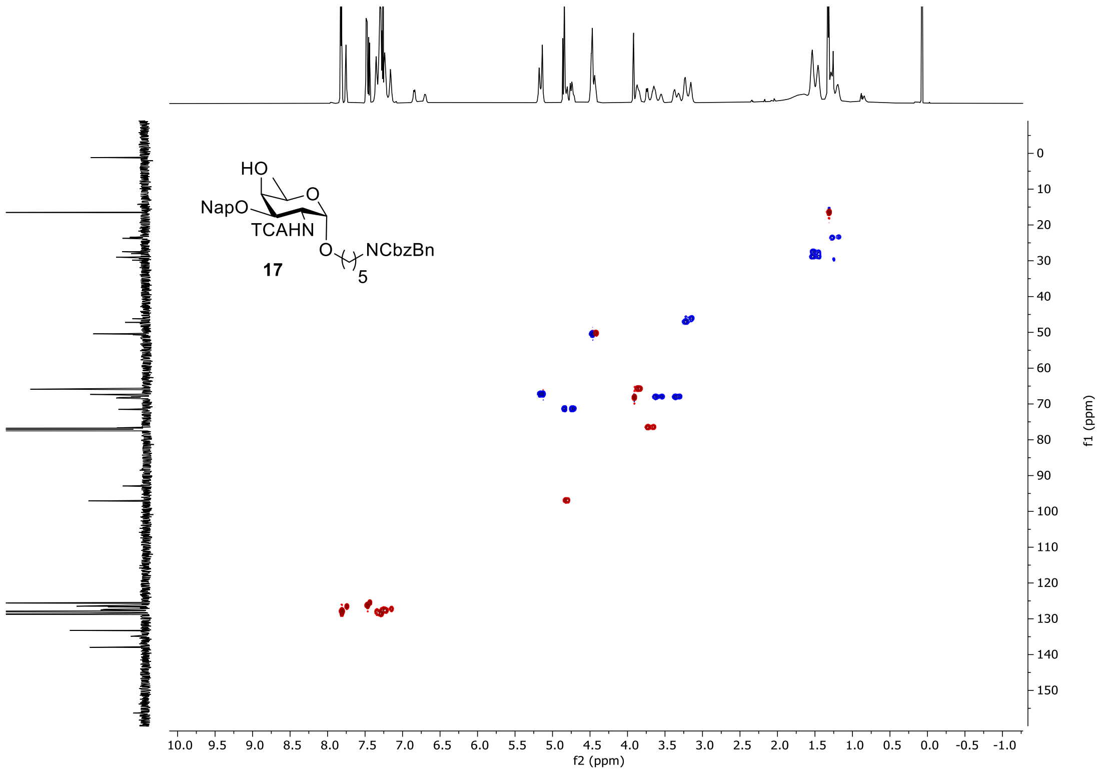

^{1}H-^{13}C Coupled HSQC NMR (400 MHz, $CDCl_3$)

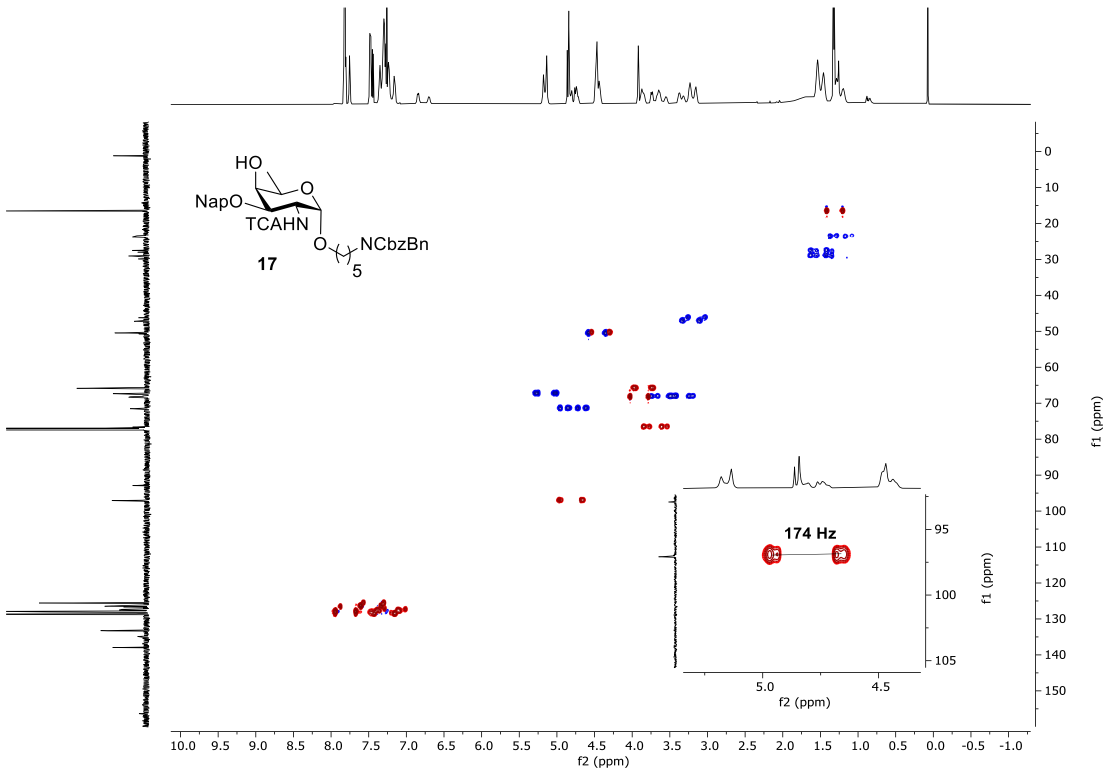

^{1}H NMR (400 MHz, $CDCl_3$)

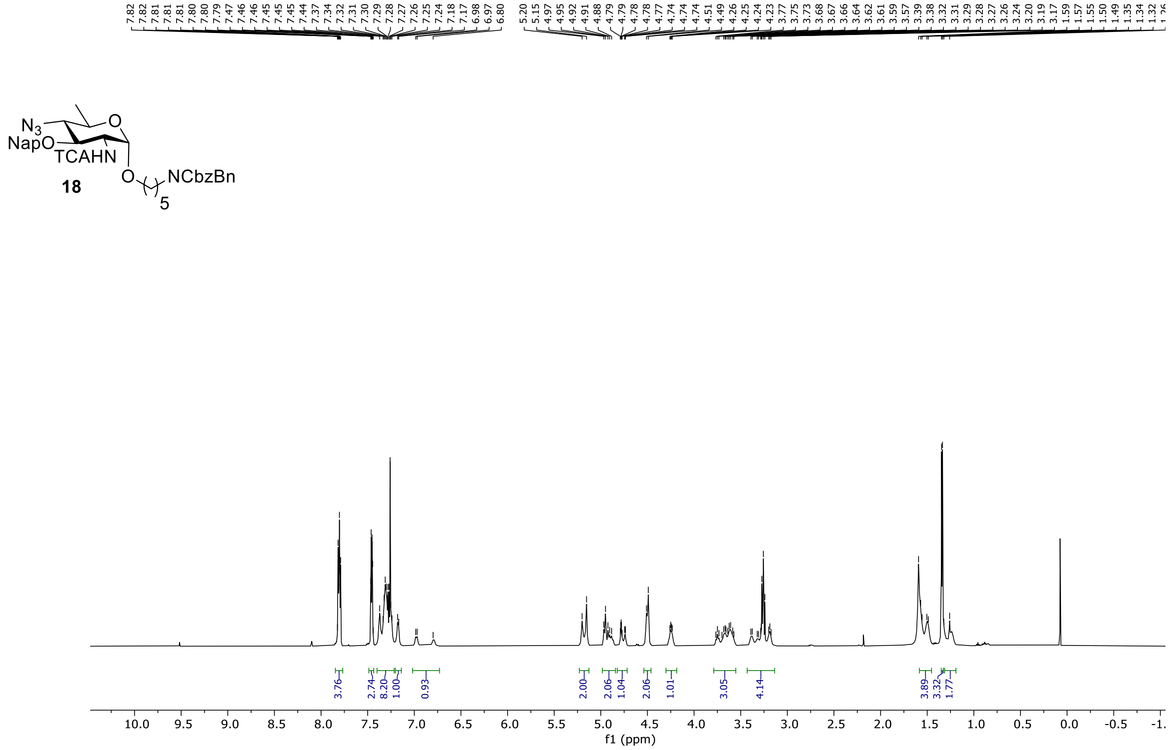

^{13}C NMR (101 MHz, $CDCl_3$)

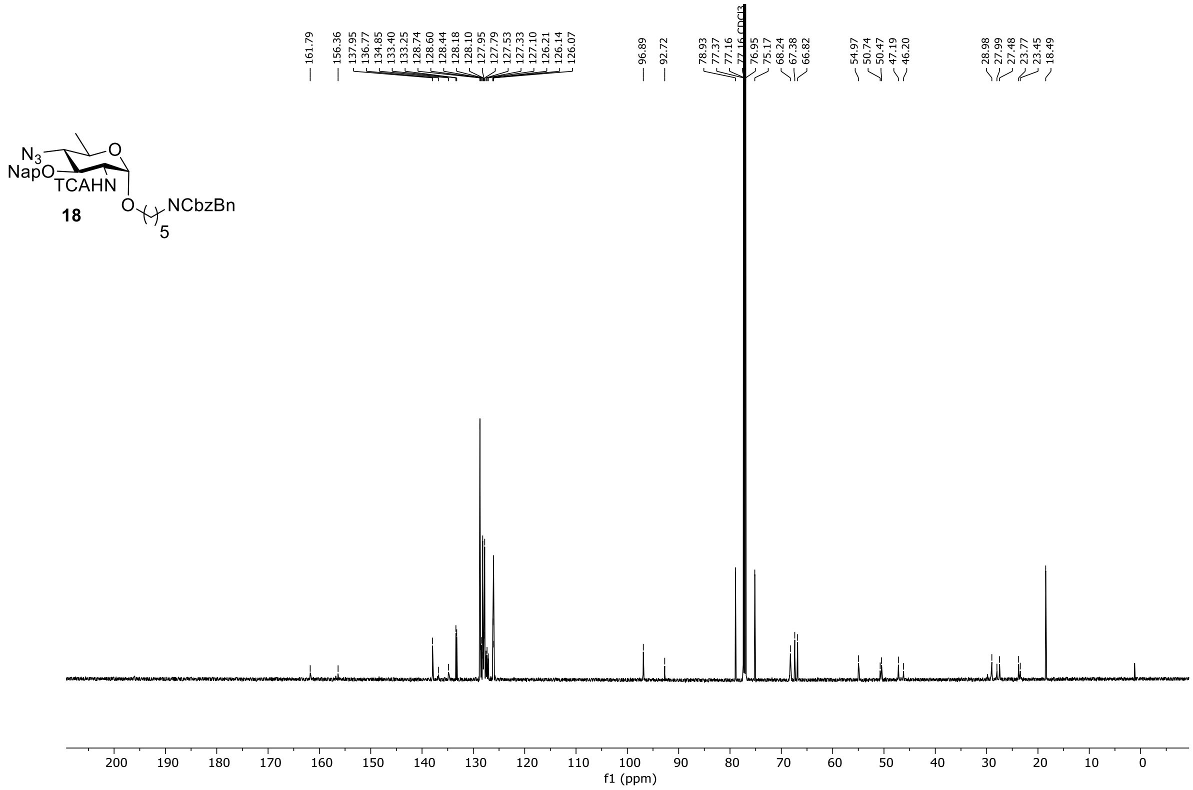

^{1}H-^{1}H COSY NMR (400 MHz, $CDCl_3$)

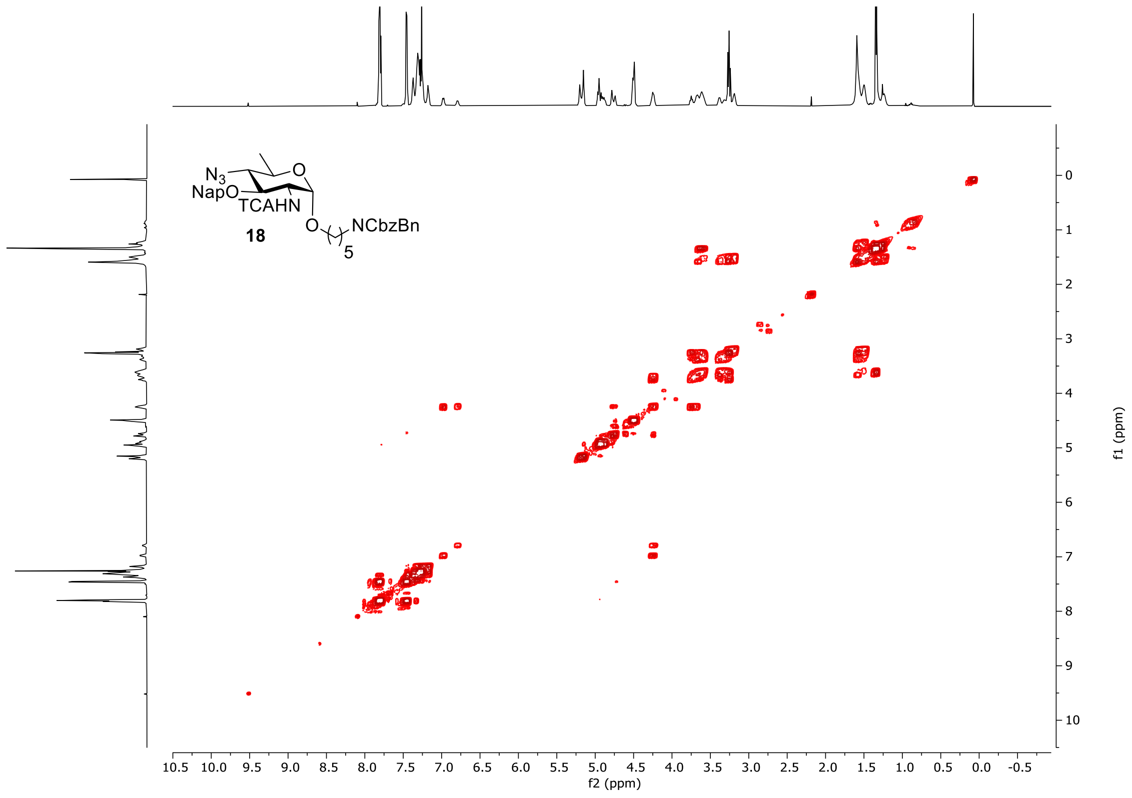

^{1}H-^{13}C HSQC NMR (400 MHz, $CDCl_3$)

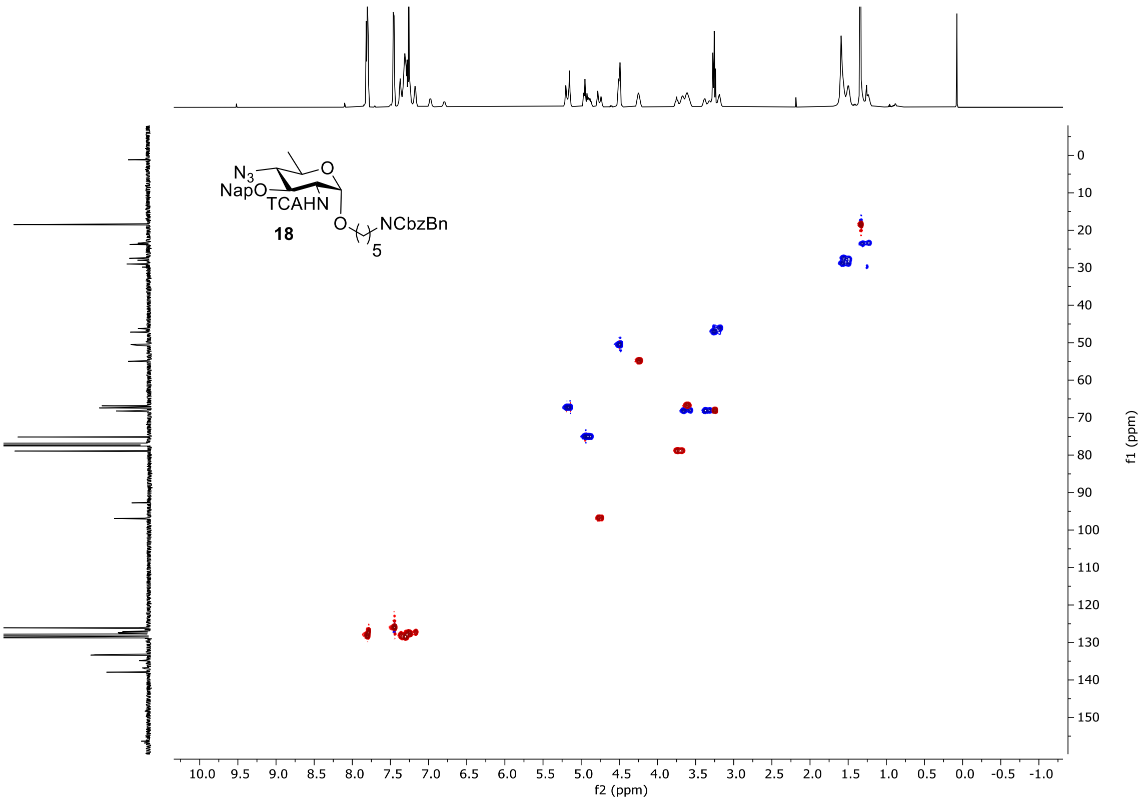

^{1}H-^{13}C Coupled HSQC NMR (400 MHz, $CDCl_3$)

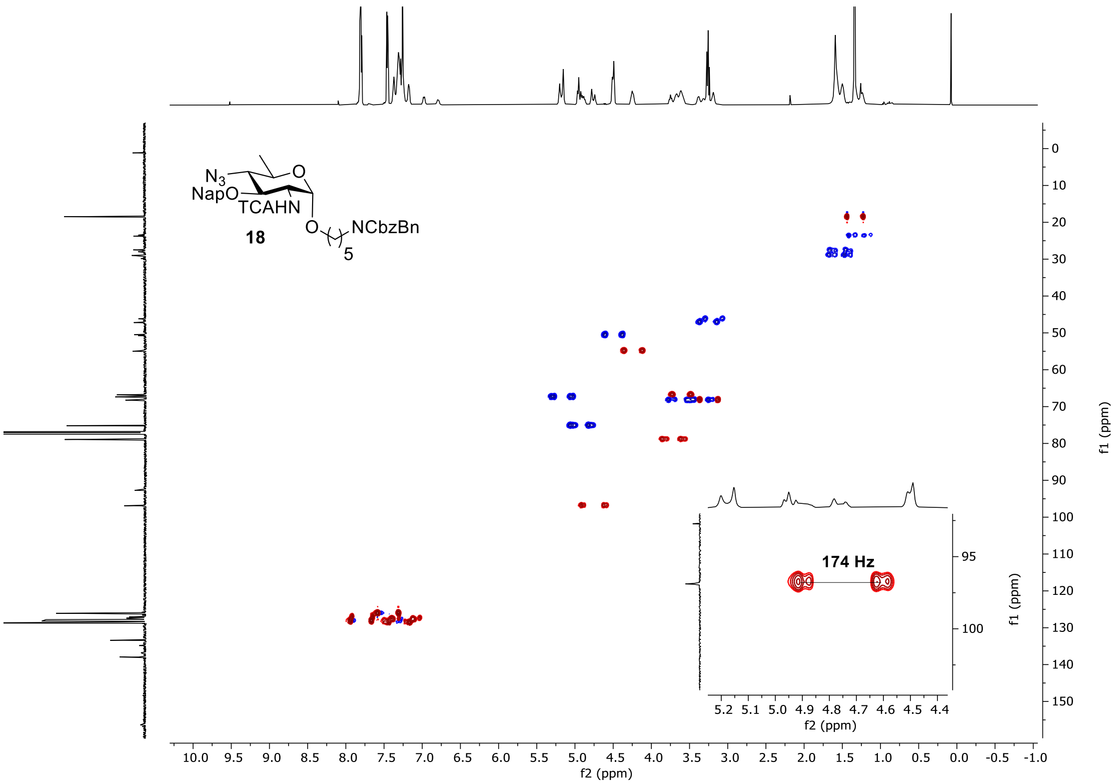

^{1}H NMR (400 MHz, $CDCl_3$)

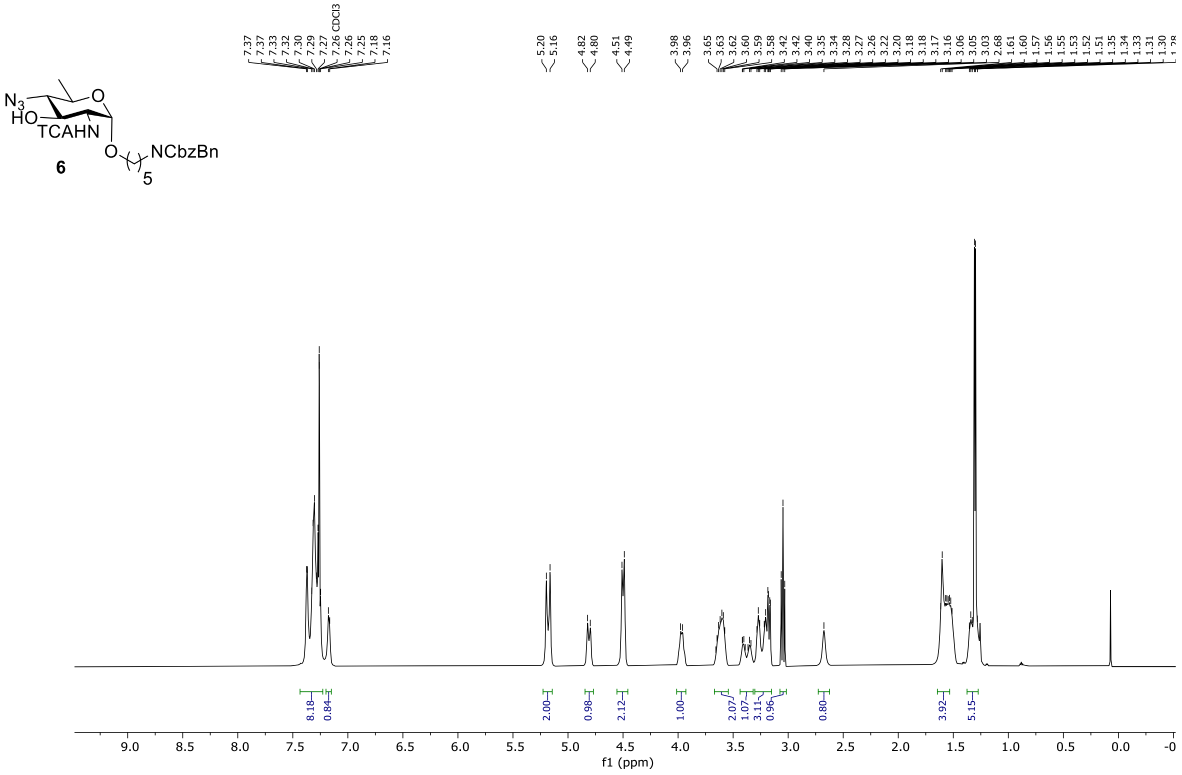

^{13}C NMR (101 MHz, $CDCl_3$)

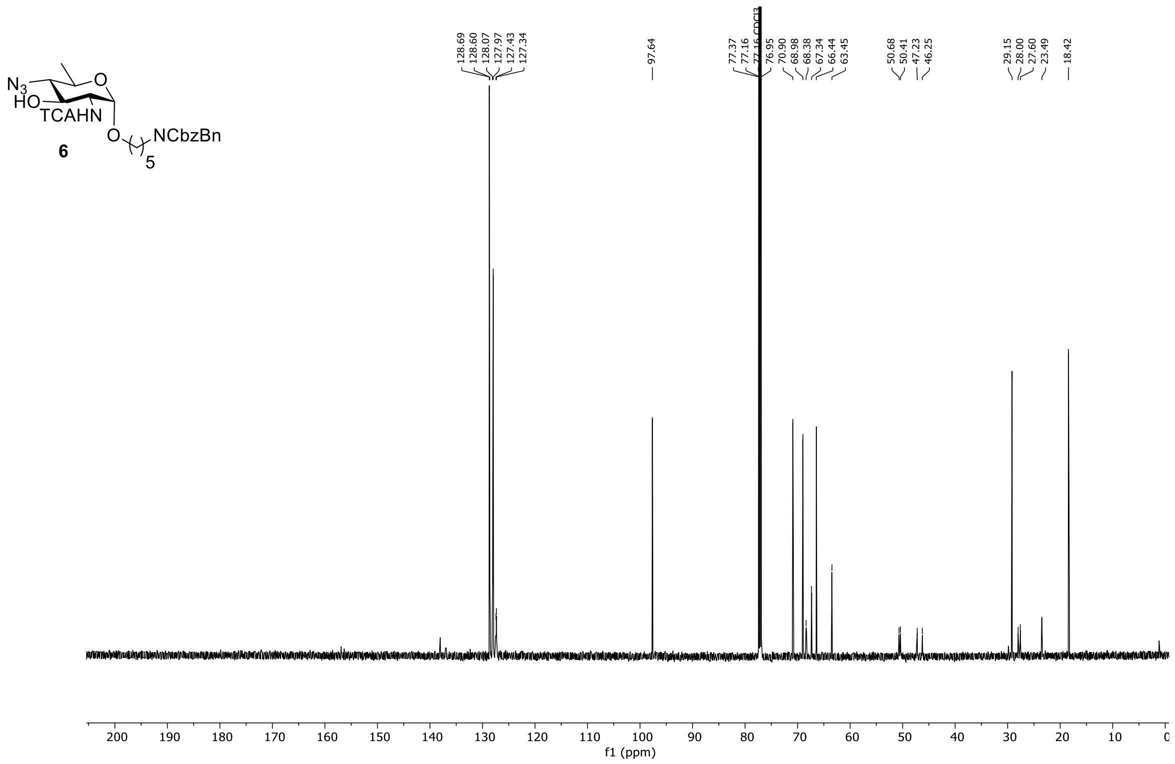

^{1}H-^{1}H COSY NMR (400 MHz, $CDCl_3$)

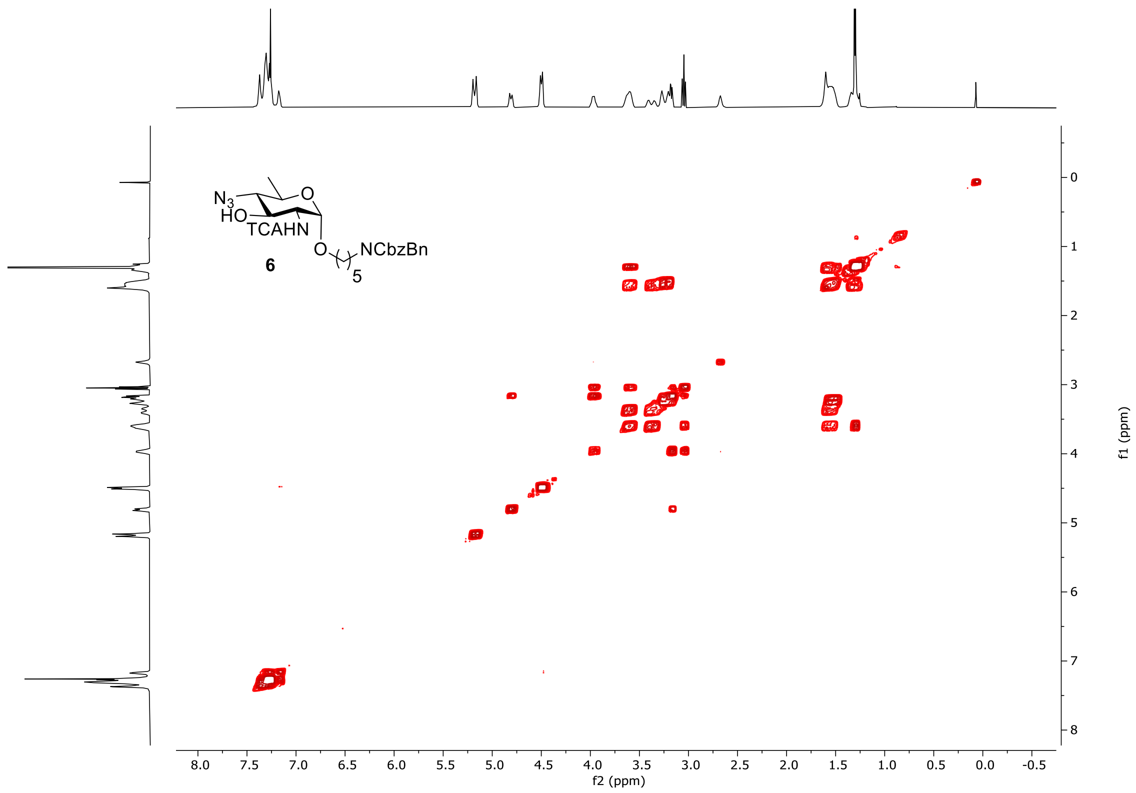

^{1}H-^{13}C HSQC NMR (400 MHz, CDCl$_3$)

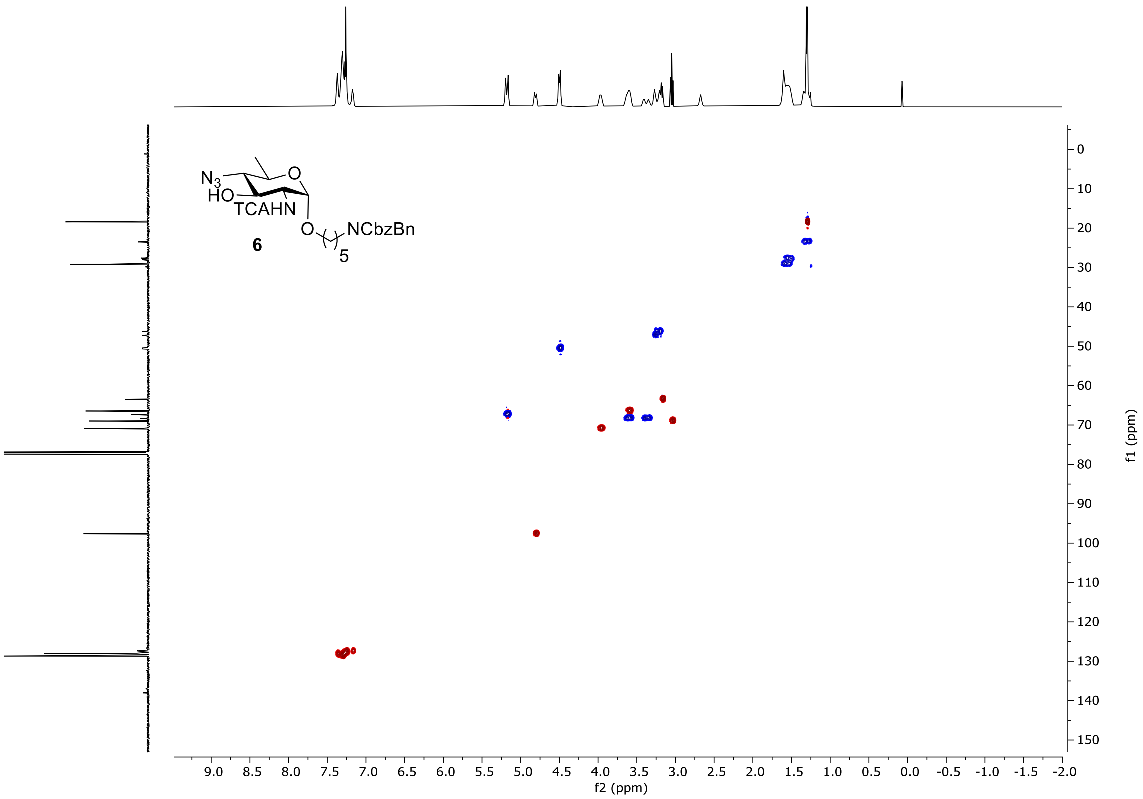

^{1}H-^{13}C Coupled HSQC NMR (400 MHz, CDCl$_3$)

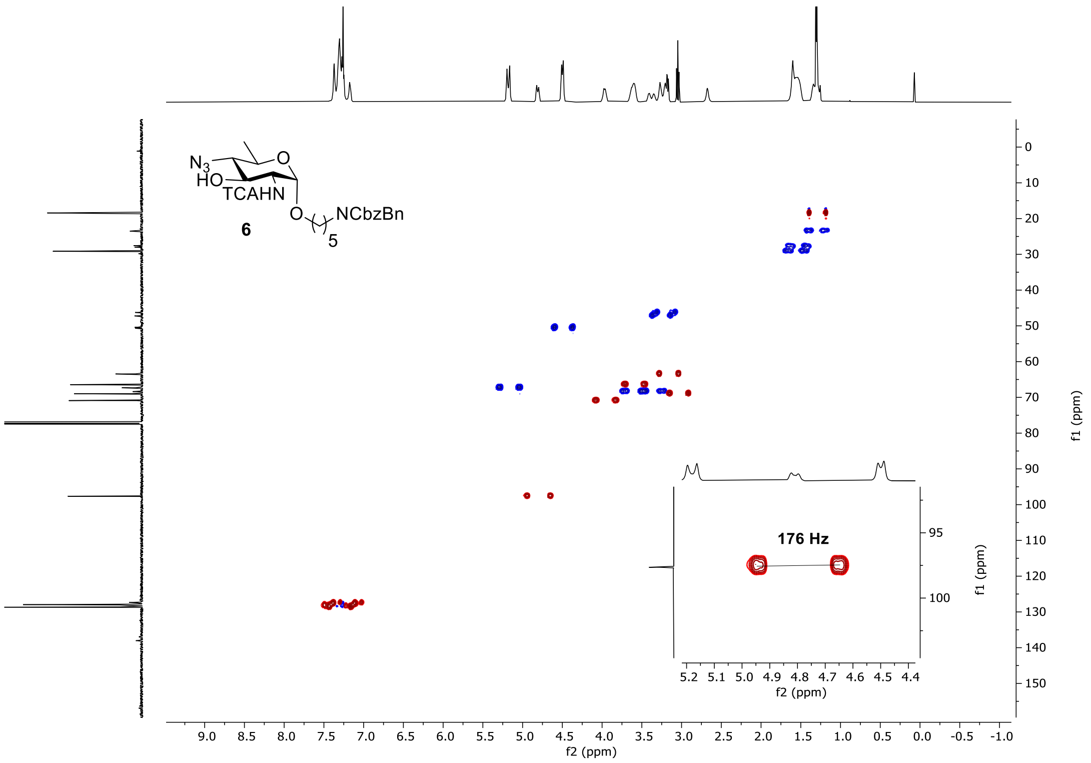

^{1}H NMR (400 MHz, CDCl$_3$)

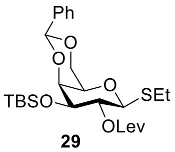

29

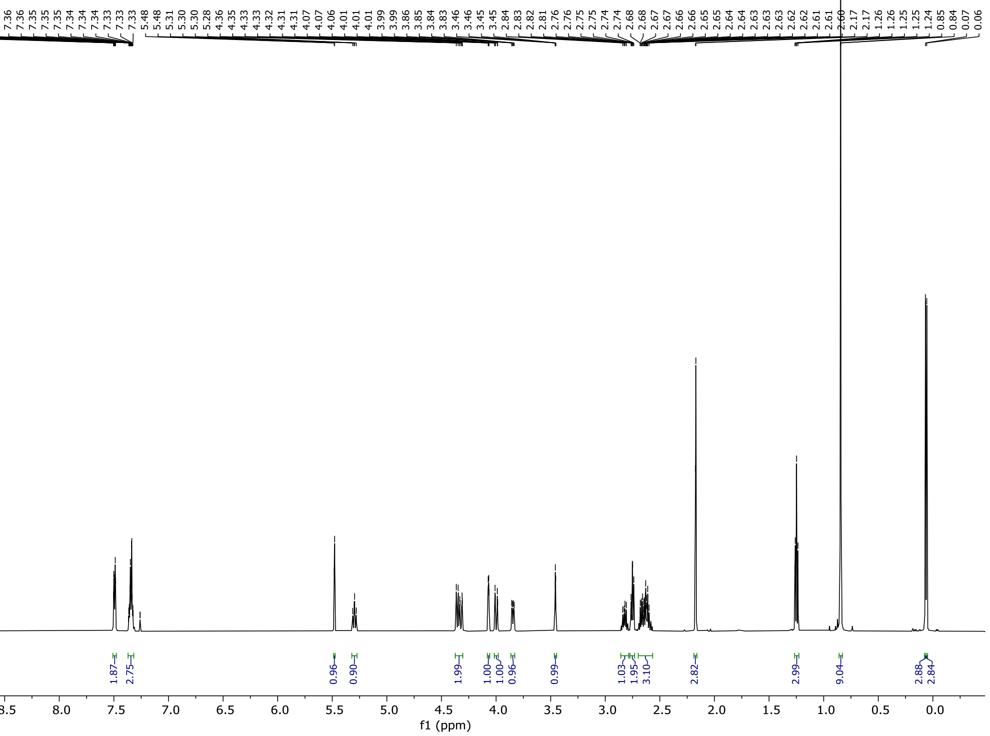

^{13}C NMR (101 MHz, CDCl$_3$)

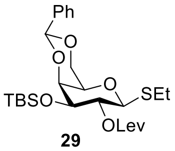

29

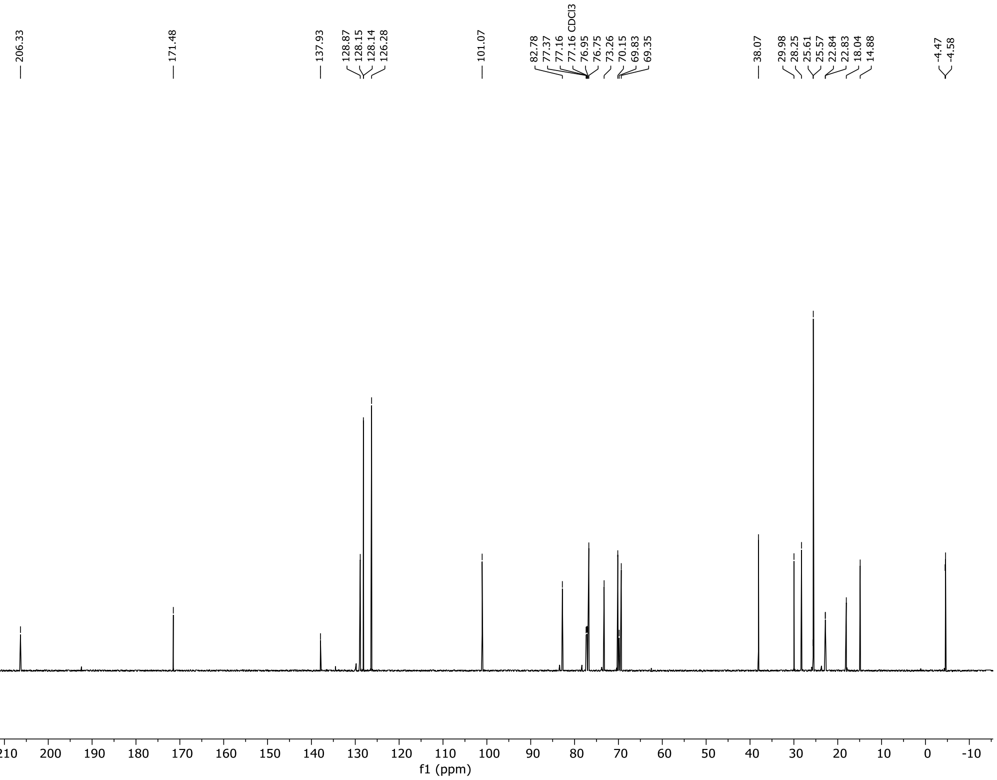

^{1}H-^{1}H COSY NMR (400 MHz, $CDCl_3$)

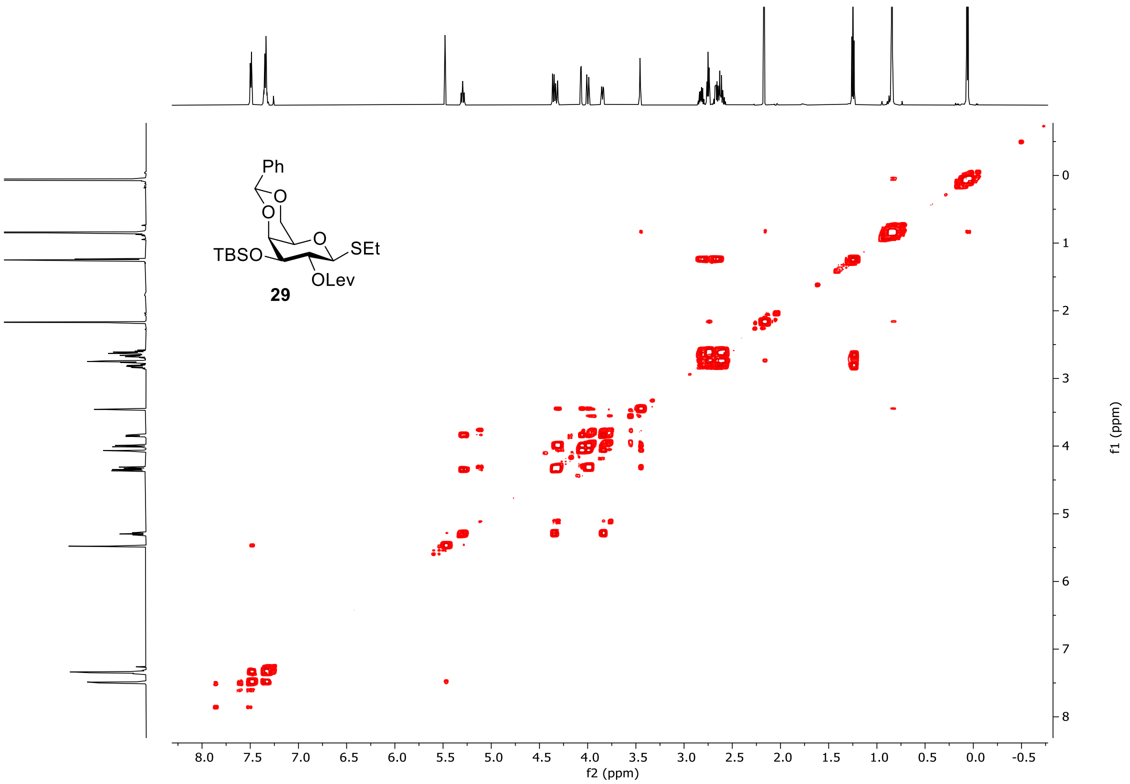

^{1}H-^{13}C HSQC NMR (400 MHz, $CDCl_3$)

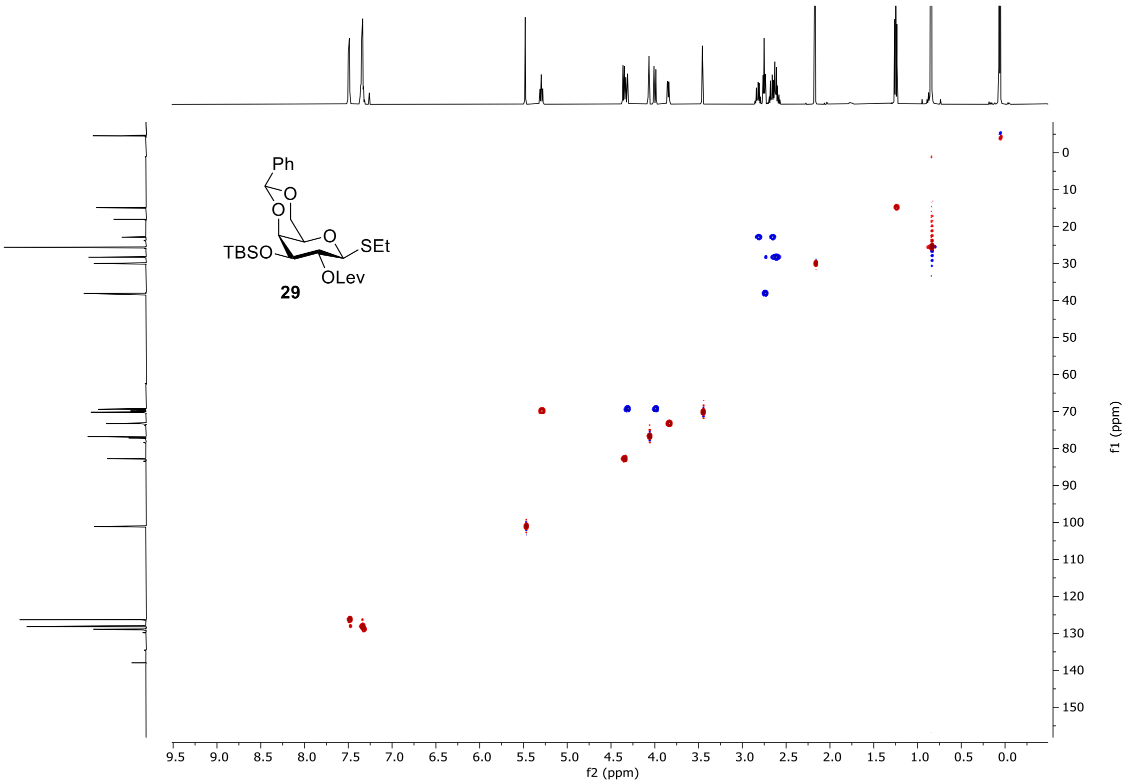

^{1}H NMR (400 MHz, $CDCl_3$)

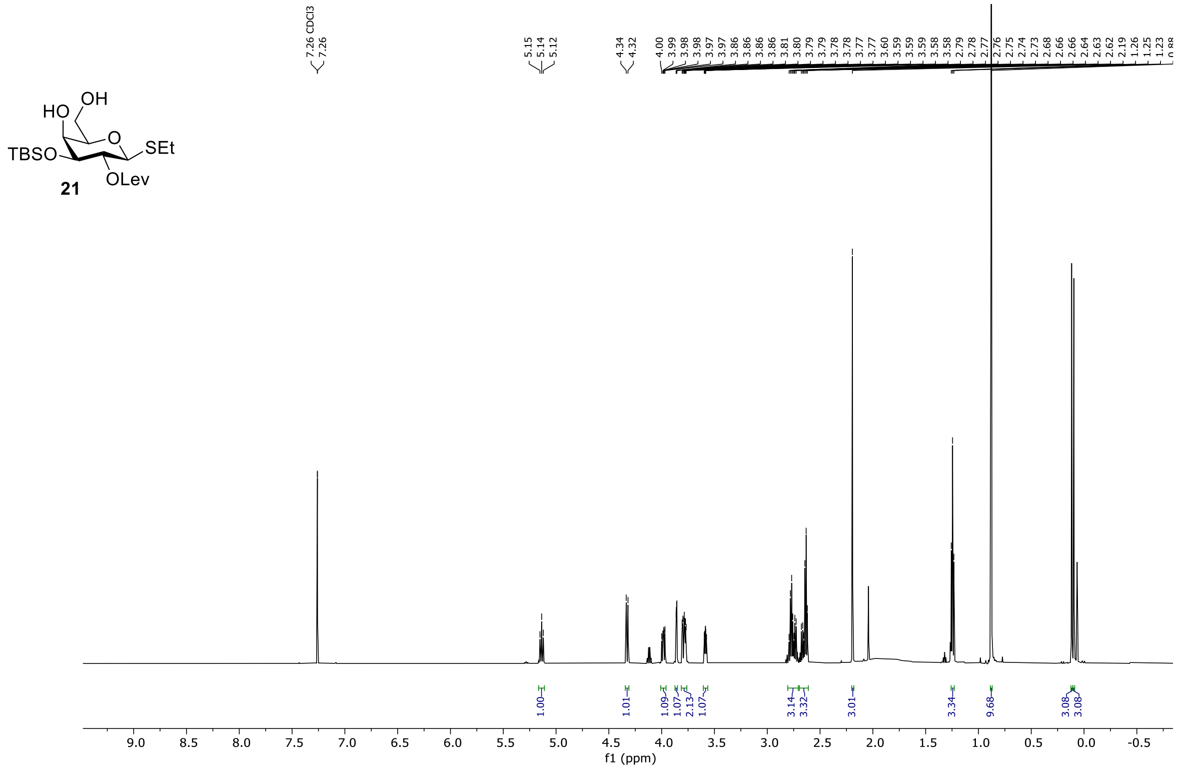

^{13}C NMR (101 MHz, $CDCl_3$)

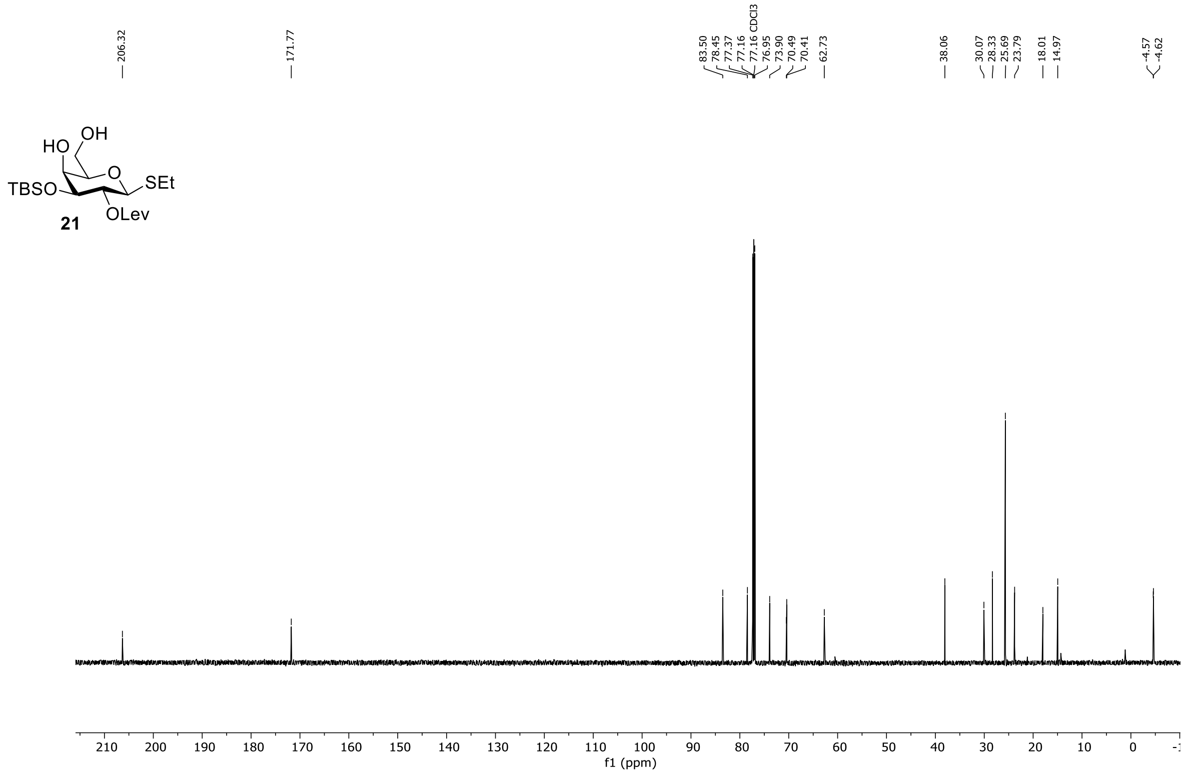

^{1}H-^{1}H COSY NMR (400 MHz, $CDCl_3$)

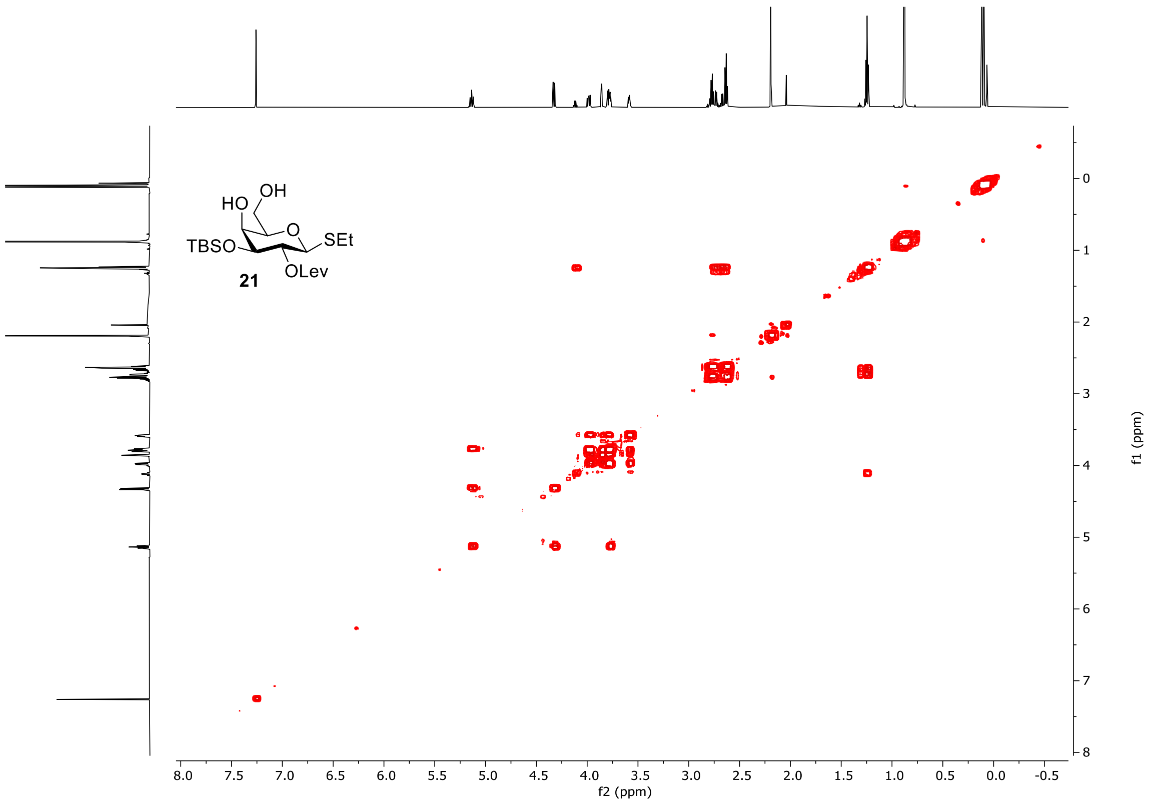

^{1}H-^{13}C HSQC NMR (400 MHz, $CDCl_3$)

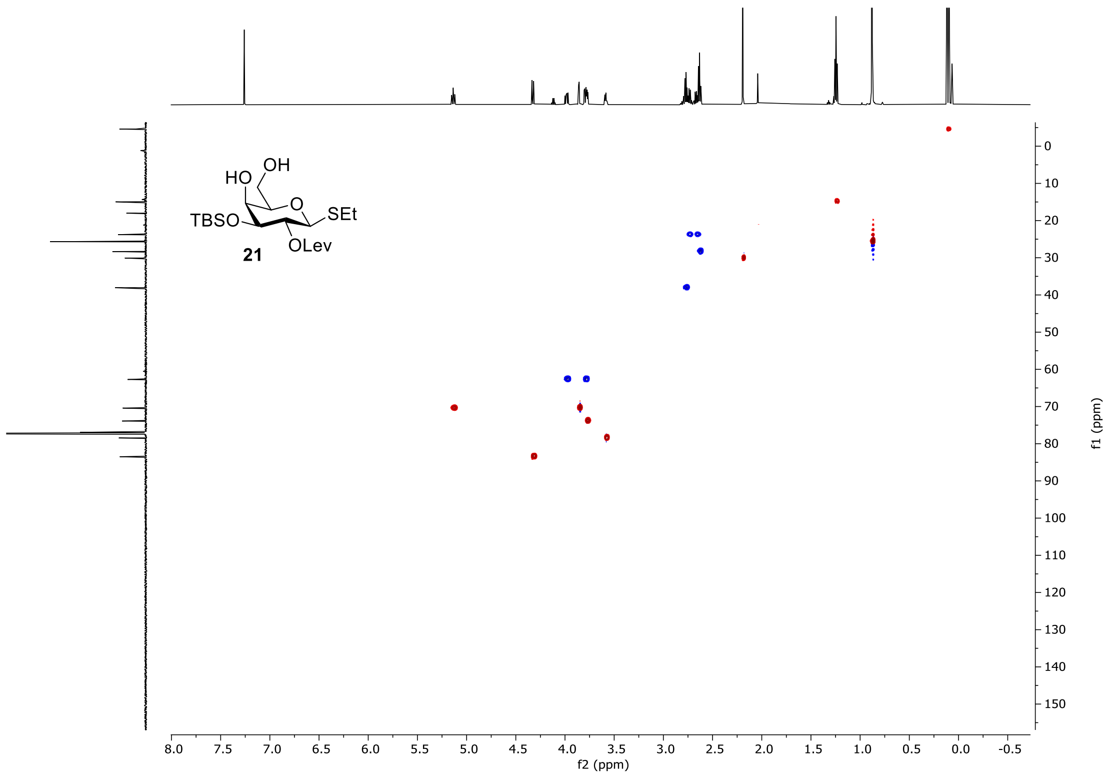

^{1}H NMR (400 MHz, $CDCl_3$)

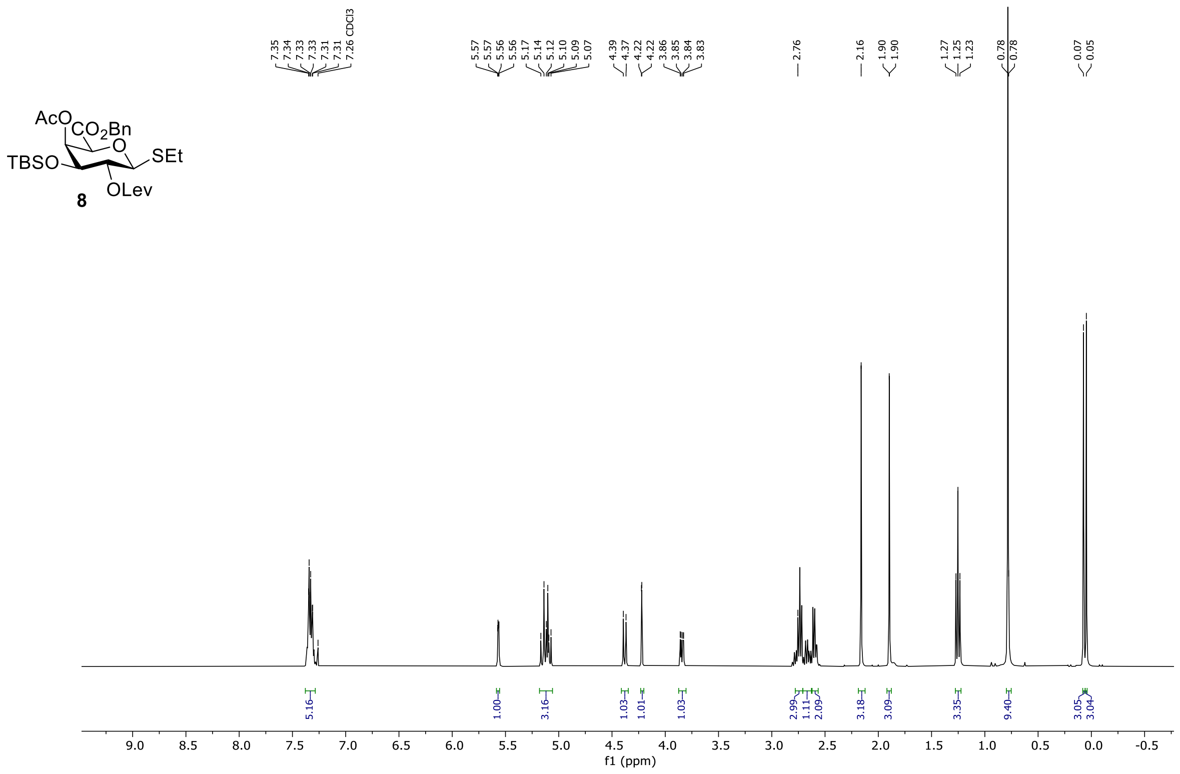

^{13}C NMR (101 MHz, $CDCl_3$)

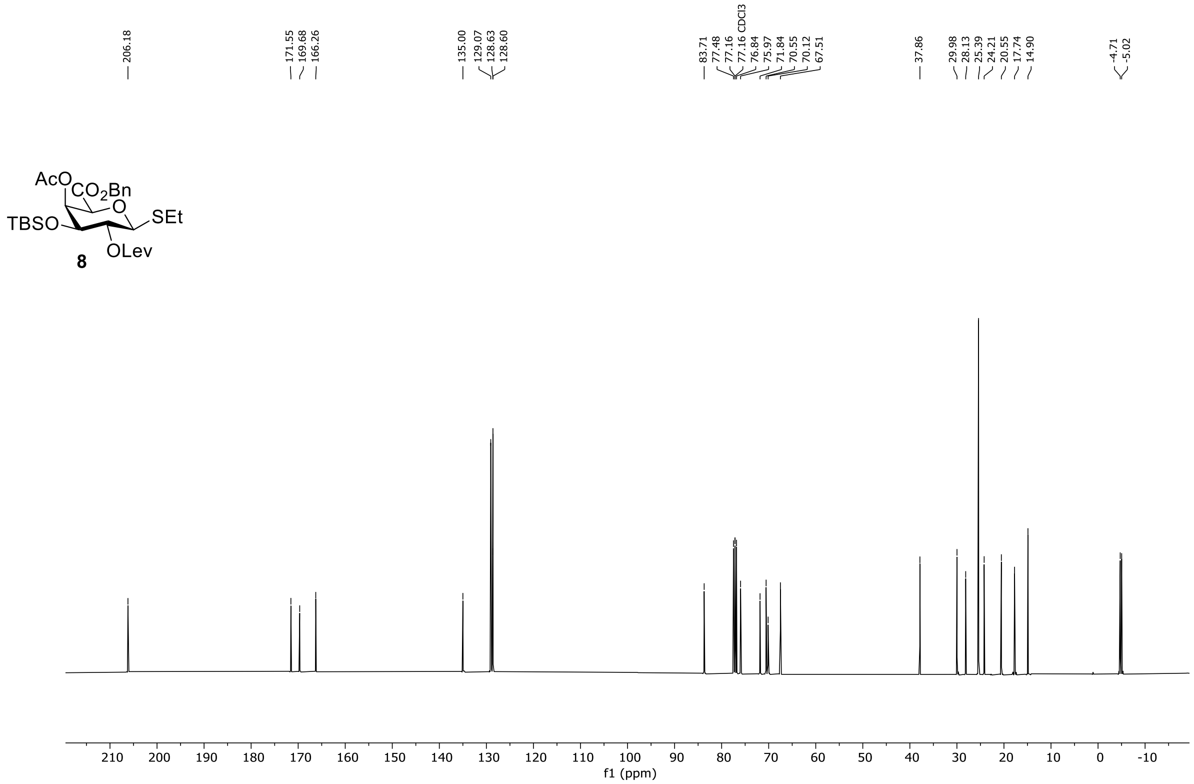

^{1}H-^{1}H COSY NMR (400 MHz, $CDCl_3$)

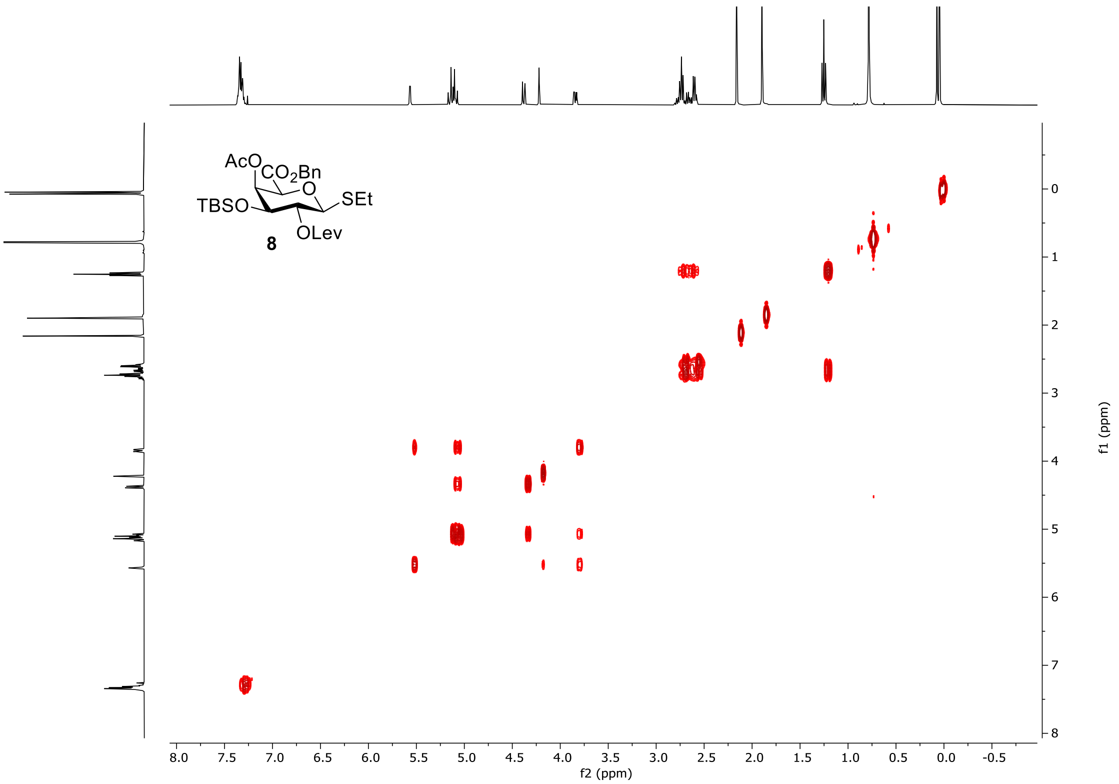

^{1}H-^{13}C HSQC NMR (400 MHz, $CDCl_3$)

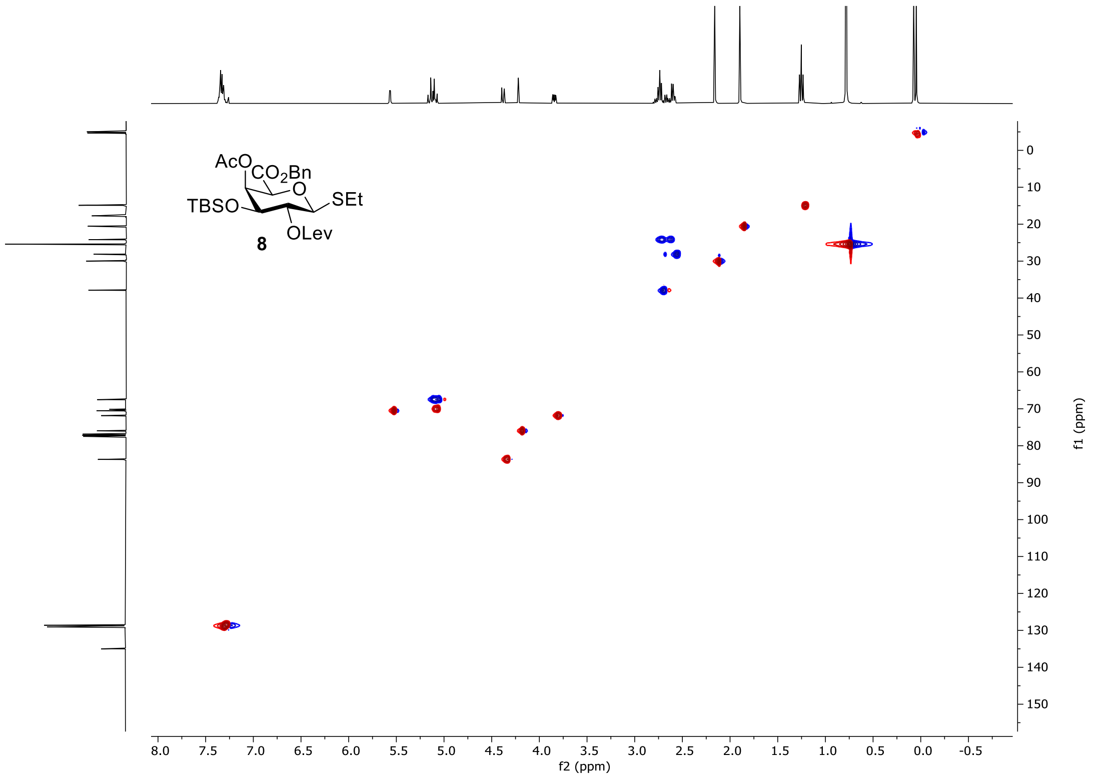

^{1}H NMR (400 MHz, $CDCl_3$)

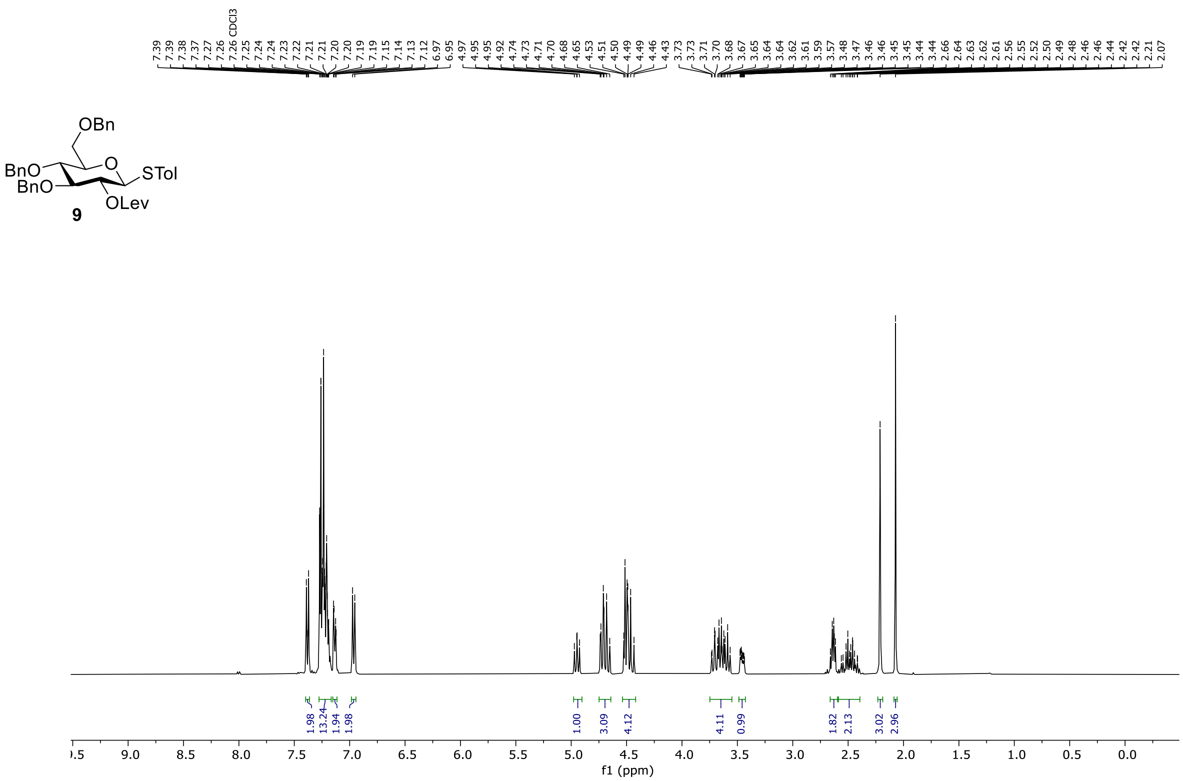

^{13}C NMR (101 MHz, $CDCl_3$)

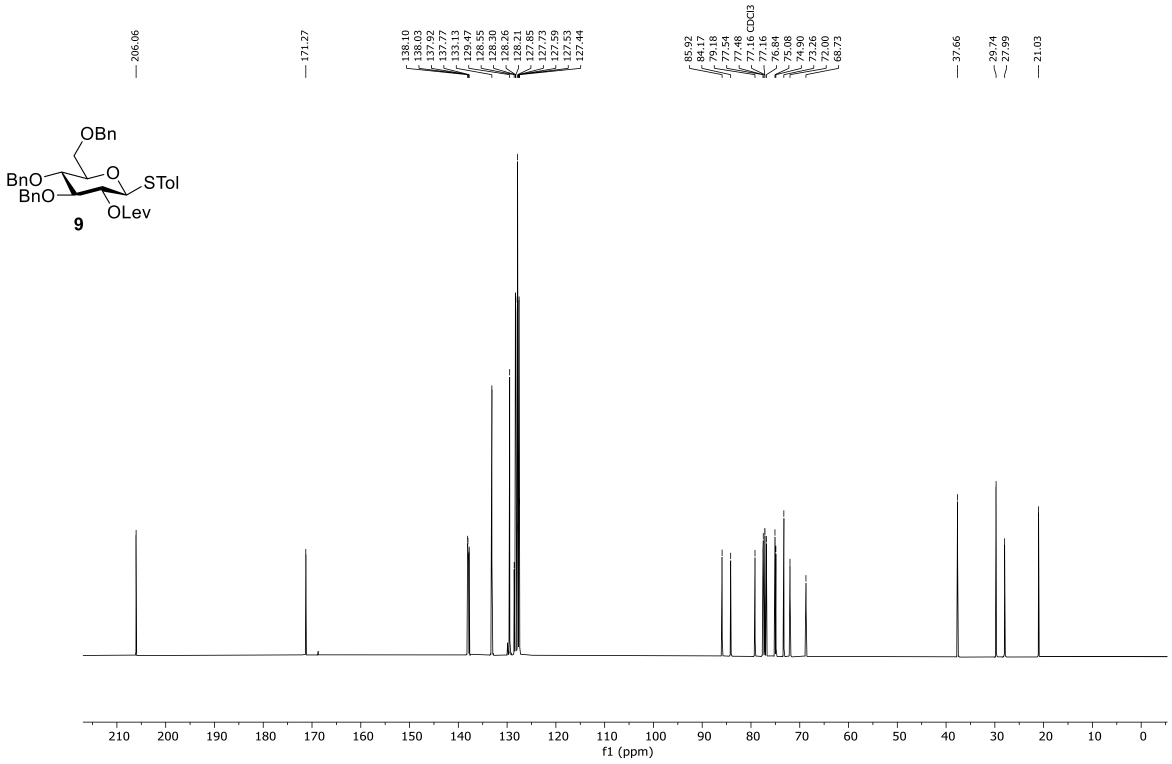

^{1}H-^{1}H COSY NMR (400 MHz, $CDCl_3$)

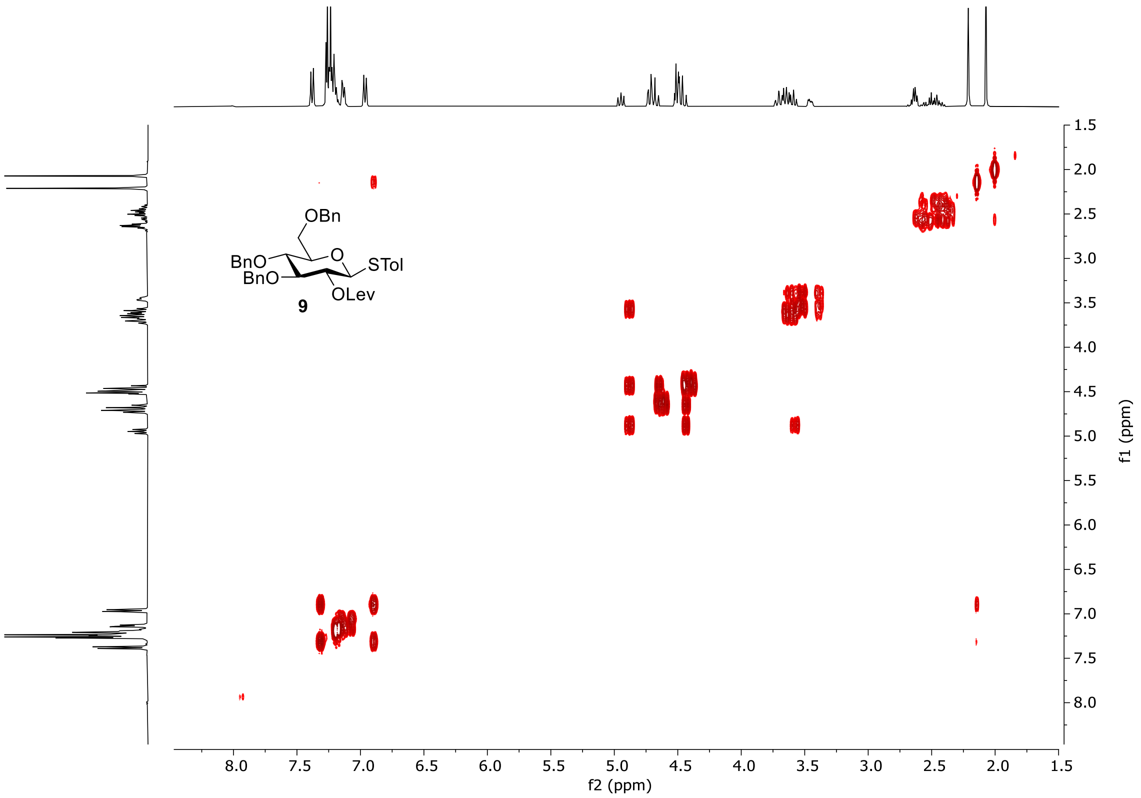

^{1}H-^{13}C HSQC NMR (400 MHz, $CDCl_3$)

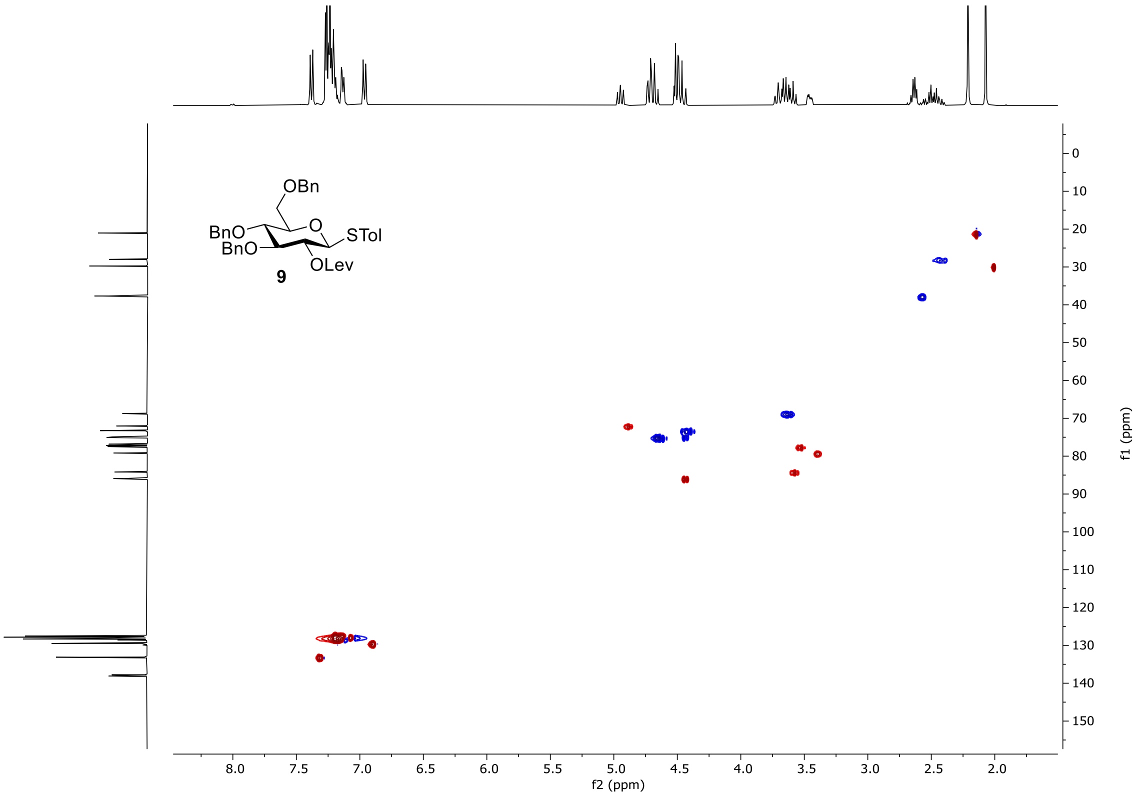

^{1}H NMR (600 MHz, $CDCl_3$)

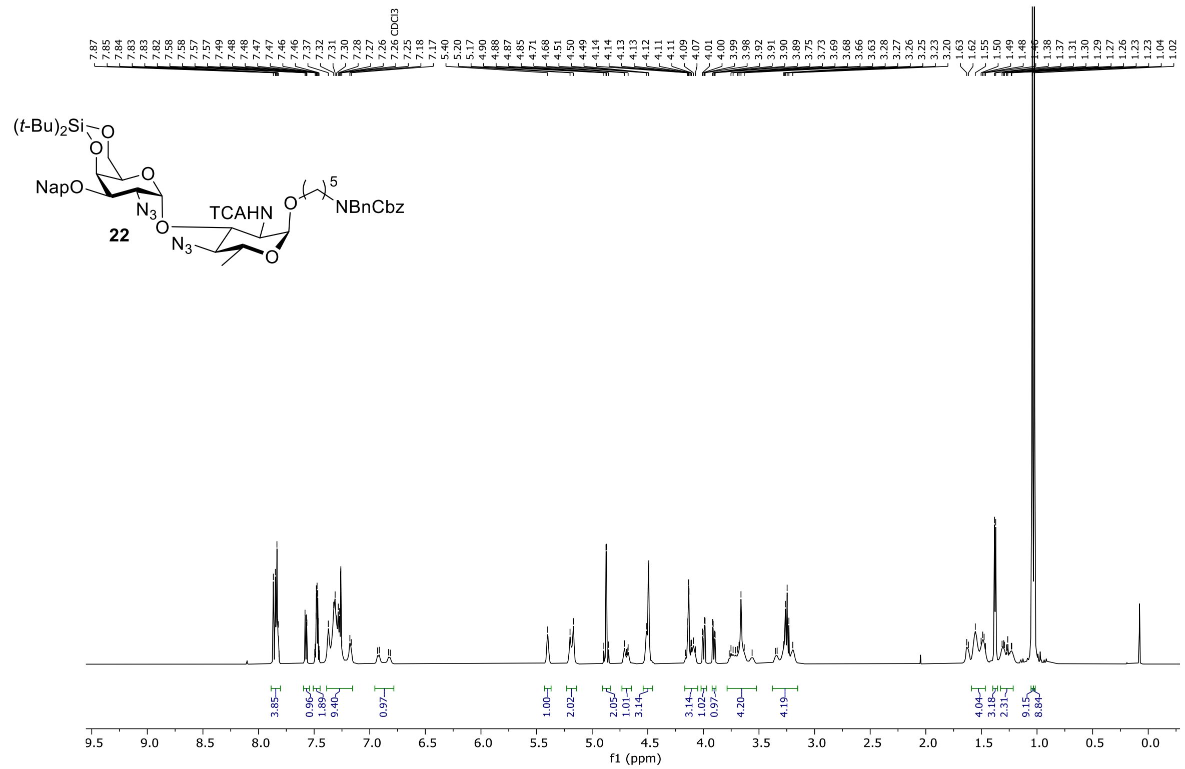

^{13}C NMR (151 MHz, $CDCl_3$)

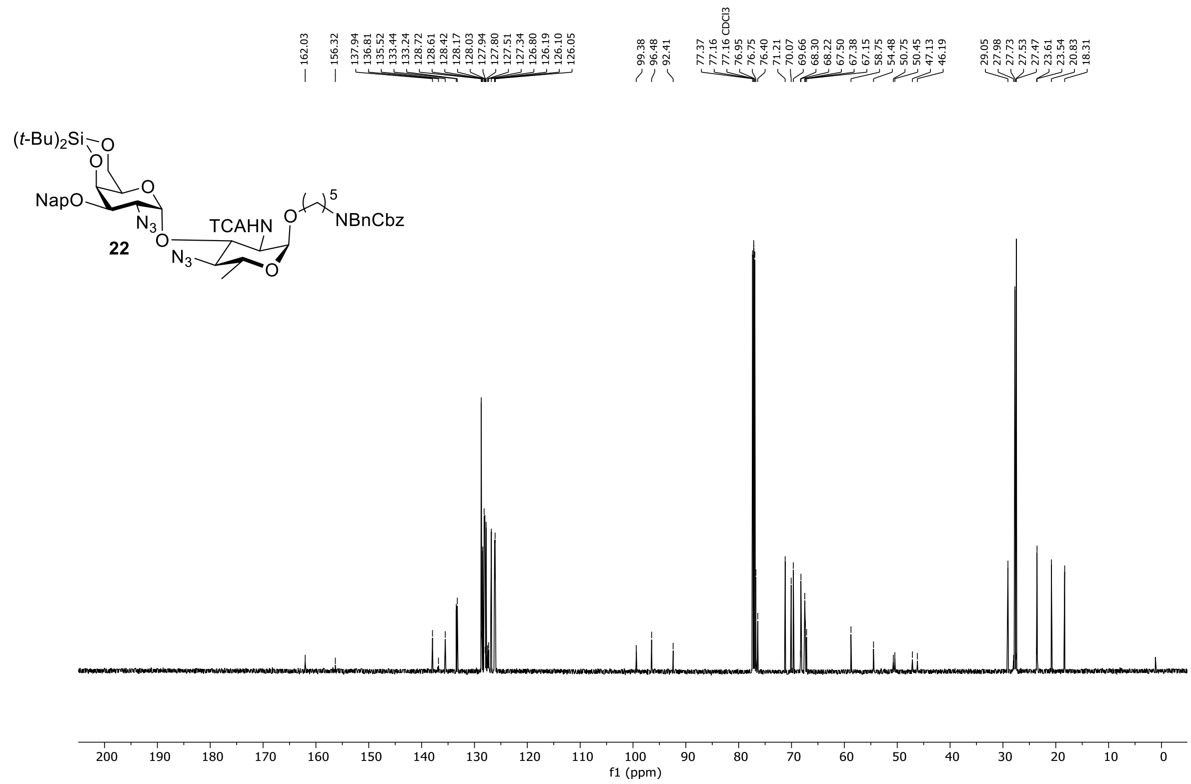

^{1}H-^{1}H COSY NMR (600 MHz, $CDCl_3$)

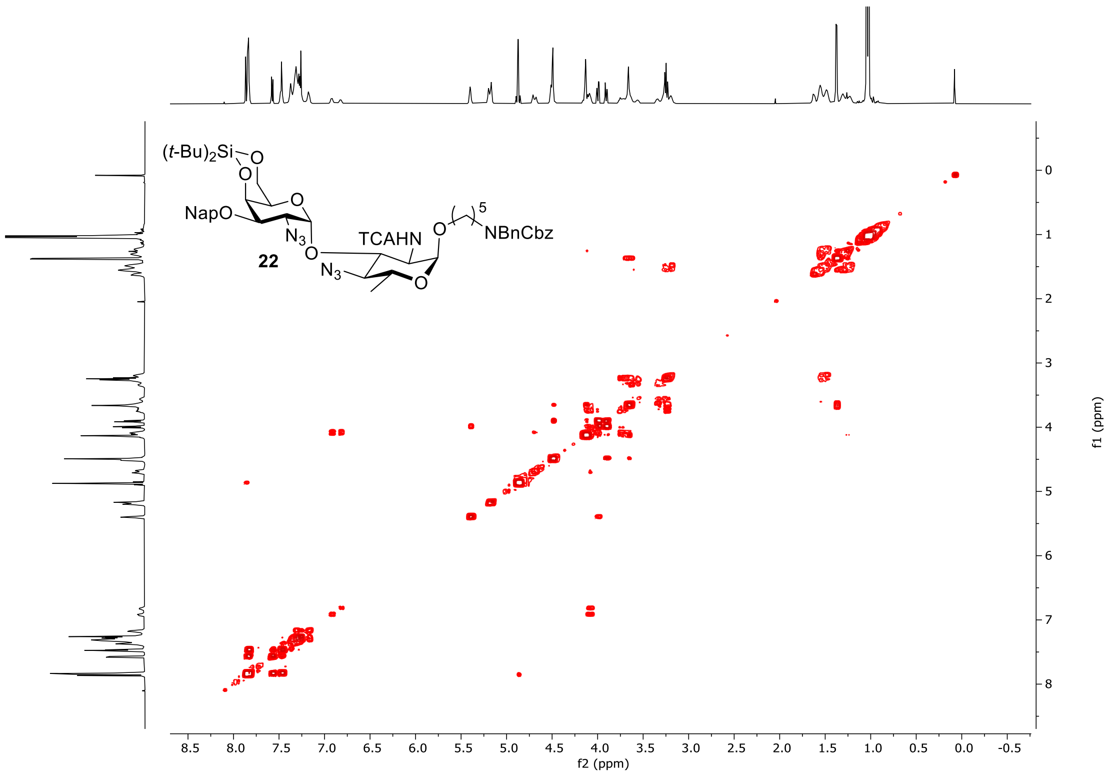

^{1}H-^{13}C HSQC NMR (600 MHz, $CDCl_3$)

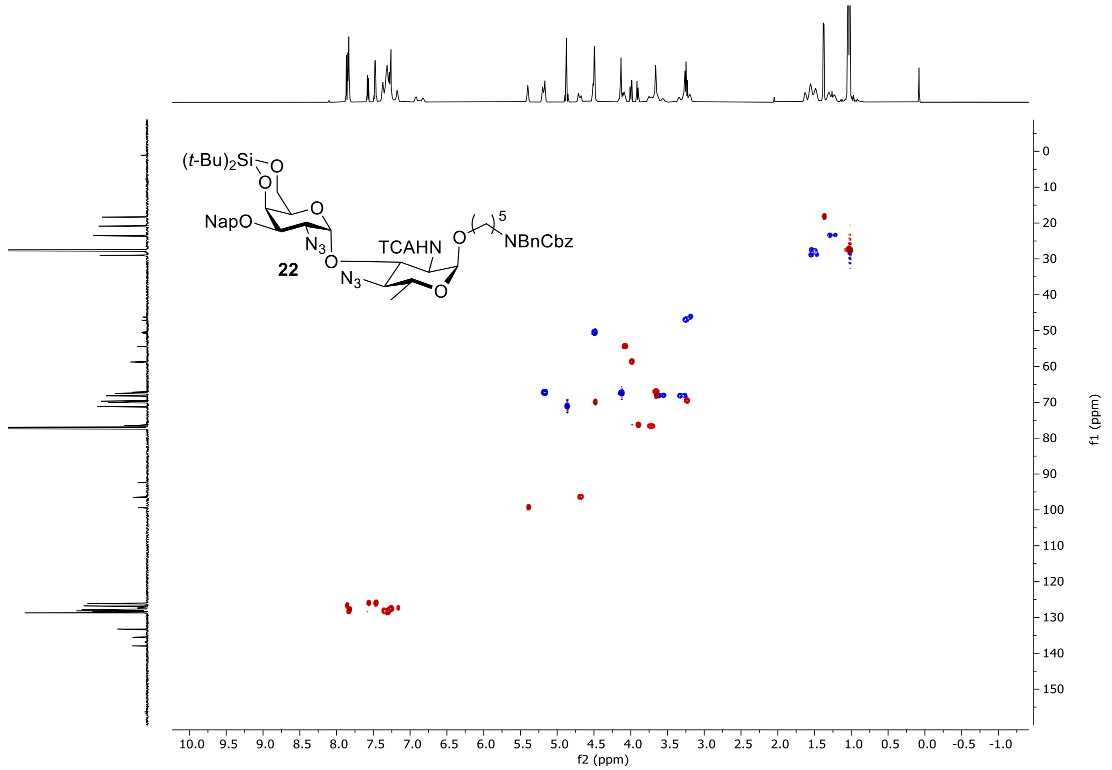

^{1}H-^{13}C Coupled HSQC NMR (600 MHz, $CDCl_3$)

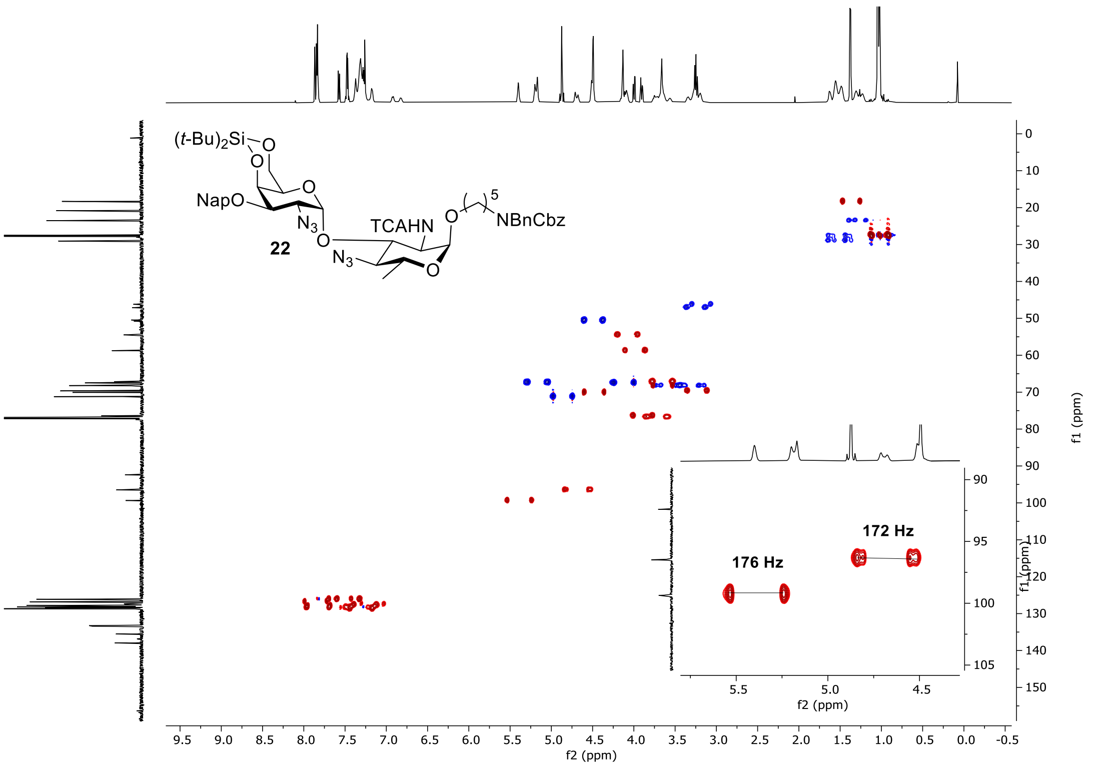

^{1}H NMR (600 MHz, $CDCl_3$)

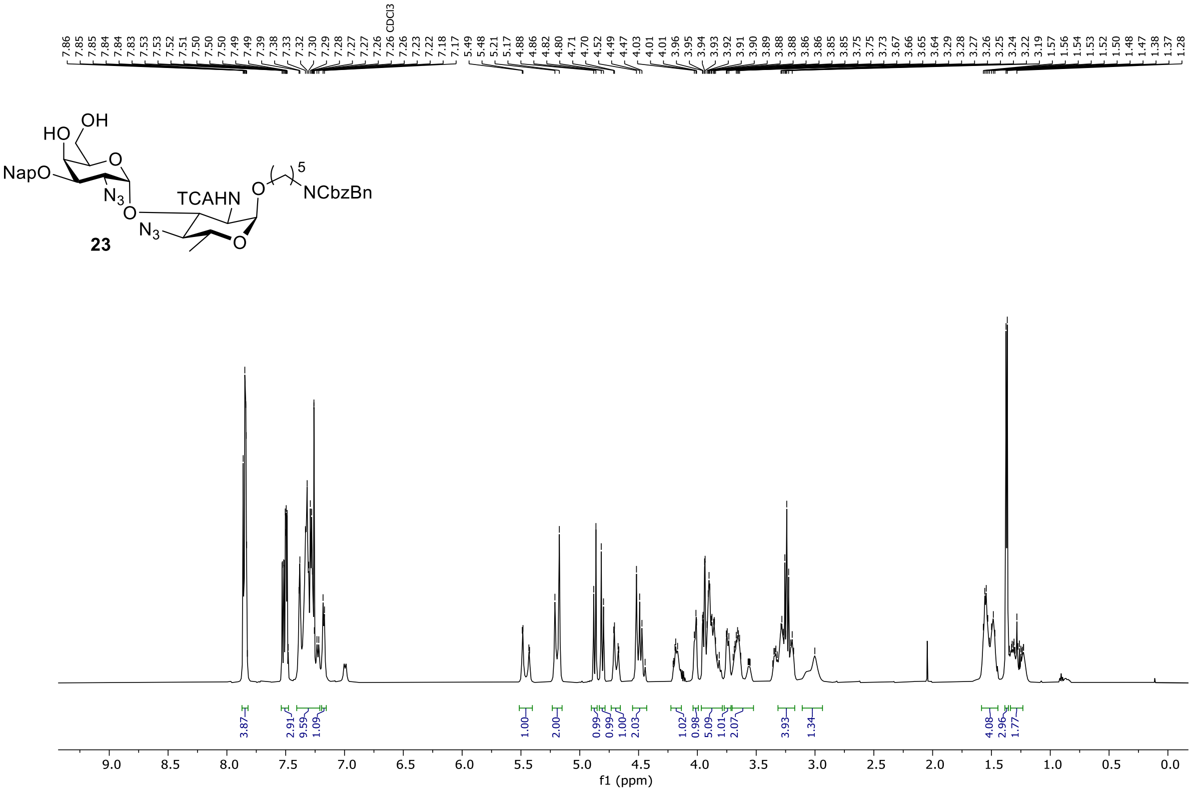

^{13}C NMR (151 MHz, $CDCl_3$)

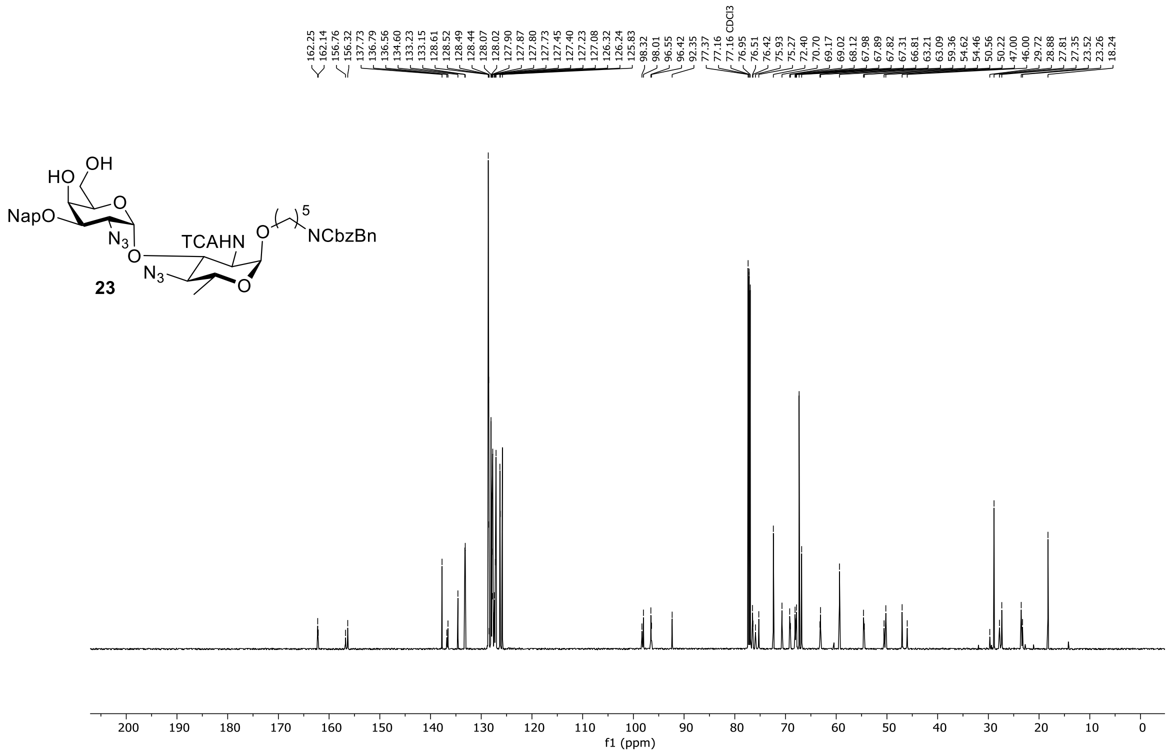

^{1}H-^{1}H COSY NMR (600 MHz, $CDCl_3$)

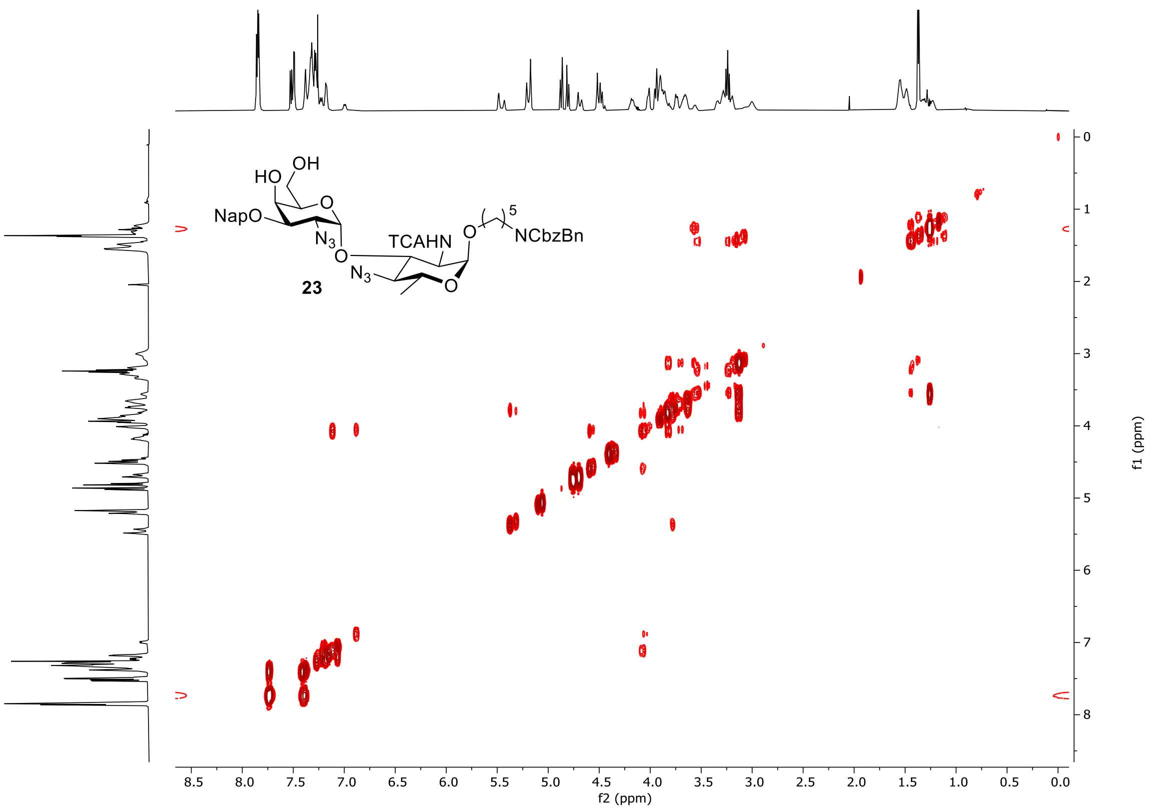

^{1}H-^{13}C HSQC NMR (600 MHz, $CDCl_3$)

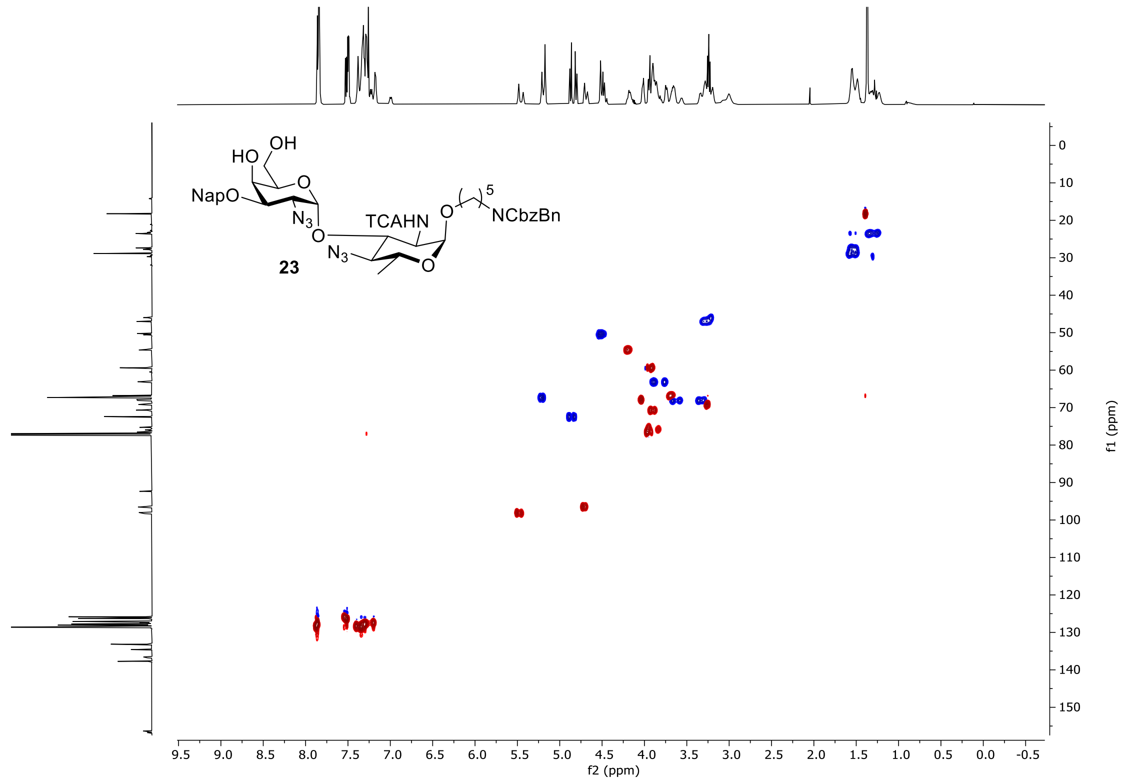

^{1}H-^{13}C Coupled HSQC NMR (600 MHz, $CDCl_3$)

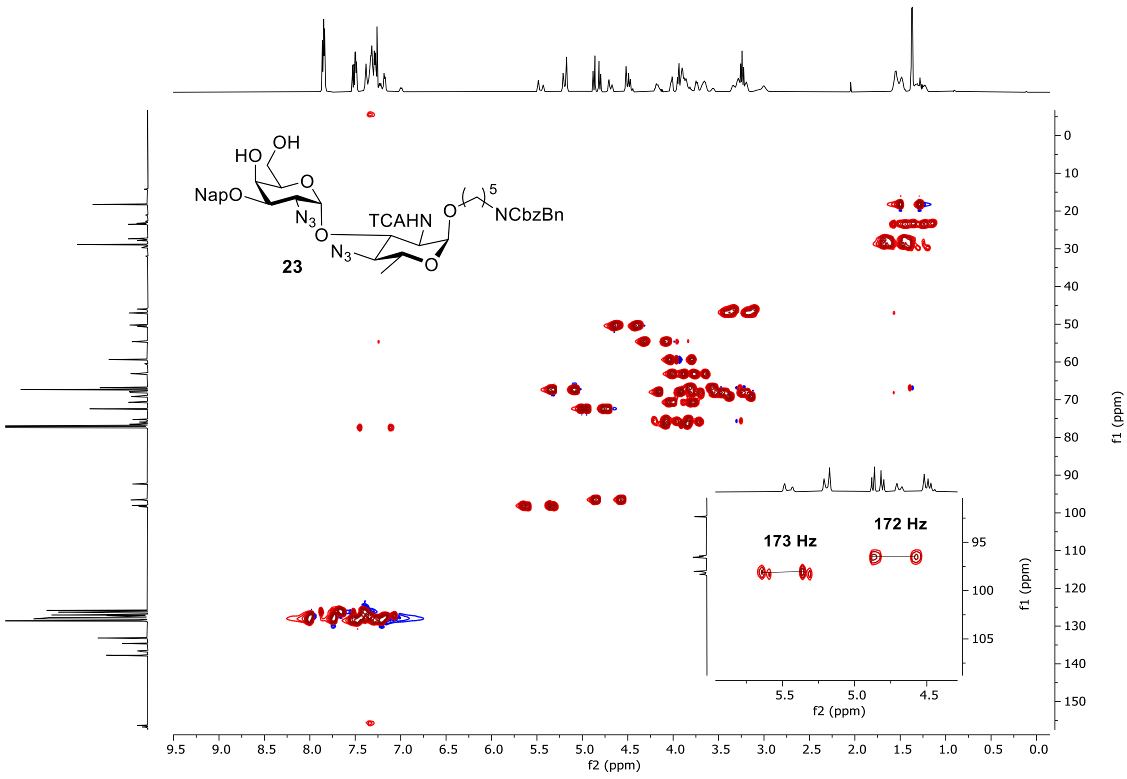

^{1}H NMR (600 MHz, $CDCl_3$)

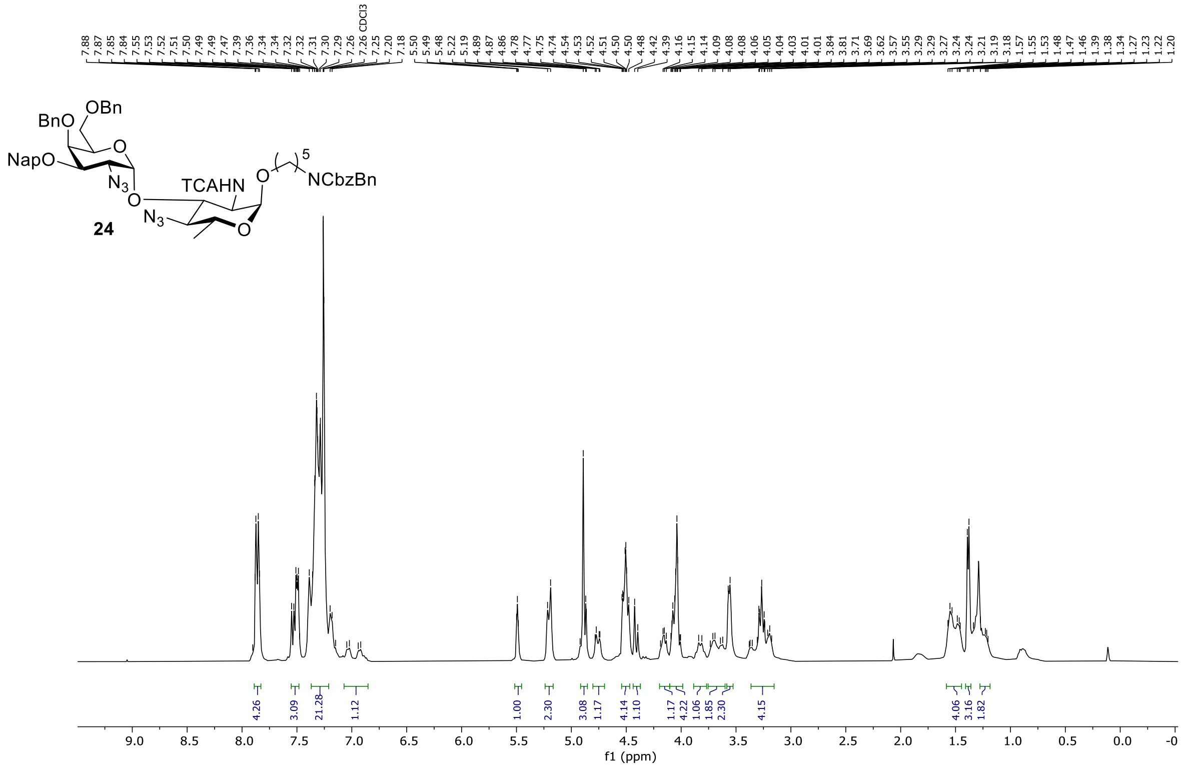

^{1}H-^{13}C NMR (151 MHz, $CDCl_3$)

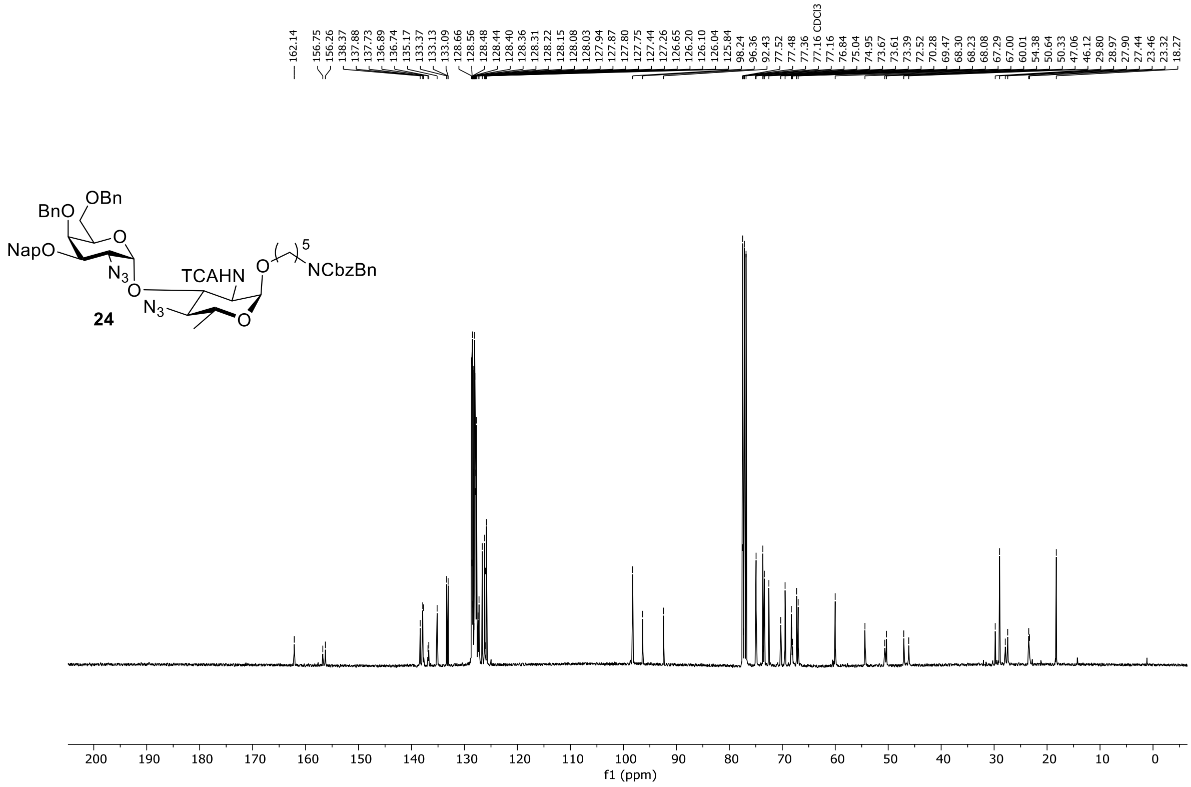

^{1}H-^{1}H COSY NMR (600 MHz, $CDCl_3$)

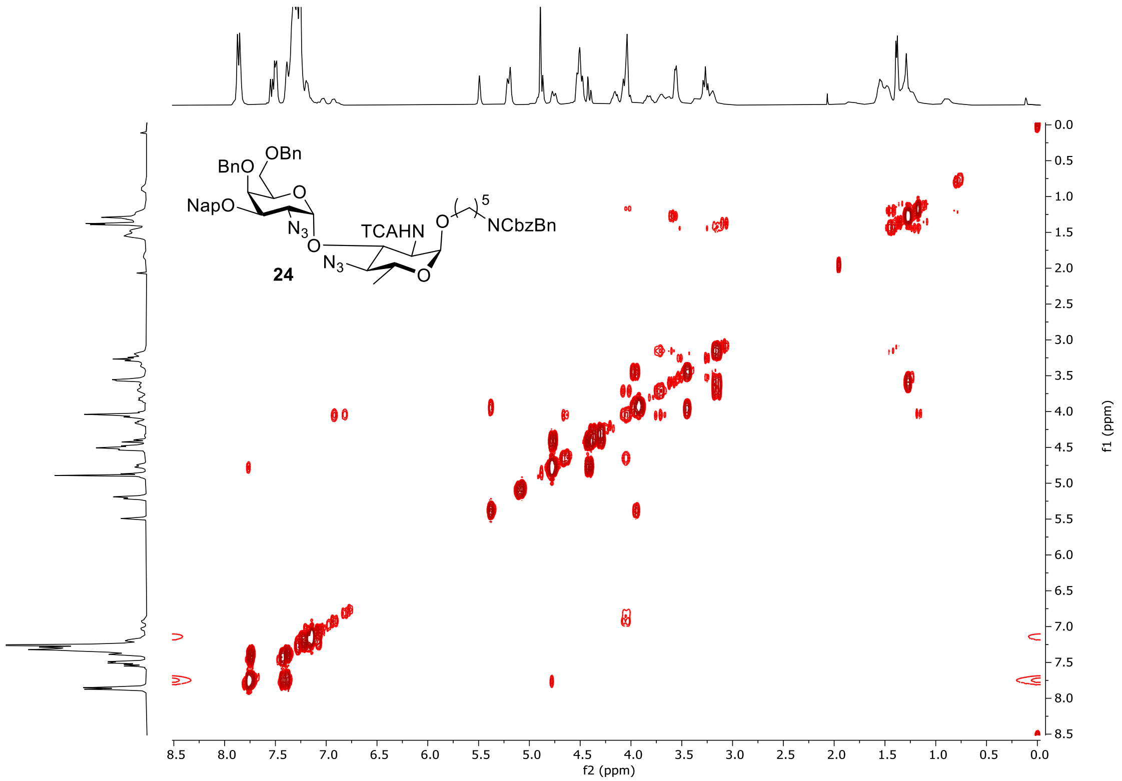

^{1}H-^{13}C HSQC NMR (600 MHz, $CDCl_3$)

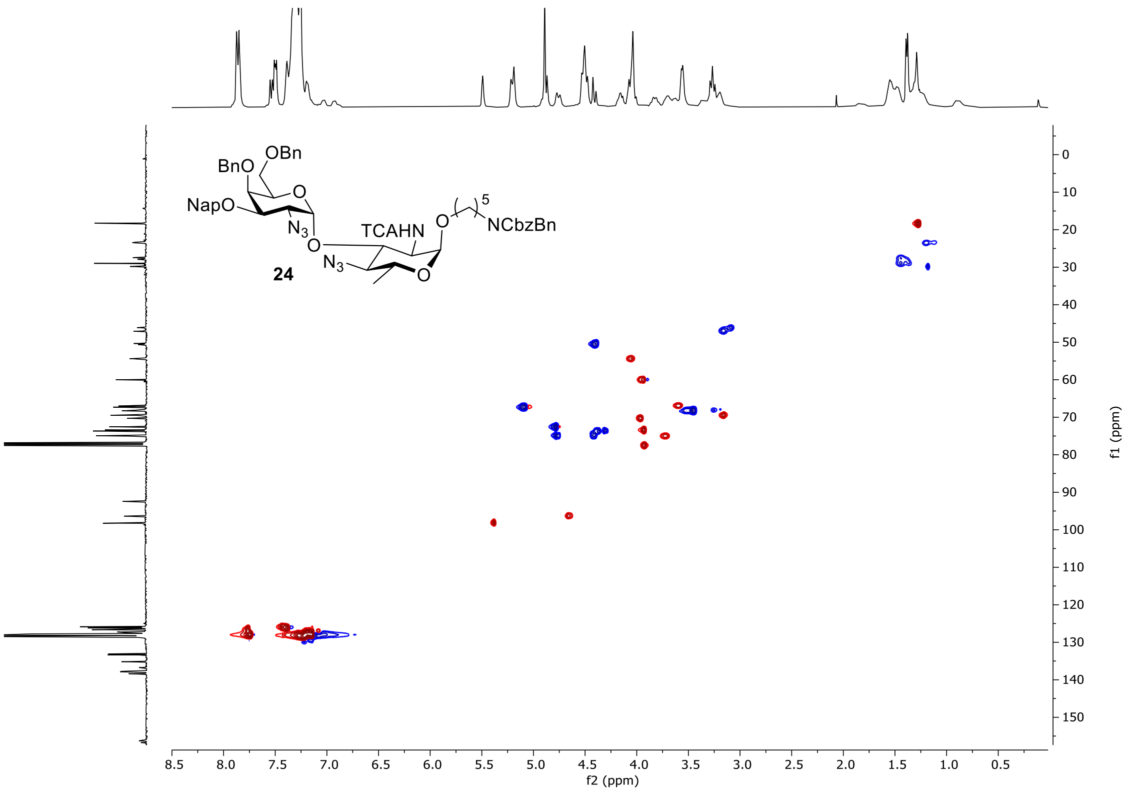

^{1}H-^{13}C Coupled HSQC NMR (600 MHz, $CDCl_3$)

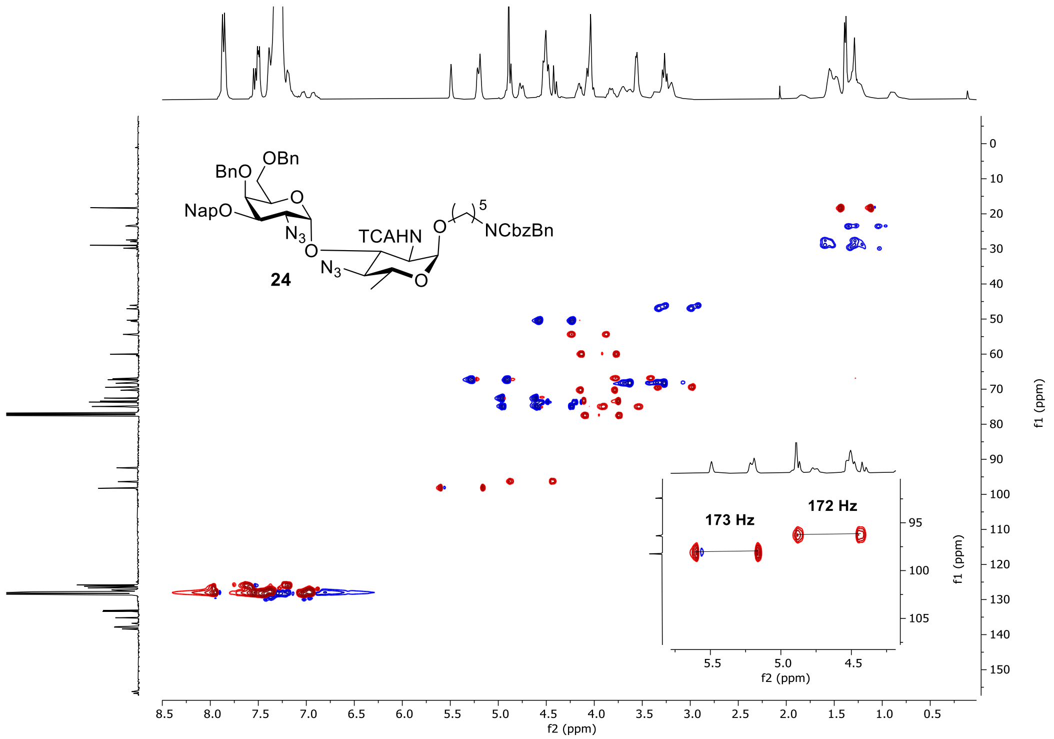

^{1}H NMR (600 MHz, $CDCl_3$)

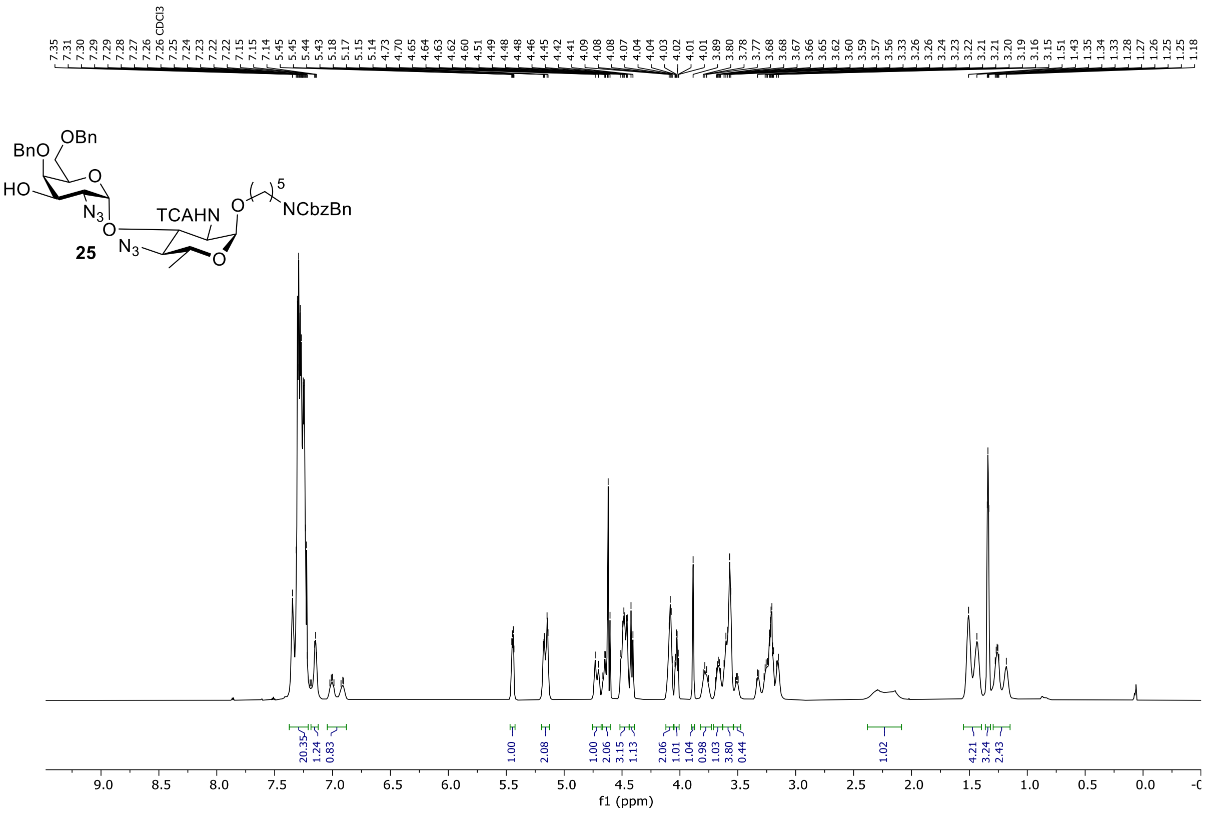

^{13}C NMR (151 MHz, $CDCl_3$)

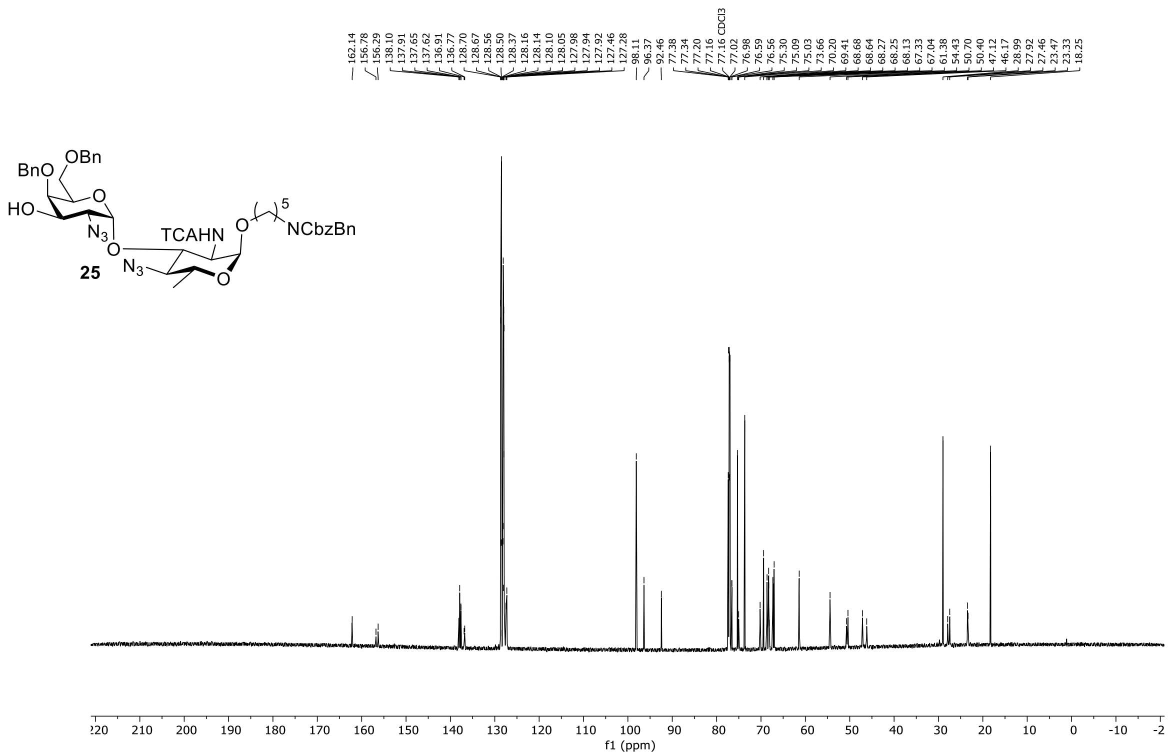

^{1}H-^{1}H COSY NMR (600 MHz, $CDCl_3$)

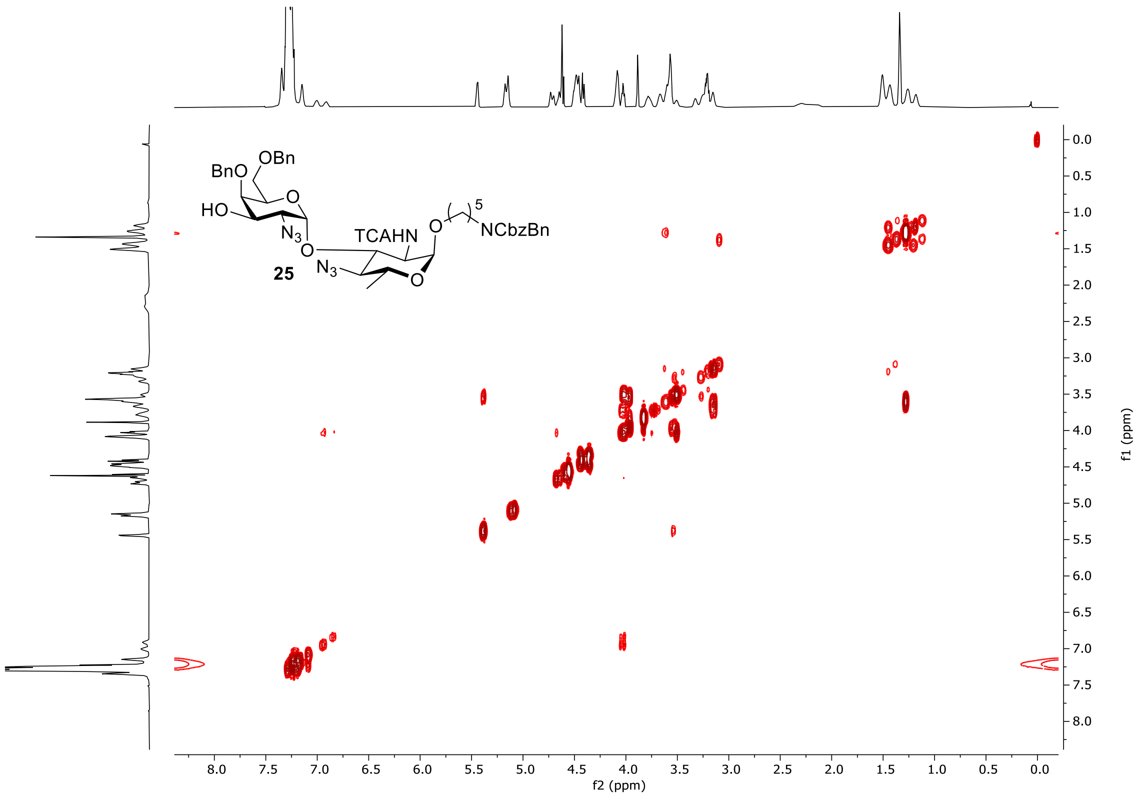

^1H-^{13}C HSQC NMR (600 MHz, $CDCl_3$)

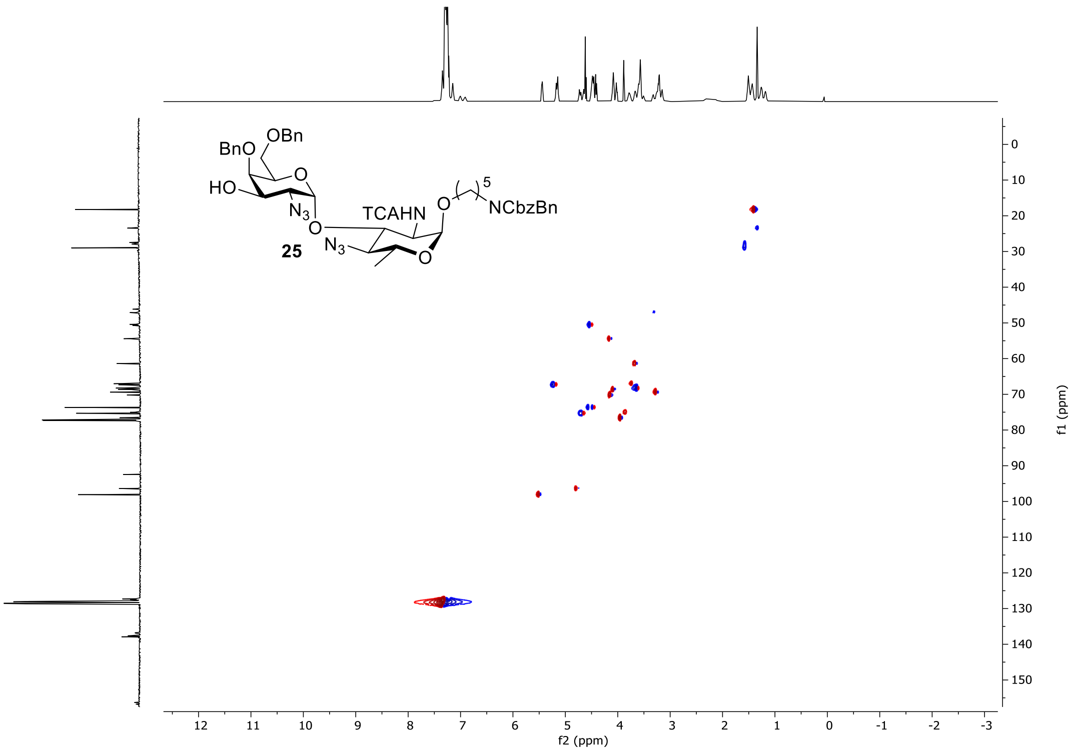

^1H-^{13}C HSQC Coupled NMR (600 MHz, $CDCl_3$)

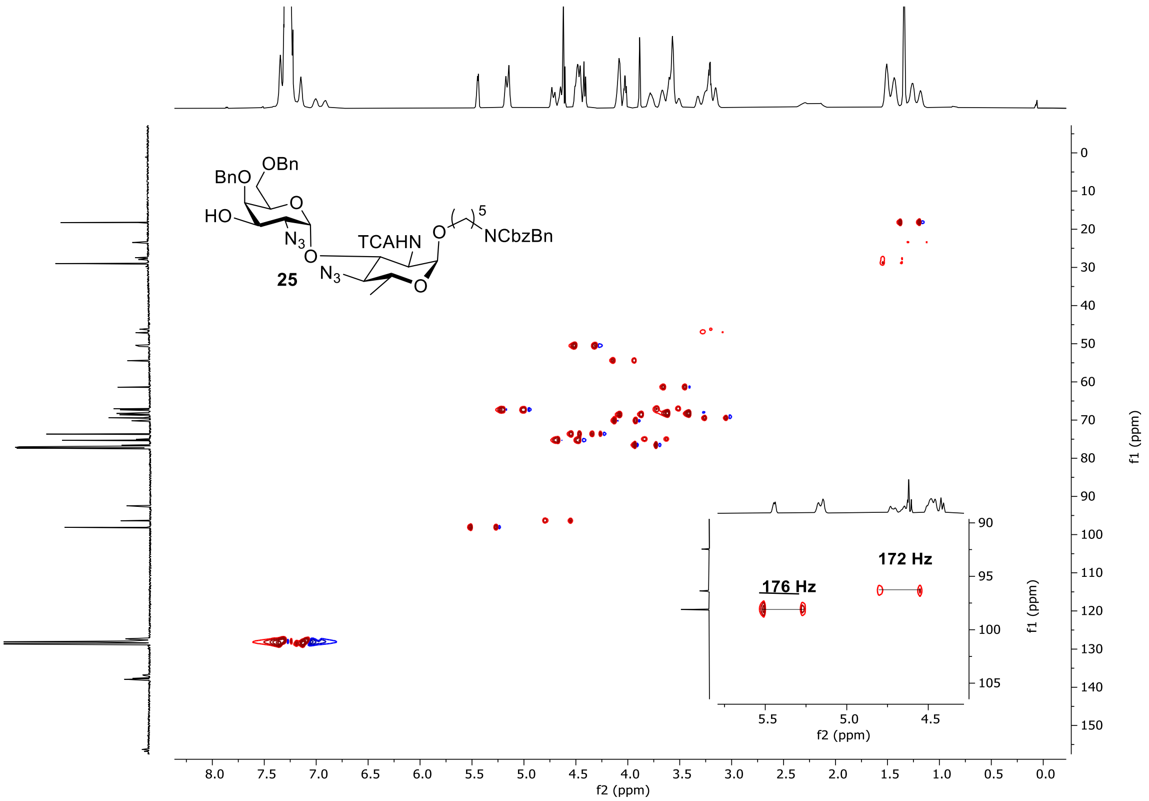

^{1}H NMR (400 MHz, $CDCl_3$)

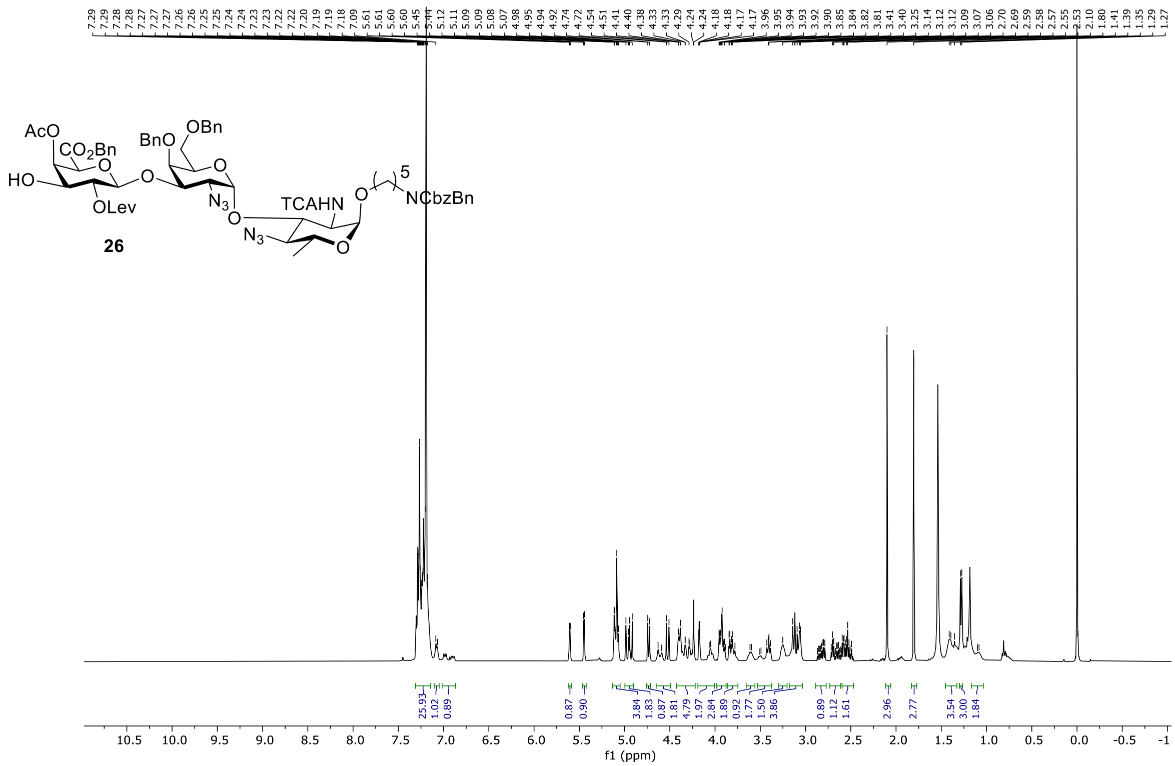

^{13}C NMR (101 MHz, $CDCl_3$)

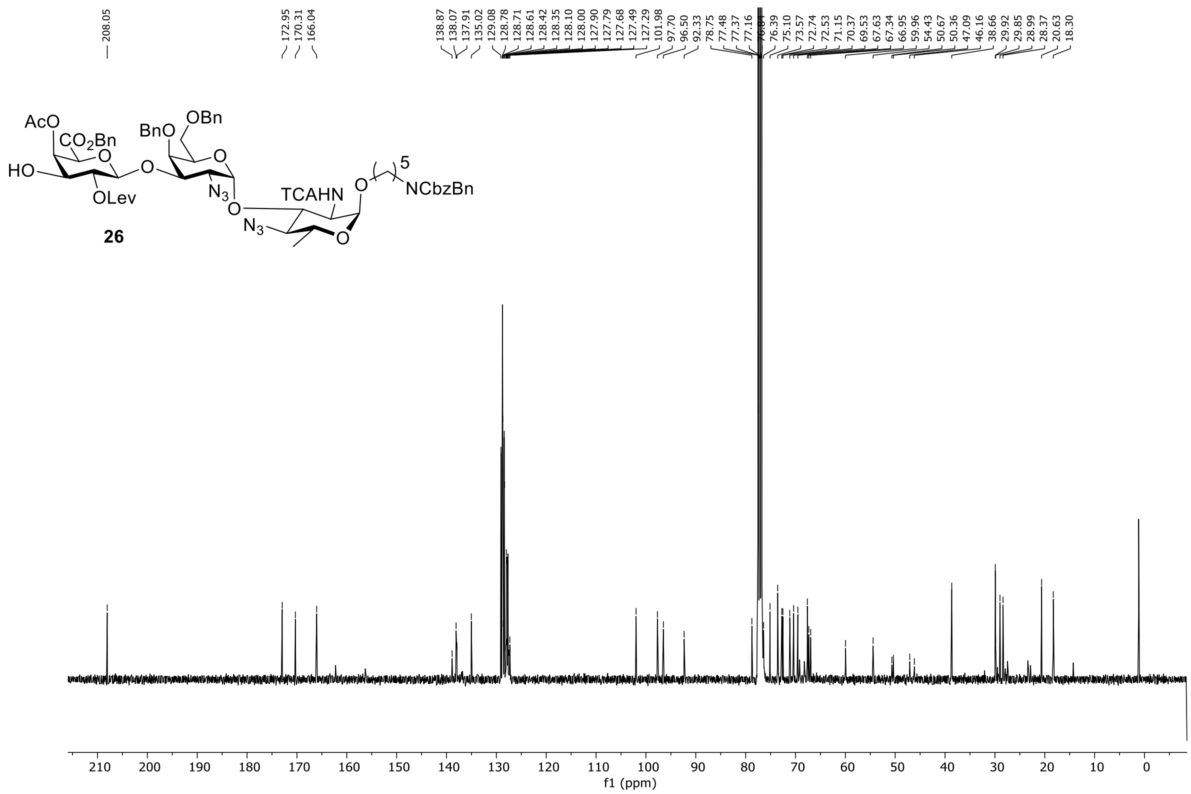

^{1}H-^{13}C HSQC NMR (400 MHz, $CDCl_3$)

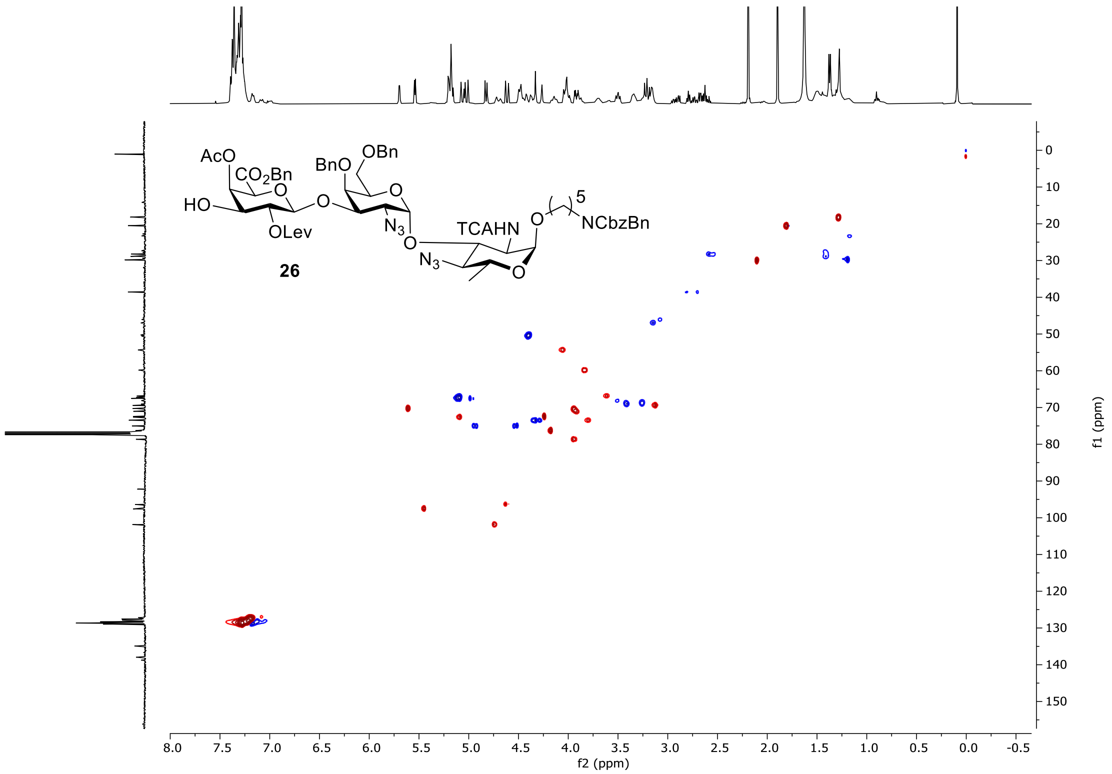

^{1}H-^{1}H COSY NMR (400 MHz, $CDCl_3$)

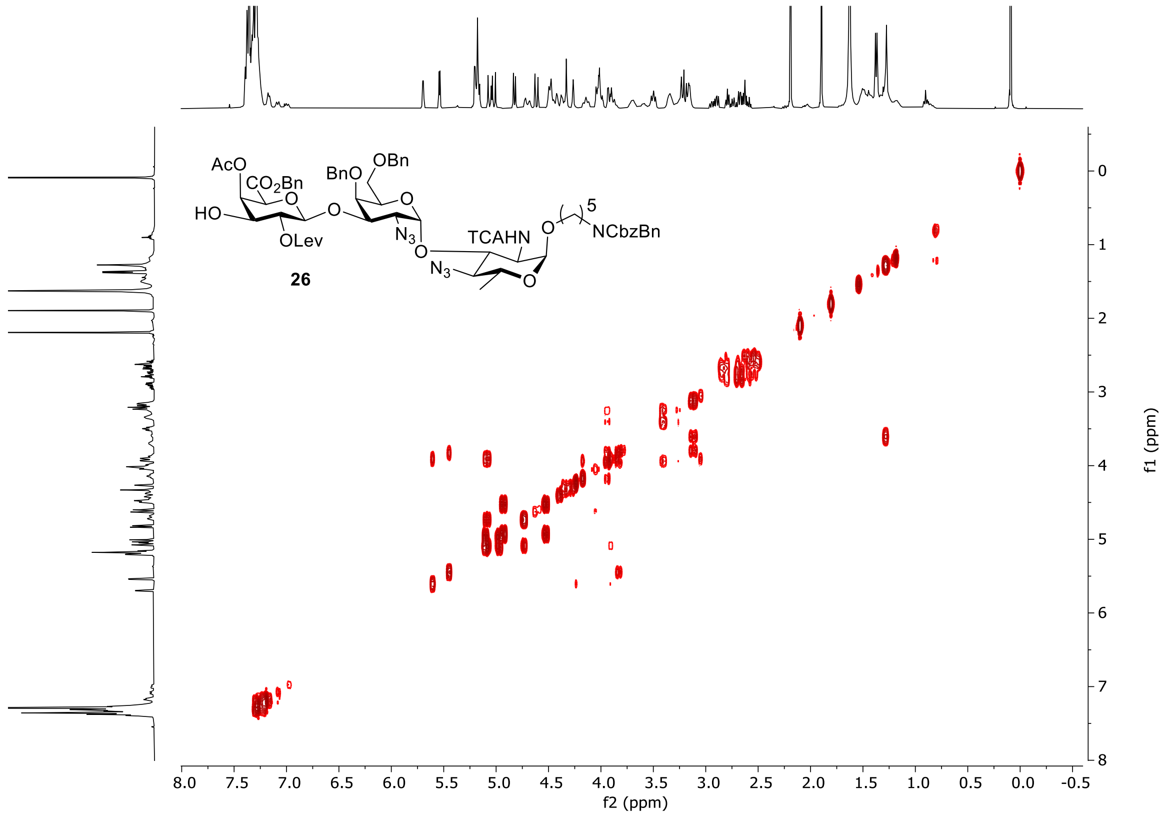

^{1}H NMR (600 MHz, $CDCl_3$)

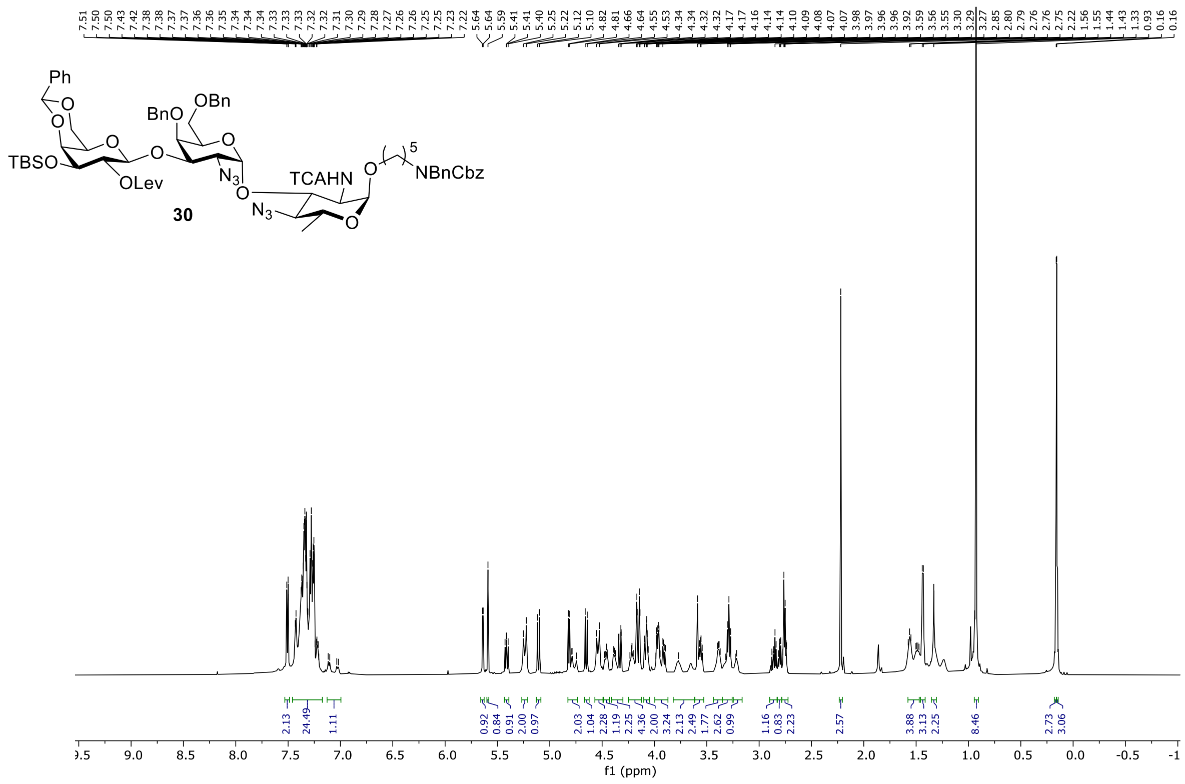

^{13}C NMR (151 MHz, $CDCl_3$)

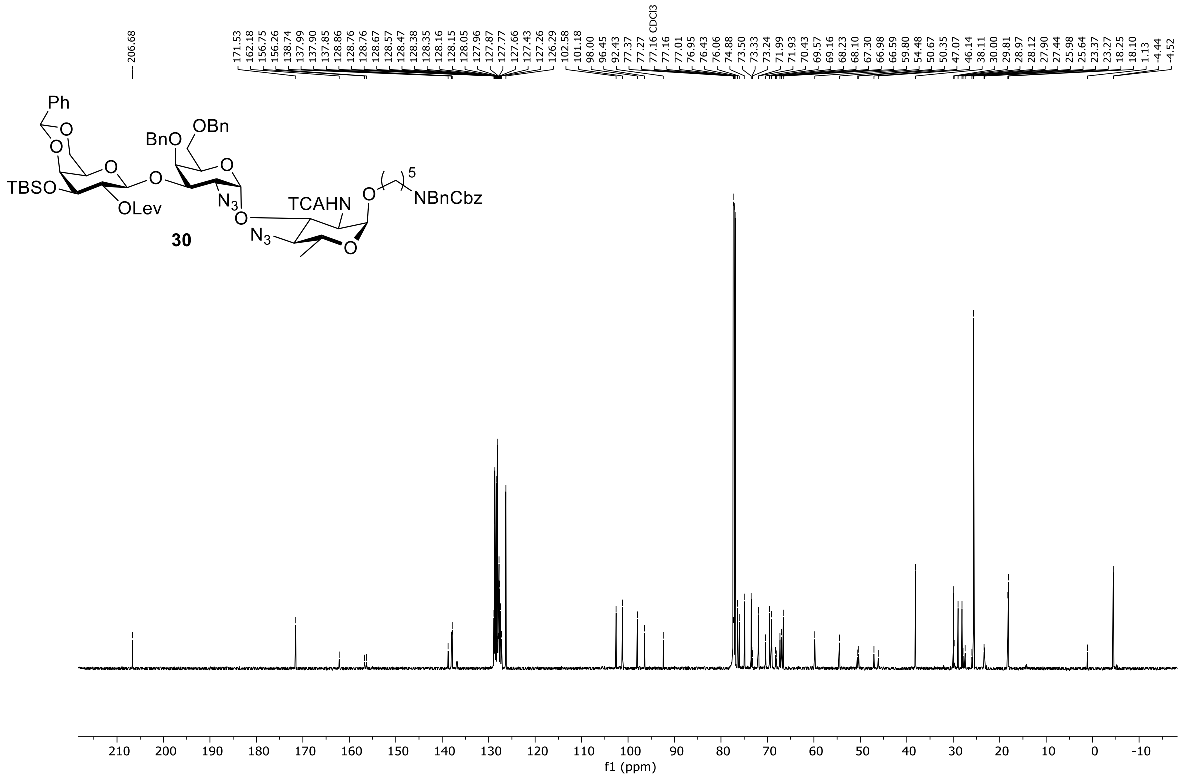

^{1}H-^{1}H COSY NMR (600 MHz, $CDCl_3$)

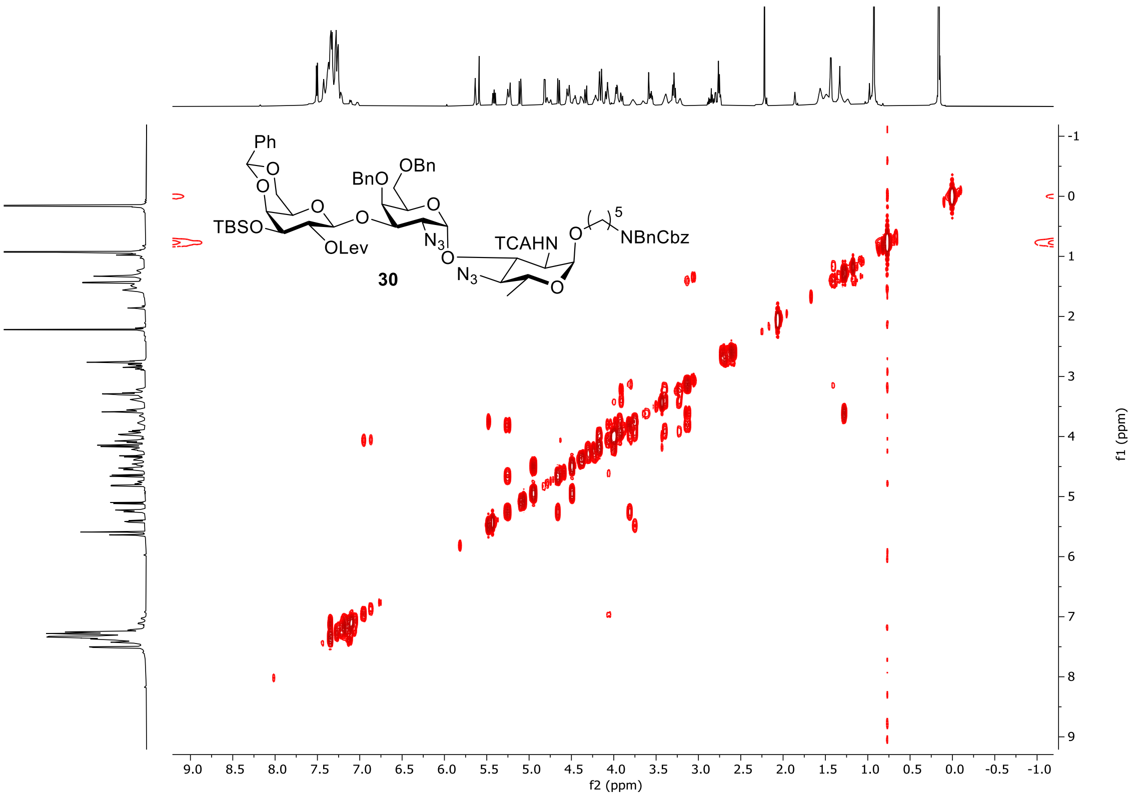

^{1}H-^{13}C HSQC NMR (600 MHz, $CDCl_3$)

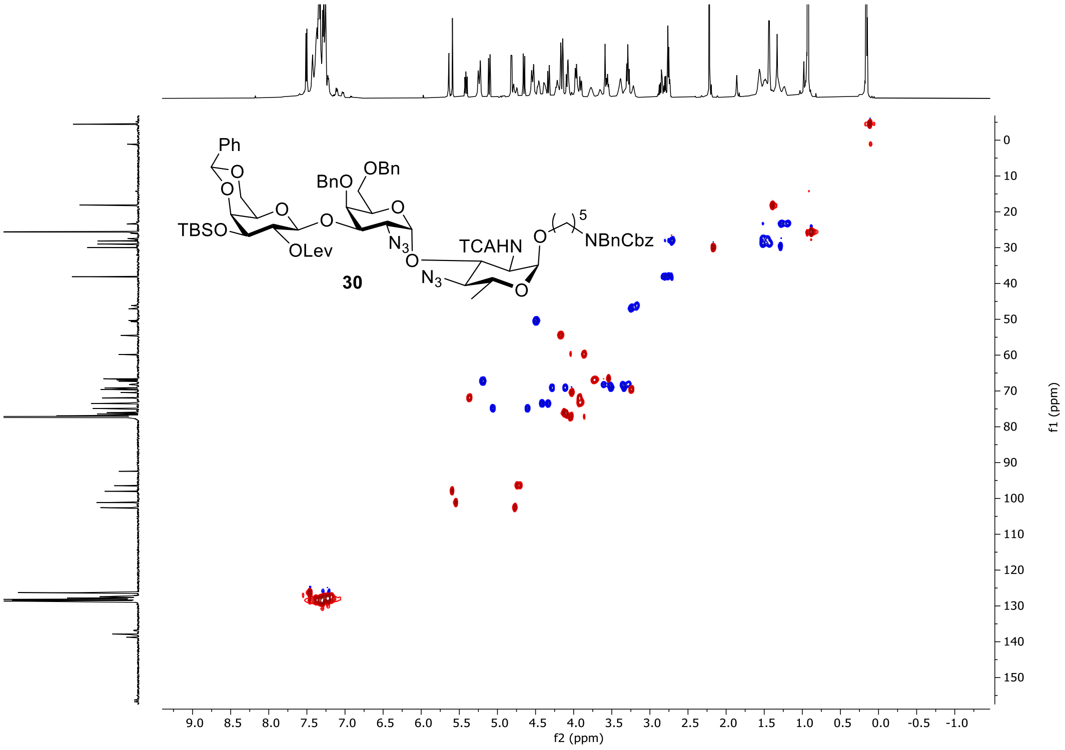

^{1}H-^{1}H Coupled HSQC NMR (600 MHz, $CDCl_3$)

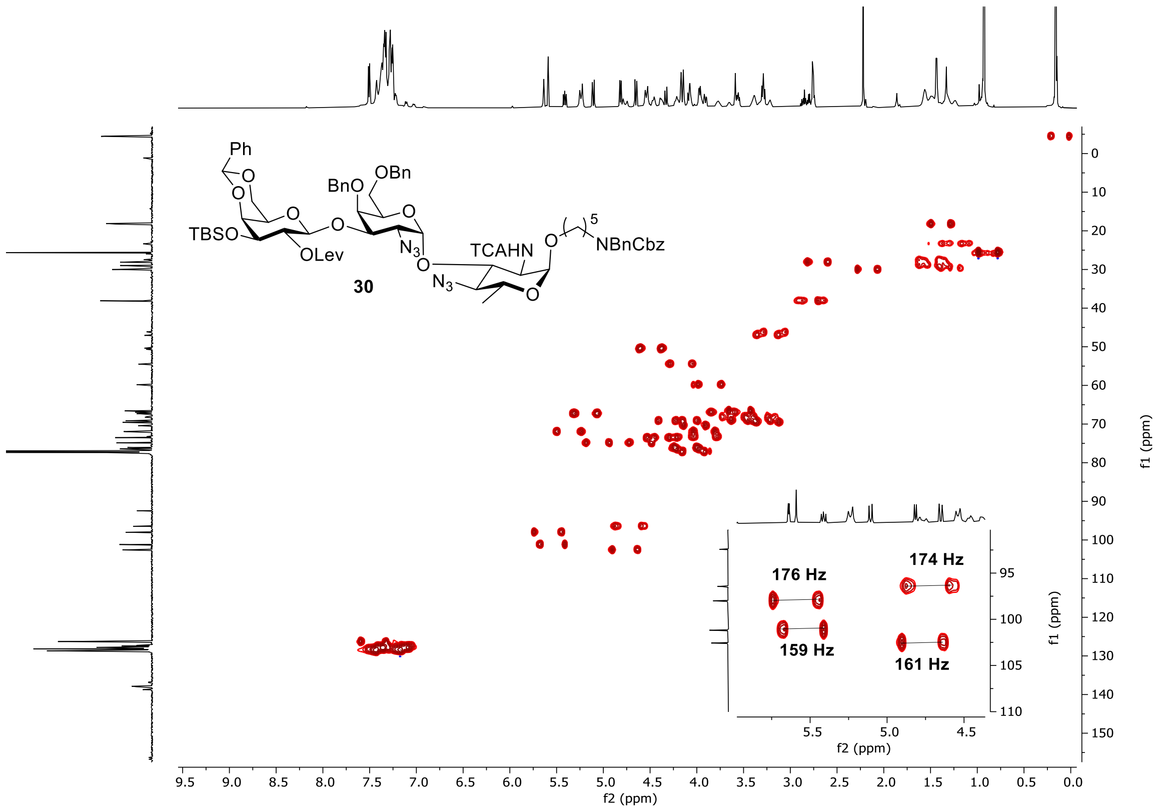

^{1}H NMR (400 MHz, $CDCl_3$)

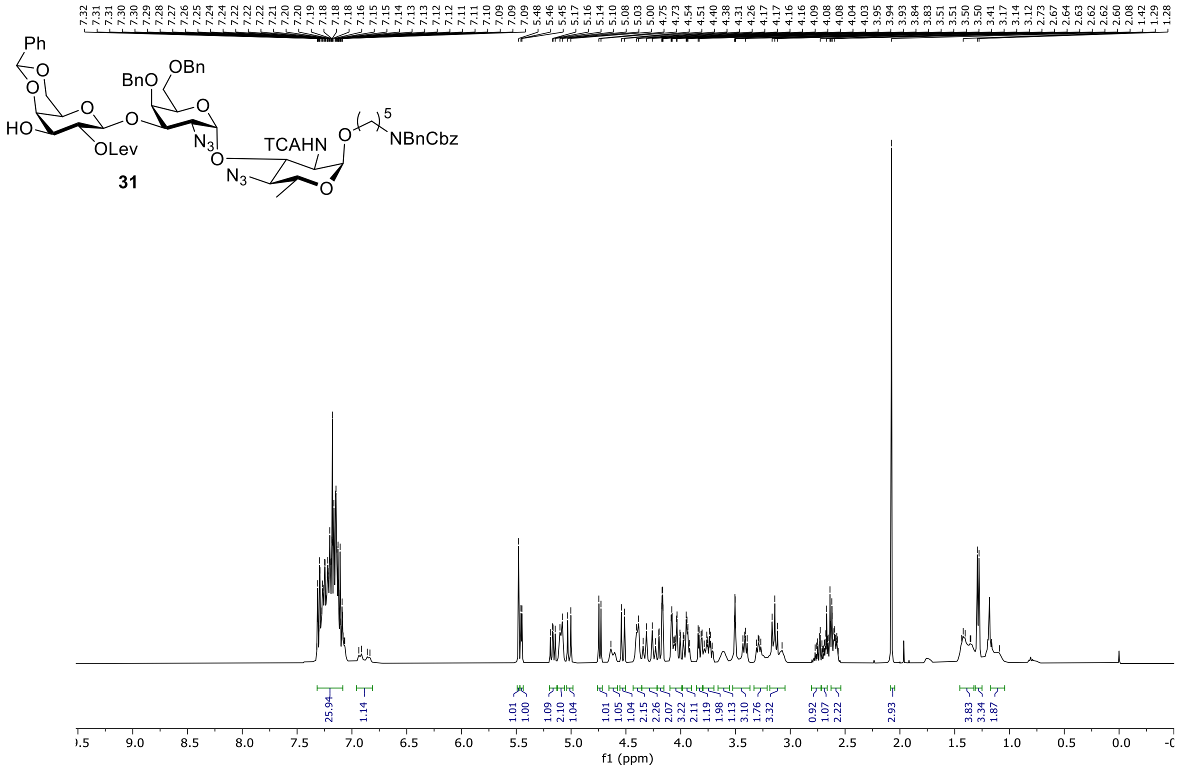

^{13}C NMR (101 MHz, $CDCl_3$)

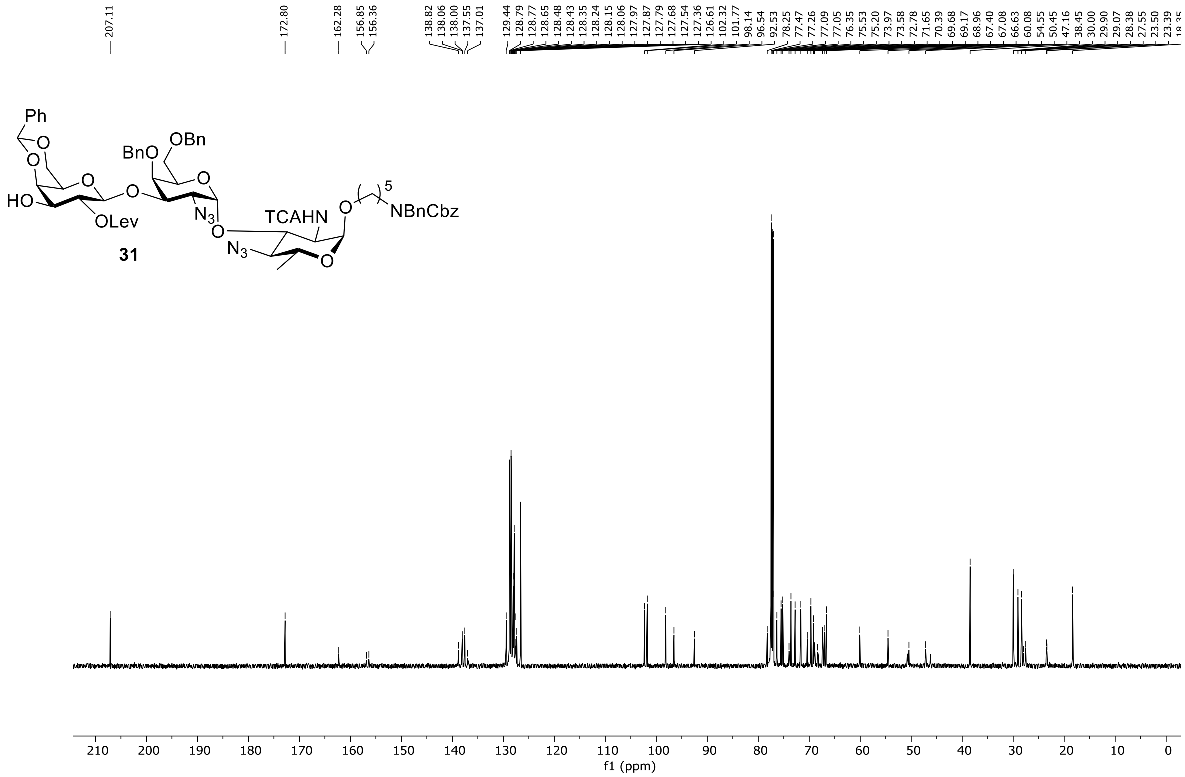

^1H-^1H COSY NMR (400 MHz, $CDCl_3$)

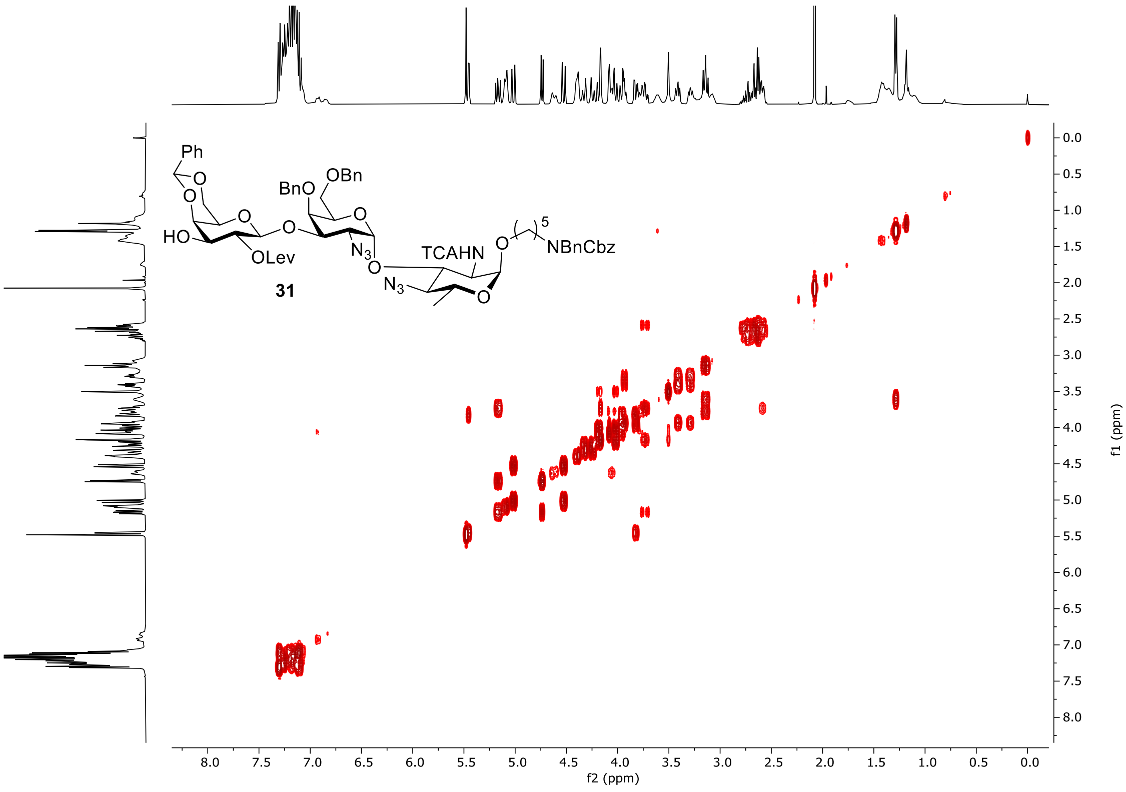

^{1}H-^{13}C HSQC NMR (600 MHz, $CDCl_3$)

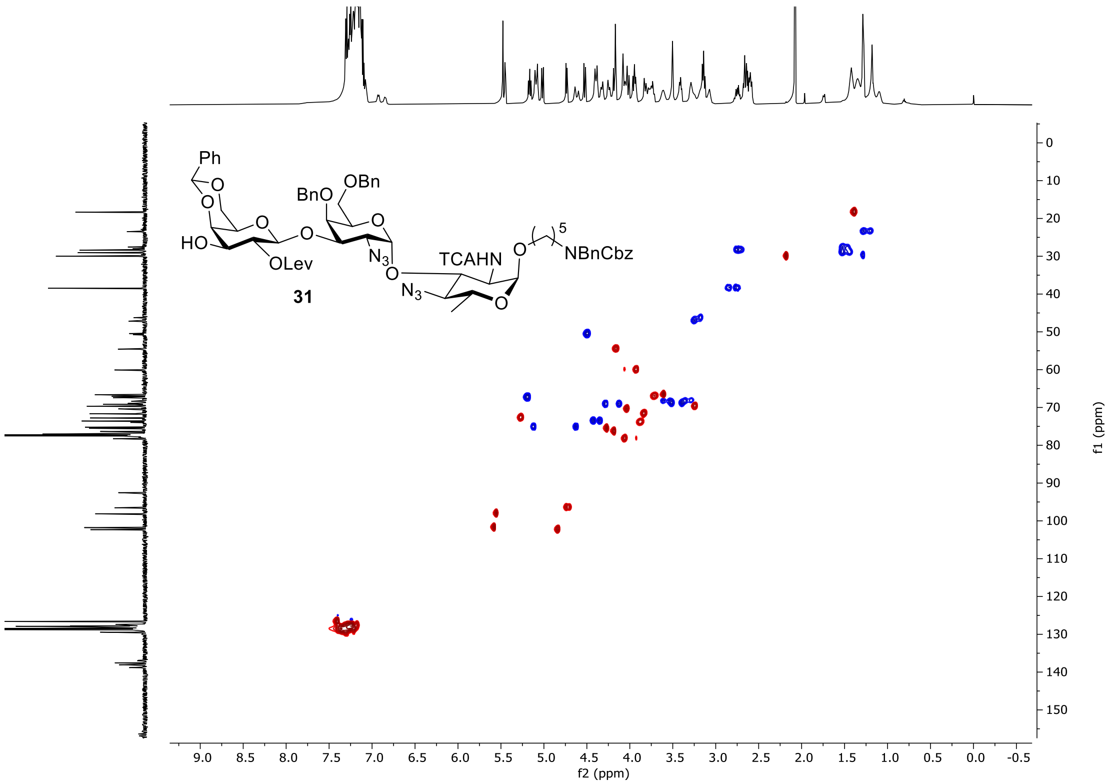

^{1}H-^{13}C Coupled HSQC NMR (600 MHz, $CDCl_3$)

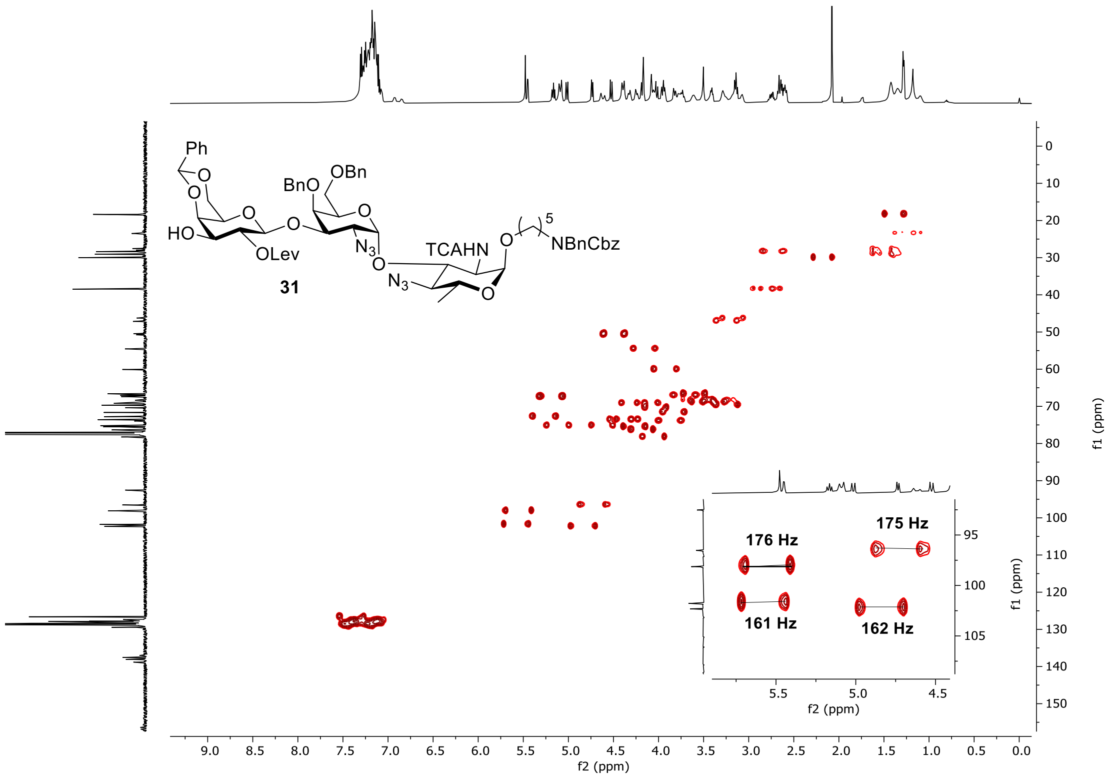

^{1}H NMR (600 MHz, $CDCl_3$)

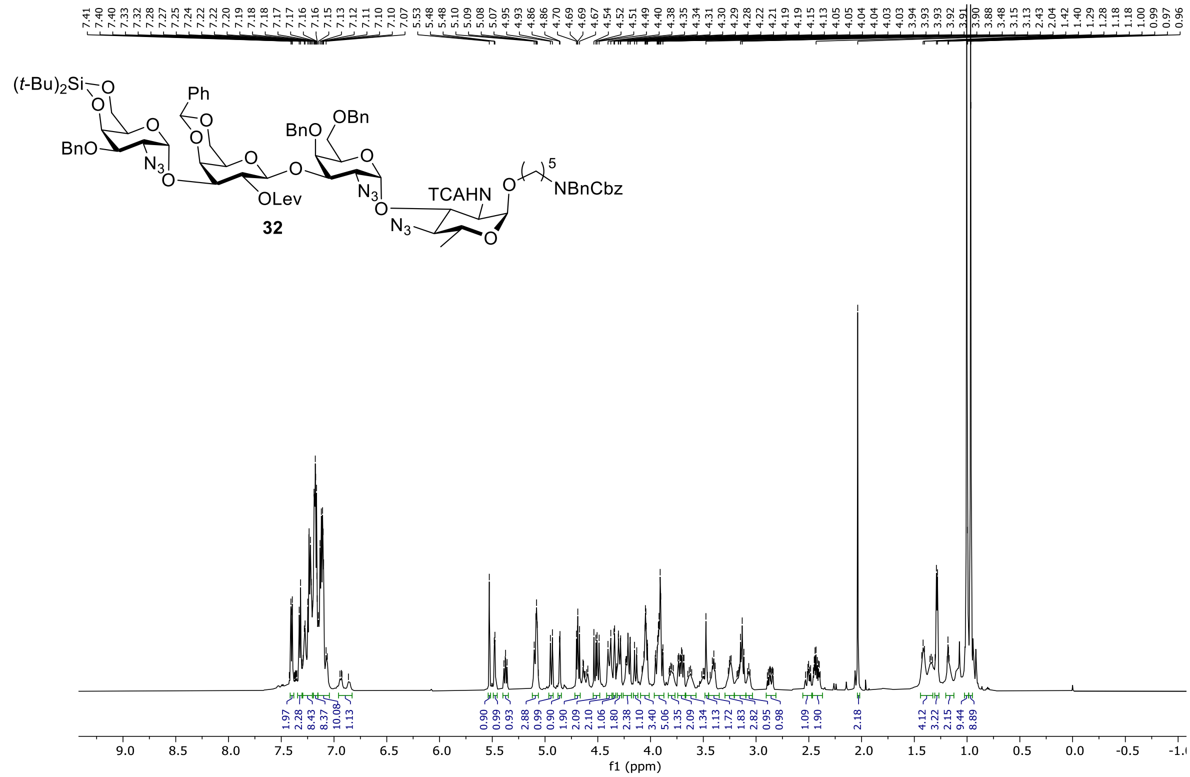

^{13}C NMR (151 MHz, $CDCl_3$)

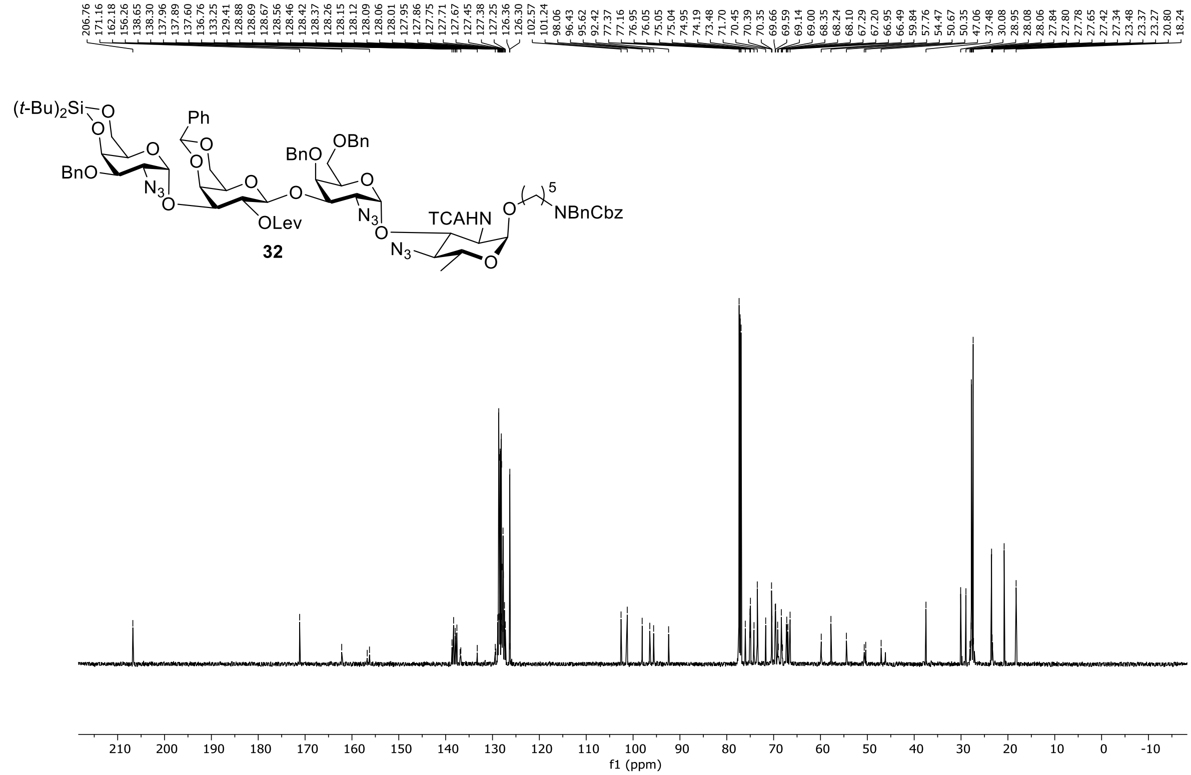

^{1}H-^{1}H COSY NMR (600 MHz, $CDCl_3$)

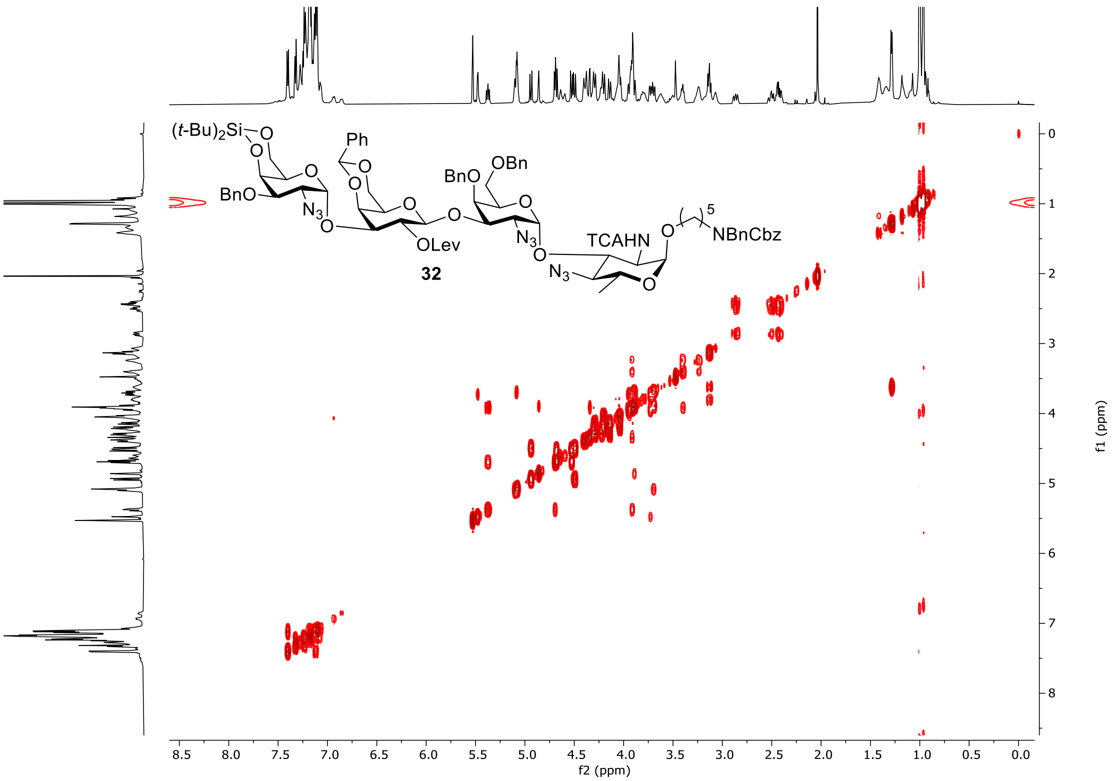

^{1}H-^{13}C HSQC NMR (600 MHz, $CDCl_3$)

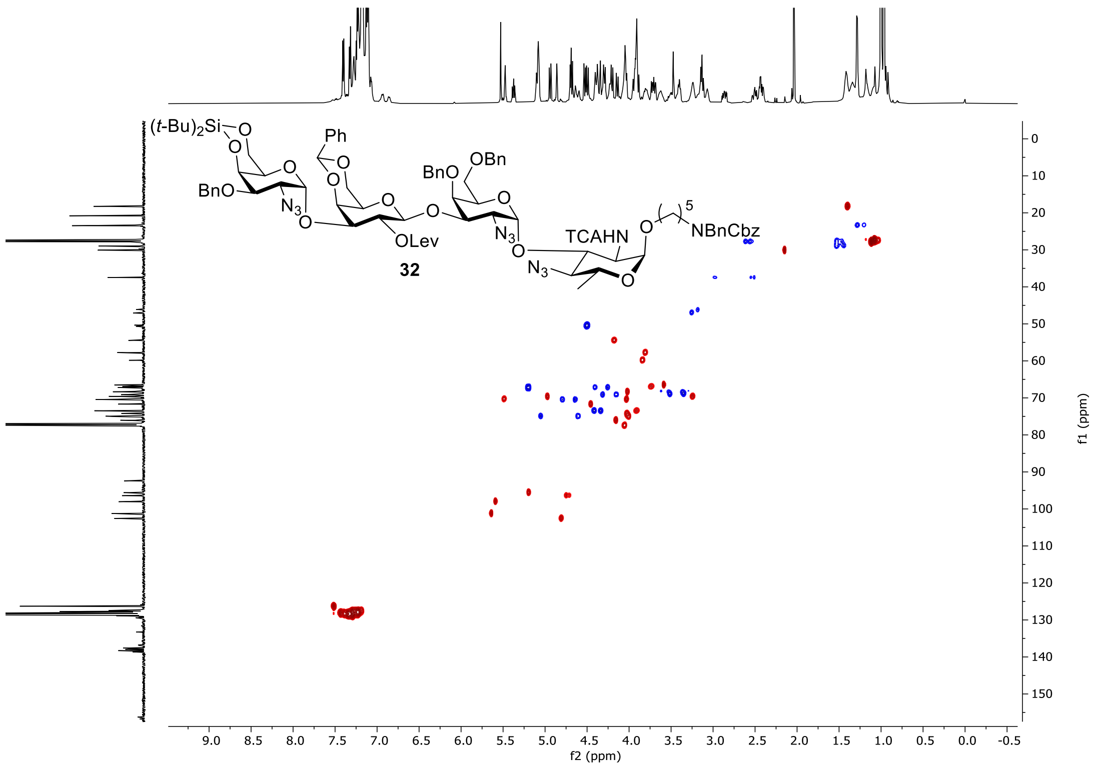

^1H-^1H Coupled HSQC NMR (600 MHz, $CDCl_3$)

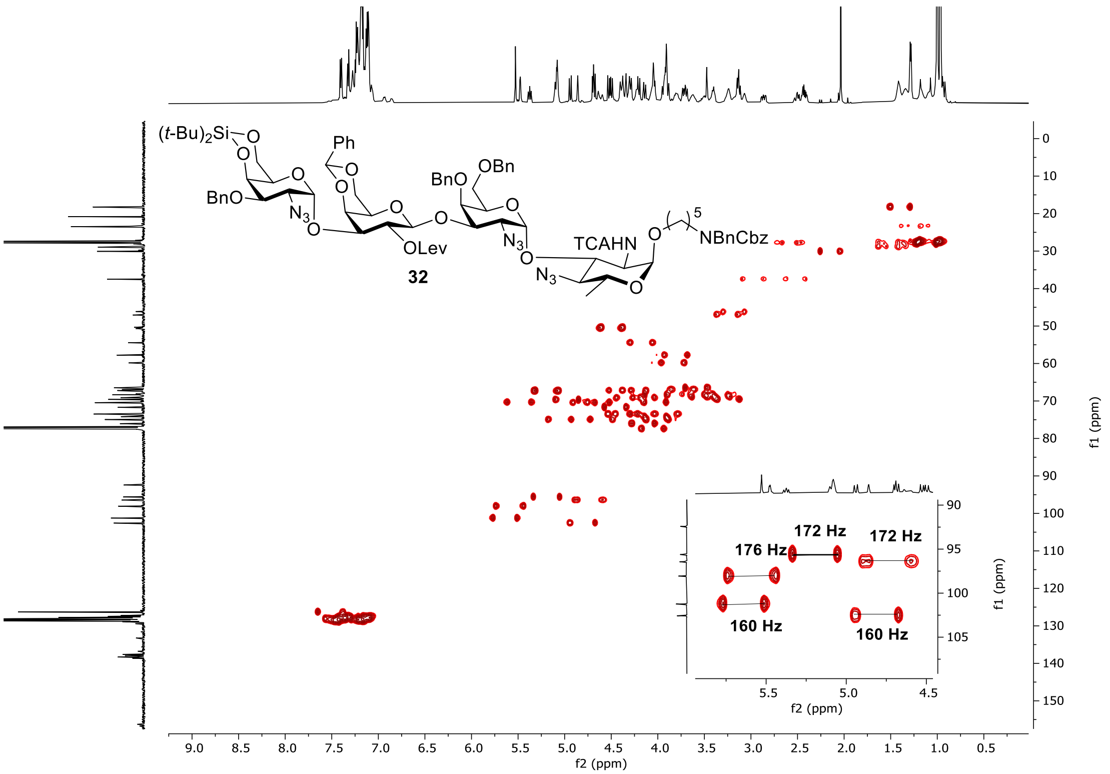

^1H NMR (600 MHz, $CDCl_3$)

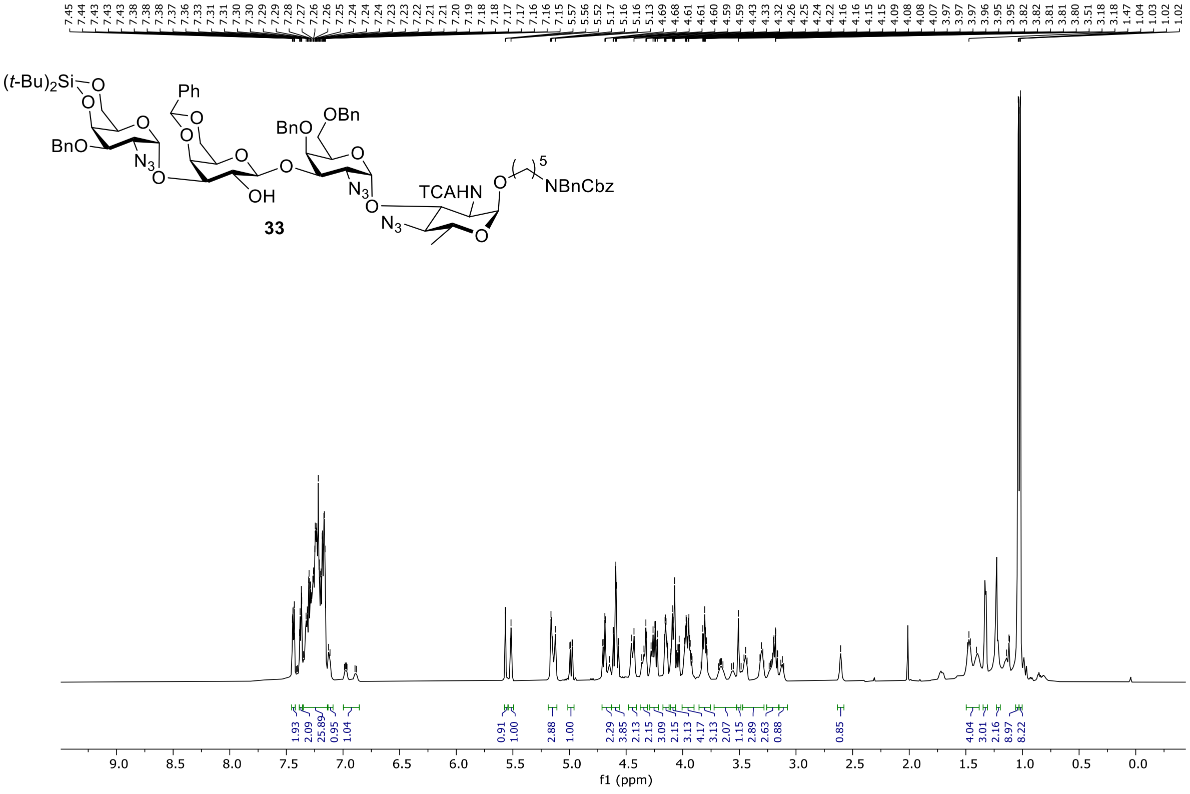

^{13}C NMR (151 MHz, CDCl$_3$)

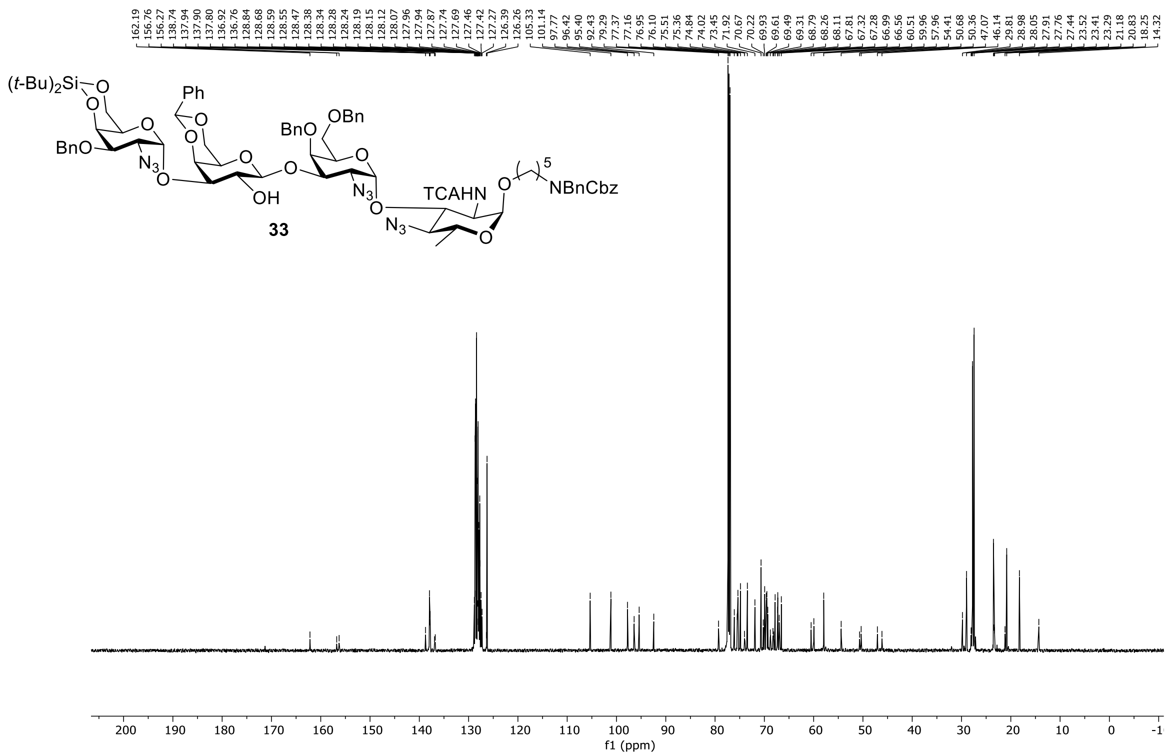

^{1}H-^{1}H COSY NMR (600 MHz, CDCl$_3$)

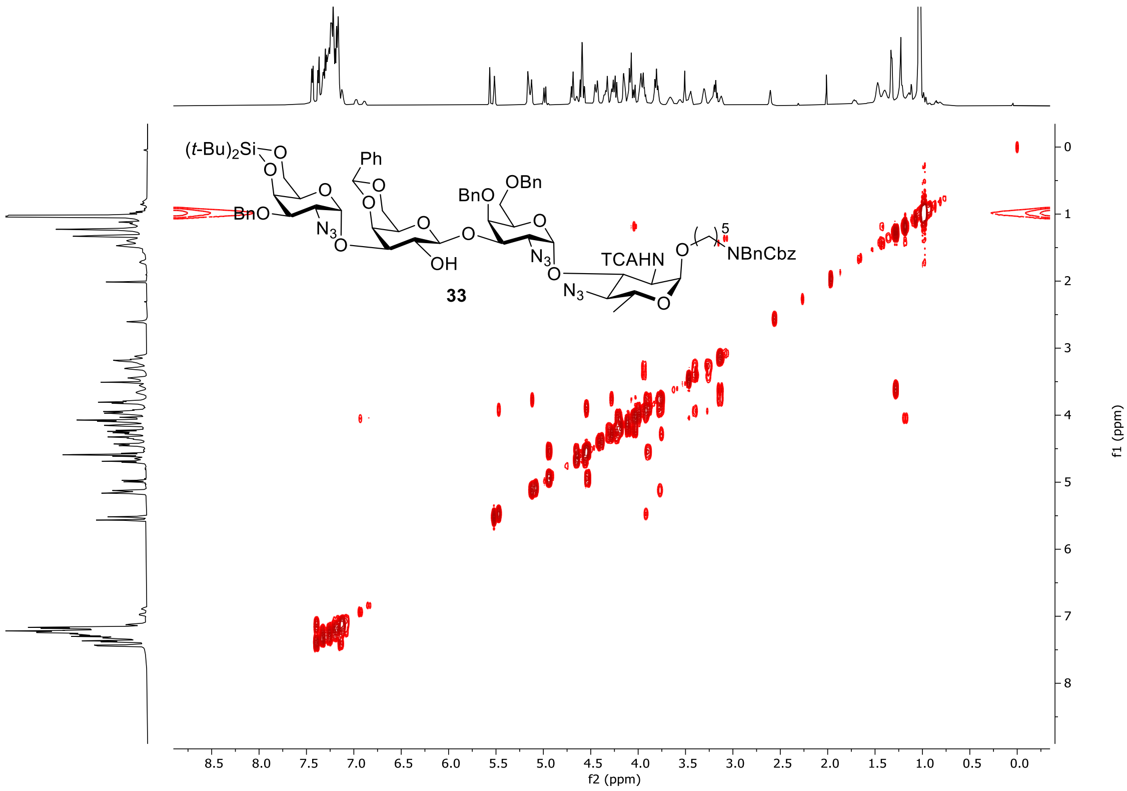

^{1}H-^{13}C HSQC NMR (600 MHz, $CDCl_3$)

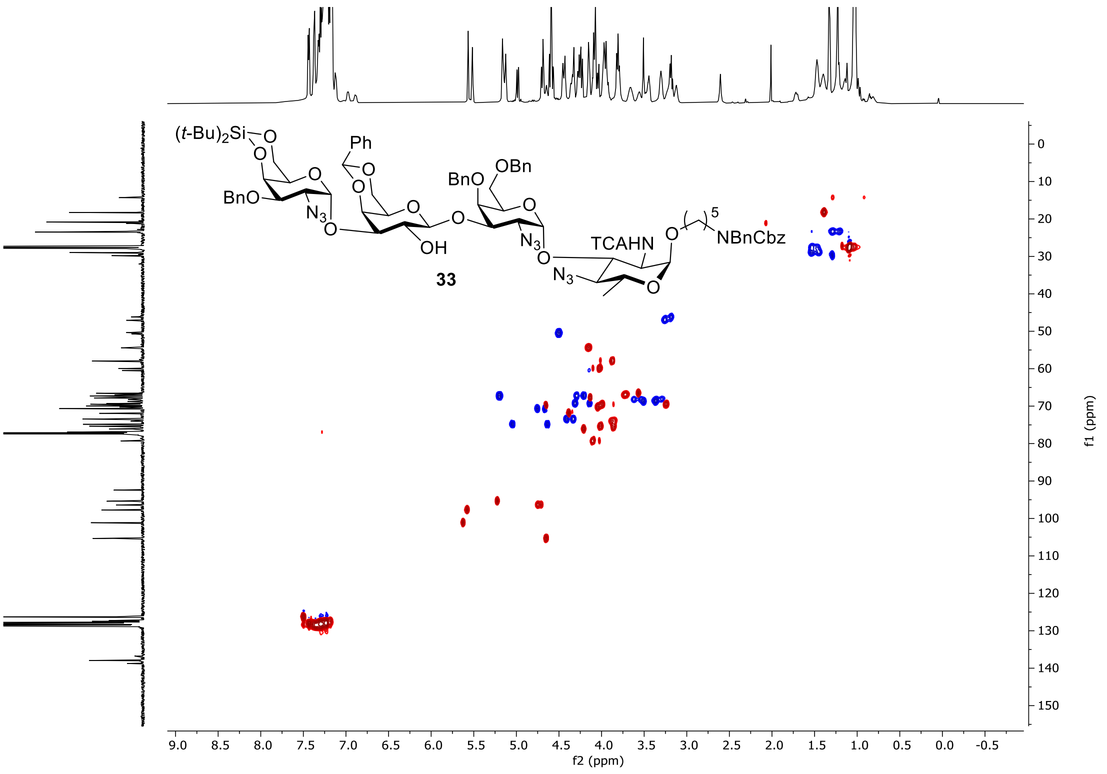

^{1}H-^{13}C Coupled HSQC NMR (600 MHz, $CDCl_3$)

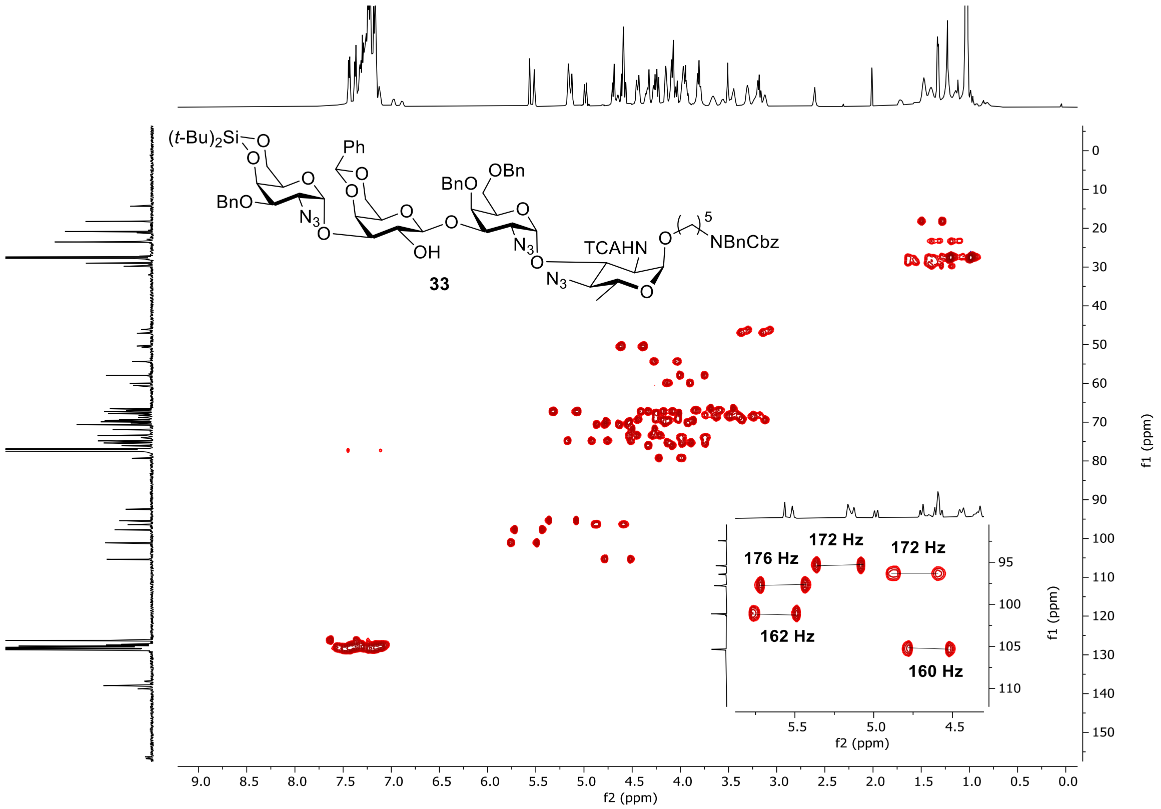

^{1}H NMR (600 MHz, $CDCl_3$)

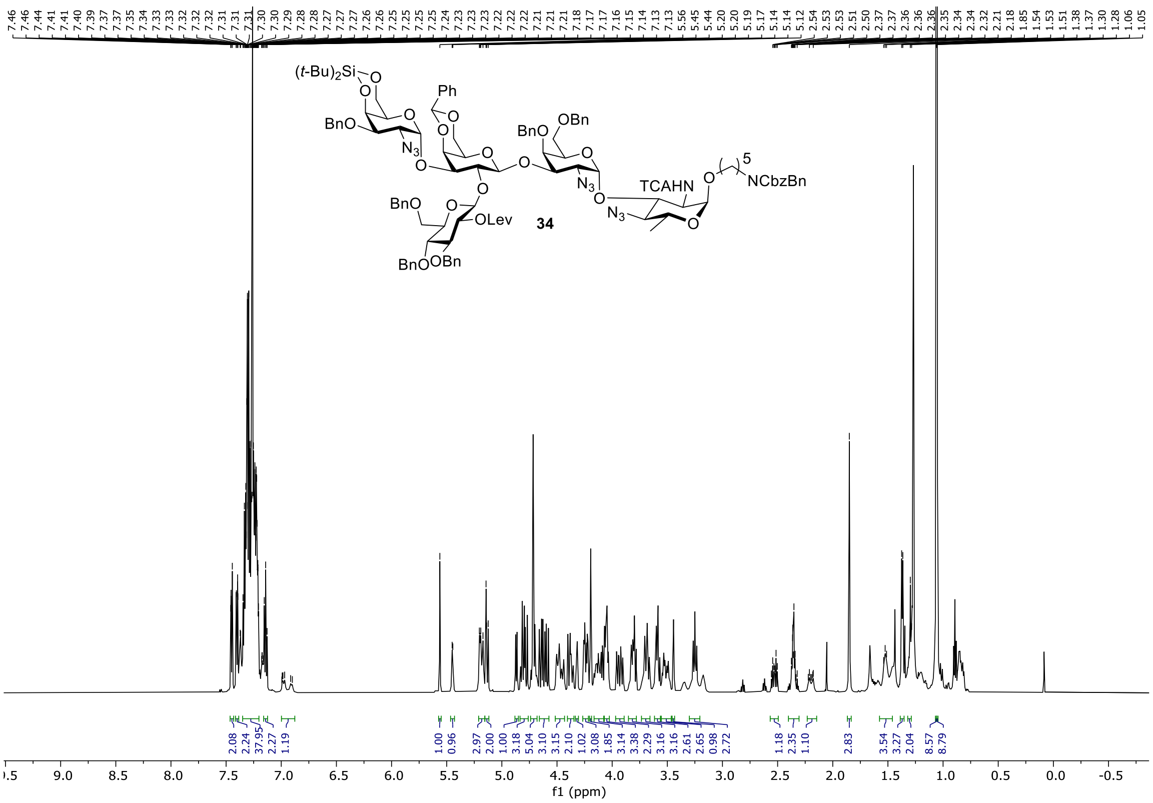

^{1}H-^{13}C NMR (151 MHz, $CDCl_3$)

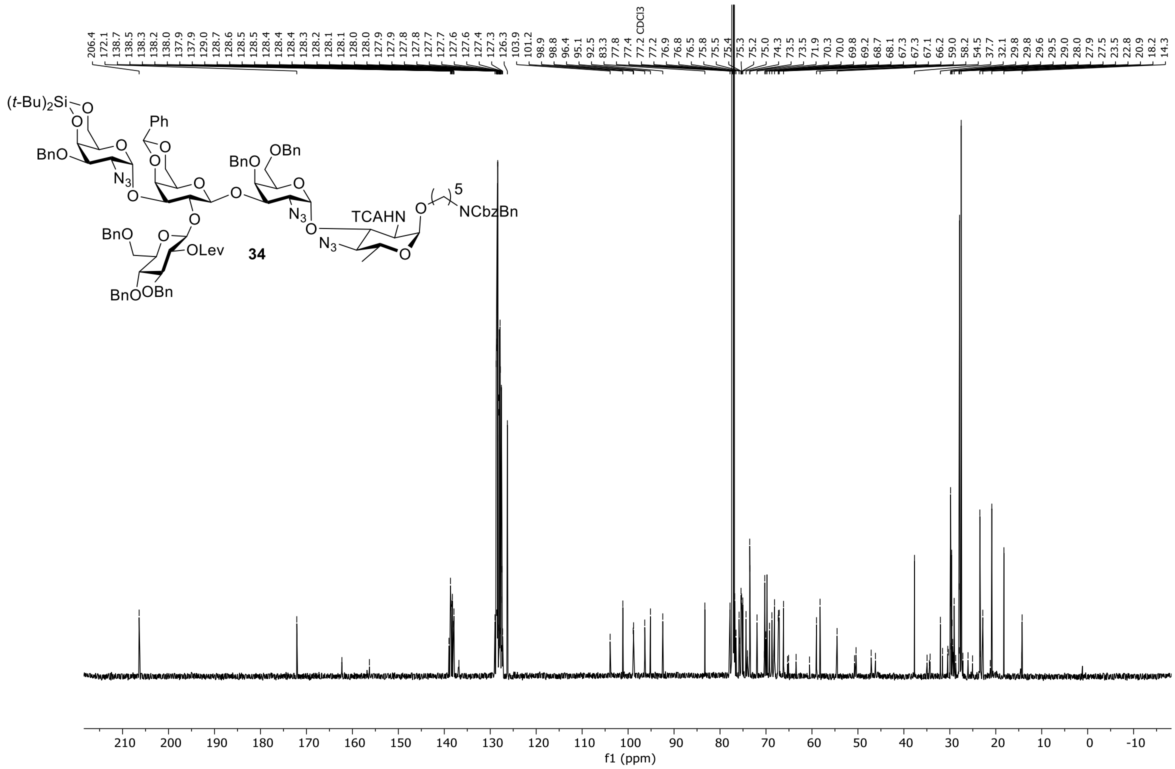

^{1}H-^{1}H COSY NMR (400 MHz, $CDCl_3$)

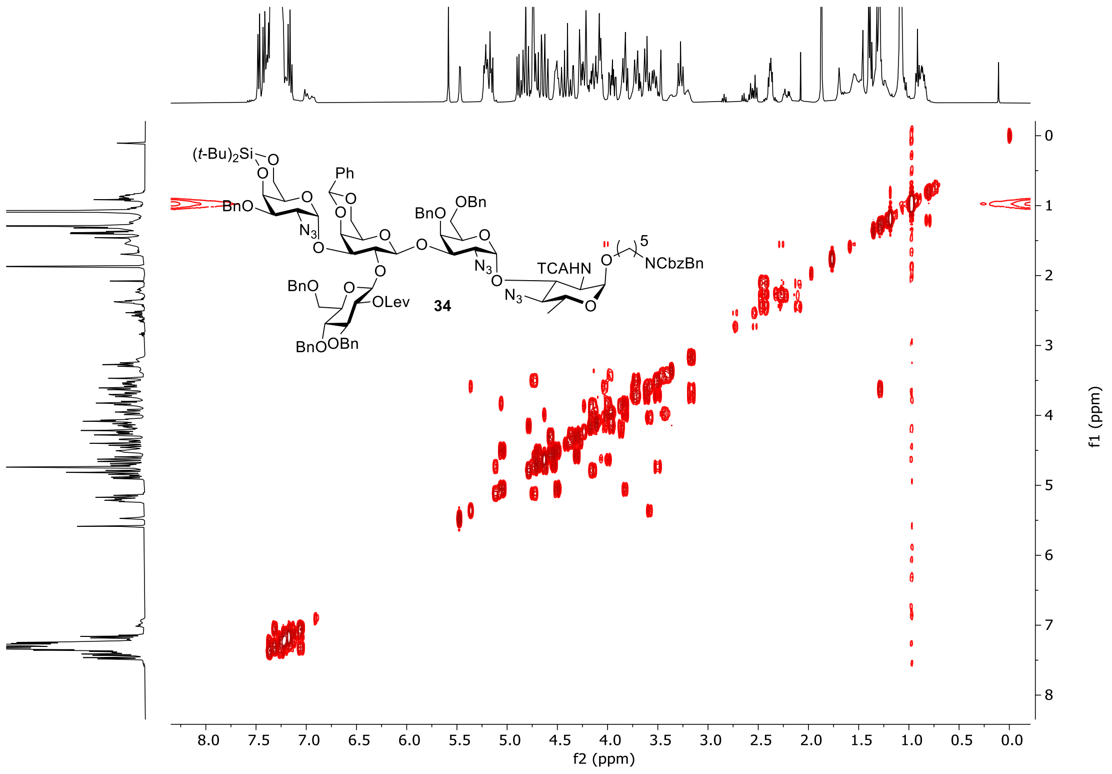

^{1}H-^{13}C HSQC NMR (600 MHz, $CDCl_3$)

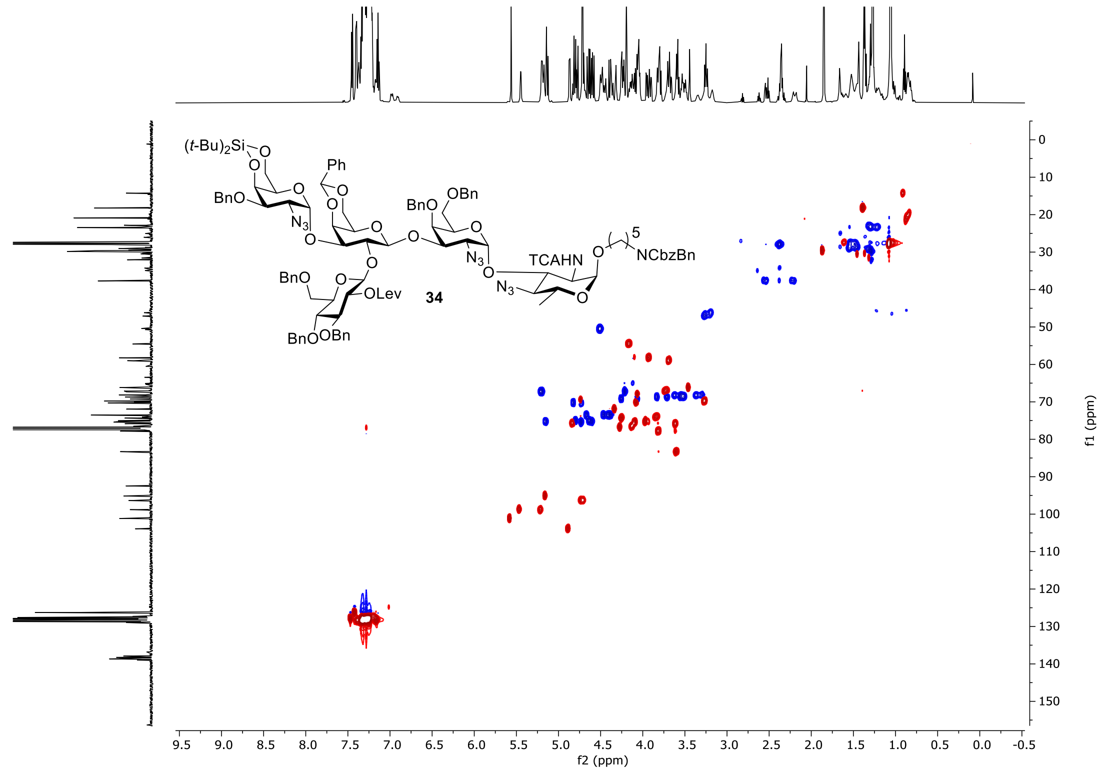

^{1}H-^{13}C Coupled HSQC NMR (600 MHz, $CDCl_3$)

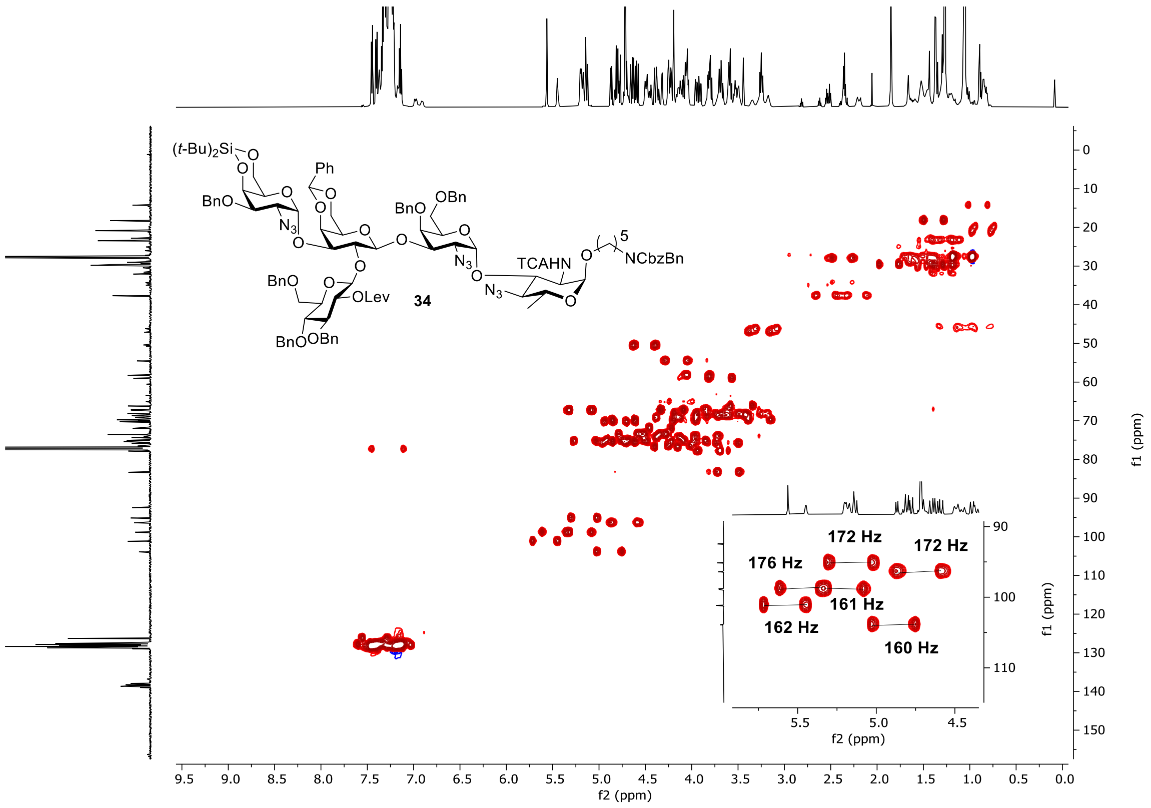

^{1}H NMR (600 MHz, $CDCl_3$)

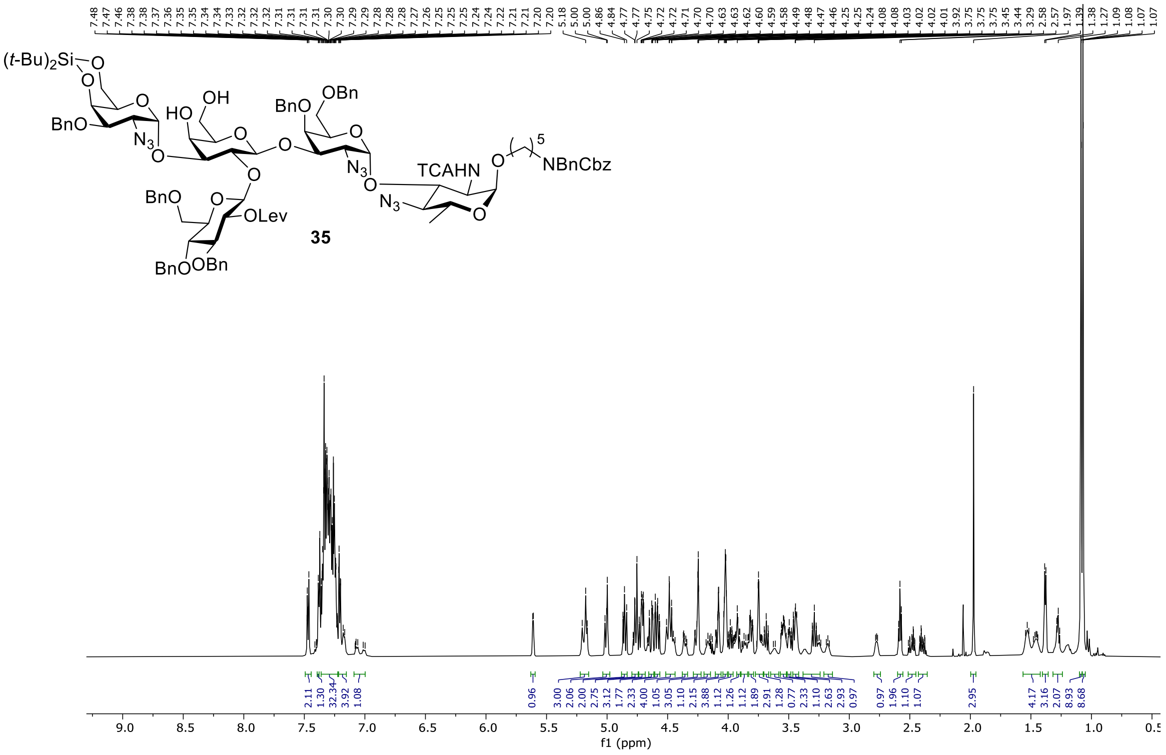

^{13}C NMR (151 MHz, $CDCl_3$)

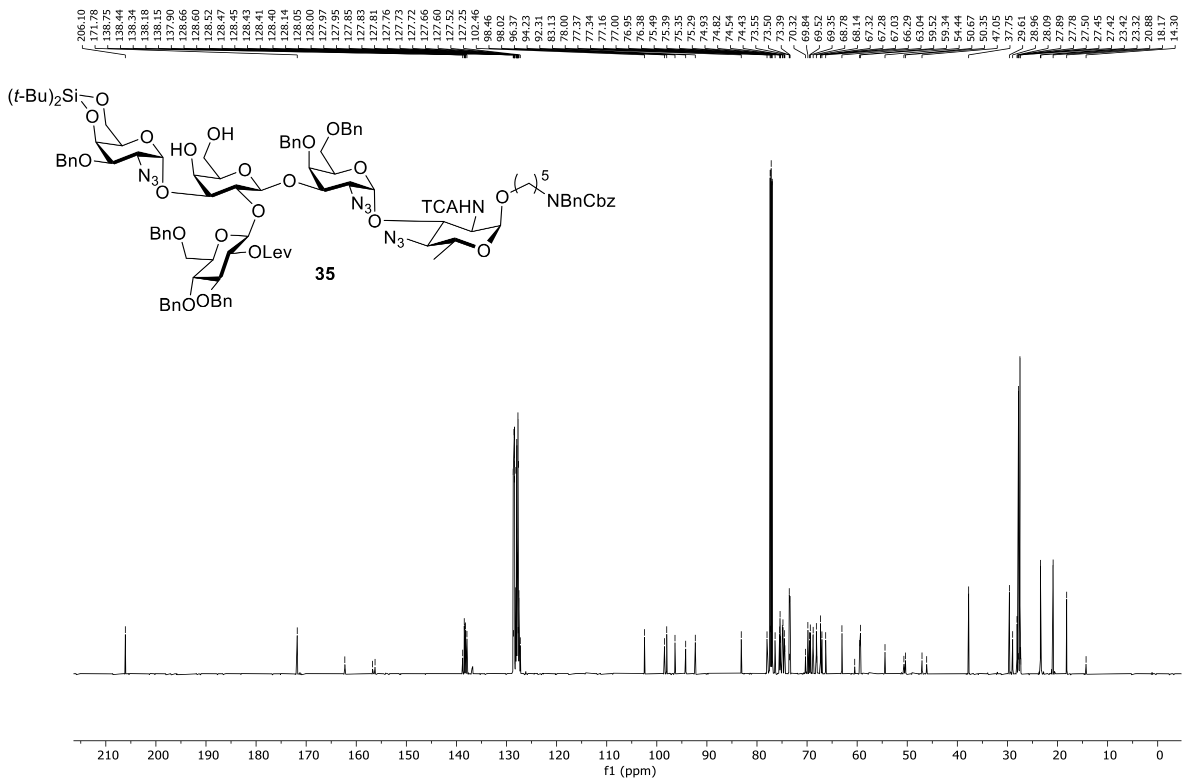

^{1}H-^{1}H COSY NMR (600 MHz, $CDCl_3$)

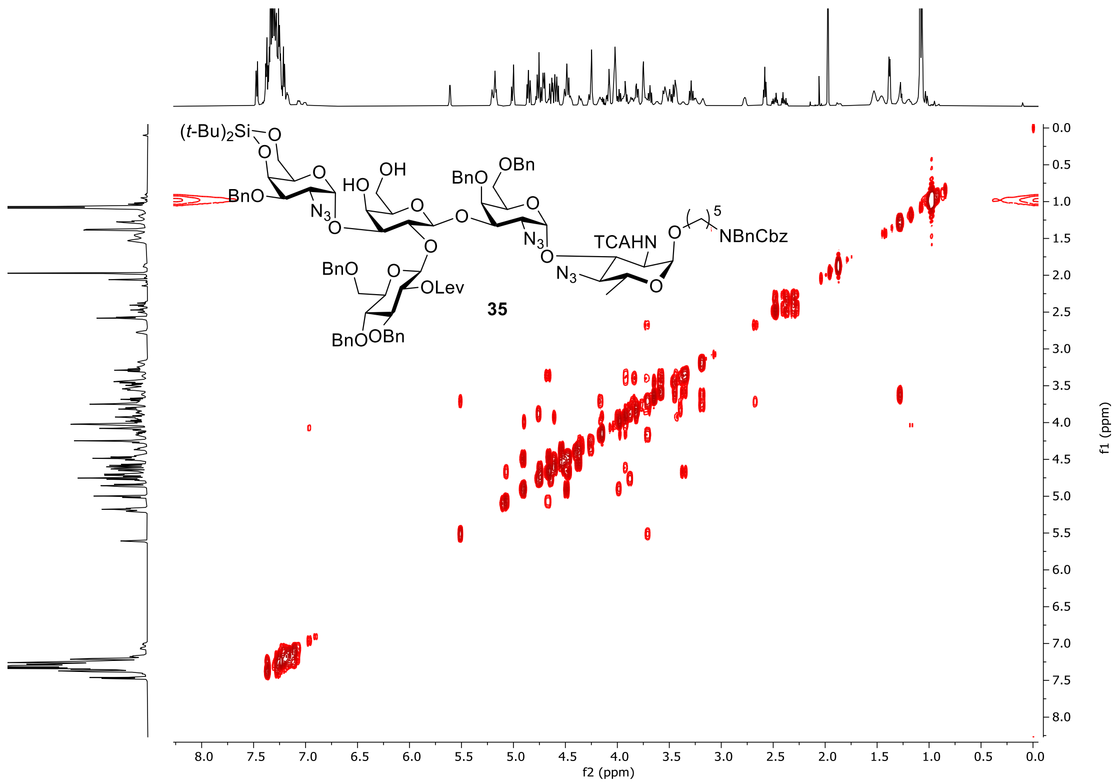

^{1}H-^{13}C HSQC NMR (600 MHz, $CDCl_3$)

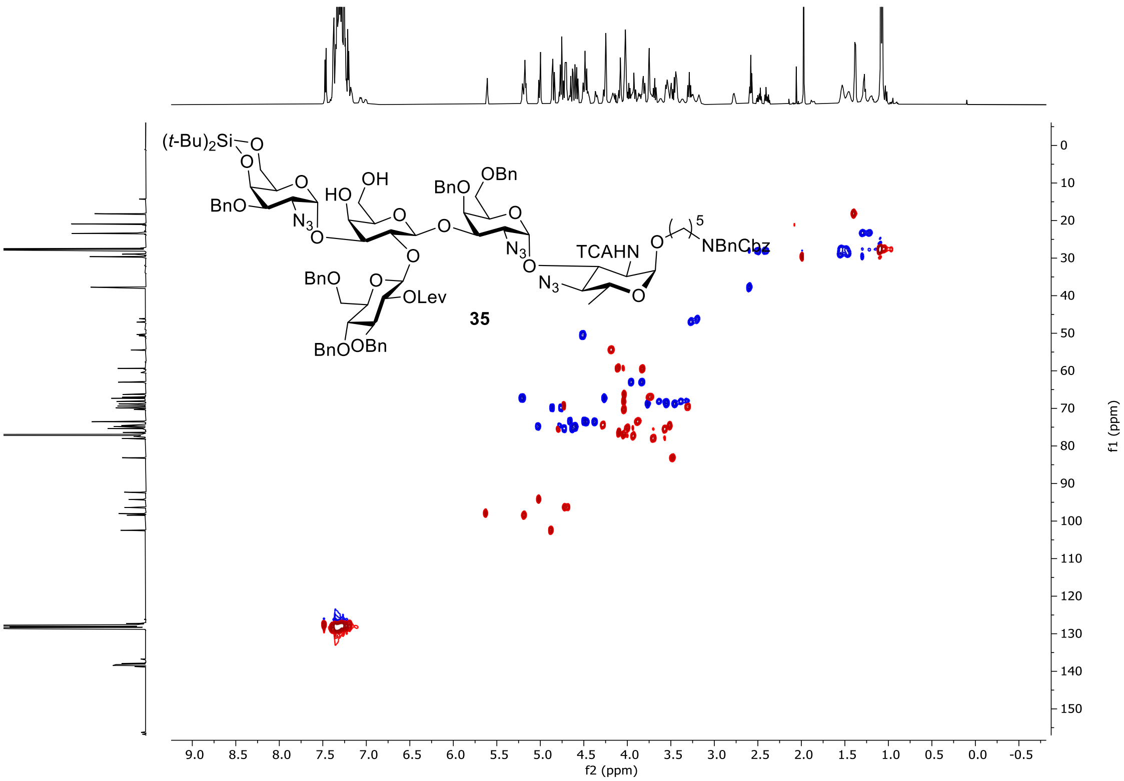

^{1}H-^{13}C Coupled HSQC NMR (600 MHz, $CDCl_3$)

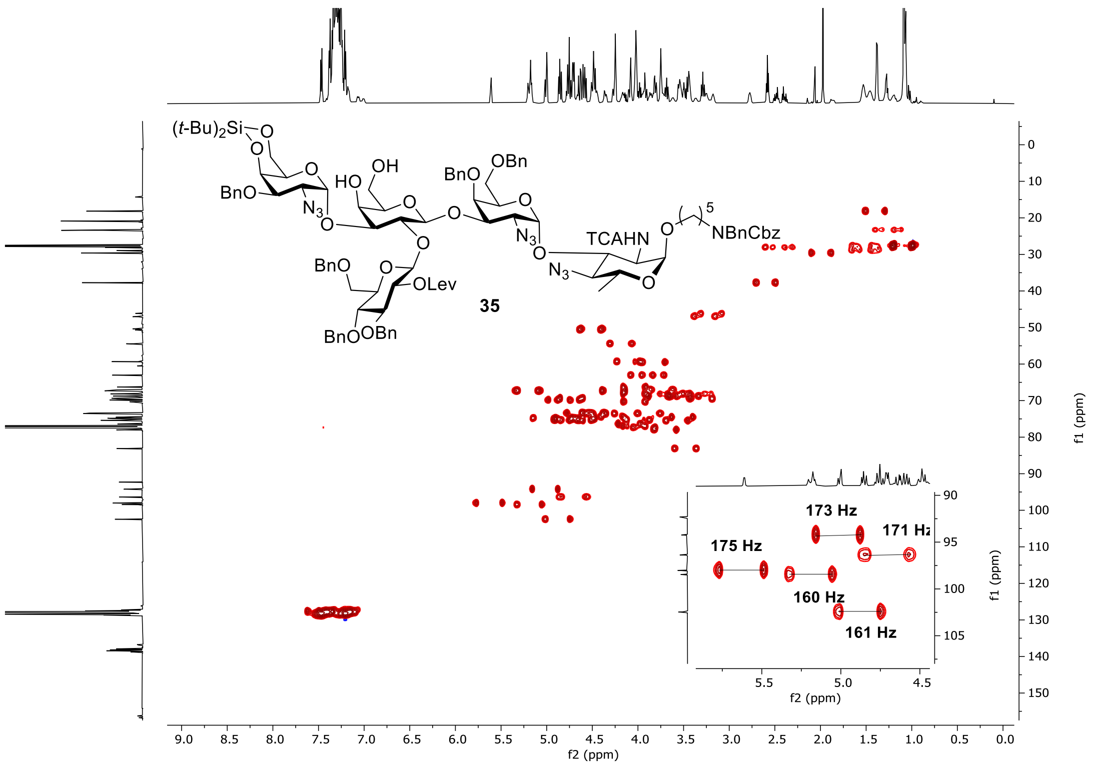

^{1}H NMR (400 MHz, CDCl$_3$)

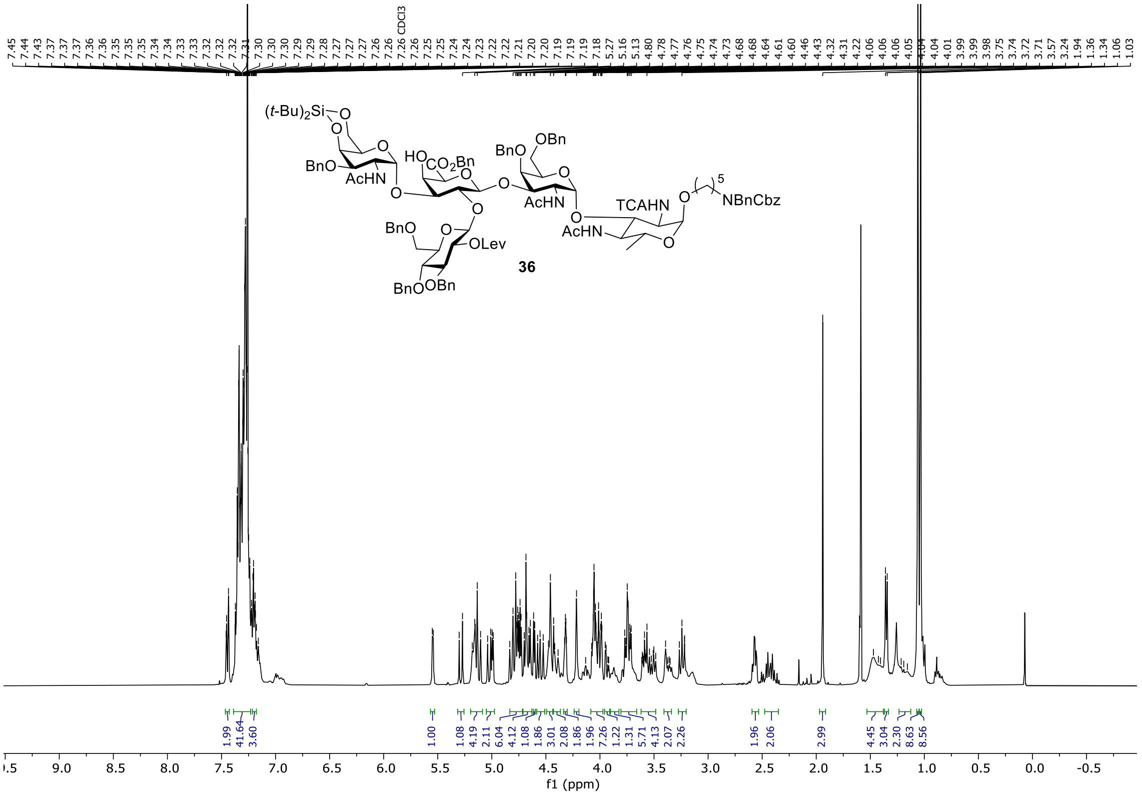

^{13}C NMR (101 MHz, CDCl$_3$)

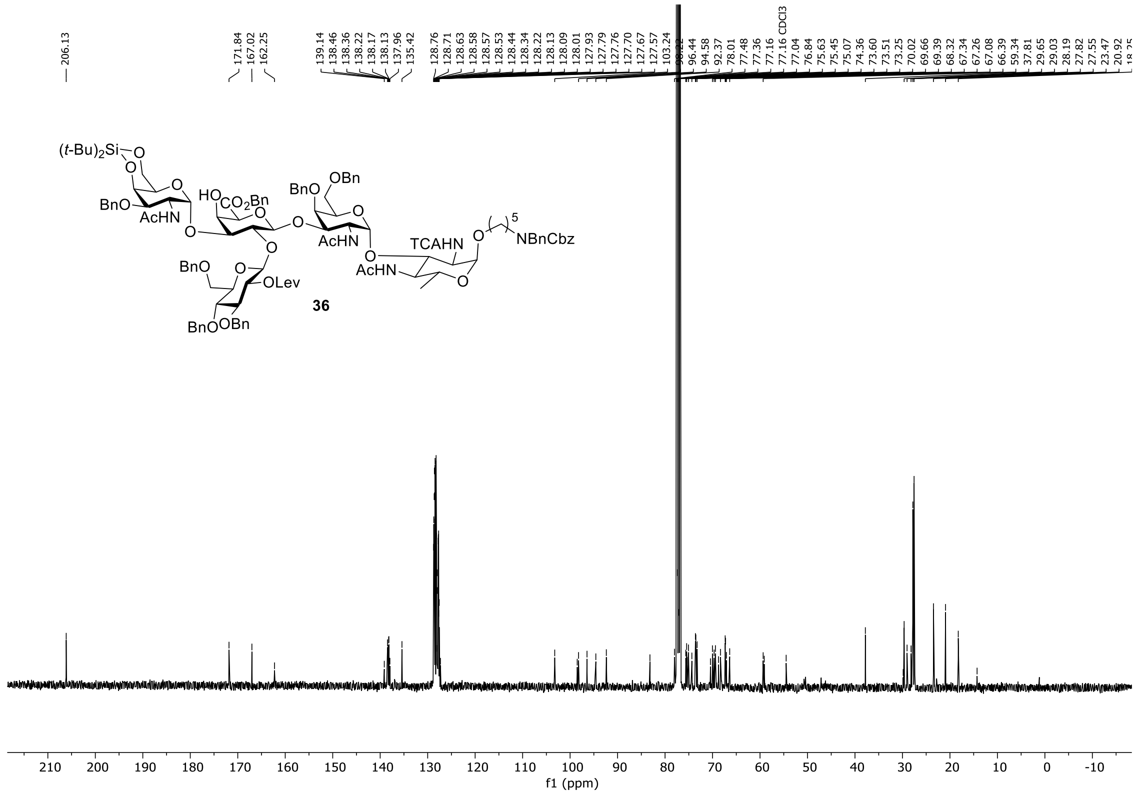

^{1}H-^{1}H COSY NMR (400 MHz, $CDCl_3$)

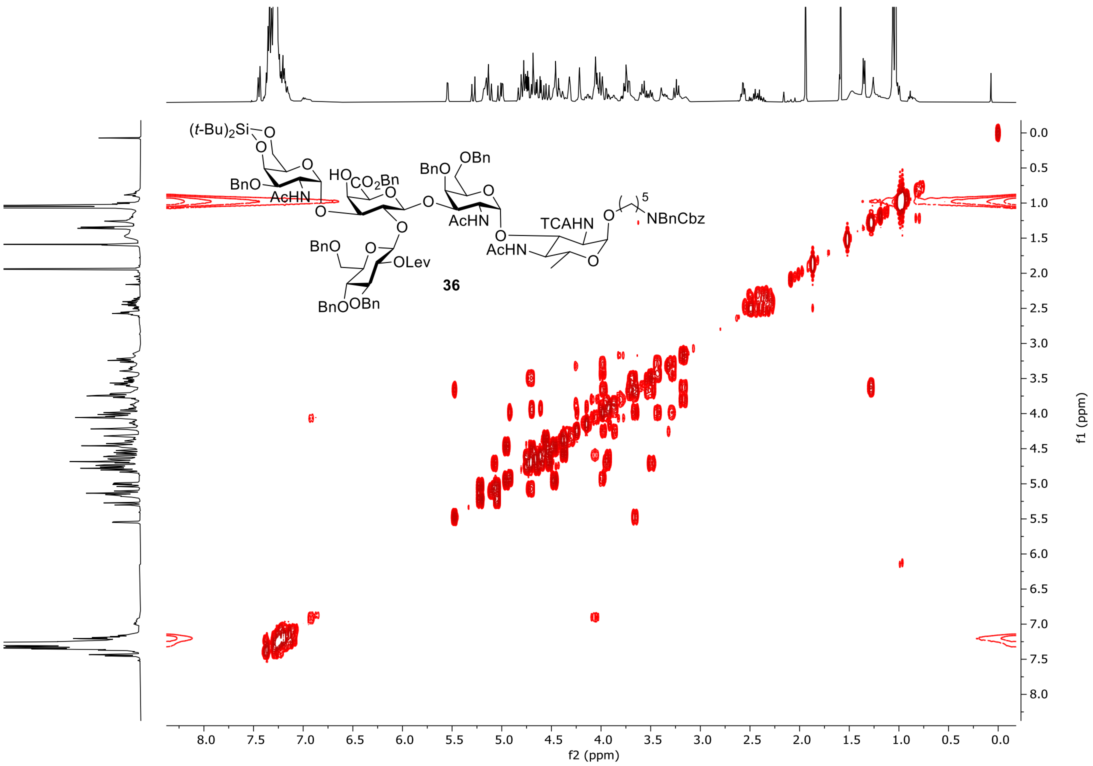

^{1}H-^{13}C HSQC NMR (400 MHz, $CDCl_3$)

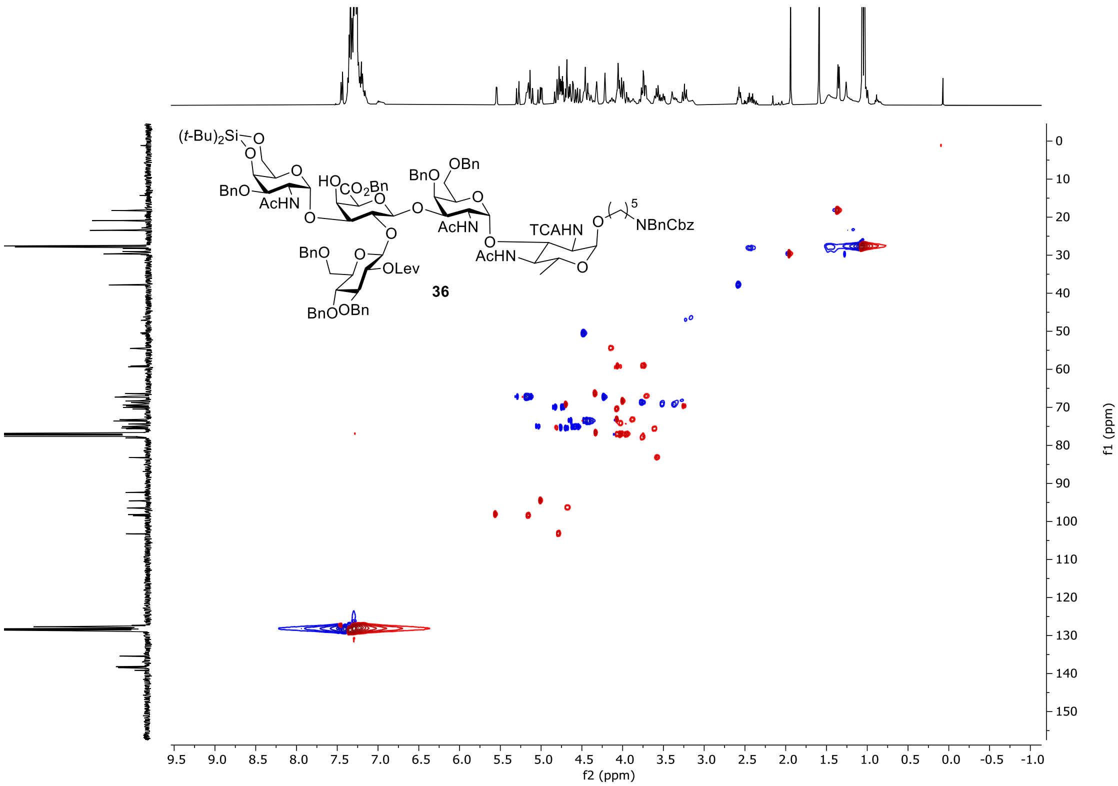

^1H-^{13}C Coupled HSQC NMR (400 MHz, $CDCl_3$)

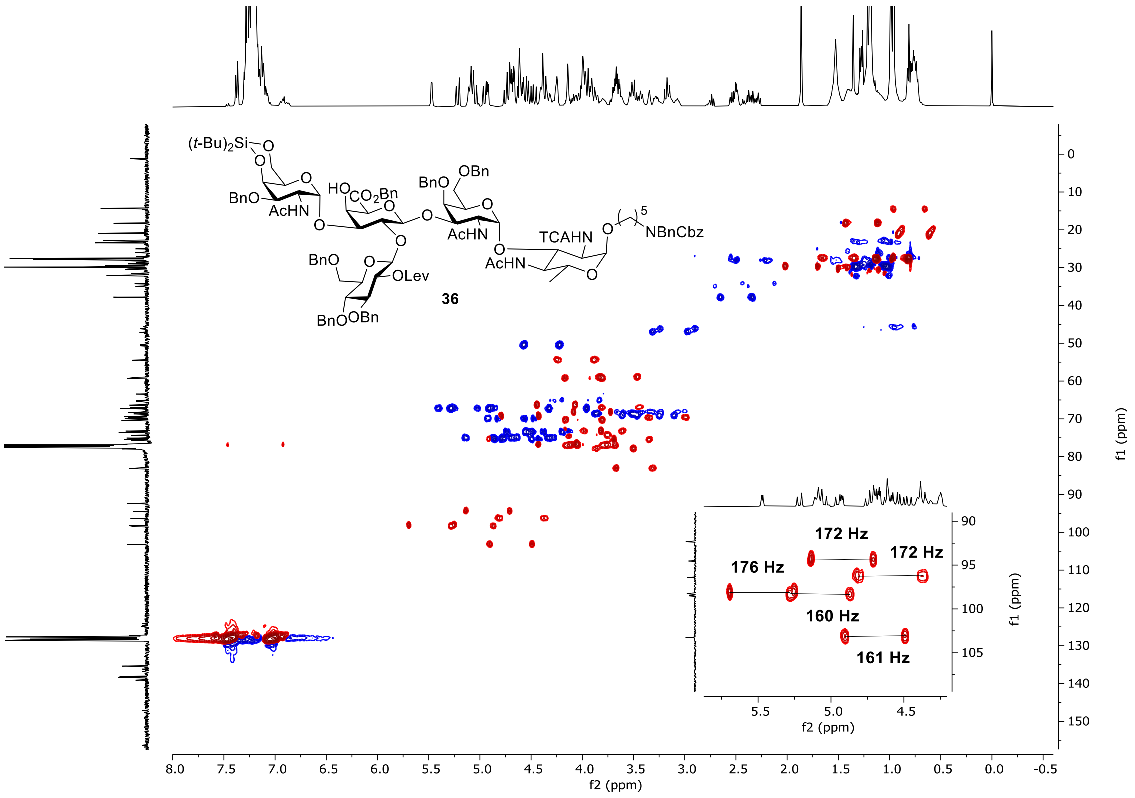

^1H NMR (700 MHz, $CDCl_3$)

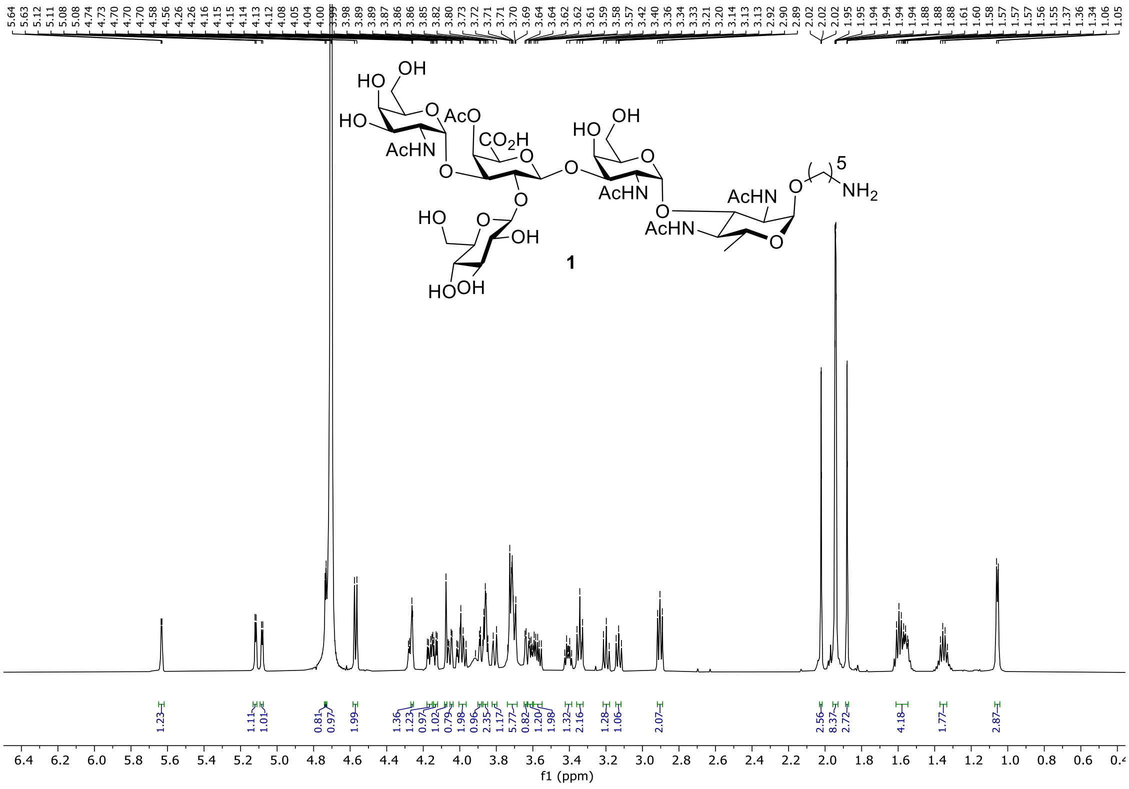

^{13}C NMR (176 MHz, $CDCl_3$)

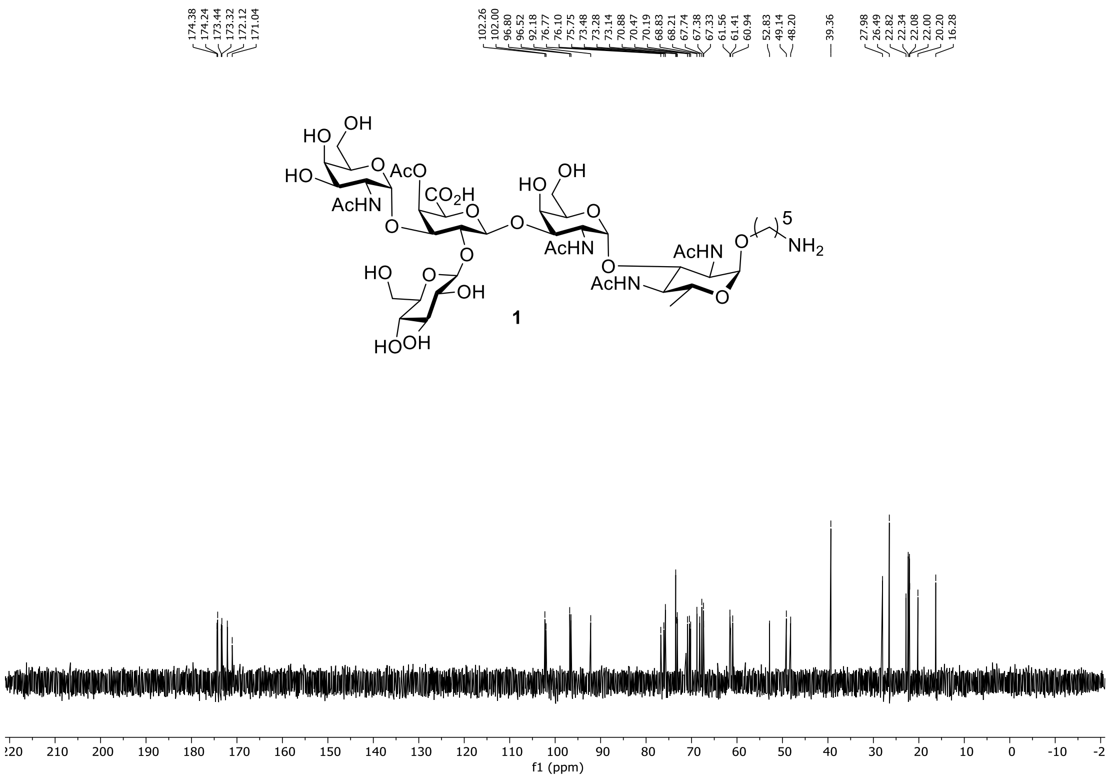

^{1}H-^{1}H COSY NMR (700 MHz, $CDCl_3$)

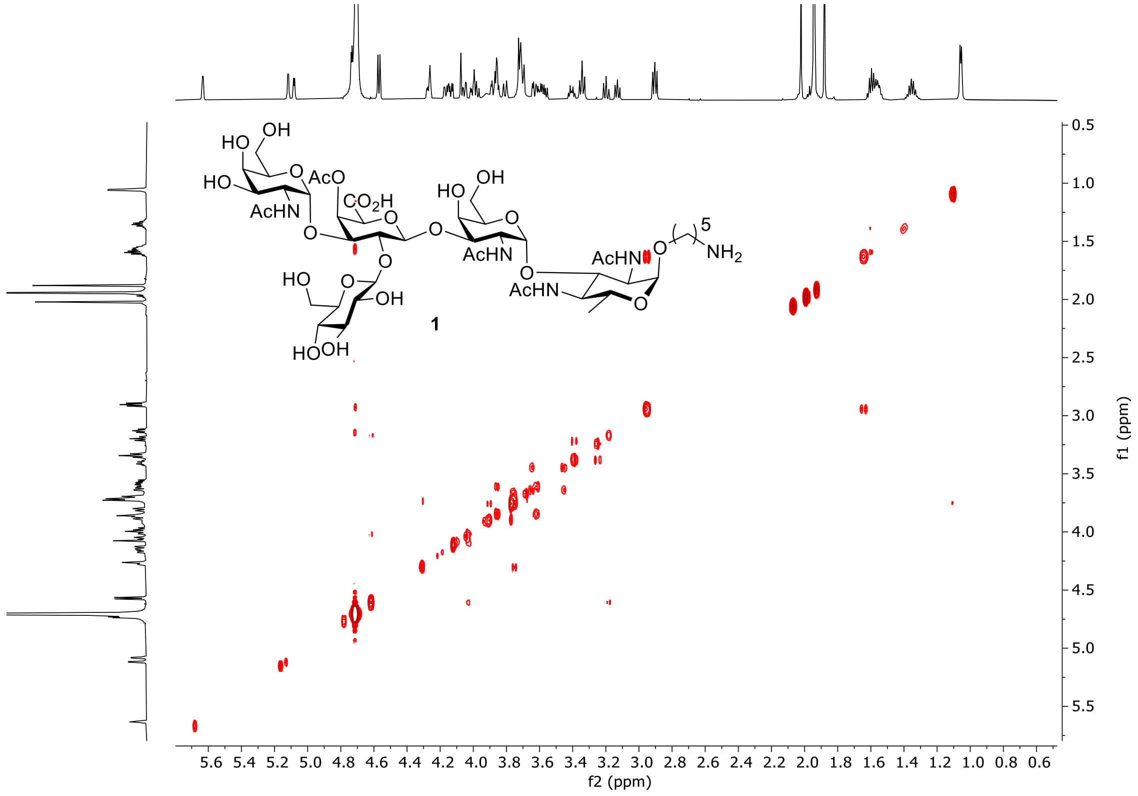

^{1}H-^{13}C HSQC NMR (700 MHz, $CDCl_3$)

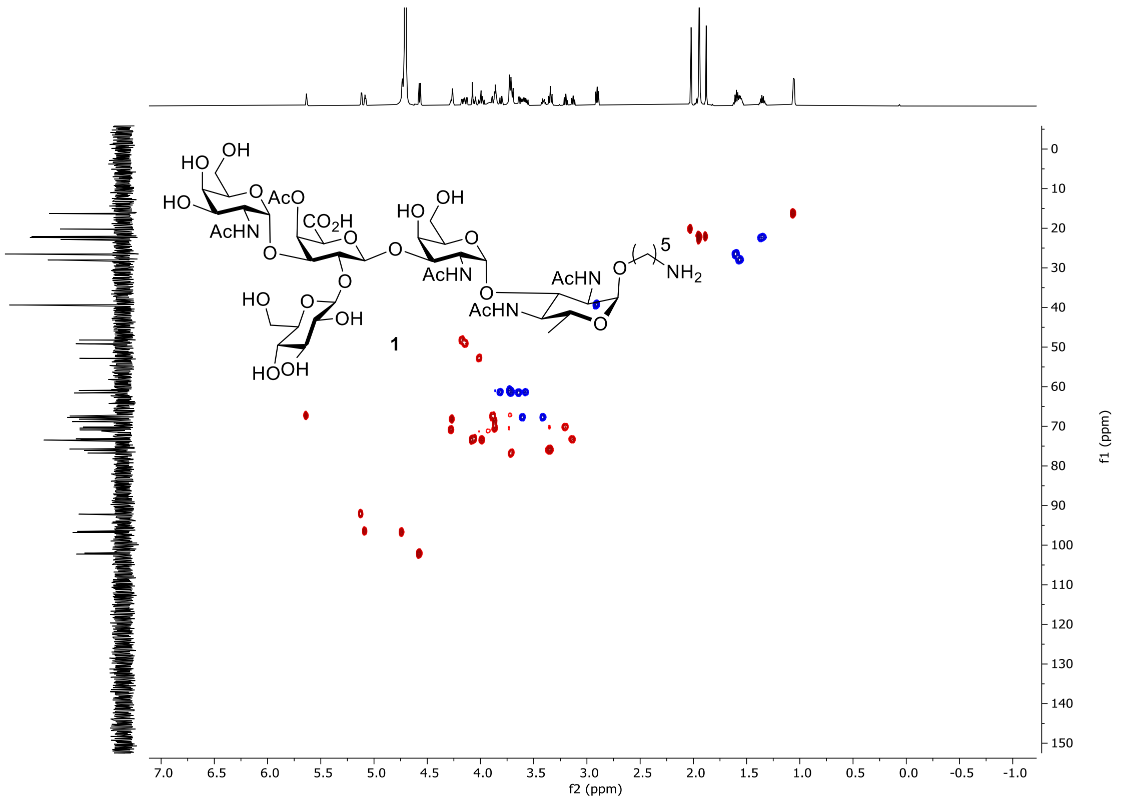

^{1}H-^{13}C Coupled HSQC NMR (700 MHz, $CDCl_3$)

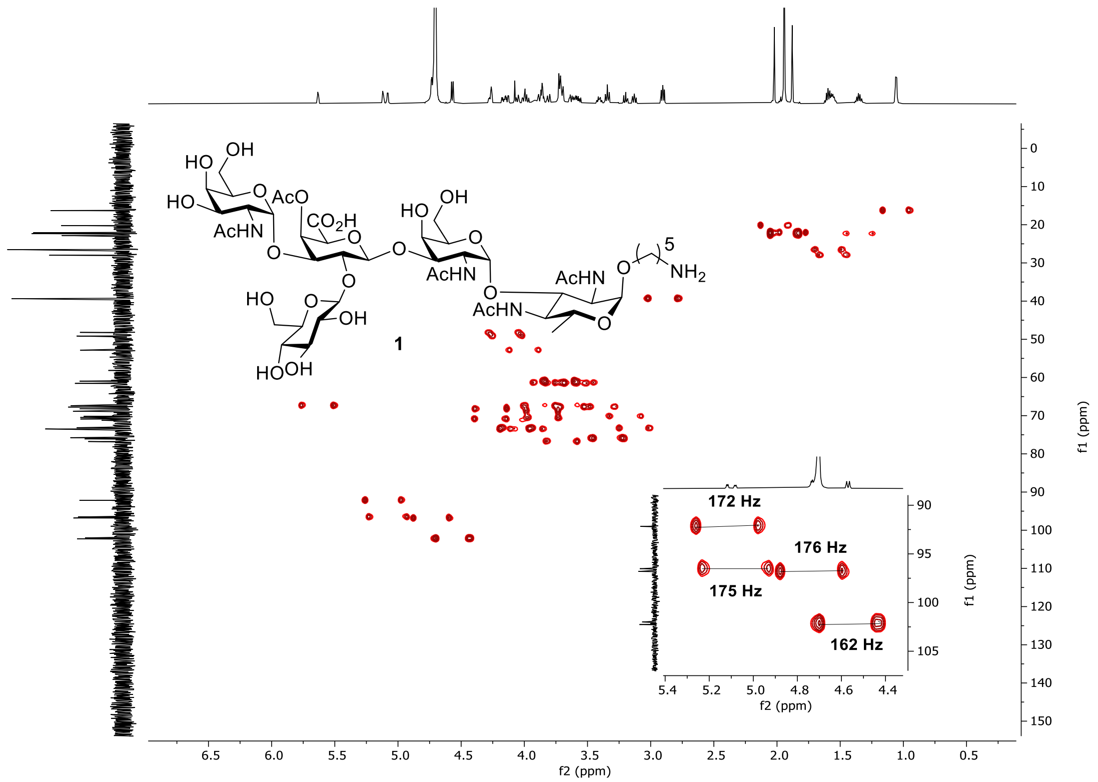

^{1}H NMR (700 MHz, $CDCl_3$)

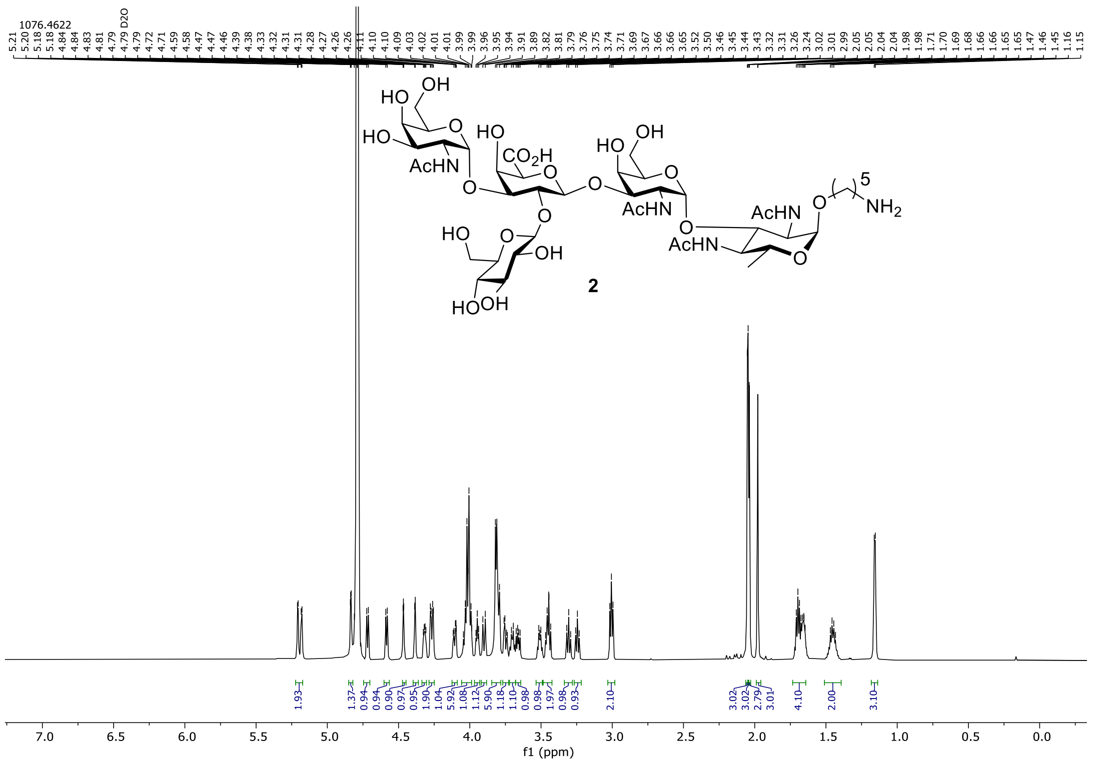

^{13}C NMR (176 MHz, $CDCl_3$)

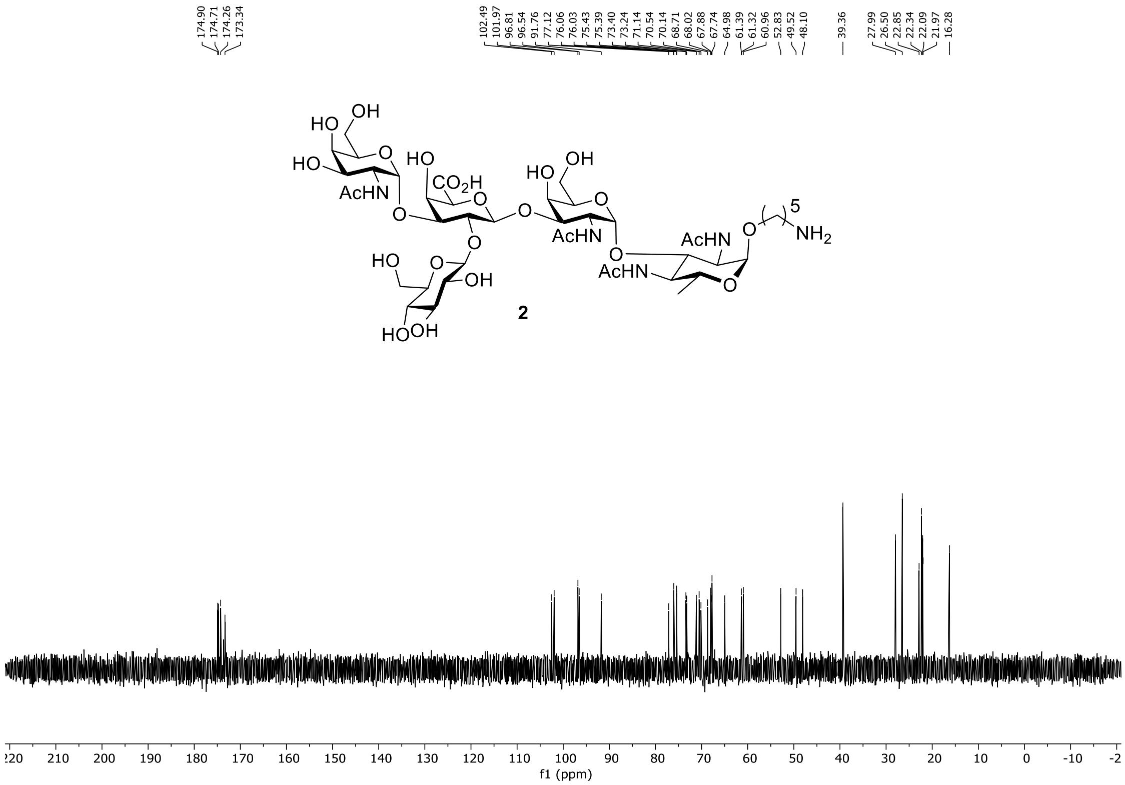

^{1}H-^{1}H COSY NMR (700 MHz, $CDCl_3$)

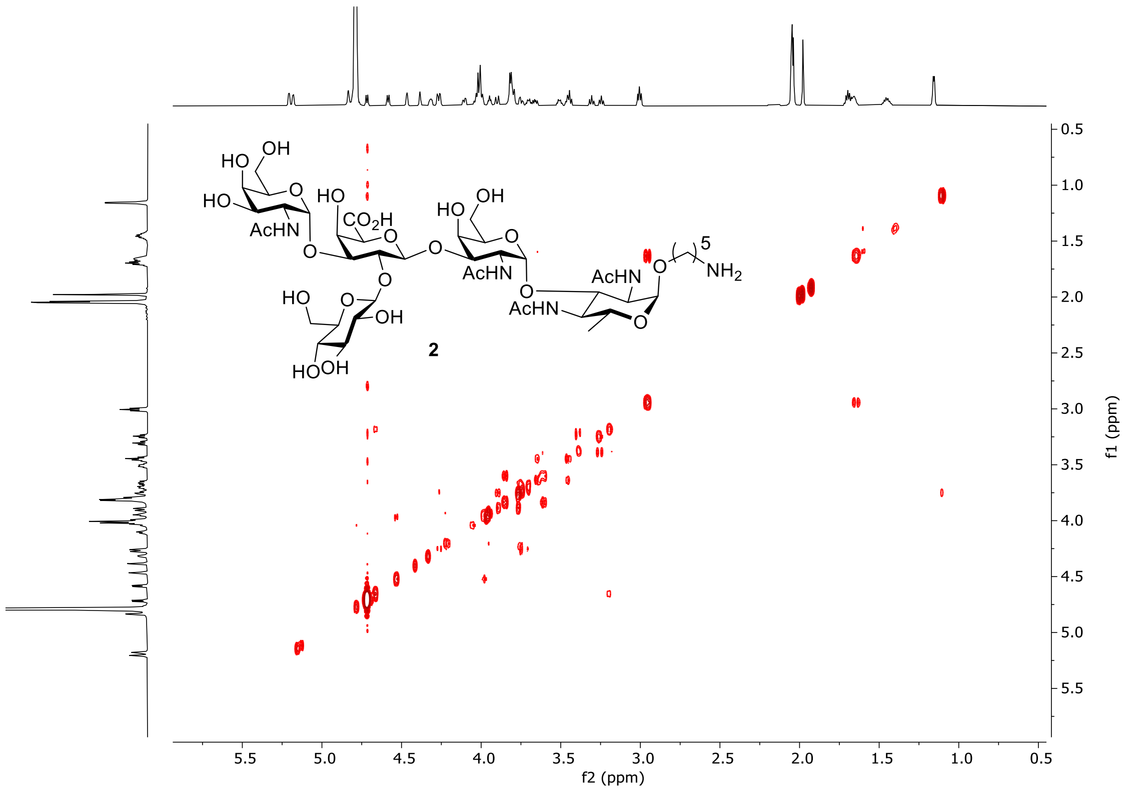

^{1}H-^{13}C HSQC NMR (700 MHz, $CDCl_3$)

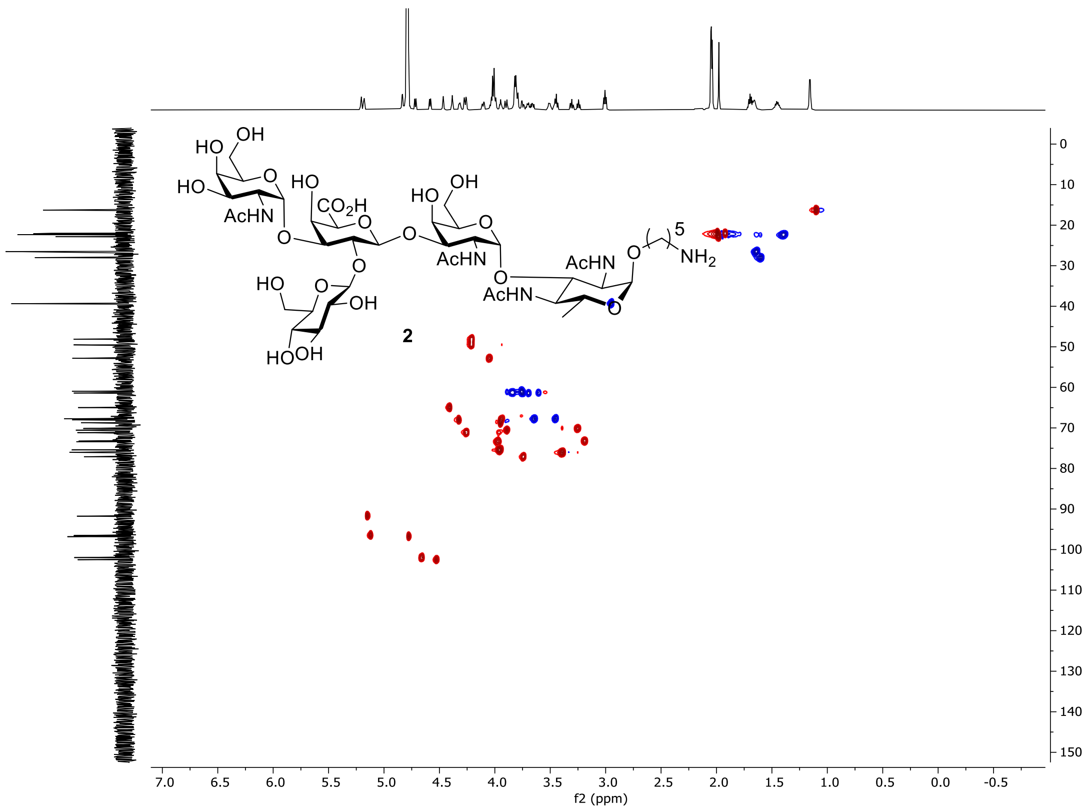

^1H-^{13}C Coupled HSQC NMR (700 MHz, $CDCl_3$)

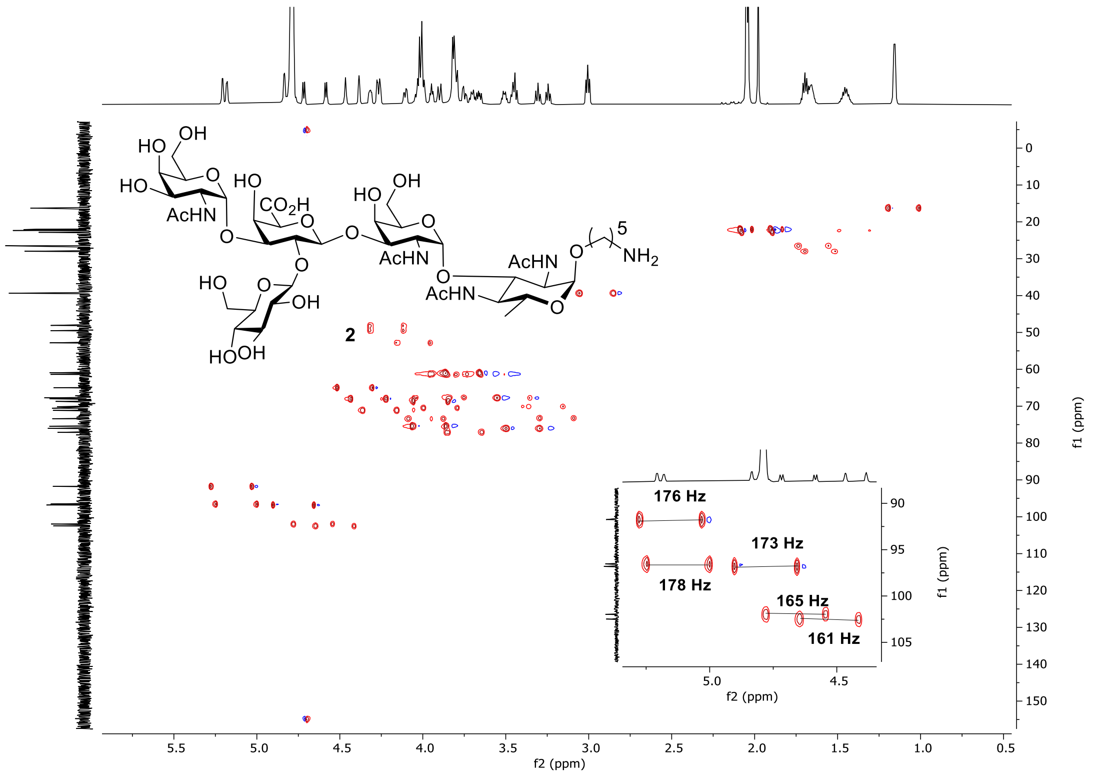

^1H NMR (400 MHz, $CDCl_3$)

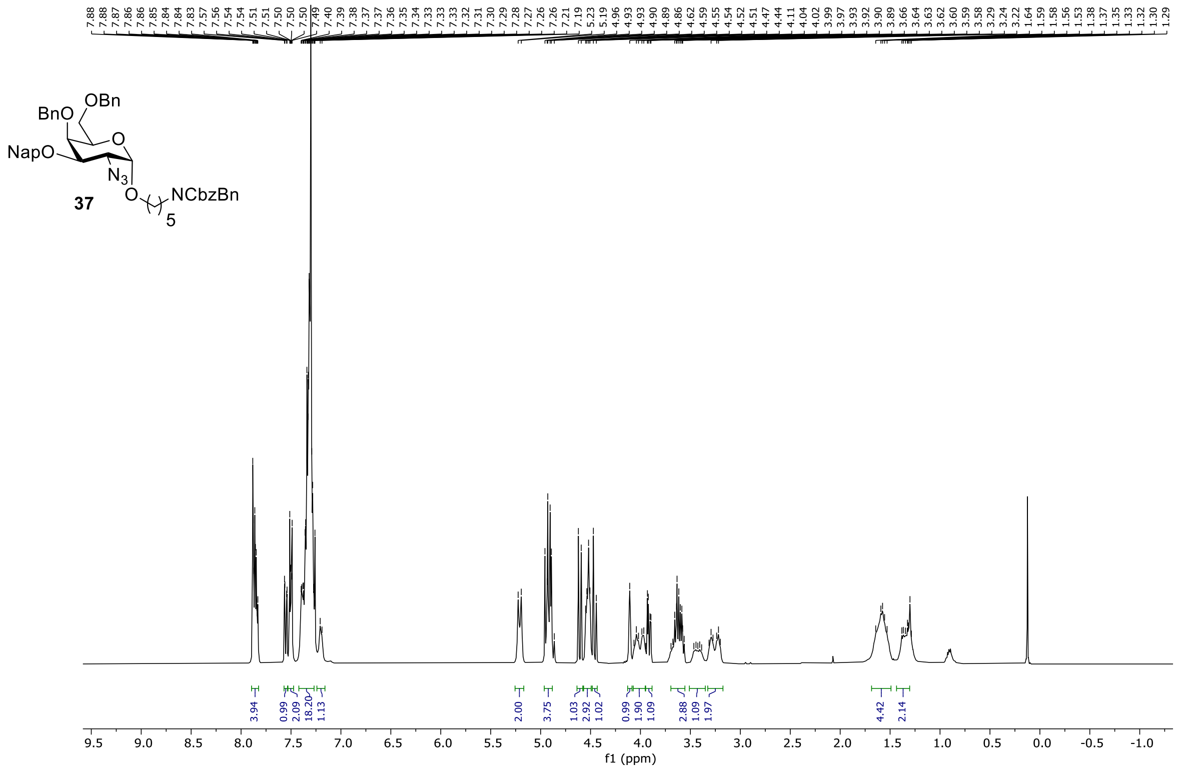

^{13}C NMR (101 MHz, $CDCl_3$)

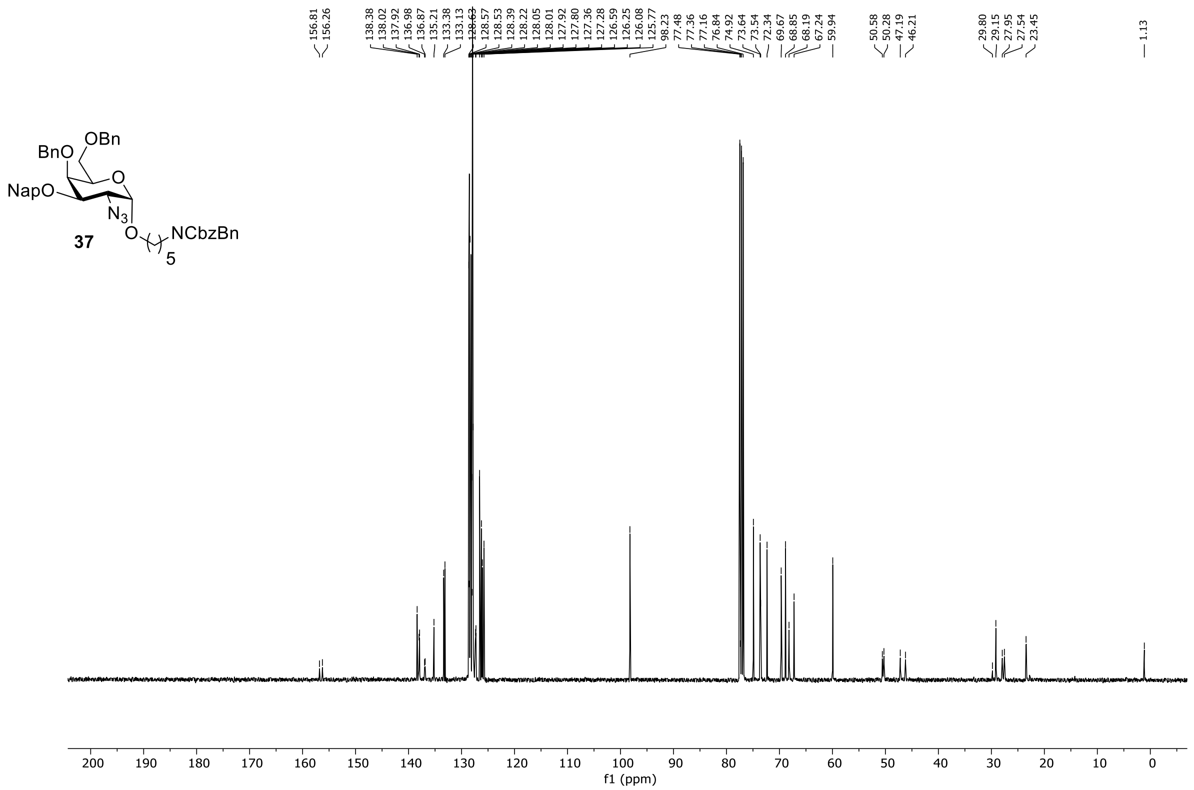

^{1}H-^{1}H COSY NMR (400 MHz, $CDCl_3$)

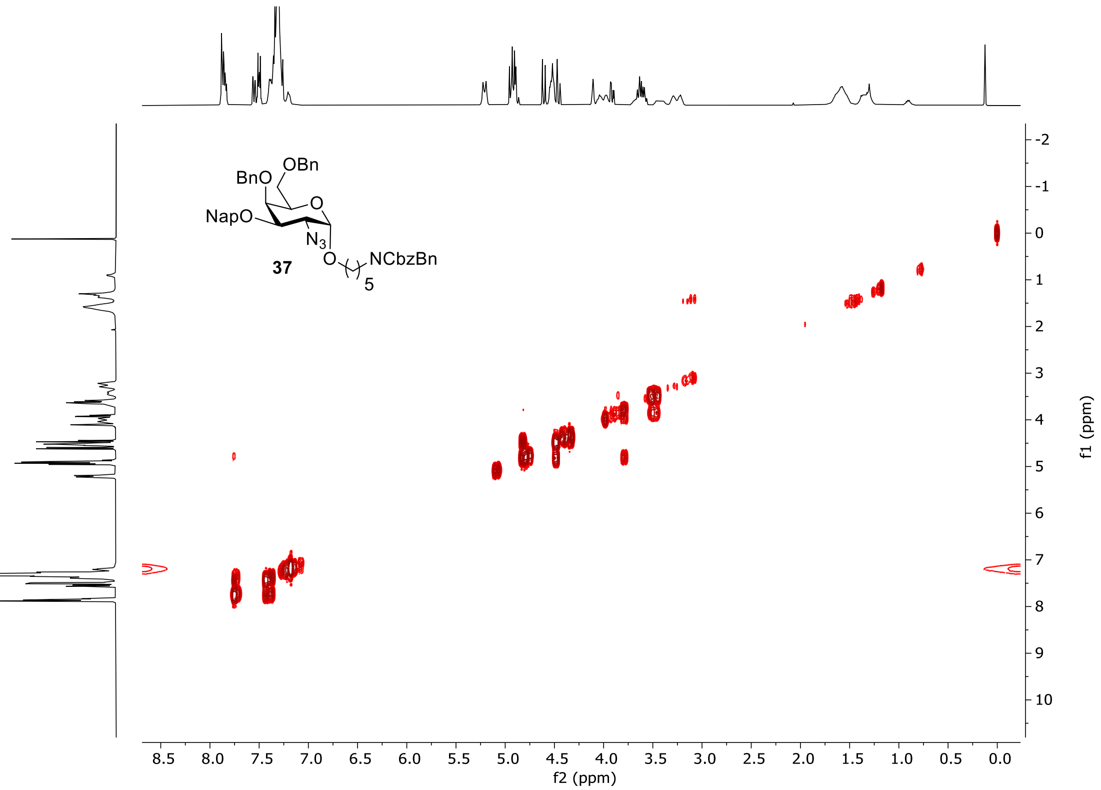

^{1}H-^{13}C HSQC NMR (400 MHz, $CDCl_3$)

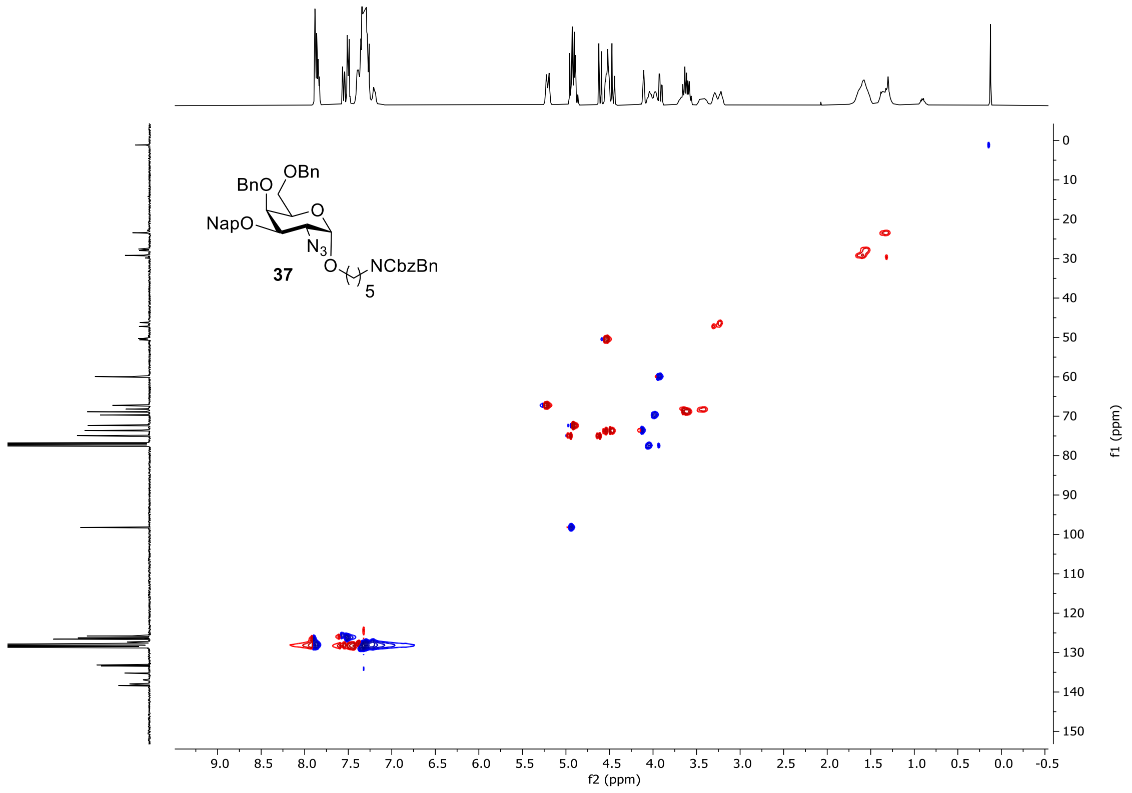

^{1}H NMR (400 MHz, $CDCl_3$)

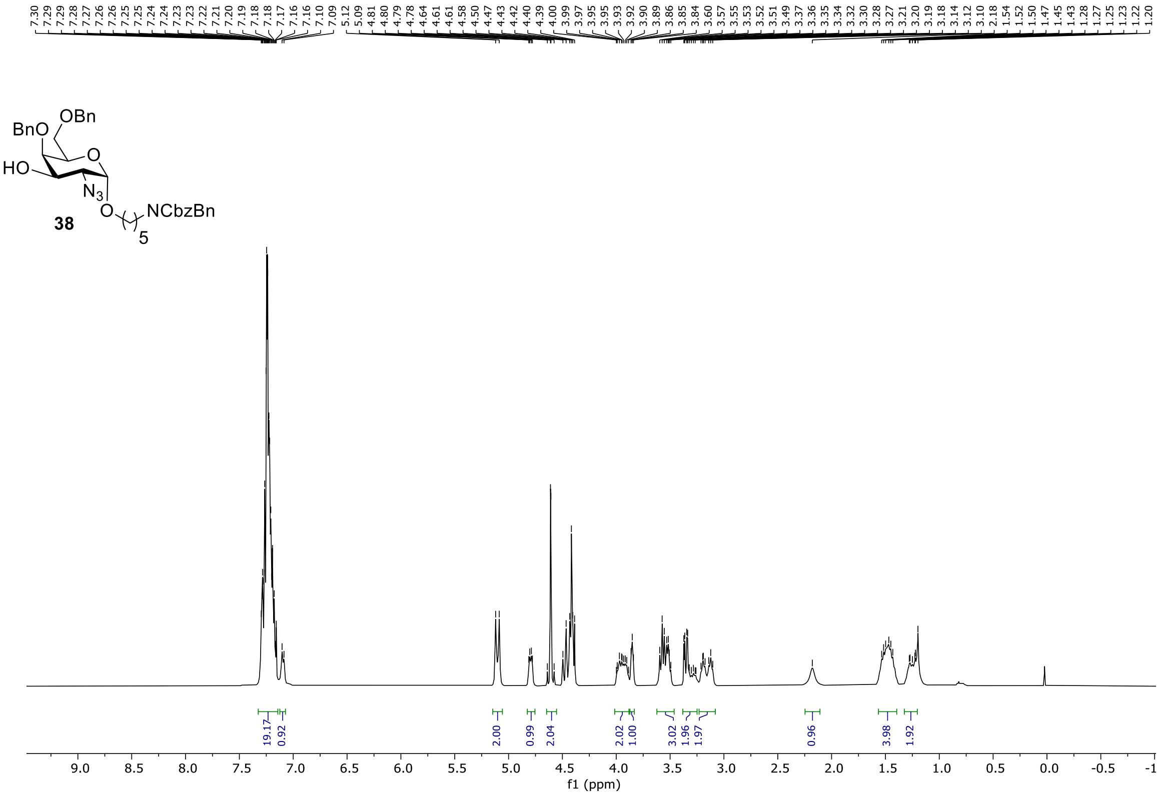

^{13}C NMR (101 MHz, CDCl$_3$)

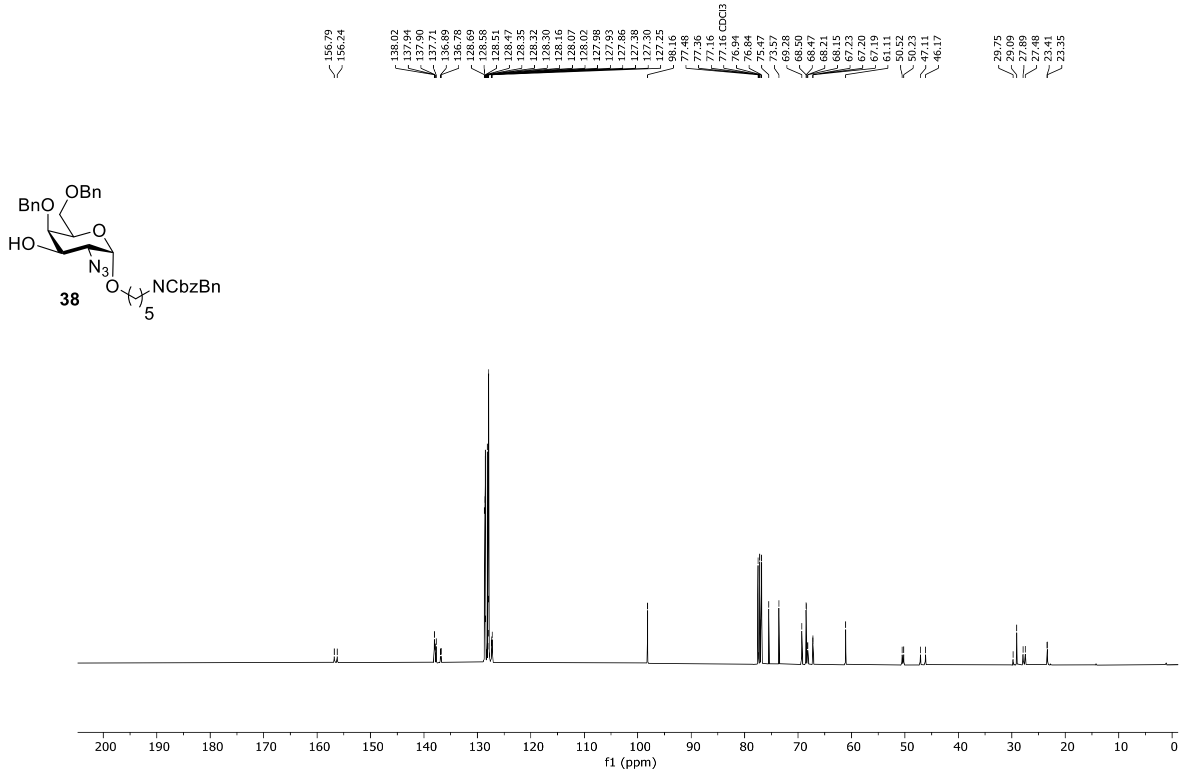

^{1}H-^{1}H COSY NMR (400 MHz, CDCl$_3$)

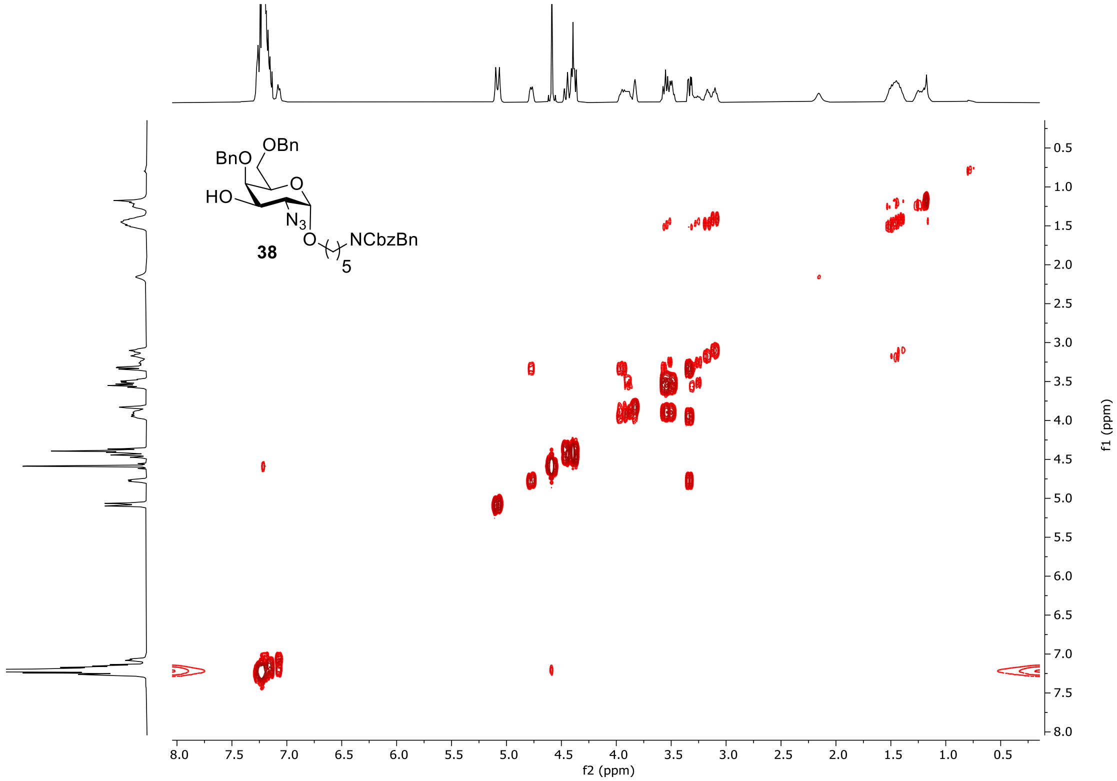

^{1}H-^{13}C HSQC NMR (400 MHz, $CDCl_3$)

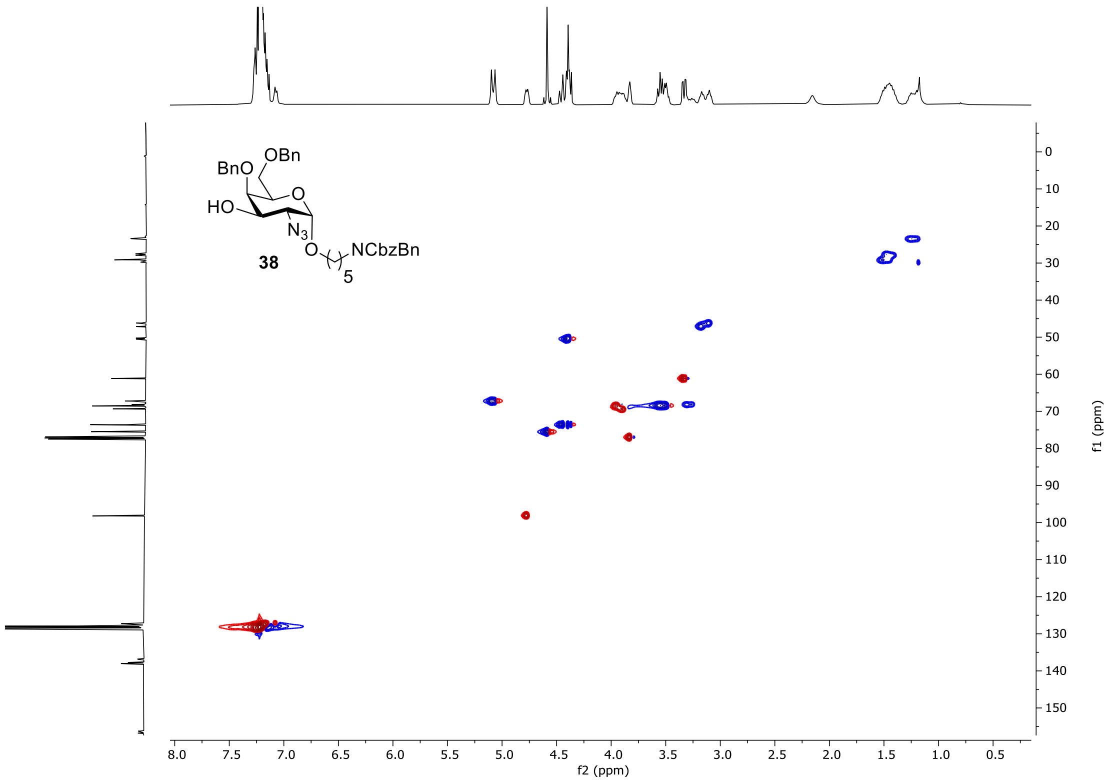

^{1}H-^{13}C Coupled HSQC NMR (400 MHz, $CDCl_3$)

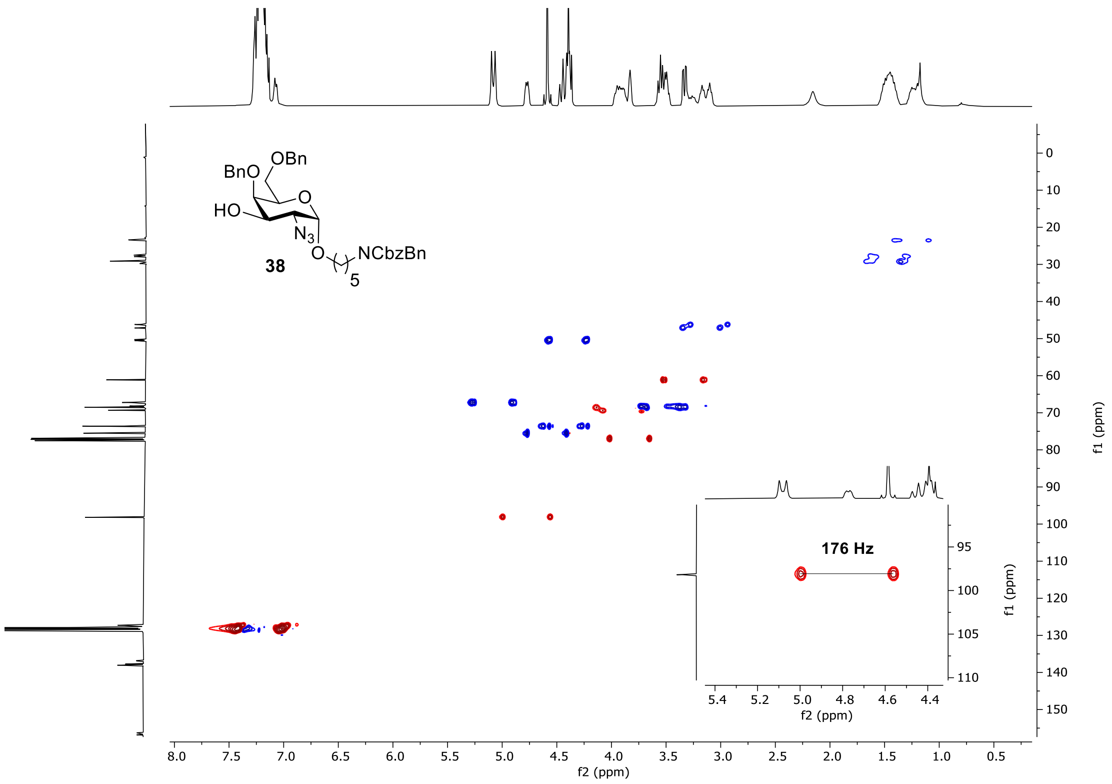

^{1}H NMR (600 MHz, $CDCl_3$)

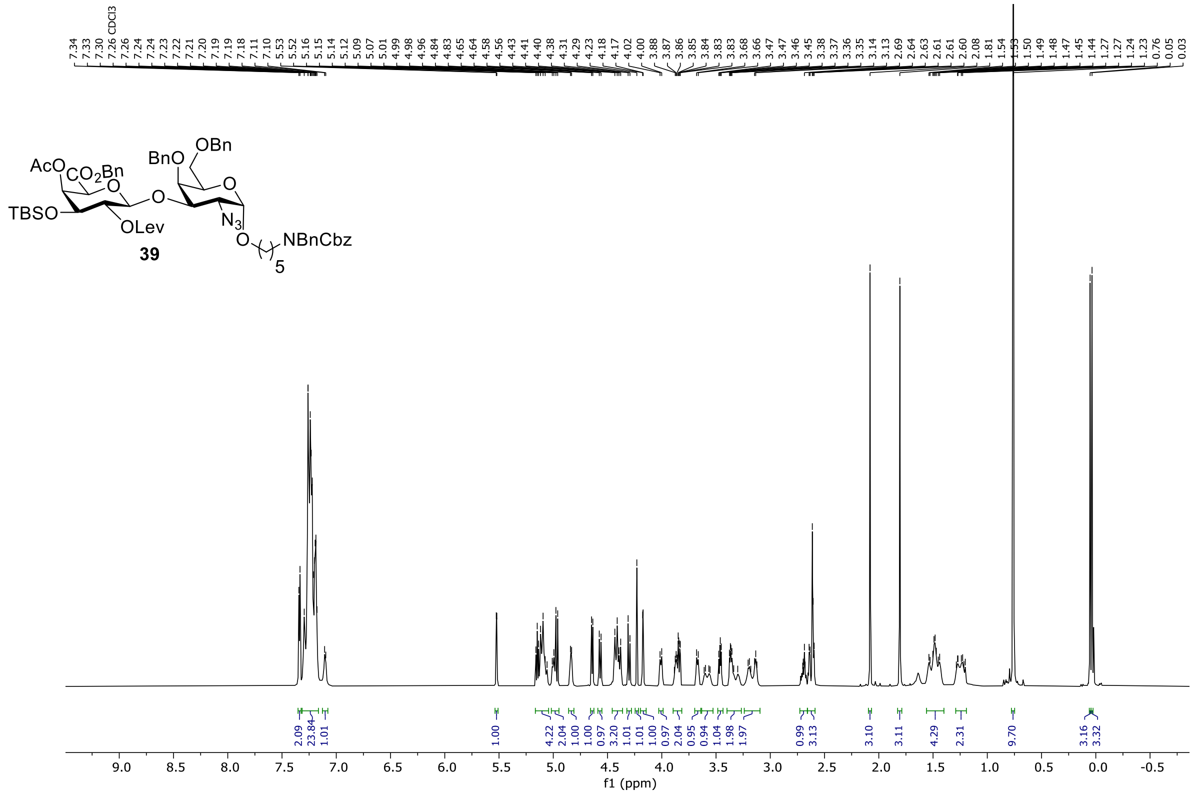

^{13}C NMR (151 MHz, $CDCl_3$)

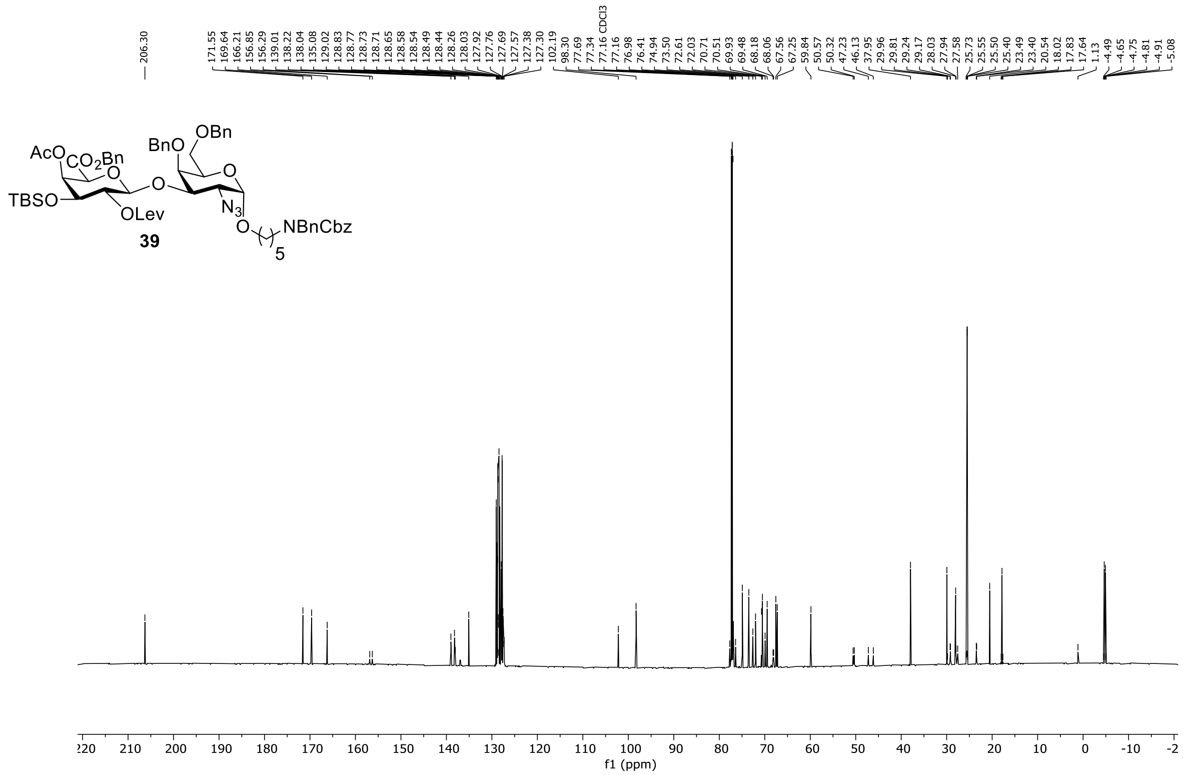

^{1}H-^{1}H COSY NMR (600 MHz, $CDCl_3$)

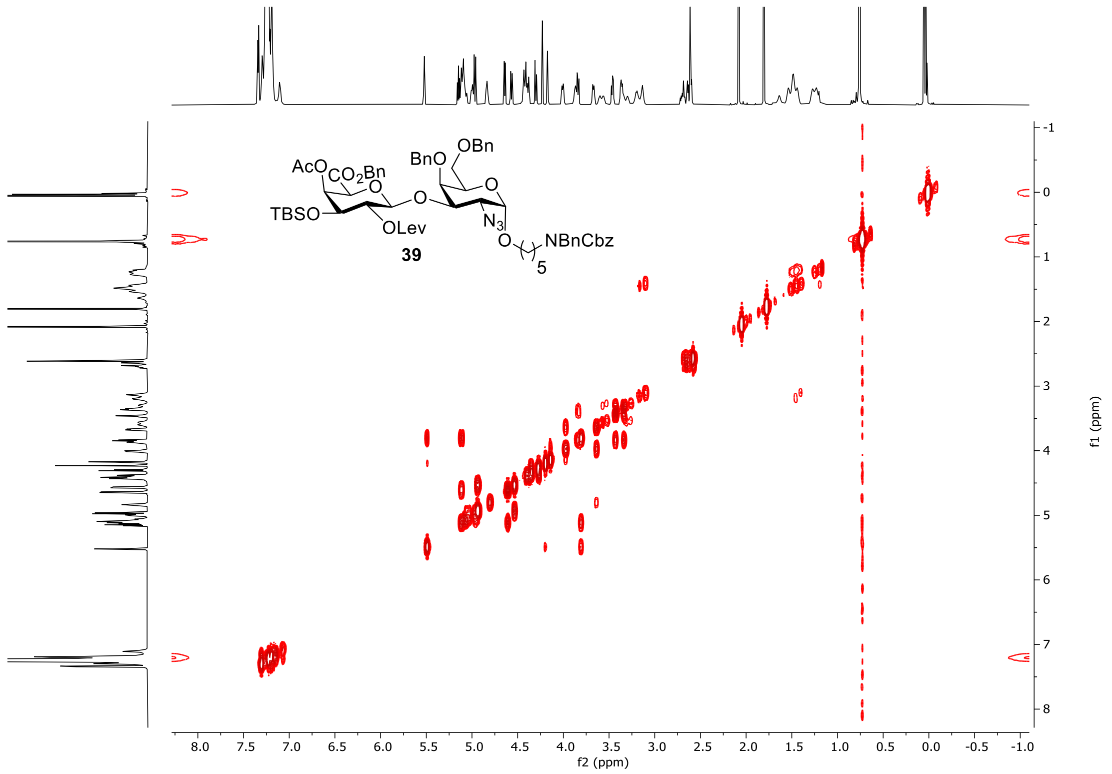

^{1}H-^{13}C HSQC NMR (600 MHz, $CDCl_3$)

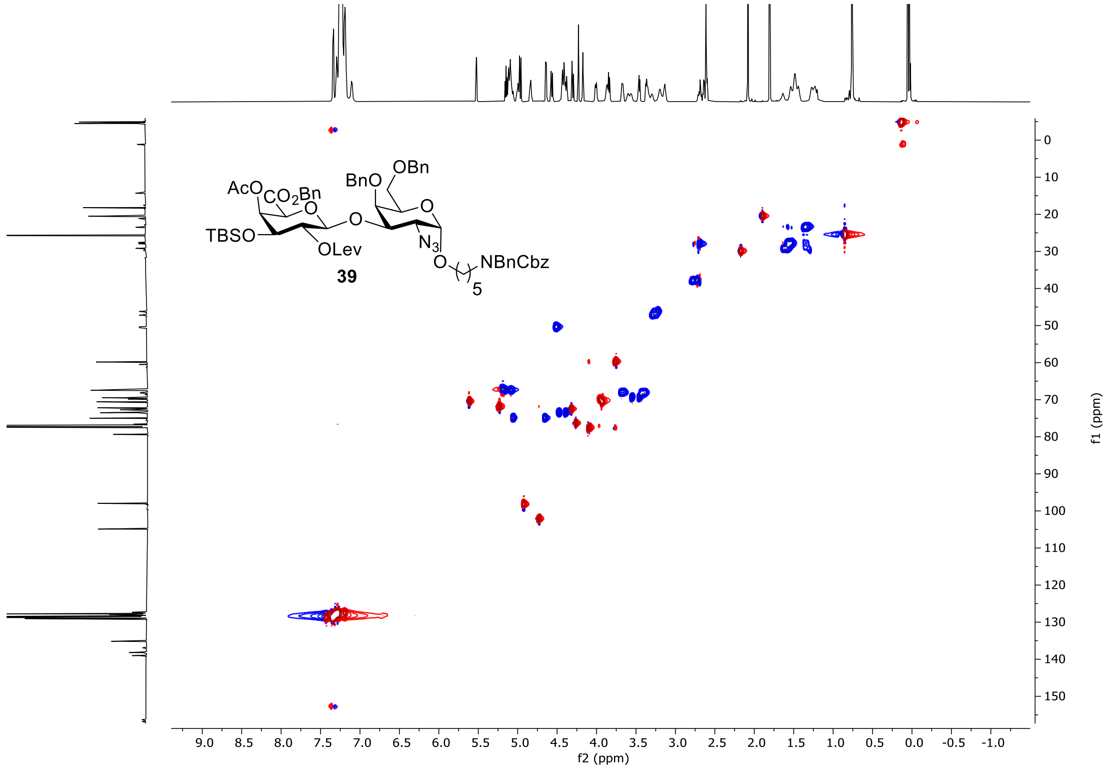

^{1}H-^{13}C Coupled HSQC NMR (600 MHz, $CDCl_3$)

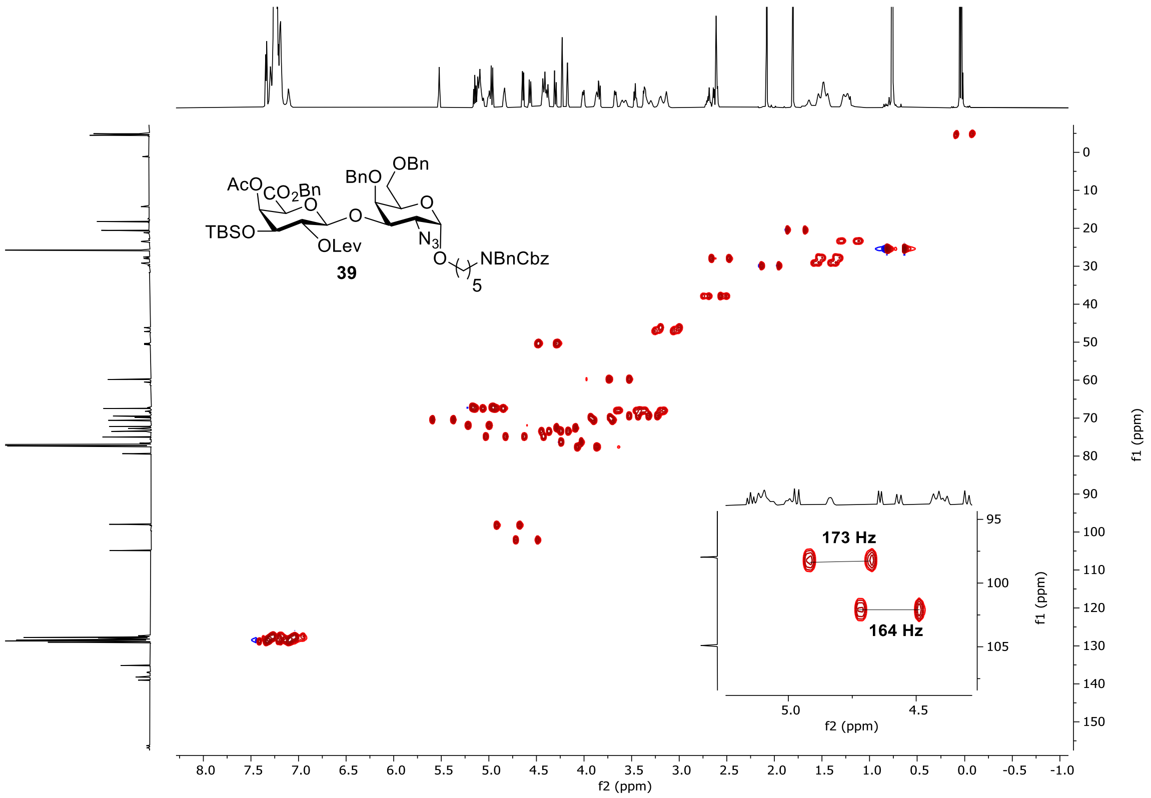

^{1}H NMR (400 MHz, $CDCl_3$)

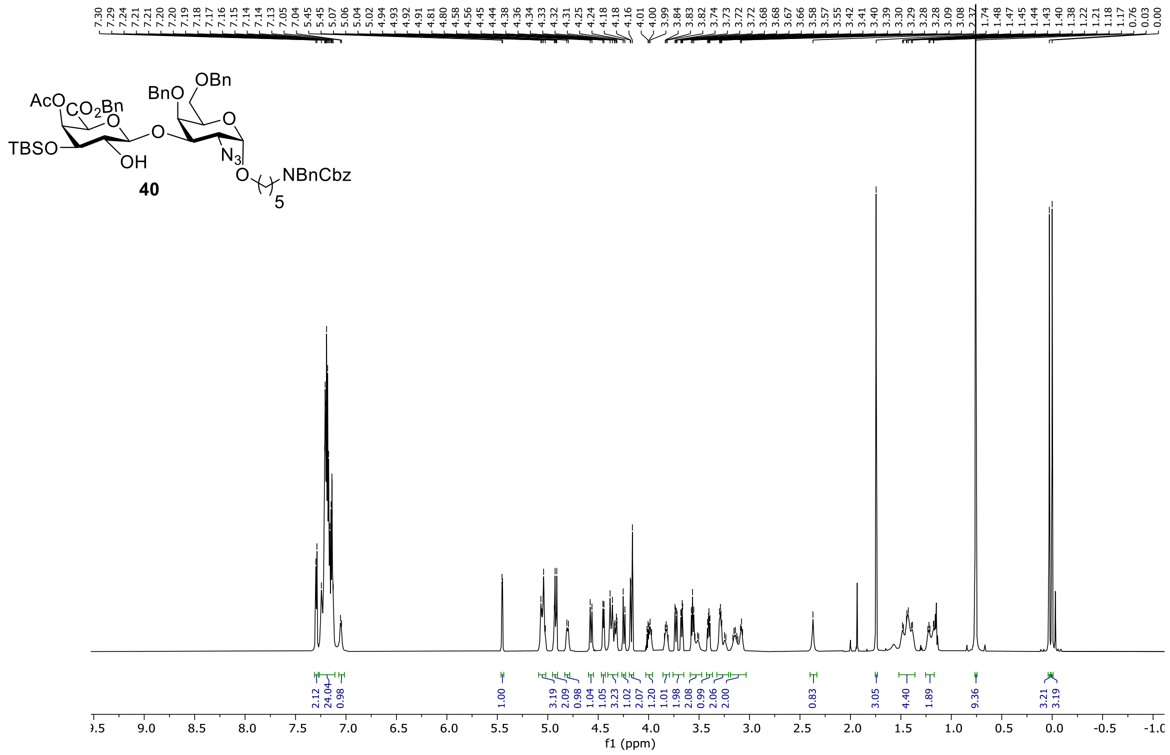

^{13}C NMR (101 MHz, $CDCl_3$)

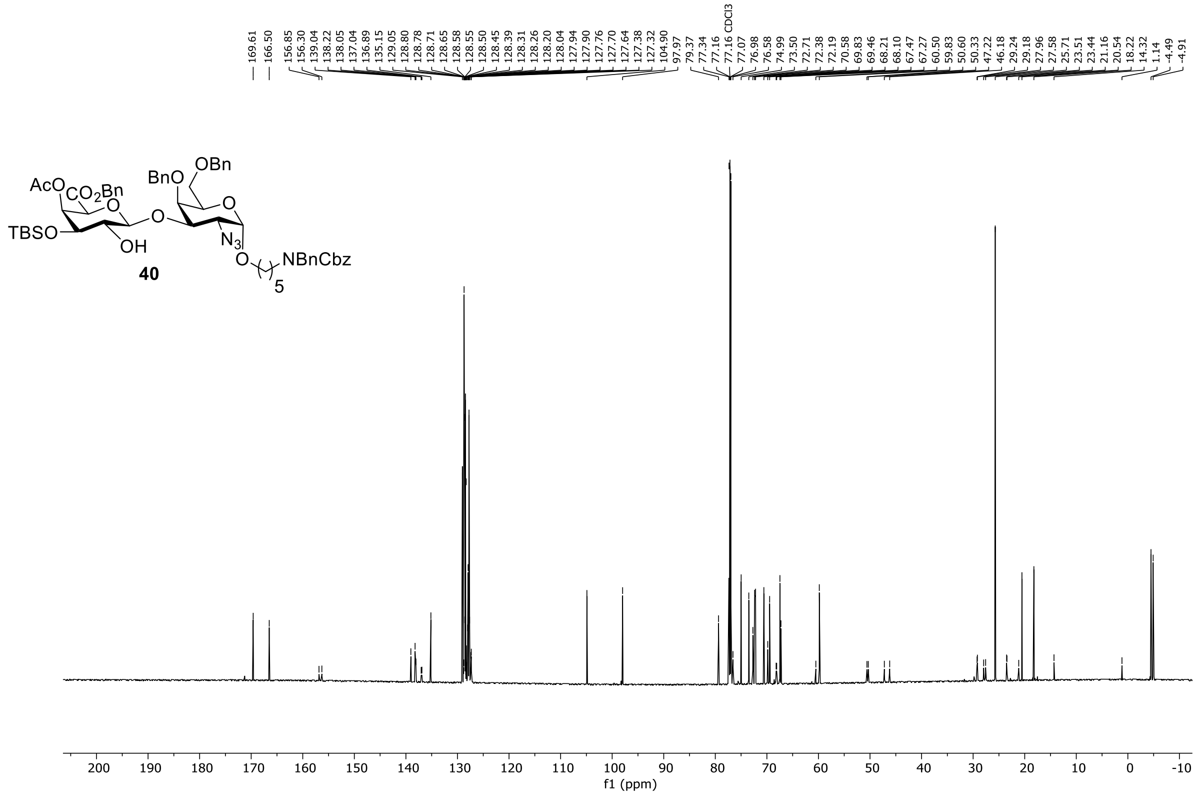

^{1}H-^{1}H COSY NMR (400 MHz, $CDCl_3$)

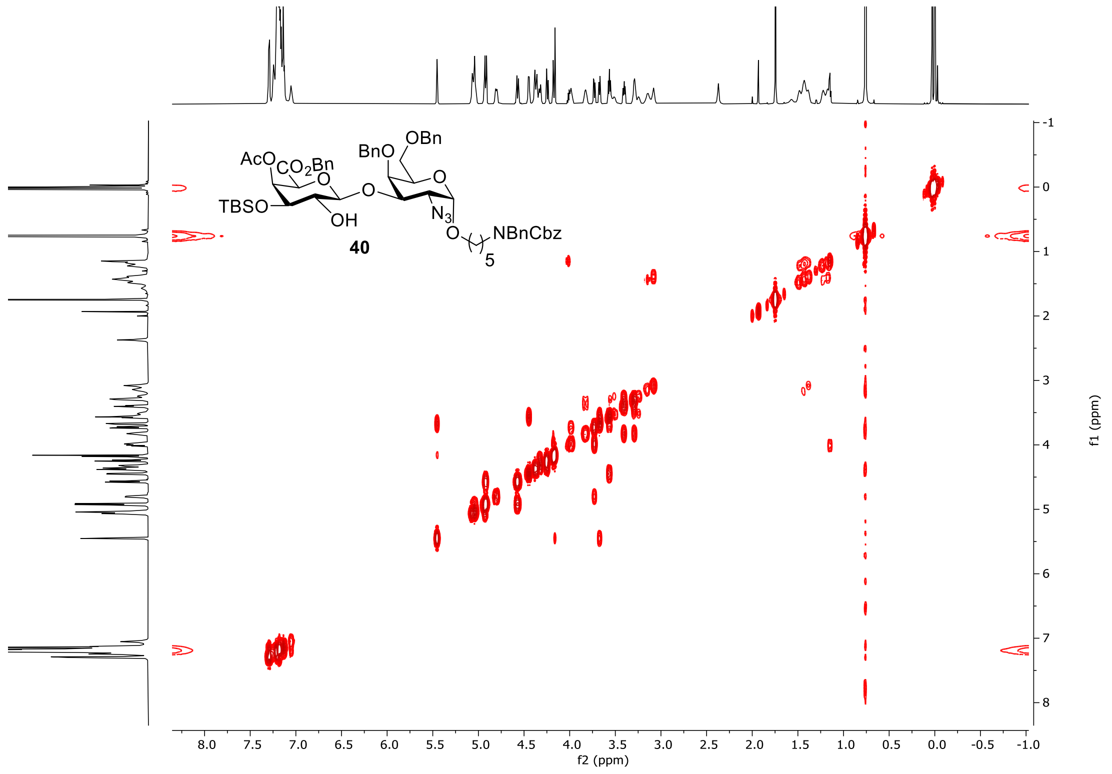

^{1}H-^{13}C HSQC NMR (400 MHz, $CDCl_3$)

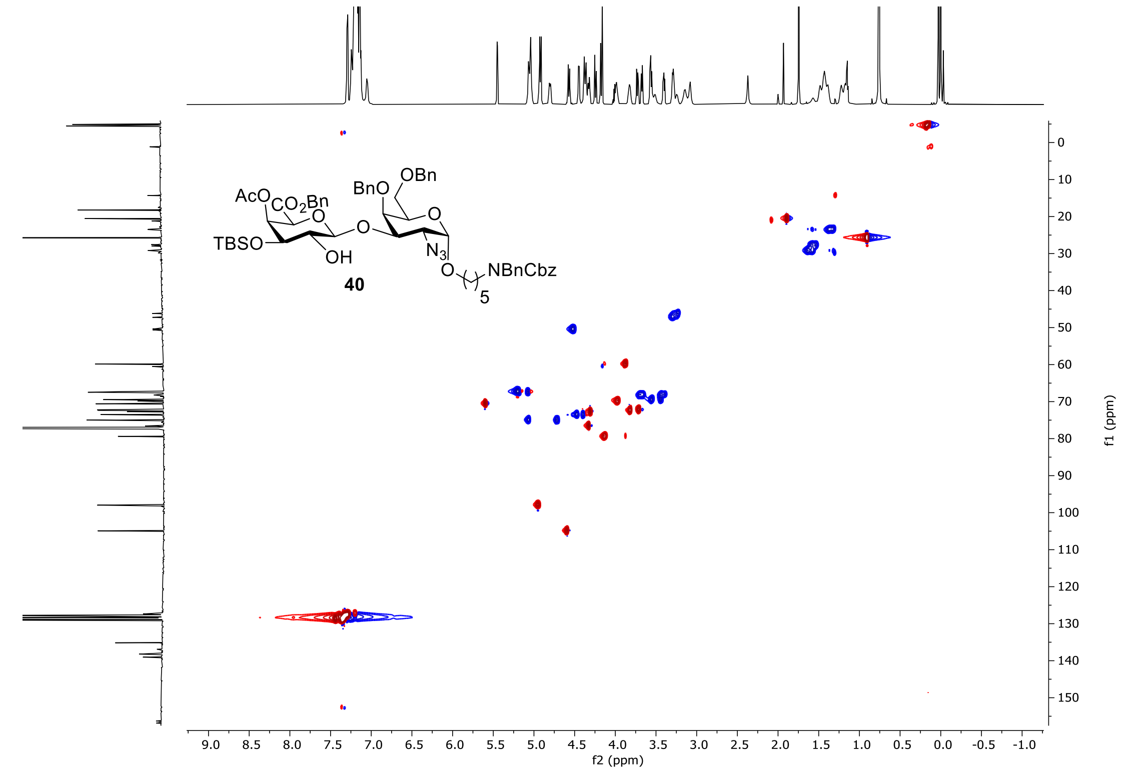

^{1}H-^{13}C Coupled HSQC NMR (400 MHz, $CDCl_3$)

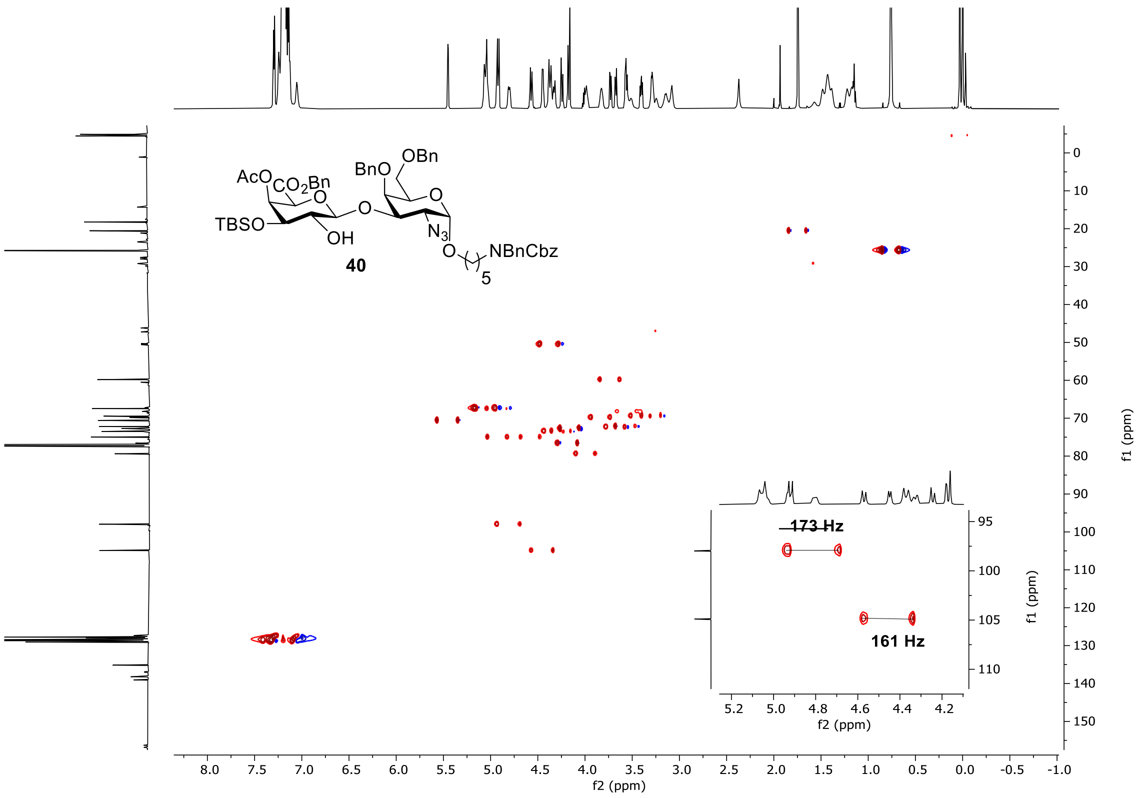

^{1}H NMR (700 MHz, $CDCl_3$)

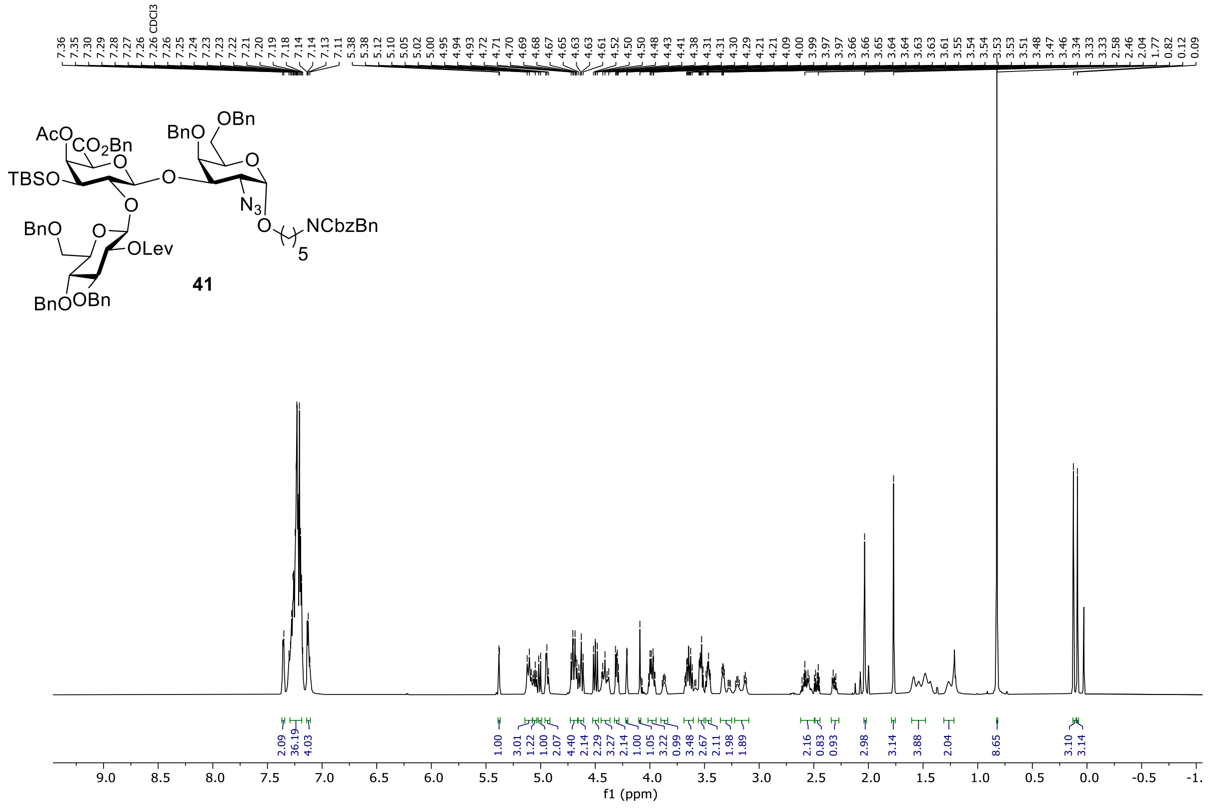

^{1}H-^{13}C NMR (176 MHz, $CDCl_3$)

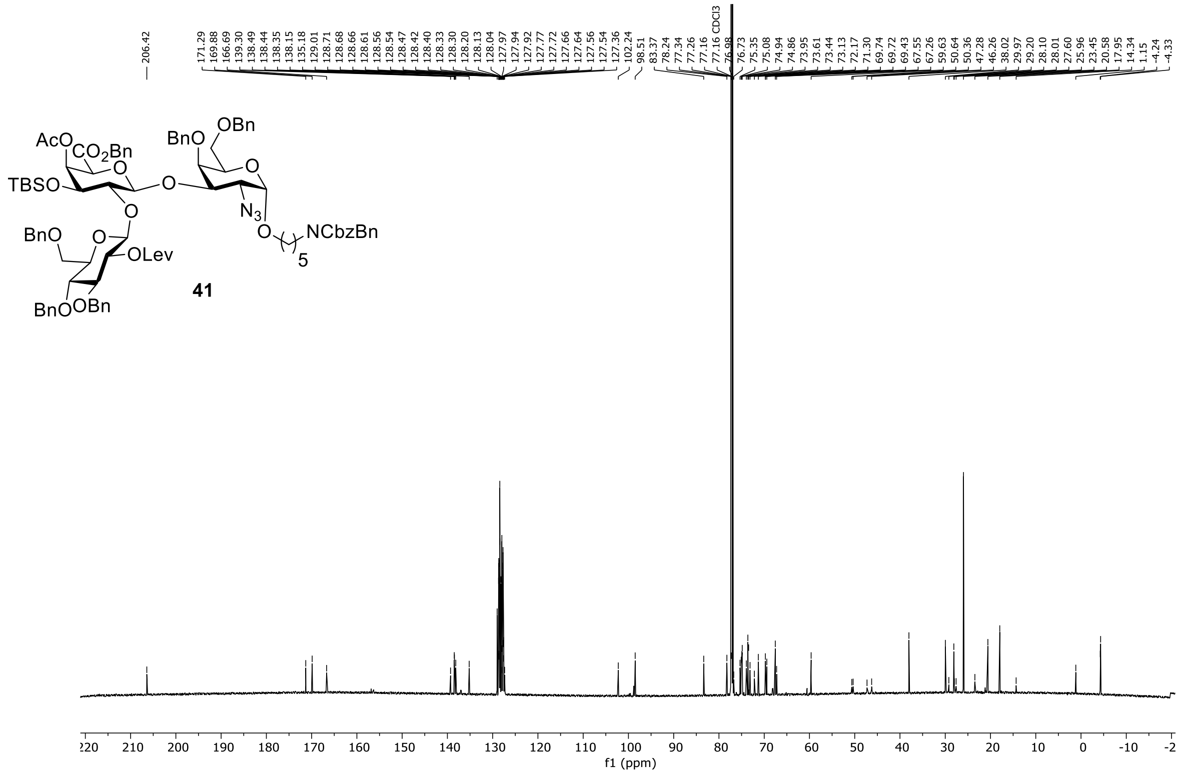

^{1}H-^{1}H COSY NMR (700 MHz, $CDCl_3$)

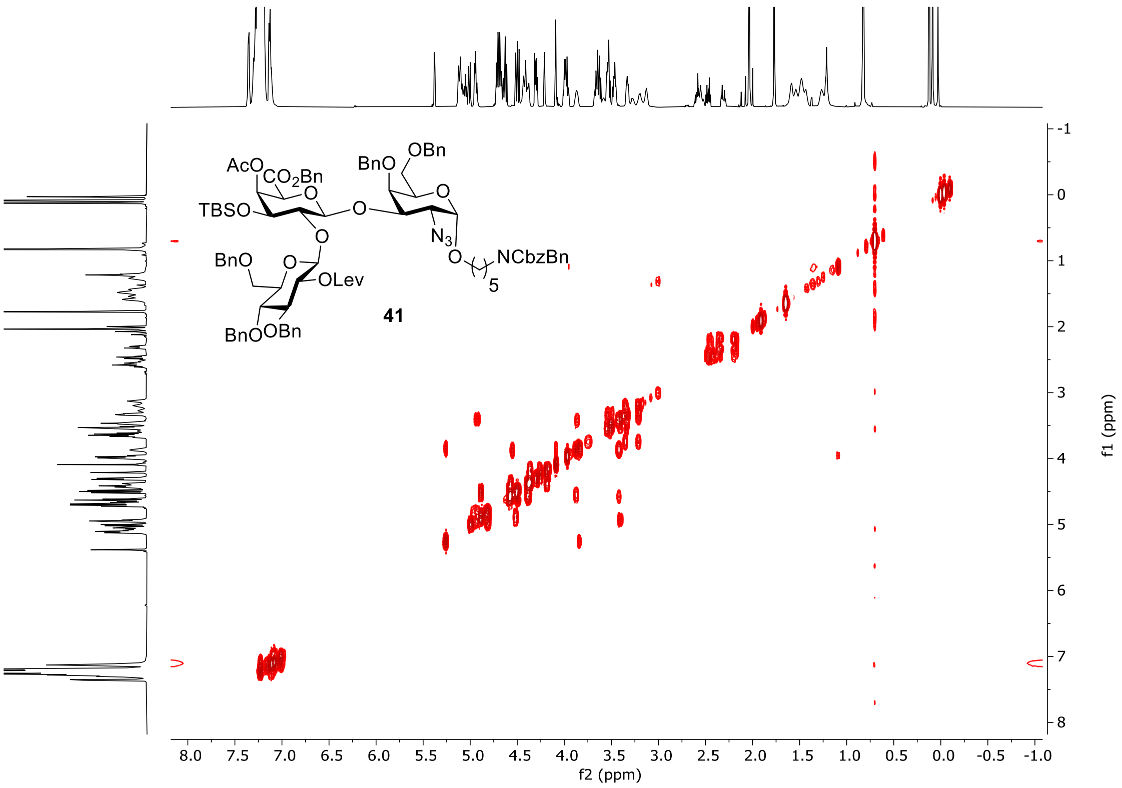

^{1}H-^{13}C HSQC NMR (700 MHz, $CDCl_3$)

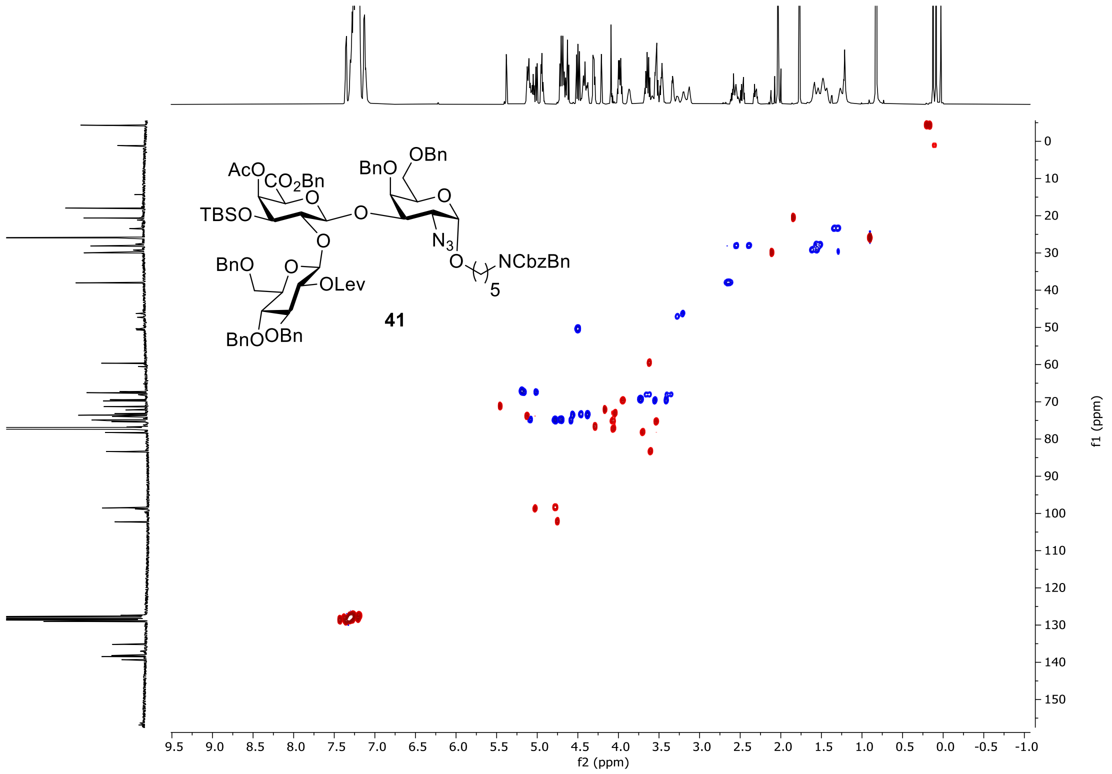

^{1}H-^{13}C Coupled HSQC NMR (700 MHz, $CDCl_3$)

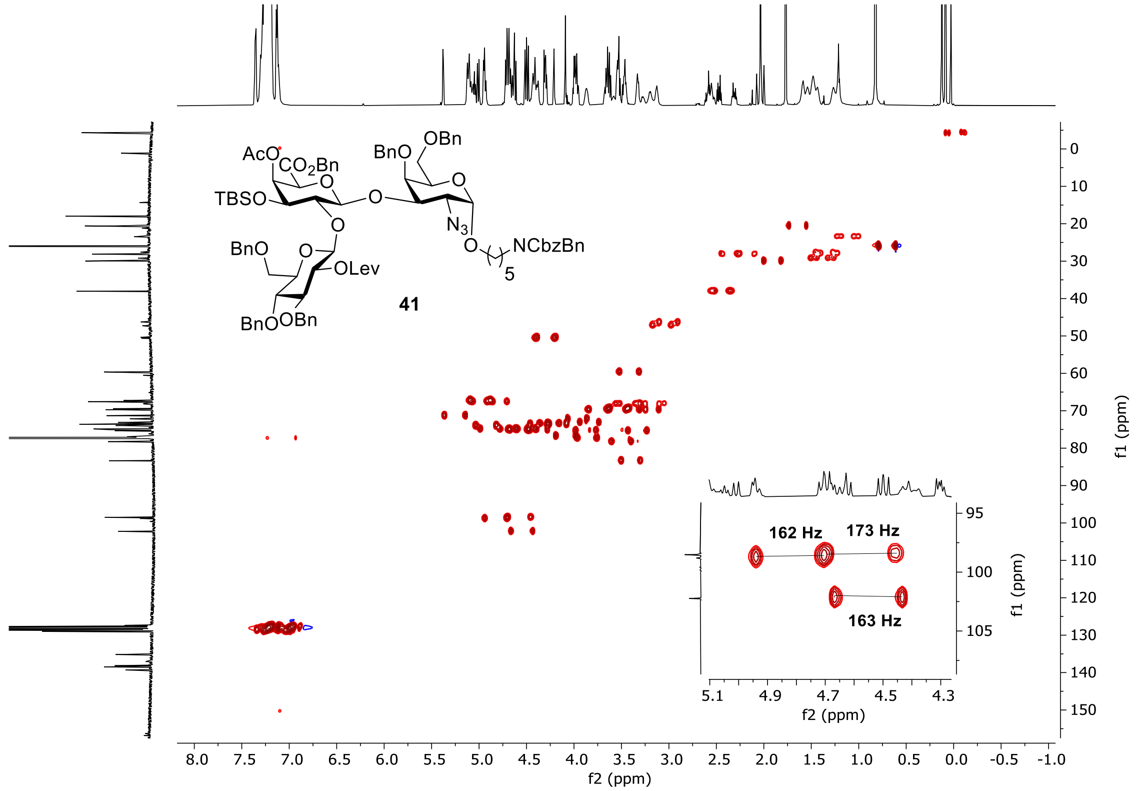

41

^{1}H NMR (700 MHz, $CDCl_3$)

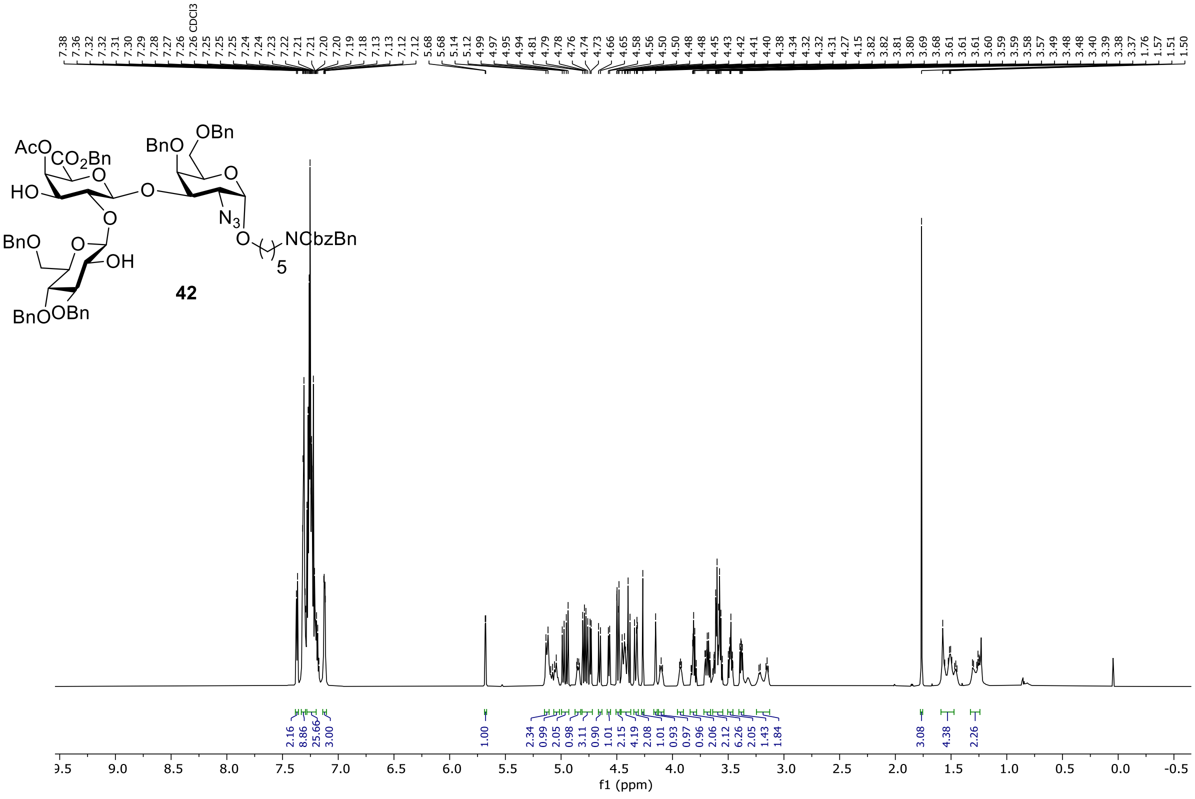

42

^{1}H-^{13}C Coupled HSQC NMR (176 MHz, $CDCl_3$)

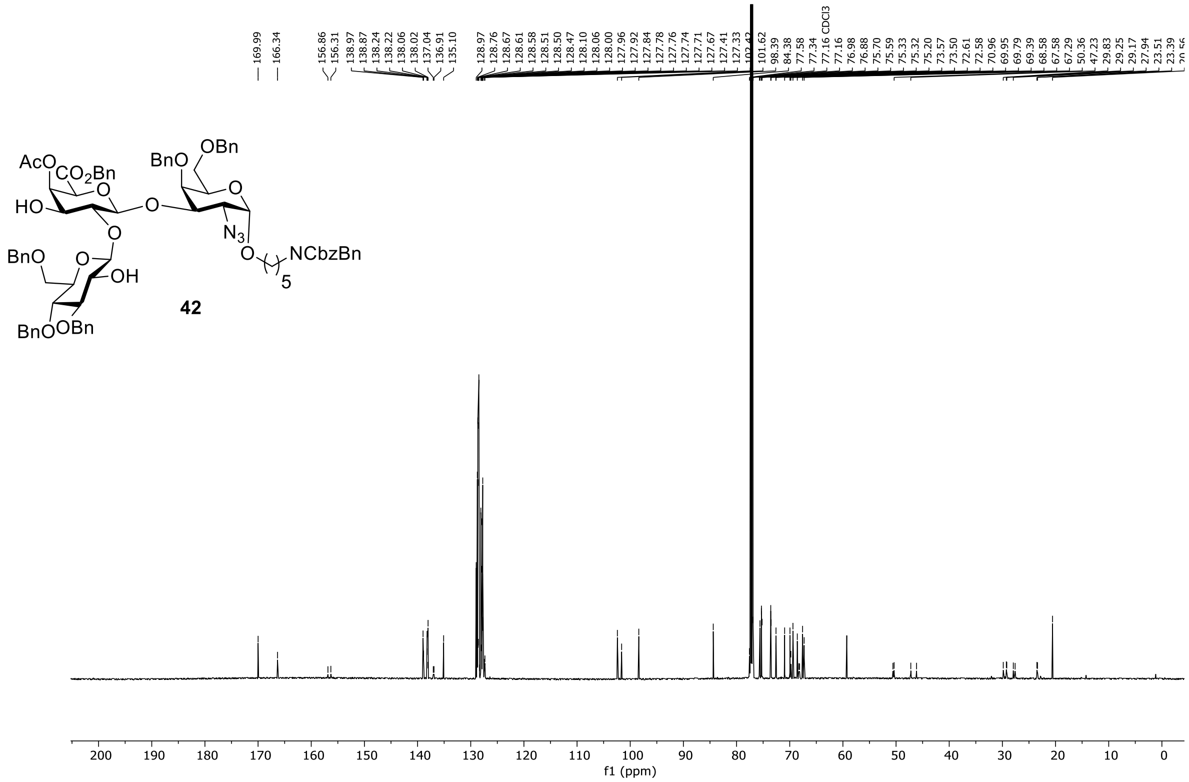

^{1}H-^{1}H COSY NMR (700 MHz, $CDCl_3$)

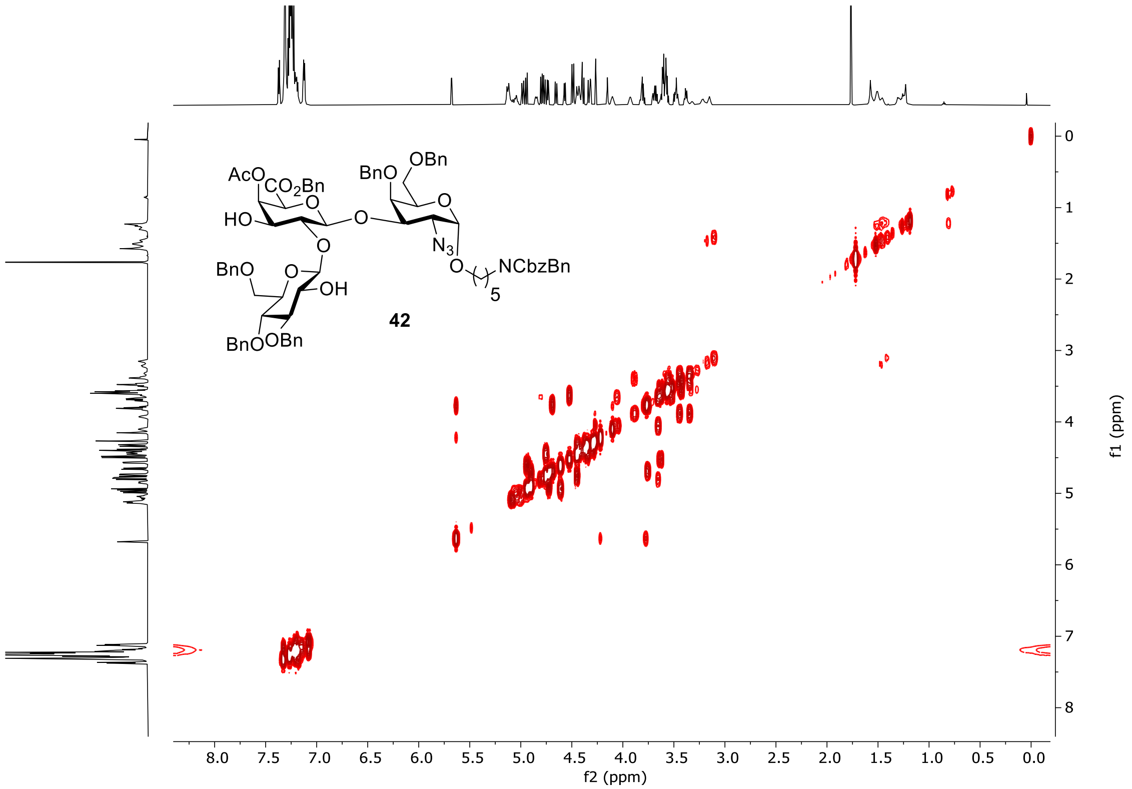

^{1}H-^{13}C HSQC NMR (700 MHz, $CDCl_3$)

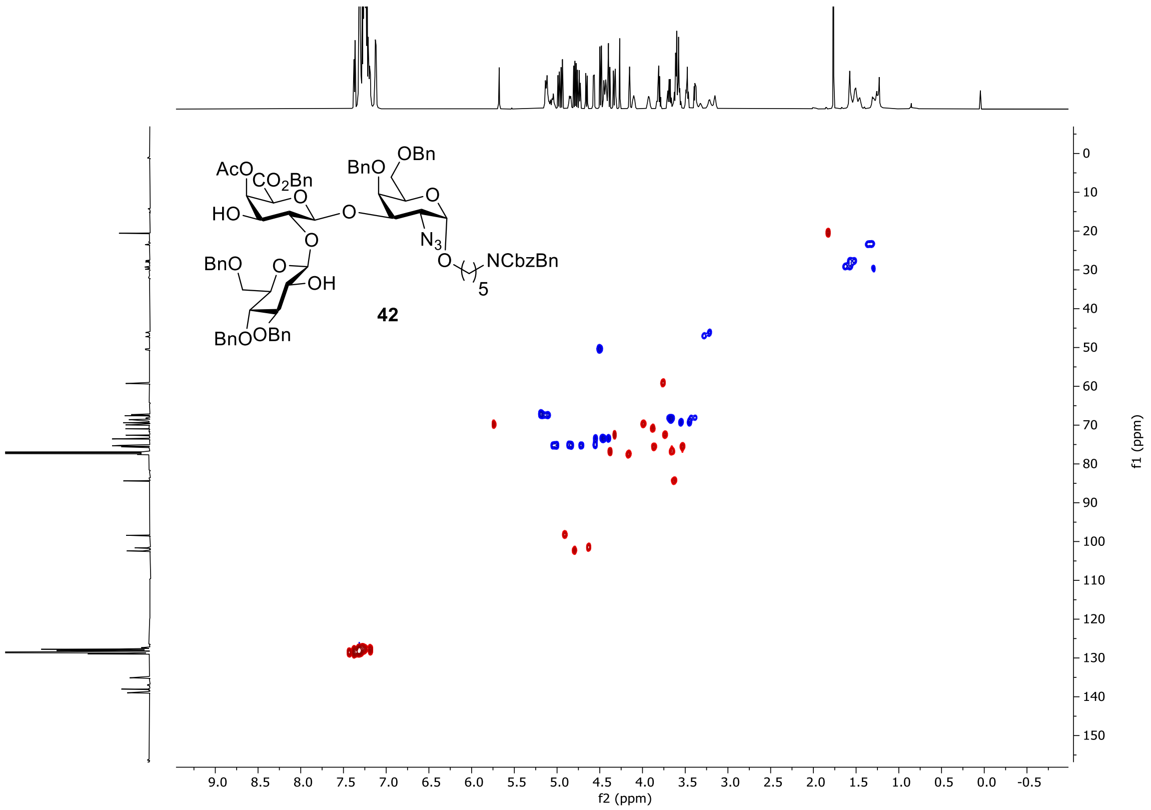

^{1}H-^{13}C Coupled HSQC NMR (700 MHz, $CDCl_3$)

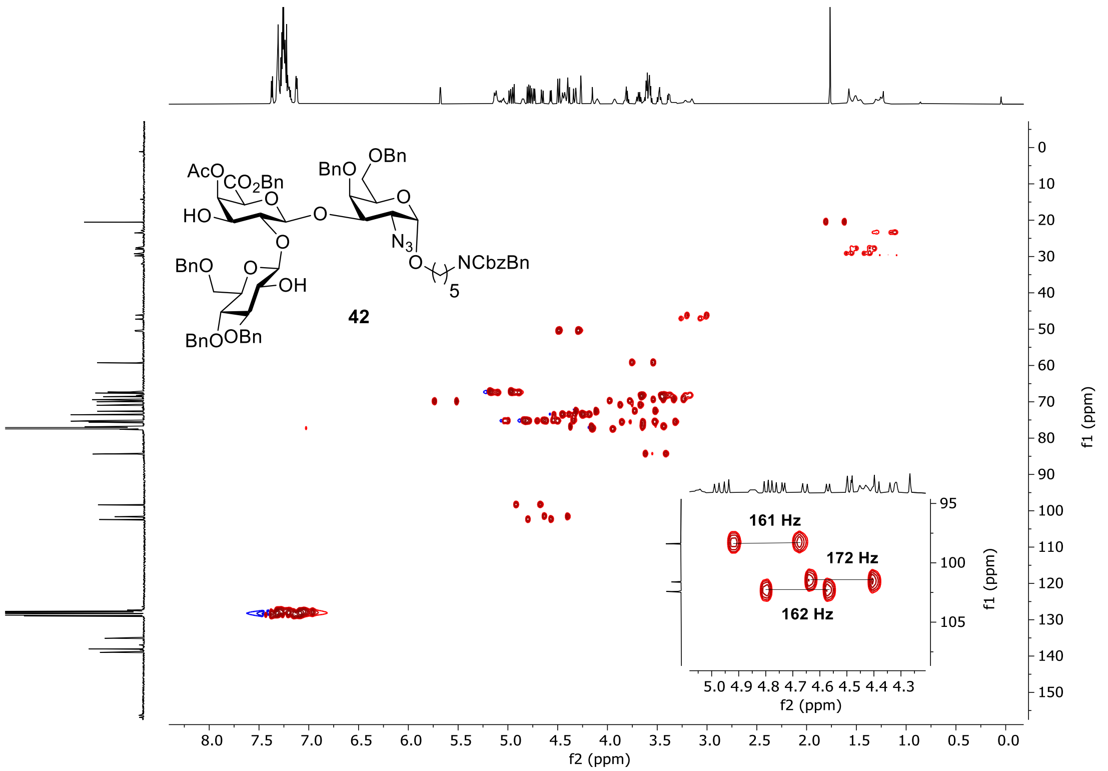

^{1}H NMR (700 MHz, $CDCl_3$)

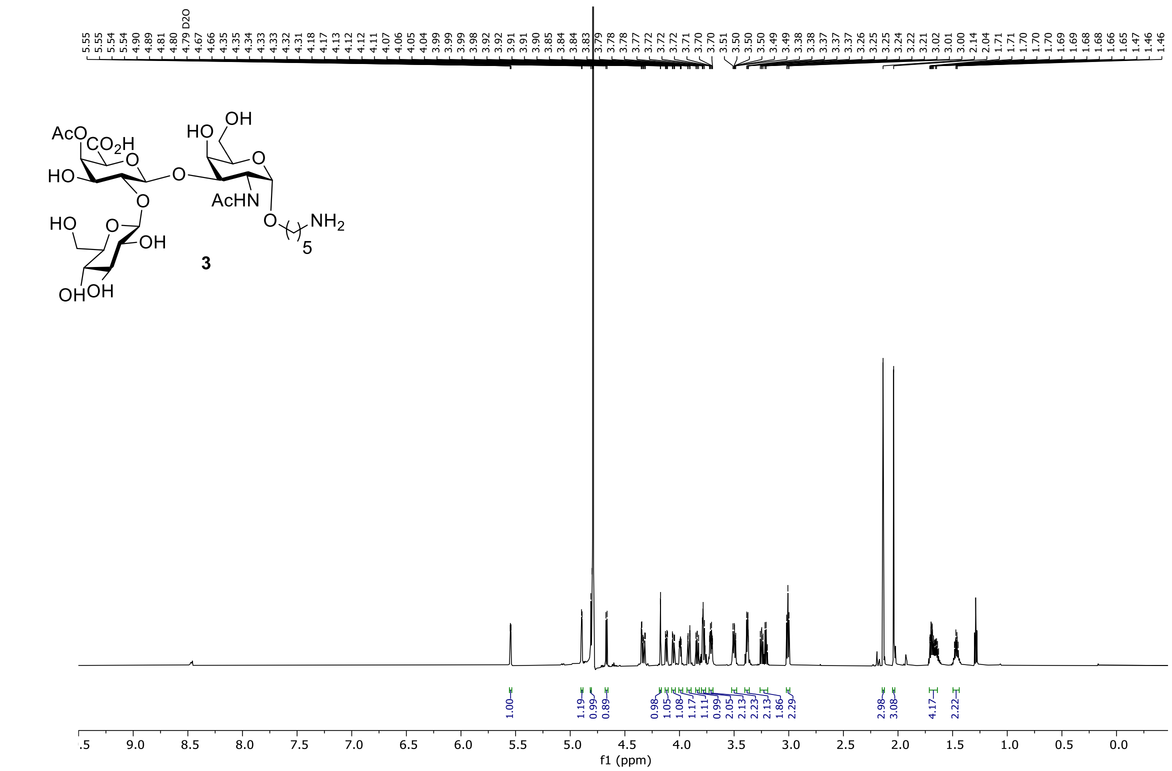

^{13}C NMR (176 MHz, $CDCl_3$)

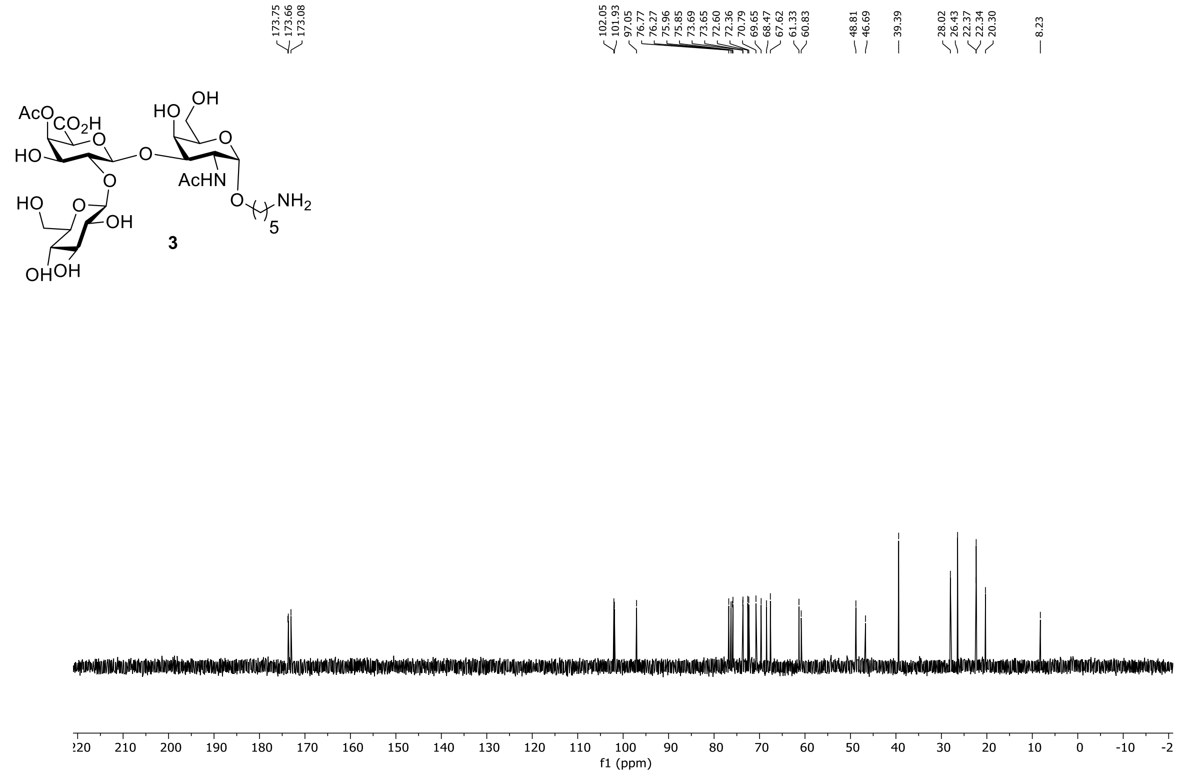

^1H-^1H COSY NMR (700 MHz, $CDCl_3$)

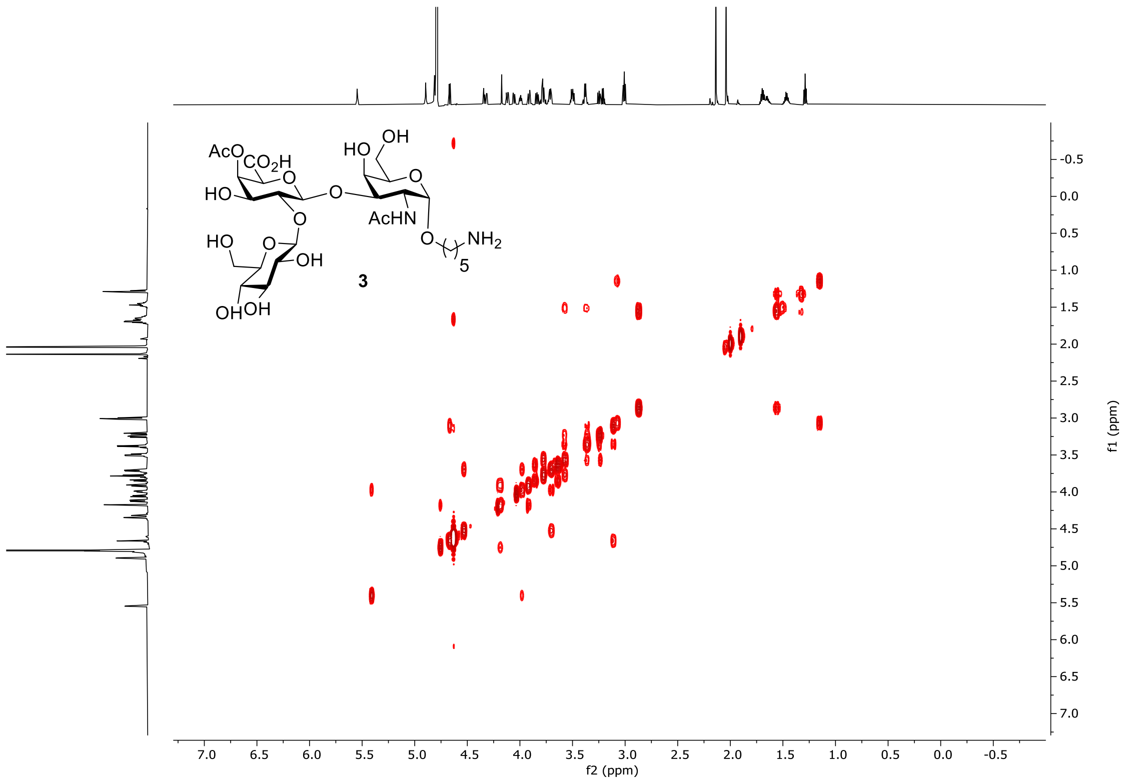

^1H-^{13}C HSQC NMR (700 MHz, $CDCl_3$)

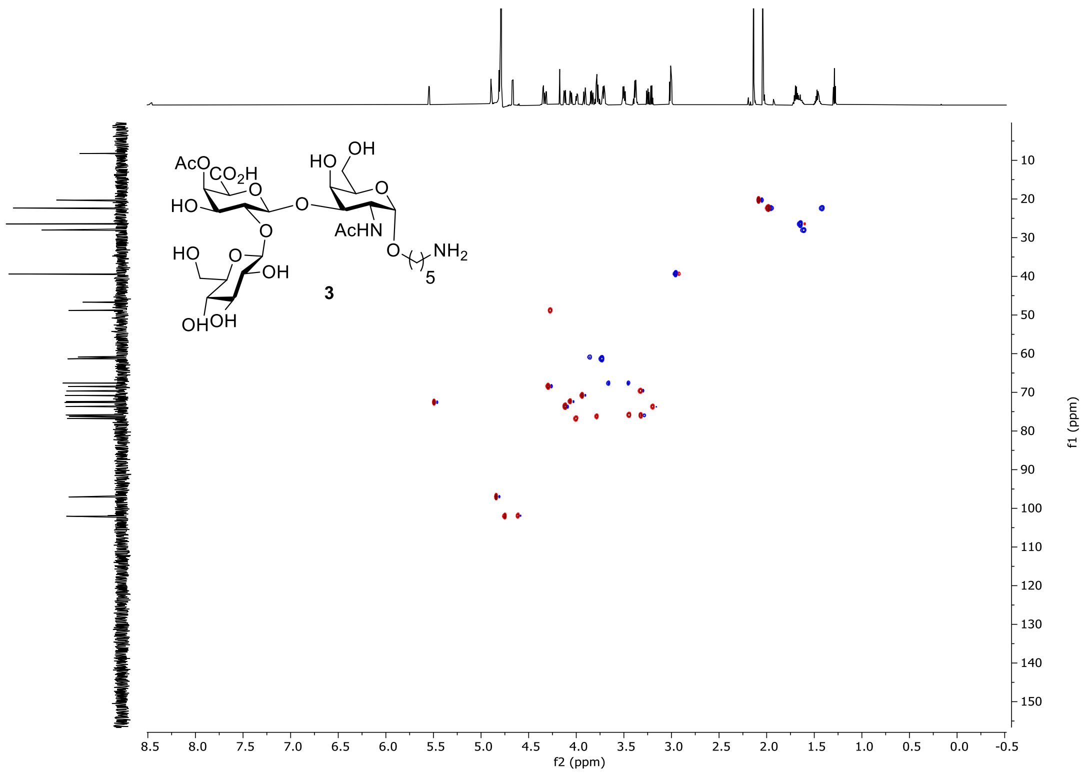

^{1}H-^{13}C Coupled HSQC NMR (700 MHz, $CDCl_3$)

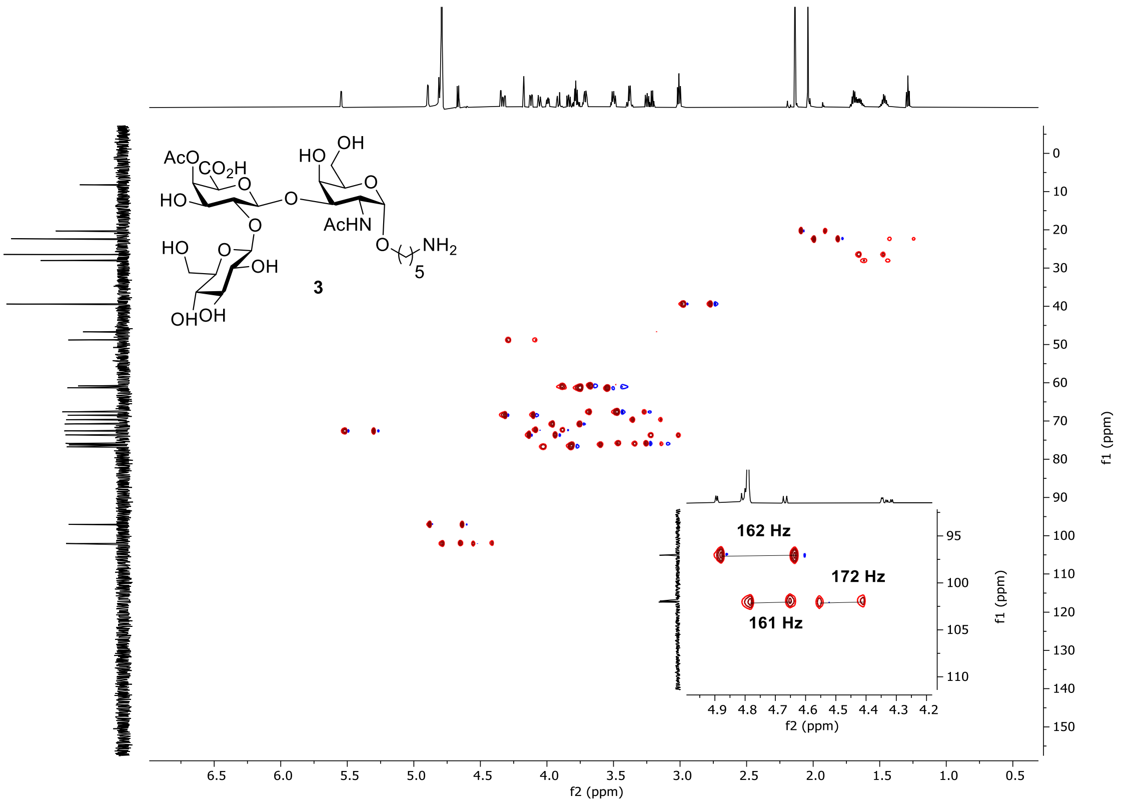

^{1}H NMR (400 MHz, $CDCl_3$)

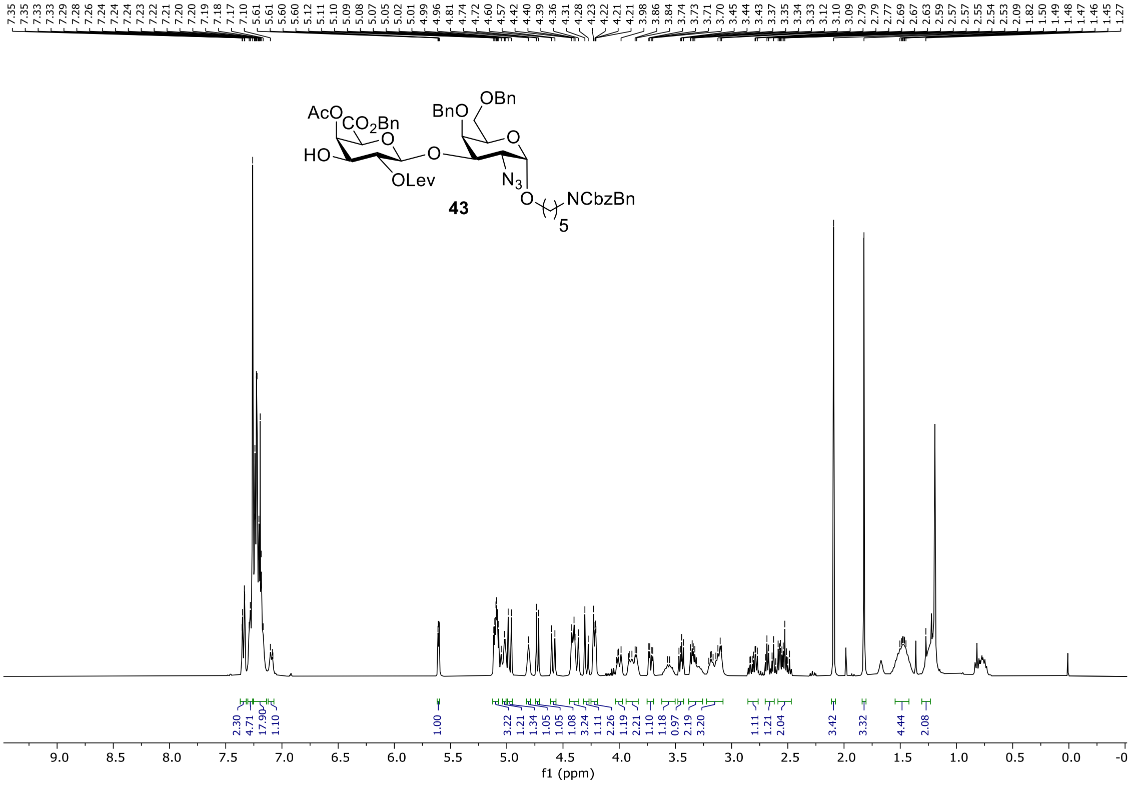

^{13}C NMR (101 MHz, $CDCl_3$)

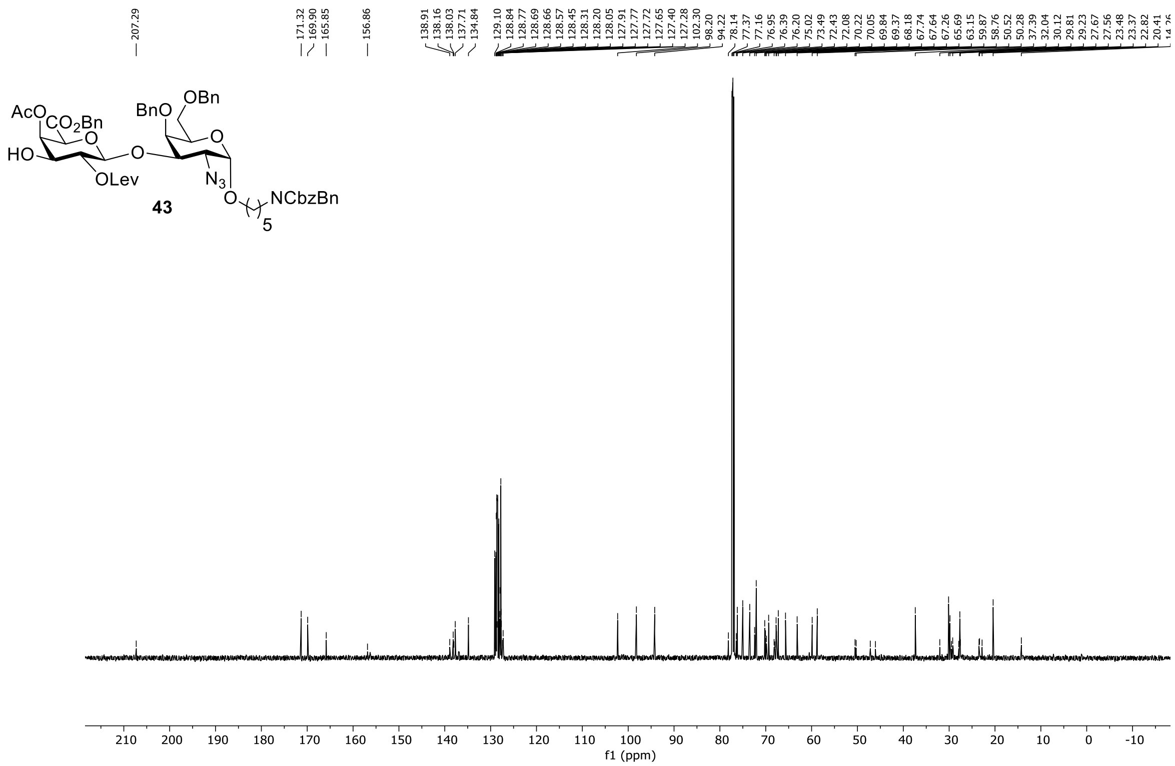

^{1}H-^{1}H COSY NMR (400 MHz, $CDCl_3$)

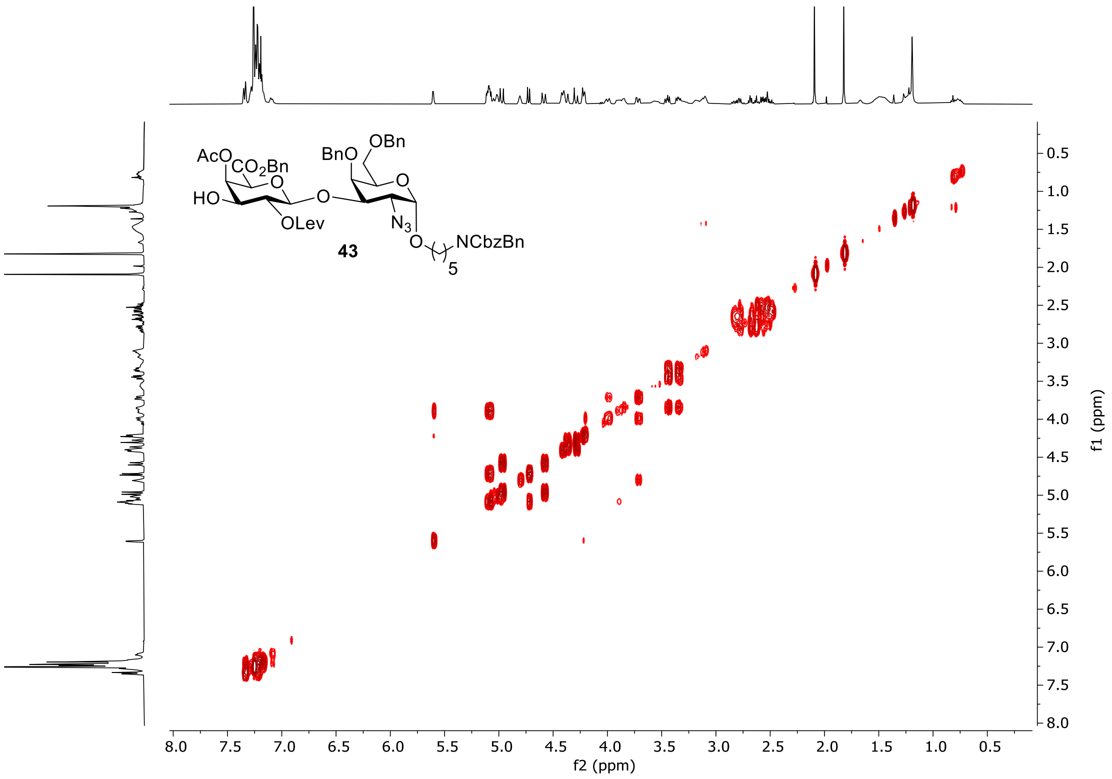

^{1}H-^{13}C HSQC NMR (400 MHz, $CDCl_3$)

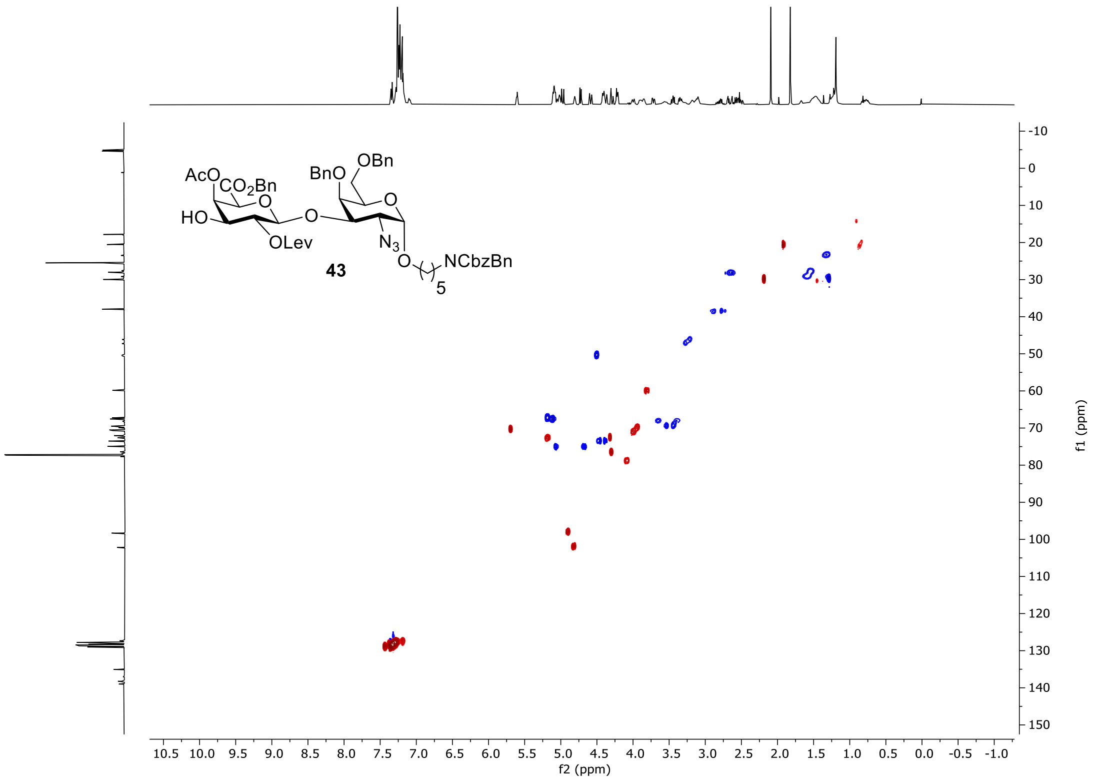

^{1}H-^{13}C Coupled HSQC NMR (400 MHz, $CDCl_3$)

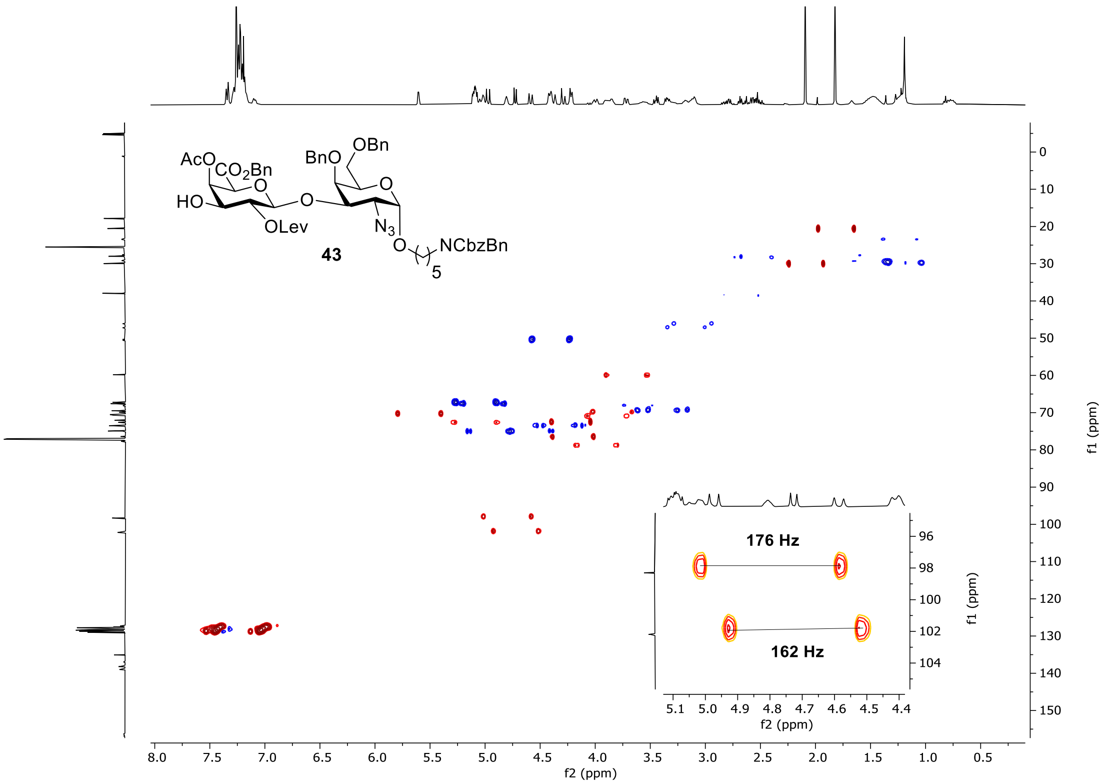

^{1}H NMR (400 MHz, $CDCl_3$)

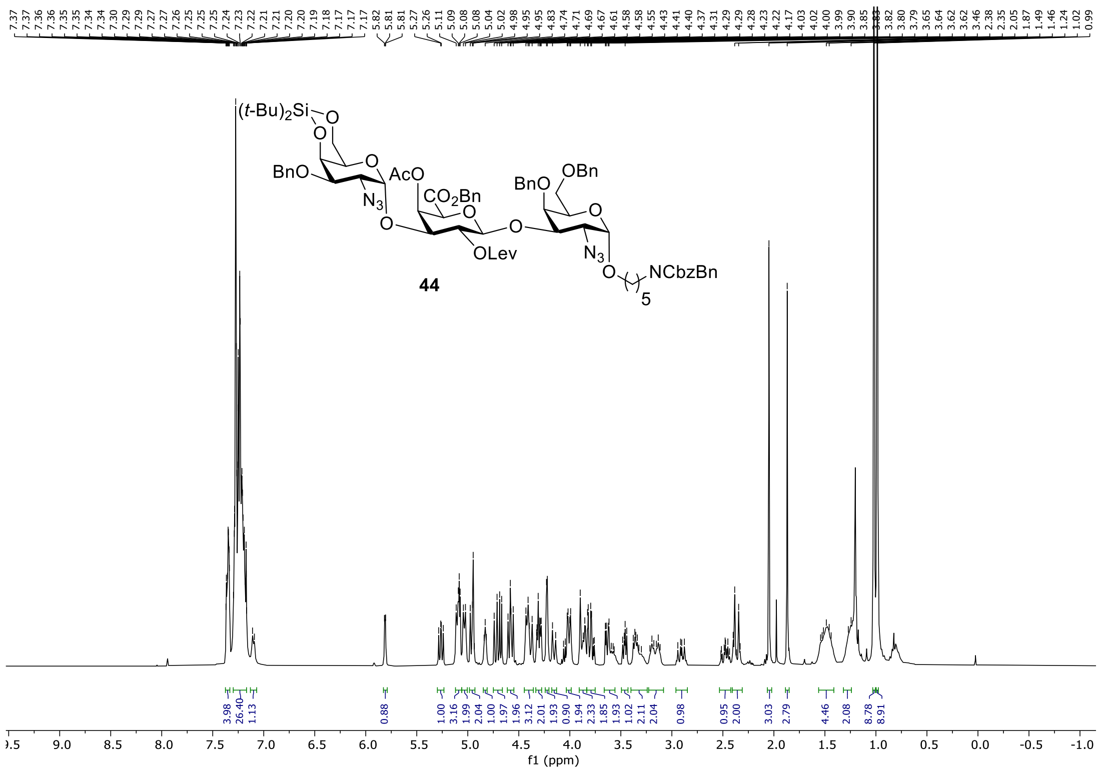

^{1}H-^{13}C NMR (101 MHz, $CDCl_3$)

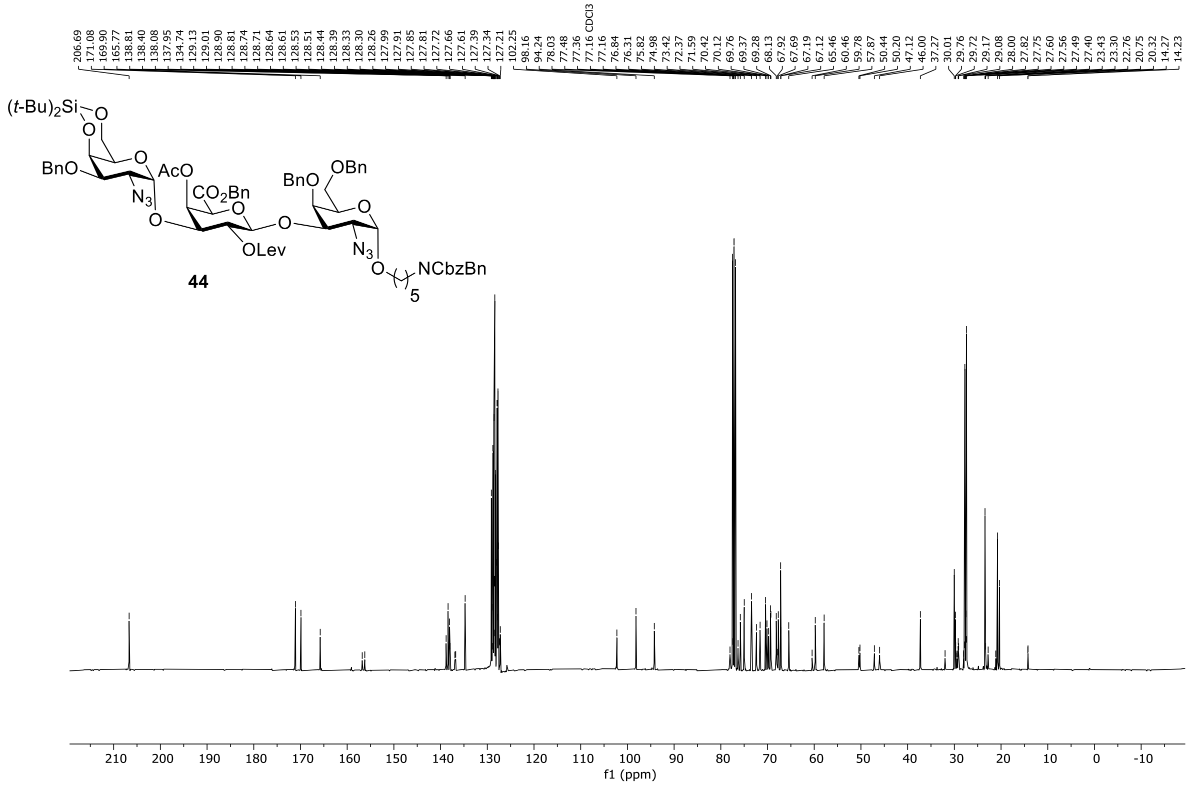

^{1}H-^{1}H COSY NMR (400 MHz, $CDCl_3$)

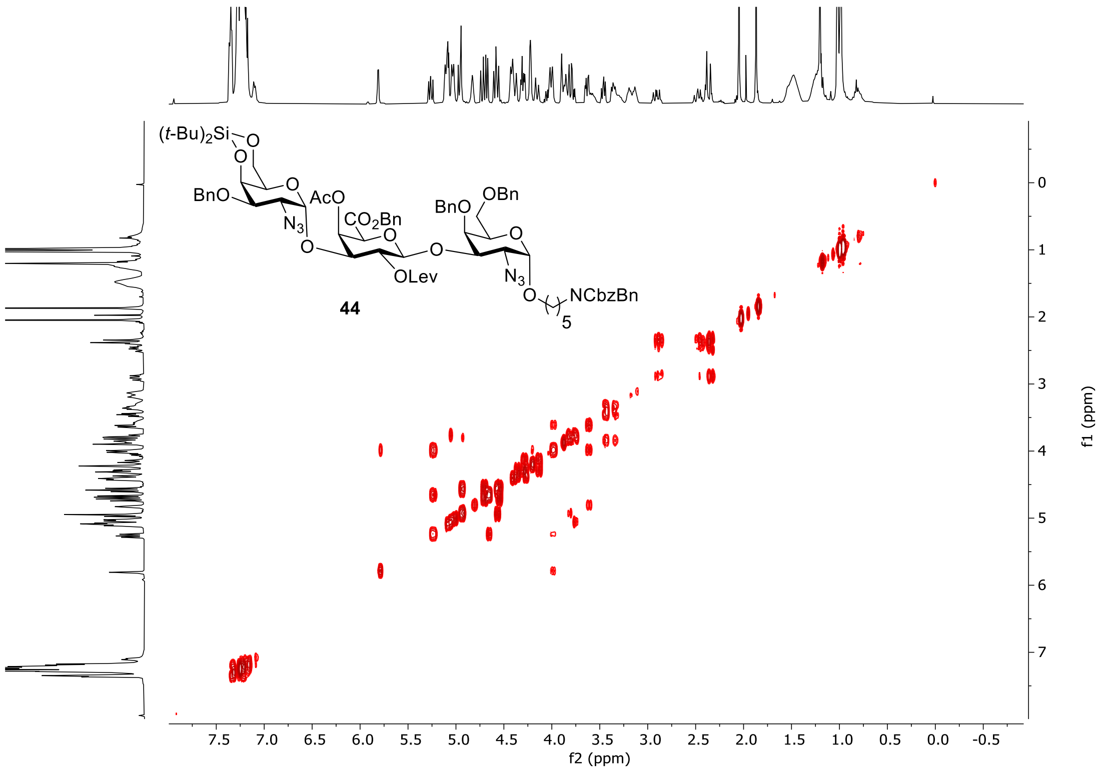

^{1}H-^{13}C HSQC NMR (400 MHz, $CDCl_3$)

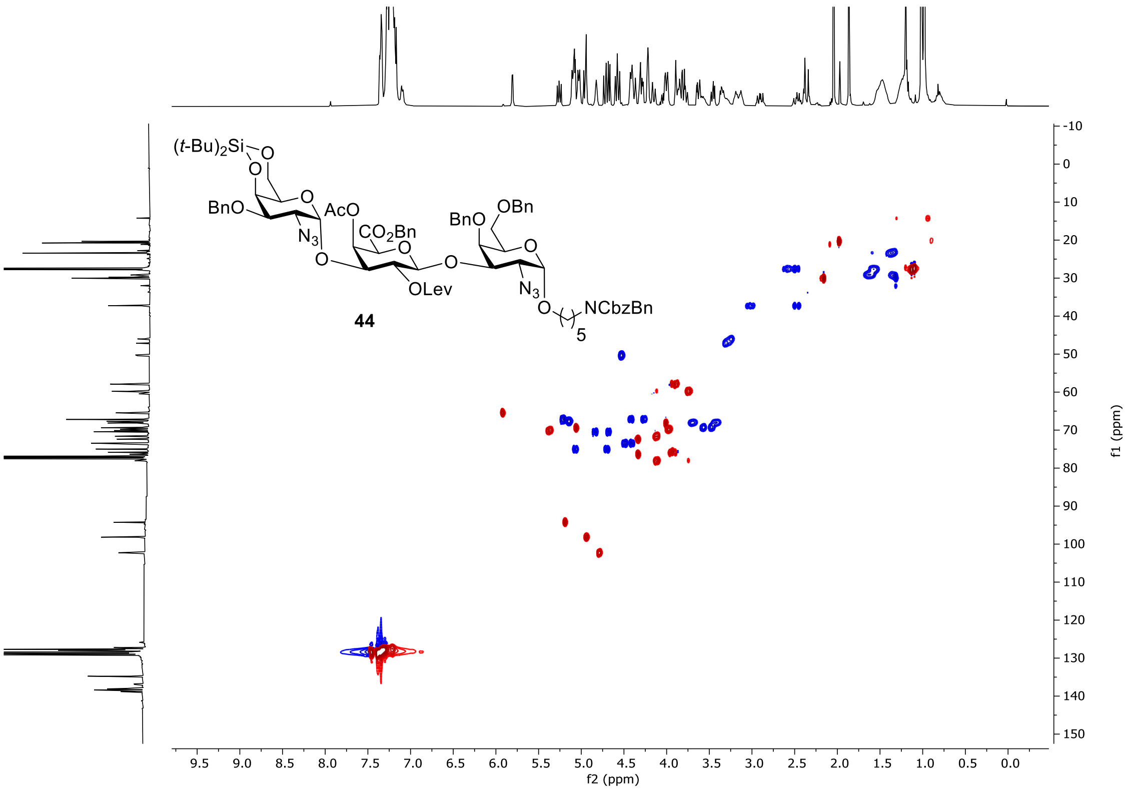

^{1}H NMR (400 MHz, $CDCl_3$)

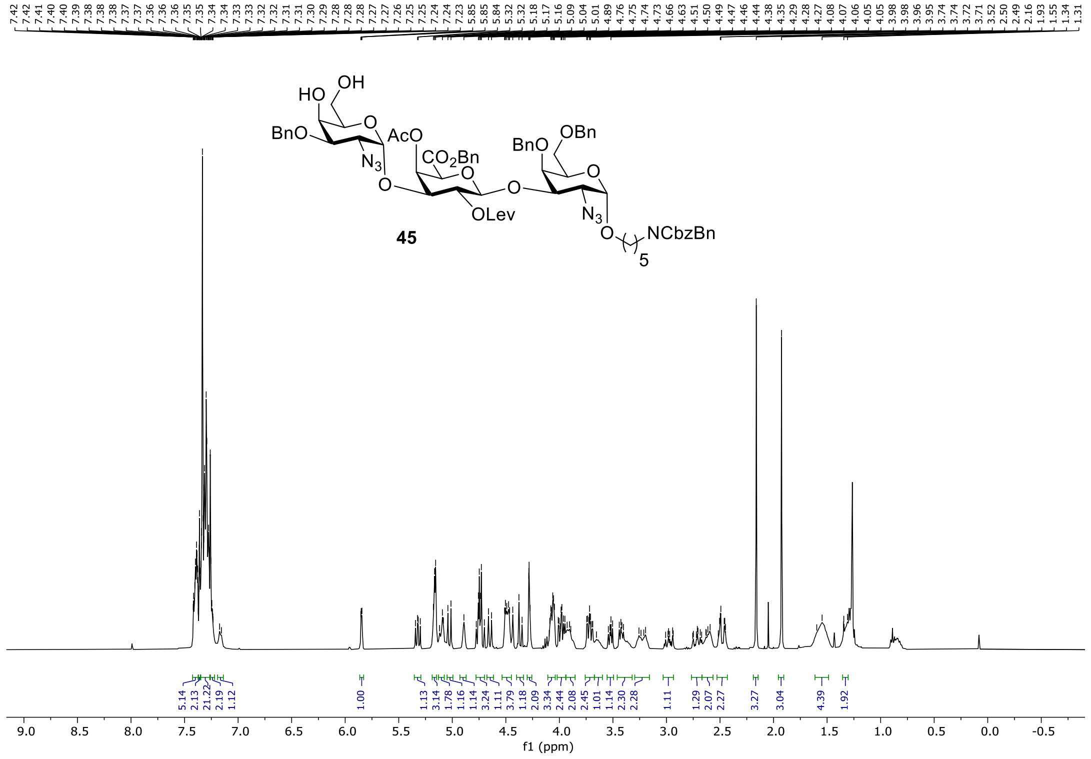

^{13}C NMR (101 MHz, $CDCl_3$)

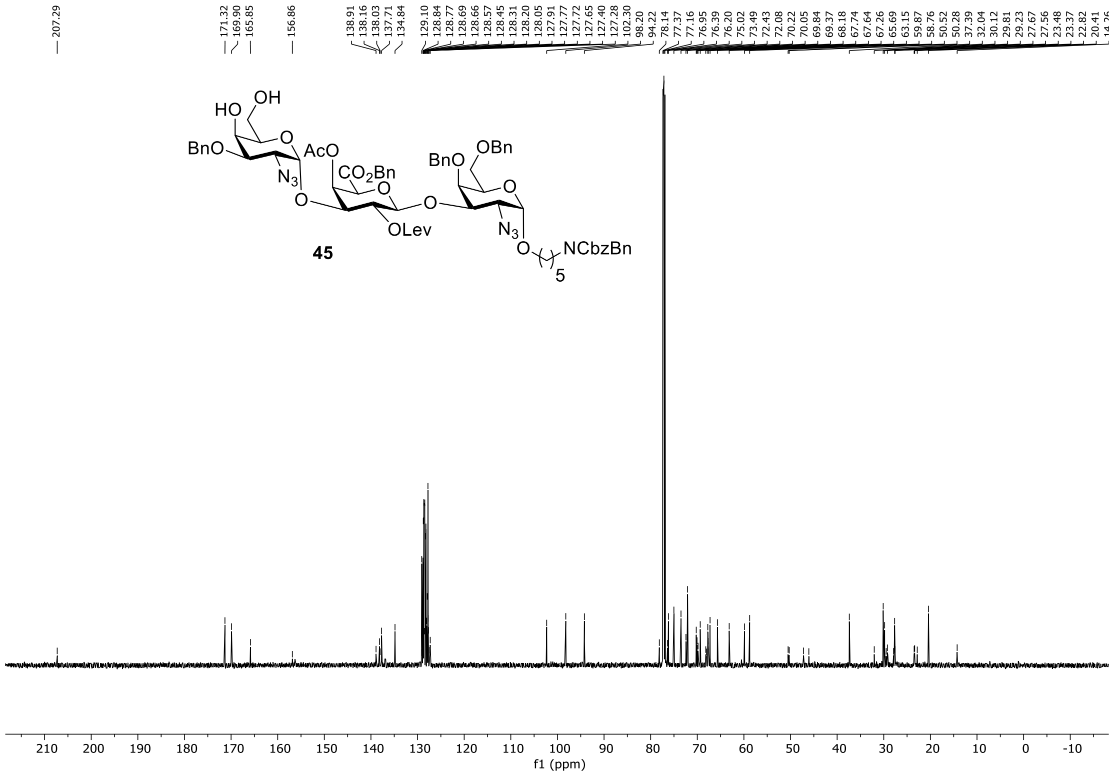

^{1}H-^{1}H COSY NMR (400 MHz, $CDCl_3$)

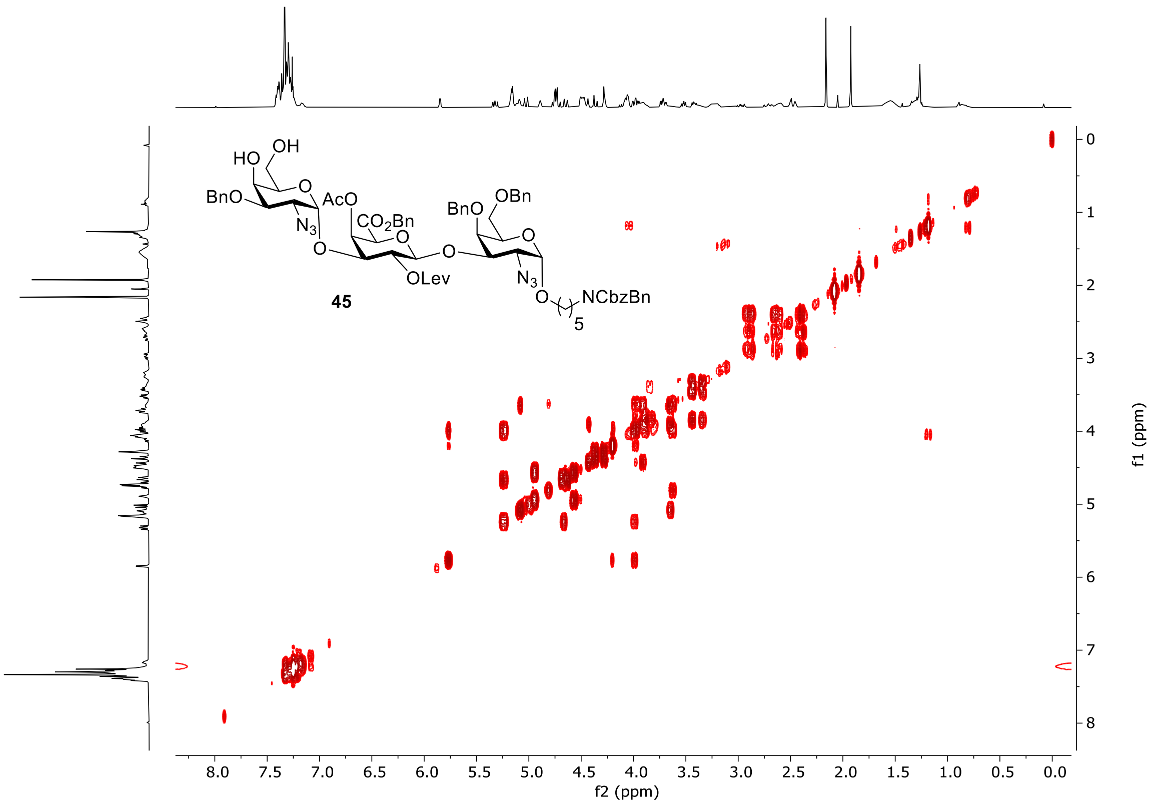

^{1}H-^{13}C HSQC NMR (400 MHz, $CDCl_3$)

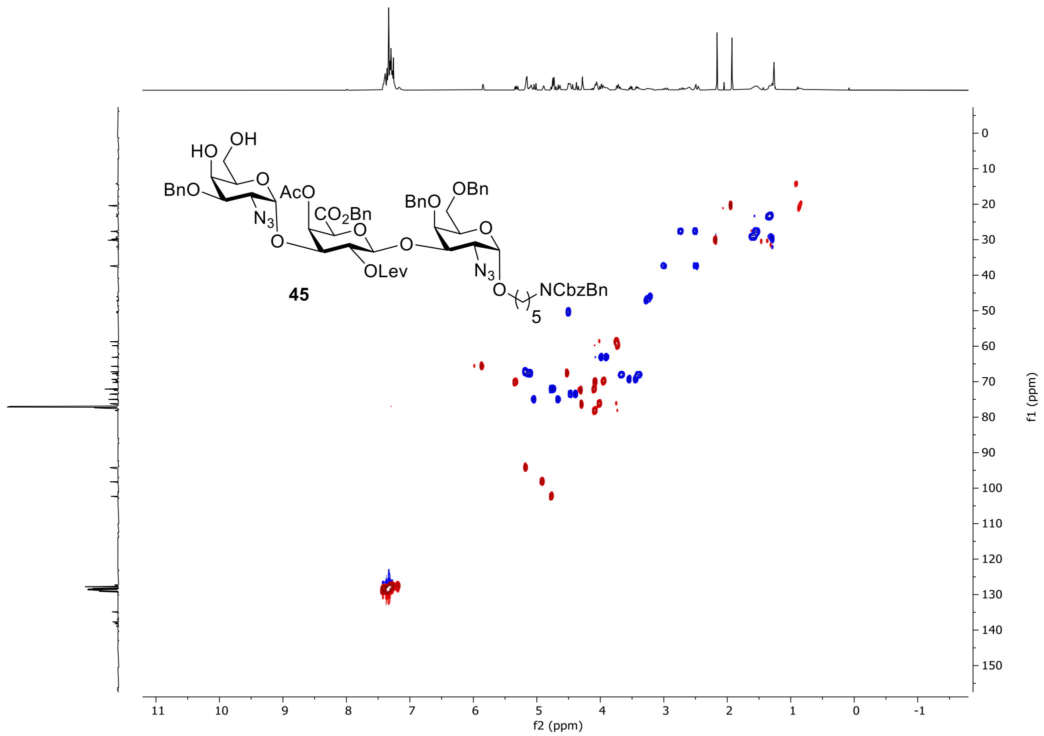

^{1}H-^{13}C Coupled HSQC NMR (400 MHz, $CDCl_3$)

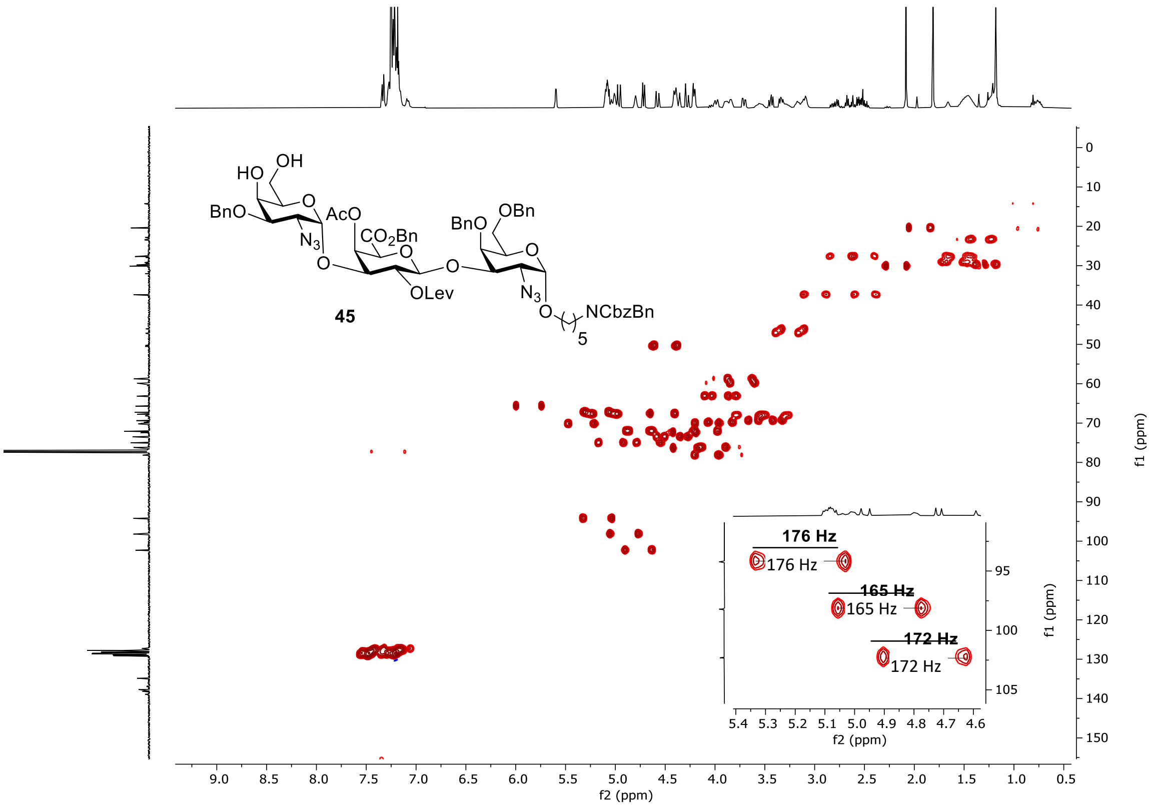

^{1}H NMR (700 MHz, $CDCl_3$)

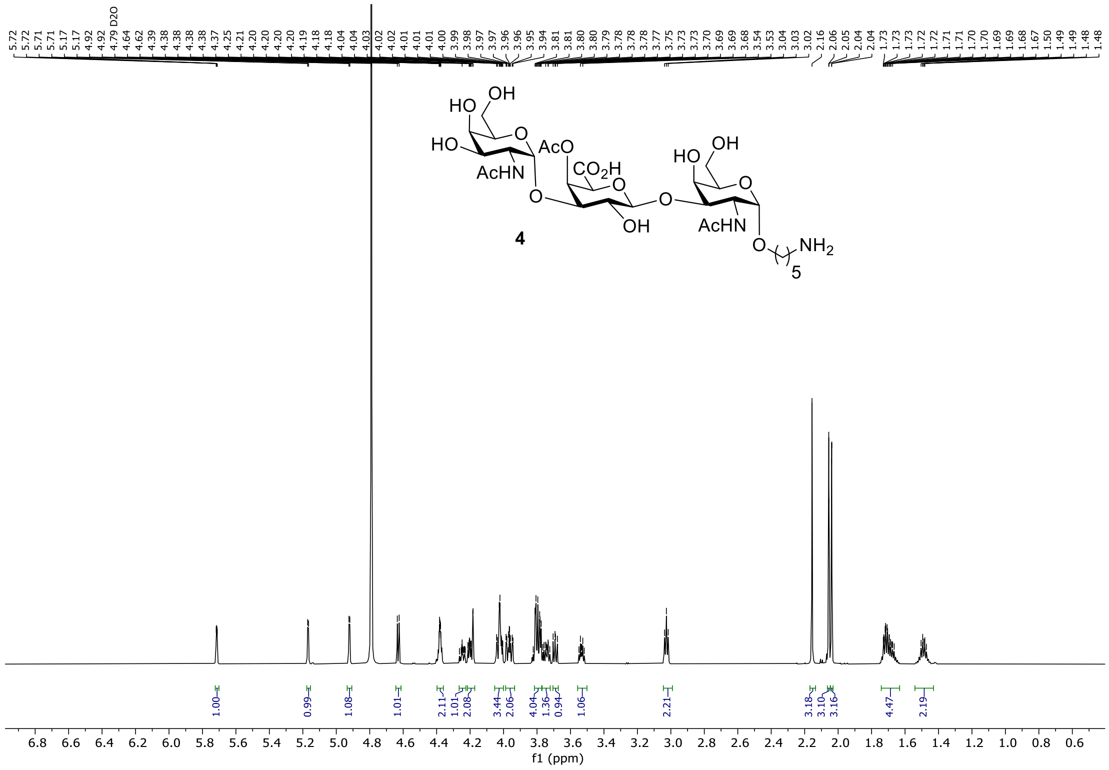

^{13}C NMR (176 MHz, $CDCl_3$)

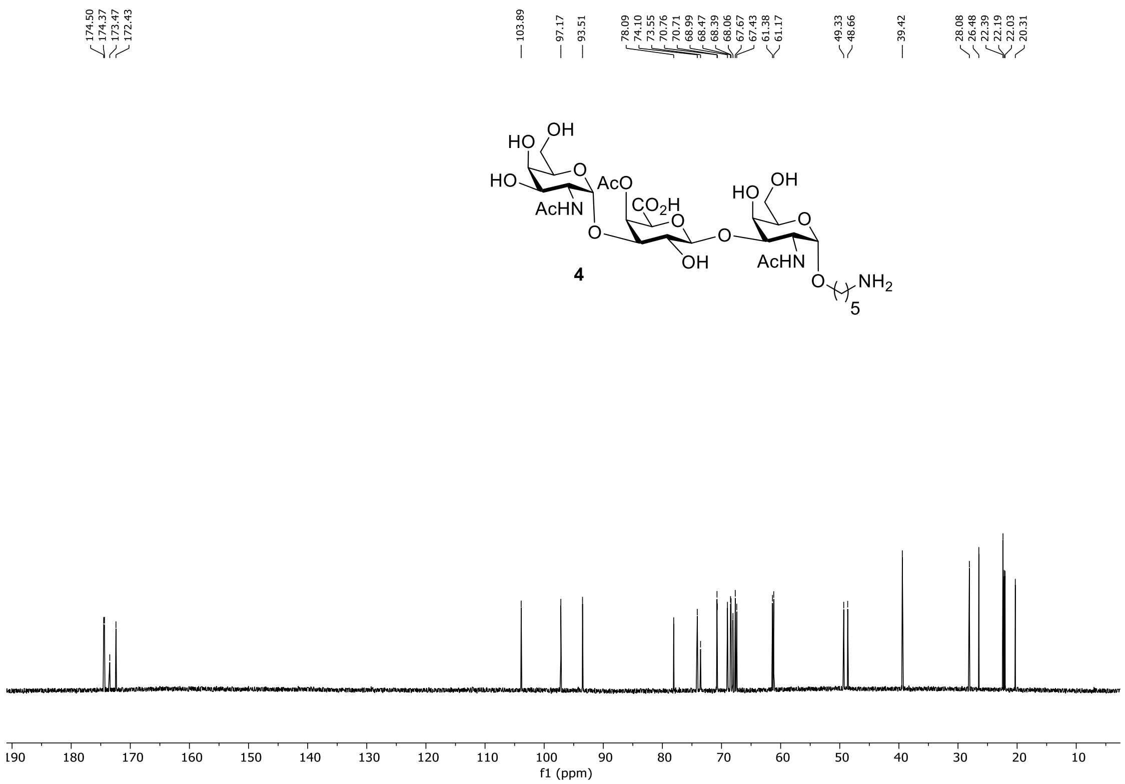

^1H-^{13}C HSQC NMR (700 MHz, $CDCl_3$)

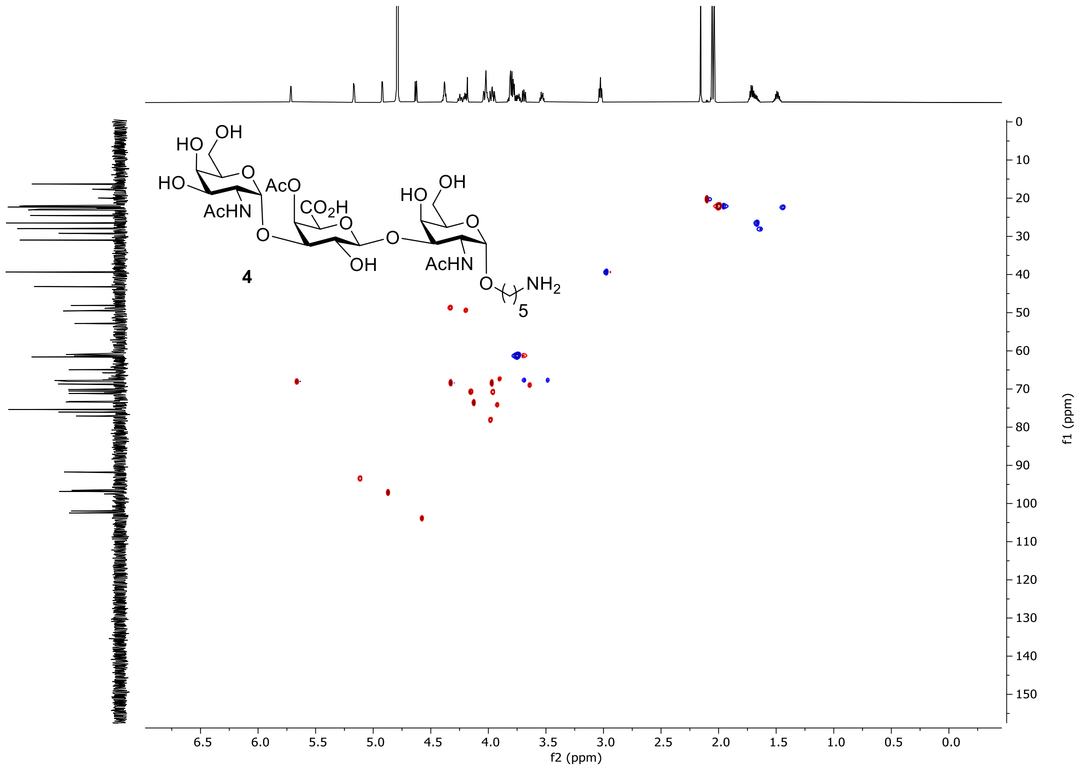

^1H-^{13}C Coupled HSQC NMR (700 MHz, $CDCl_3$)

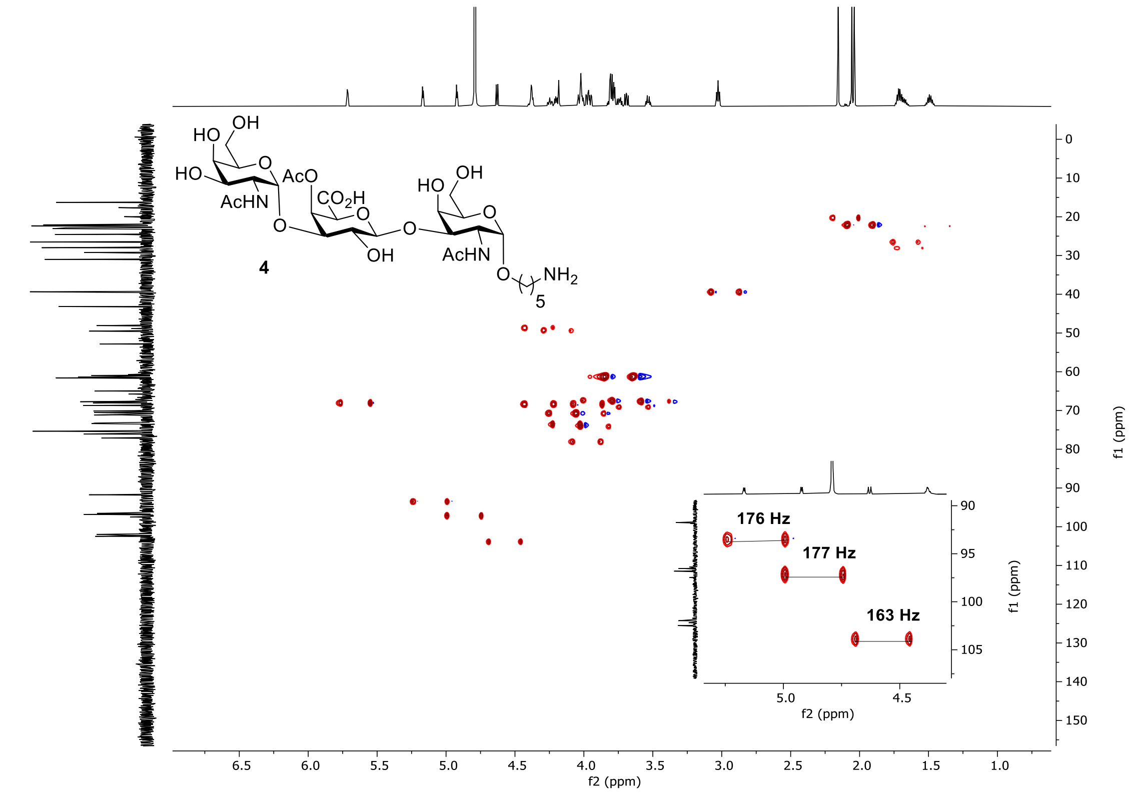

^1H-^1H COSY NMR (700 MHz, $CDCl_3$)

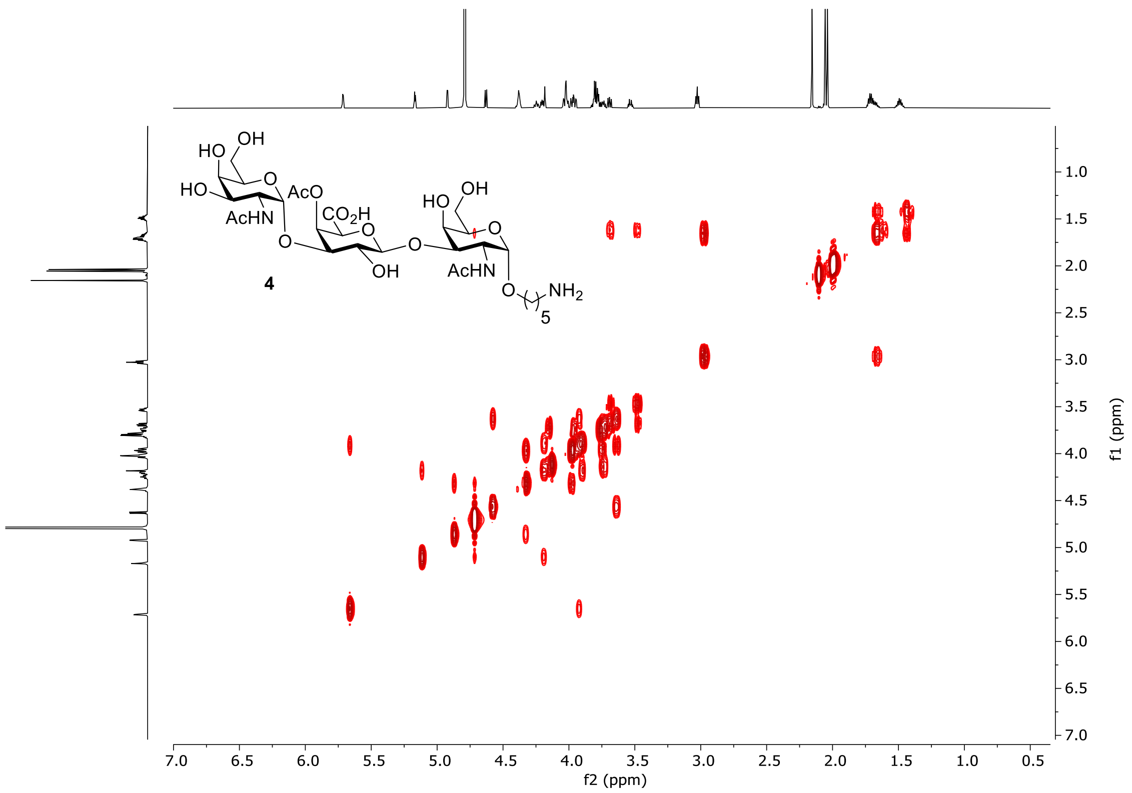

^{1}H NMR (400 MHz, $CDCl_3$)

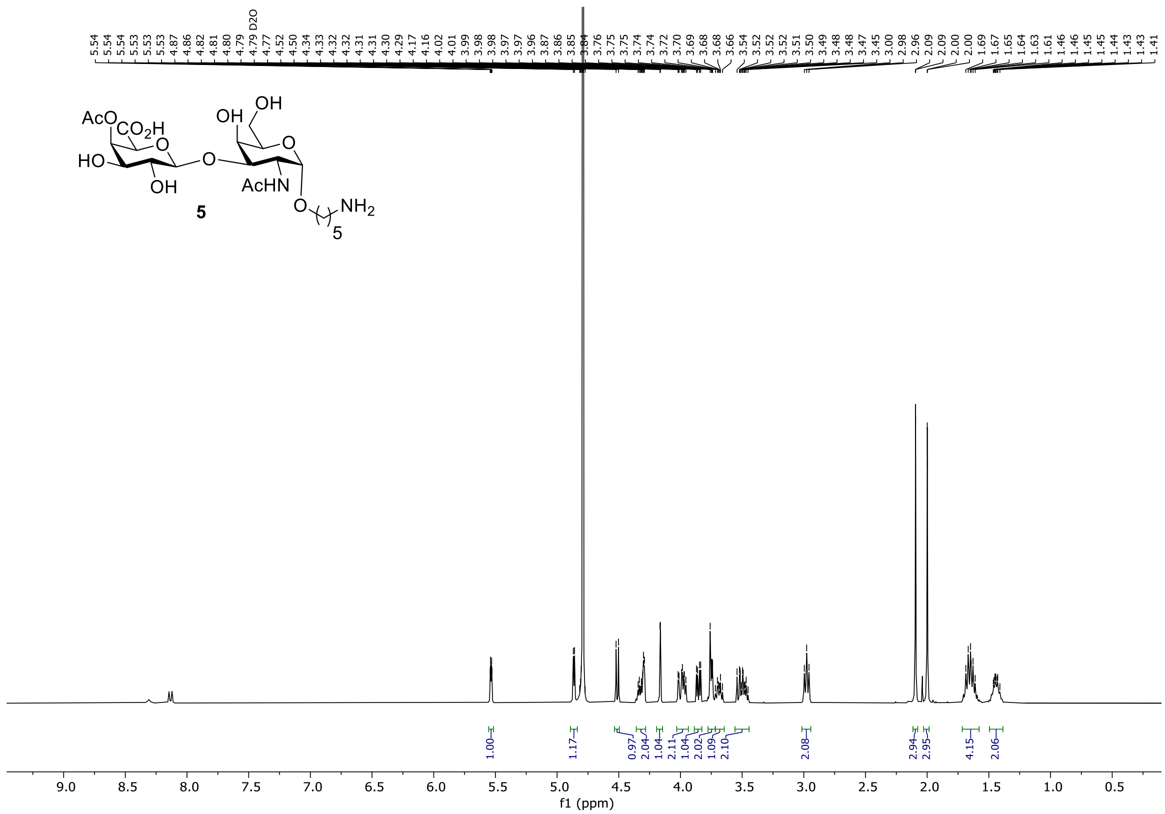

^{13}C NMR (101 MHz, $CDCl_3$)

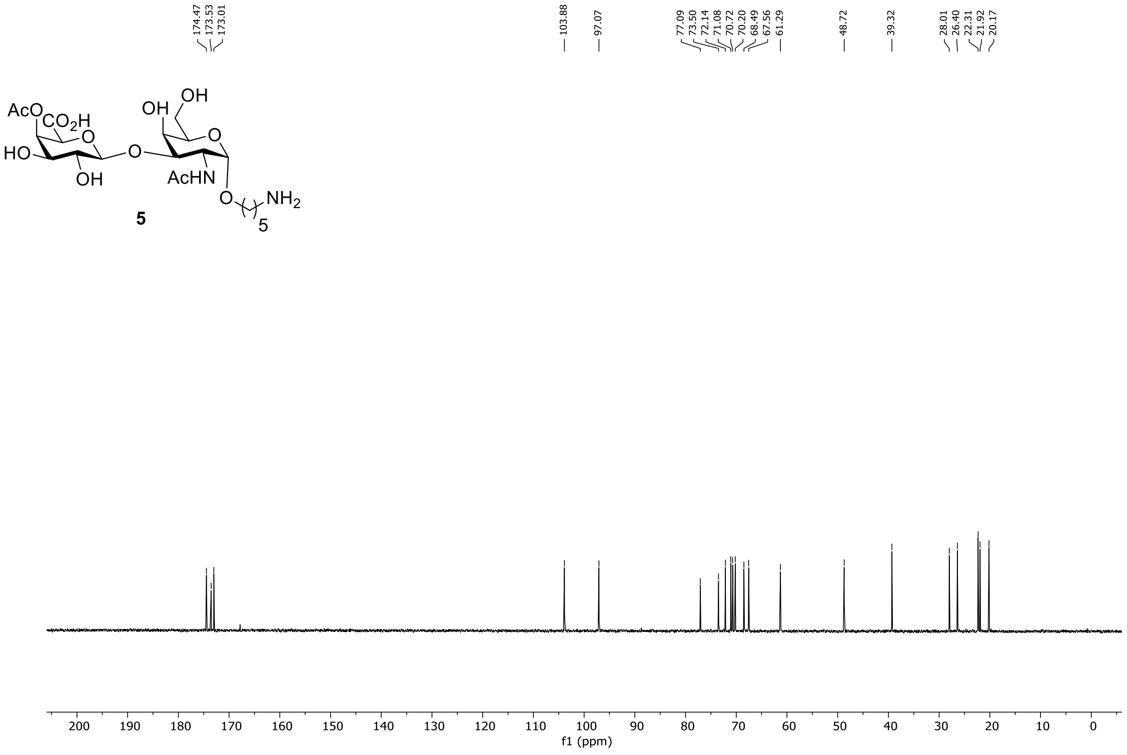

^{1}H-^{1}H COSY NMR (400 MHz, $CDCl_3$)

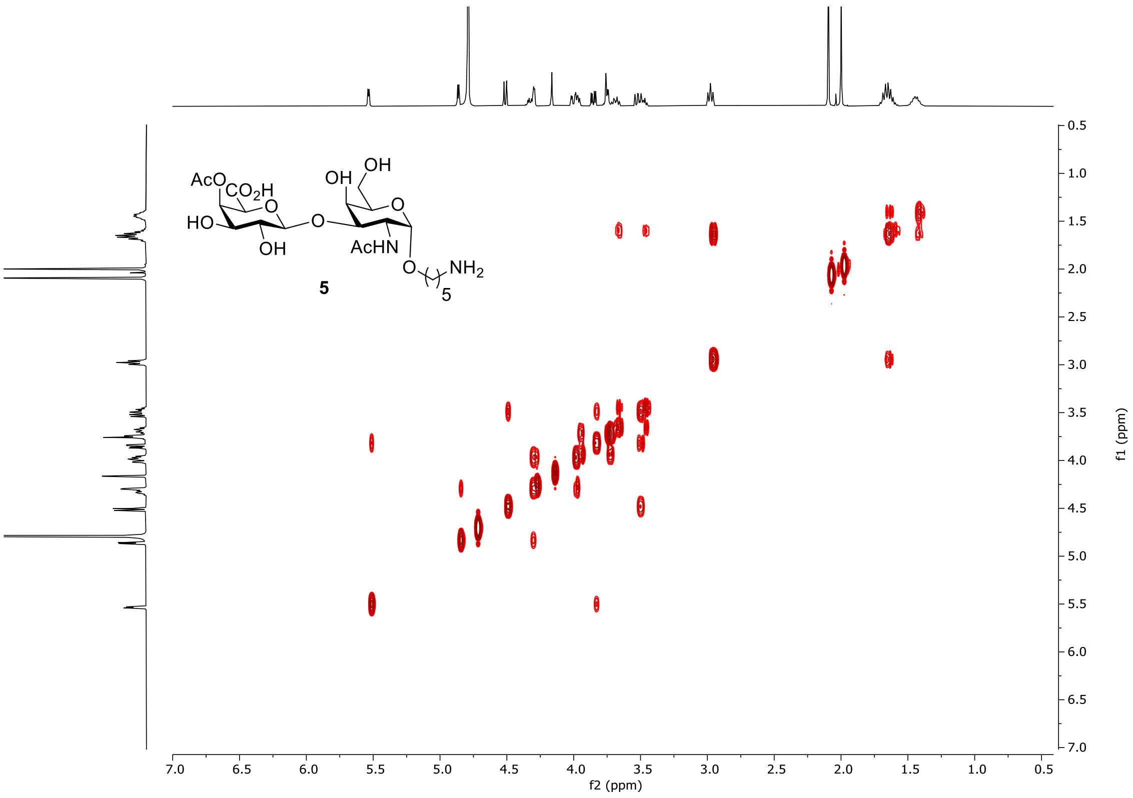

^{1}H-^{13}C HSQC NMR (400 MHz, $CDCl_3$)

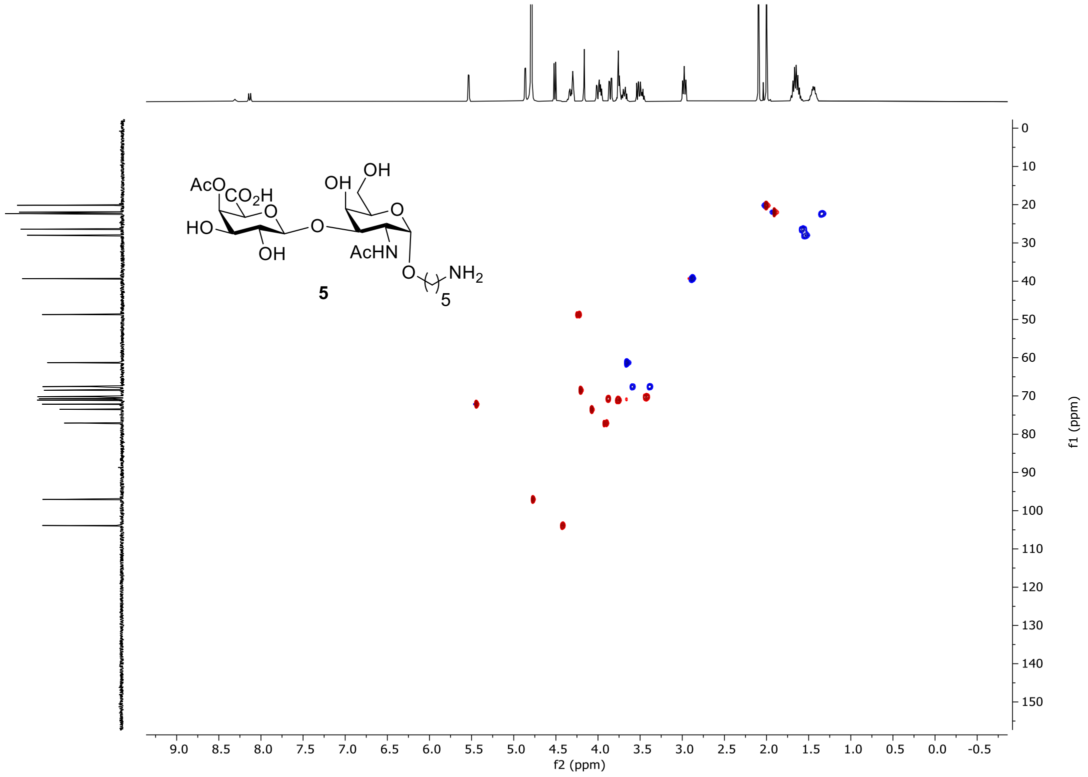

^{1}H-^{13}C Coupled HSQC NMR (400 MHz, $CDCl_3$)

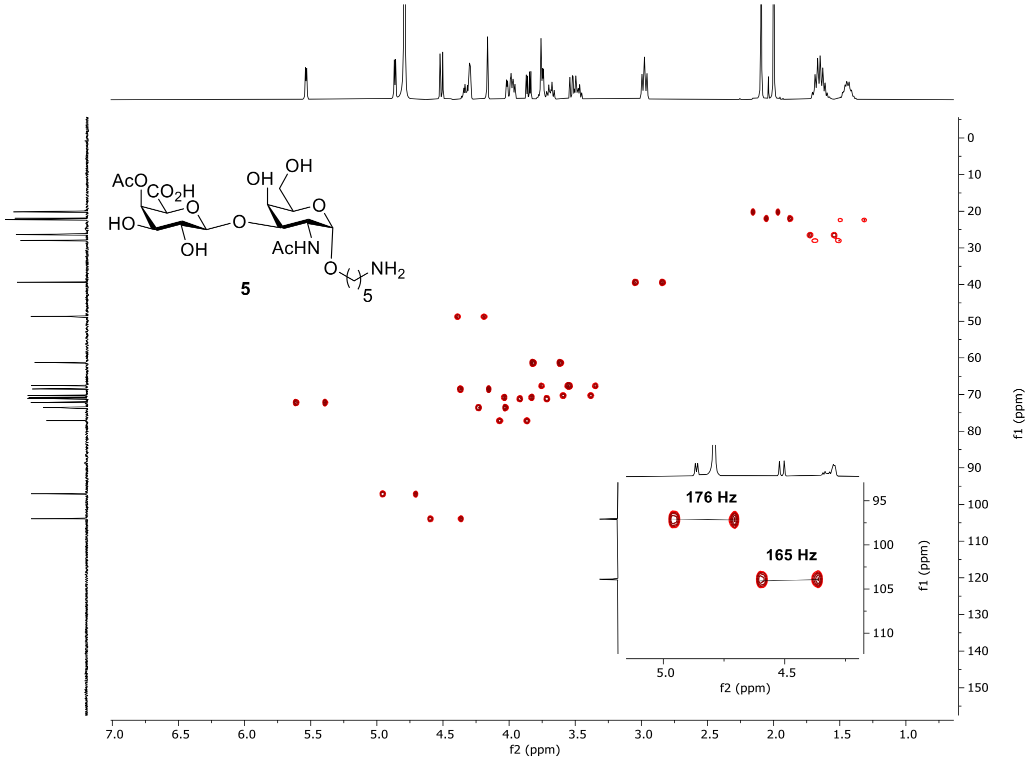

References:

(1) Daan; Nadia, A. G.; Diana, L.; Herman, S. O.; Johannes, H.; Gijsbert, A.; Jeroen, D. C. C., Synthesis of E. faecium wall teichoic acid fragments. *Bioorg. Med. Chem.* **2016,** *24*, 3893-3907.

(2) Ghosh, B.; Lai, Y.-H.; Shih, Y.-Y.; Pradhan, T. K.; Lin, C.-H.; Mong, K.-K. T., Total Synthesis of a Glycoglycerolipid from Meiothermus taiwanensis through a One-Pot Glycosylation Reaction and Exploration of its Immunological Properties. *Chem. Asian J.* **2013,** *8*, 3191-3199.
